# The SGLT2 inhibitor empagliflozin in patients hospitalized for acute heart failure: a multinational randomized trial

**DOI:** 10.1038/s41591-021-01659-1

**Published:** 2022-02-28

**Authors:** Adriaan A. Voors, Christiane E. Angermann, John R. Teerlink, Sean P. Collins, Mikhail Kosiborod, Jan Biegus, João Pedro Ferreira, Michael E. Nassif, Mitchell A. Psotka, Jasper Tromp, C. Jan Willem Borleffs, Changsheng Ma, Joseph Comin-Colet, Michael Fu, Stefan P. Janssens, Robert G. Kiss, Robert J. Mentz, Yasushi Sakata, Henrik Schirmer, Morten Schou, P. Christian Schulze, Lenka Spinarova, Maurizio Volterrani, Jerzy K. Wranicz, Uwe Zeymer, Shelley Zieroth, Martina Brueckmann, Jonathan P. Blatchford, Afshin Salsali, Piotr Ponikowski

**Affiliations:** 1 University of Groningen Department of Cardiology, University Medical Center Groningen, Groningen, The Netherlands; 2 Comprehensive Heart Failure Centre, University and University Hospital of Würzburg, Würzburg, Germany; 3 Section of Cardiology, San Francisco Veterans Affairs Medical Center and School of Medicine, University of California San Francisco, San Francisco, CA USA; 4 Department of Emergency Medicine, Vanderbilt University Medical Center and Geriatric Research and Education Clinical Care, Tennessee Valley Healthcare Facility VA Medical Center, Nashville, TN USA; 5 Saint Luke’s Mid America Heart Institute, Kansas City, MO USA; 6 School of Medicine, University of Missouri-Kansas City, Kansas City, MO USA; 7 George Institute for Global Health, Sydney, New South Wales Australia; 8 University of New South Wales, Sydney, New South Wales Australia; 9 Institute of Heart Diseases, Medical University, Wroclaw, Poland; 10 Université de Lorraine, Inserm INI-CRCT (Cardiovascular and Renal Clinical Trialists), Centre Hospitalier Régional Universitaire, Nancy, France; 11 Cardiovascular Research and Development Center, Department of Surgery and Physiology, Faculty of Medicine of the University of Porto, Porto, Portugal; 12 Inova Heart and Vascular Institute, Falls Church, VA USA; 13 Saw Swee Hock School of Public Health, National University of Singapore, and the National University Health System, Singapore, Singapore; 14 Haga Teaching Hospital, Den Haag, The Netherlands; 15 Department of Cardiology, Beijing Anzhen Hospital, Capital Medical University, Beijing, China; 16 Hospital Universitari de Bellvitge (IDIBELL), Barcelona, Spain; 17 Section of Cardiology, Sahlgrenska University Hospital, University of Gothenburg, Gothenburg, Sweden; 18 Department of Cardiovascular Sciences, Clinical Cardiology, Belgium University Hospital, Katholieke Universiteit Leuven, Leuven, Belgium; 19 Department of Cardiology, Military Hospital, Budapest, Hungary; 20 Duke Clinical Research Institute, Duke University Medical Center, Durham, NC USA; 21 Division of Cardiology, Duke University Medical Center, Durham, NC USA; 22 Department of Cardiovascular Medicine, Osaka University Graduate School of Medicine, Osaka, Japan; 23 Department of Cardiology, Division of Medicine, Akershus University Hospital, Lørenskog, Norway; 24 Department of Cardiology, Gentofte University Hospital Copenhagen, Copenhagen, Denmark; 25 University Hospital Jena, Jena, Germany; 26 First Department of Medicine, Masaryk University Hospital, Brno, Czech Republic; 27 Department of Cardiology, IRCCS San Raffaele Pisana, Rome, Italy; 28 Department of Electrocardiology, Medical University of Lodz, Central Clinical Hospital, Lodz, Poland; 29 Klinikum Ludwigshafen, Ludwigshafen, Germany; 30 Section of Cardiology, Max Rady College of Medicine, University of Manitoba, Winnipeg, Manitoba Canada; 31 Boehringer Ingelheim International GmbH, Ingelheim, Germany; 32 First Department of Medicine, Faculty of Medicine Mannheim, University of Heidelberg, Mannheim, Germany; 33 Elderbrook Solutions GmbH on behalf of Boehringer Ingelheim Pharma GmbH & Co. KG, Biberach, Germany; 34 Boehringer Ingelheim Pharmaceuticals Inc., Ridgefield, CT USA; 35 Faculty of Medicine, Rutgers University, New Brunswick, NJ USA

**Keywords:** Heart failure, Drug therapy

## Abstract

The sodium–glucose cotransporter 2 inhibitor empagliflozin reduces the risk of cardiovascular death or heart failure hospitalization in patients with chronic heart failure, but whether empagliflozin also improves clinical outcomes when initiated in patients who are hospitalized for acute heart failure is unknown. In this double-blind trial (EMPULSE; NCT04157751), 530 patients with a primary diagnosis of acute de novo or decompensated chronic heart failure regardless of left ventricular ejection fraction were randomly assigned to receive empagliflozin 10 mg once daily or placebo. Patients were randomized in-hospital when clinically stable (median time from hospital admission to randomization, 3 days) and were treated for up to 90 days. The primary outcome of the trial was clinical benefit, defined as a hierarchical composite of death from any cause, number of heart failure events and time to first heart failure event, or a 5 point or greater difference in change from baseline in the Kansas City Cardiomyopathy Questionnaire Total Symptom Score at 90 days, as assessed using a win ratio. More patients treated with empagliflozin had clinical benefit compared with placebo (stratified win ratio, 1.36; 95% confidence interval, 1.09–1.68; *P* = 0.0054), meeting the primary endpoint. Clinical benefit was observed for both acute de novo and decompensated chronic heart failure and was observed regardless of ejection fraction or the presence or absence of diabetes. Empagliflozin was well tolerated; serious adverse events were reported in 32.3% and 43.6% of the empagliflozin- and placebo-treated patients, respectively. These findings indicate that initiation of empagliflozin in patients hospitalized for acute heart failure is well tolerated and results in significant clinical benefit in the 90 days after starting treatment.

## Abstract (web summary)

In a multinational trial, empagliflozin has clinical benefit when administered to hospitalized patients with acute heart failure, extending the reach of SGLT2 inhibitor therapy to this patient population.

## Main

Acute heart failure is the most common cause of hospitalization in people older than 65 years and is associated with significant morbidity, mortality and poor quality of life^1–3^. Multiple randomized controlled trials testing pharmacological interventions in patients hospitalized for acute heart failure did not find improved post-discharge outcomes, highlighting a critical unmet need^4–8^.

The sodium–glucose cotransporter 2 (SGLT2) inhibitors empagliflozin and dapagliflozin significantly reduce the risk of cardiovascular death or hospitalization for heart failure in patients with chronic heart failure with a reduced left ventricular ejection fraction (LVEF)^9,10^. Empagliflozin additionally significantly reduces the risk of cardiovascular death or hospitalization for heart failure in patients with chronic heart failure with a preserved LVEF^11^. The combined SGLT1/2 inhibitor sotagliflozin has been shown to improve clinical outcomes in patients with diabetes and a recent worsening heart failure event (HFE)^12^. Whether the SGLT2 inhibitor empagliflozin provides clinical benefit in patients hospitalized for acute heart failure was unknown. In the early phase of hospital admission for heart failure, substantial fluid and electrolyte shifts as well as hemodynamic changes regularly occur. It remained uncertain whether it is safe to initiate treatment with an SGLT2 inhibitor in this phase. In addition, de novo heart failure patients were not eligible for inclusion in previous trials with SGLT2 inhibitors. Whether empagliflozin is effective and safe when started in patients with de novo hospitalization for acute heart failure who are not yet treated with background heart failure therapies remained to be established as well. We designed the present study to evaluate the effects of empagliflozin on three fundamental goals of care in patients hospitalized for acute heart failure: improvement of survival, reduction of HFEs, and improvement of symptoms.

## Results

### Patient characteristics

**Fig. 1:**
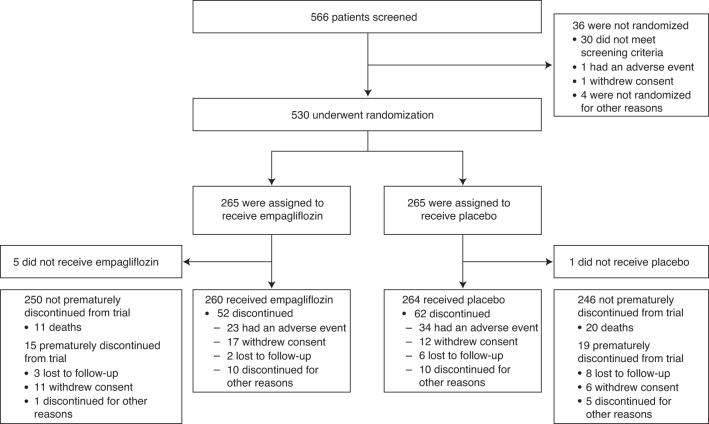
Screening, randomization, and follow-up. Flowchart of the double-blind EMPULSE trial (NCT04157751), in which 530 patients with a primary diagnosis of acute de novo or decompensated chronic heart failure, regardless of left ventricular ejection fraction, were randomly assigned to receive empagliflozin 10 mg once daily or placebo. This study was carried out at 118 centers in 15 countries.

From June 2020 to February 2021 a total of 566 patients were screened and 530 patients were randomly assigned to empagliflozin (*n* = 265) or placebo (*n* = 265) at 118 centers in 15 countries (Fig. 1 and Supplementary Table 1). Table 1 lists the baseline characteristics of the randomized patients. The median age was 71 years (interquartile range, 61–78 years), 34% were women, and 78% were white. The median time from hospital admission to randomization was 3 days (interquartile range, 2–4 days). Other patient characteristics and medications at baseline were balanced between treatment groups (Table 1).

**Table 1:** Characteristics of the patients at baseline

	Empagliflozin (*n* = 265) Median (IQR) or *n* (%)	Placebo (*n* = 265) Median (IQR) or *n* (%)
Age (years)	71 (62–78)	70 (59–78)
Sex		
Men	179 (67.5)	172 (64.9)
Women	86 (32.5)	93 (35.1)
Race or ethnic group		
White	211 (79.6)	202 (76.2)
Black	21 (7.9)	33 (12.5)
Asian	32 (12.1)	25 (9.4)
Other/mixed race	1 (0.4)	4 (1.5)
Missing	0	1 (0.4)
Geographic region		
Europe	168 (63.4)	171 (64.5)
North America	66 (24.9)	69 (26.0)
Asia	31 (11.7)	25 (9.4)
NYHA class		
I	8 (3.0)	6 (2.3)
II	95 (35.8)	91 (34.3)
III	134 (50.6)	145 (54.7)
IV	26 (9.8)	23 (8.7)
Missing	2 (0.8)	0
KCCQ-TSS	37.5 (20.8–58.3)	39.6 (22.4–58.3)
NT-proBNP (pg ml^−1^)	3,299 (1,843–6,130)	3,106 (1,588–6,013)
Blood pressure (mmHg)		
Systolic	120 (109.0–135.0)	122 (110.0–138.0)
Diastolic	72.0 (64.0–82.0)	74.0 (67.0–80.0)
Body mass index (kg m^−2^)	28.35 (24.54–32.46)	29.08 (24.69–33.60)
Left ventricular ejection fraction (%)	31.0 (23.0-45.0)	32.0 (22.5–49.0)
≤40%	182 (68.7)	172 (64.9)
>40%	76 (28.7)	93 (35.1)
Missing	7 (2.6)	0
Estimated GFR (ml min^−1^ 1.73 m^−2^)	50.0 (36.0–65.0)	54.0 (39.0–70.0)
<30 ml min^−1^ 1.73 m^−^^2^	27 (10.2)	24 (9.1)
Missing	16 (6.0)	14 (5.3)
Hemoglobin (g dl^−1^)	13.2 (11.8–14.8)	13.4 (11.8–14.8)
Medical history		
Diabetes	124 (46.8)	116 (43.8)
Hypertension	205 (77.4)	221 (83.4)
Myocardial infarction	66 (24.9)	62 (23.4)
Atrial fibrillation	134 (50.6)	128 (48.3)
CABG or PCI	78 (29.4)	78 (29.4)
Valvular heart disease	173 (65.3)	167 (63.0)
Heart failure status		
Decompensated CHF	177 (66.8)	178 (67.2)
Acute de novo	88 (33.2)	87 (32.8)
Medication		
ACE inhibitor and/or ARB and/or ARNi	186 (70.2)	185 (69.8)
ACE inhibitor	88 (33.2)	89 (33.6)
ARB	64 (24.2)	52 (19.6)
ARNi	36 (13.6)	45 (17.0)
MRA	151 (57.0)	125 (47.2)
Beta-blocker	213 (80.4)	208 (78.5)
Loop diuretic	233 (87.9)	204 (77.0)

ACE, angiotensin-converting enzyme; ARB, angiotensin receptor blocker; ARNi, angiotensin receptor neprilysin inhibitor; CABG, coronary artery bypass graft; CHF, chronic heart failure; GFR, glomerular filtration rate; MRA, mineralocorticoid receptor antagonist; NYHA, New York Heart Association; PCI, percutaneous coronary intervention.

### Follow-up

A total of 530 patients were included in the efficacy analyses using the intention-to-treat principle. Five hundred and twenty-four patients received at least one dose of the trial drug (260 in the empagliflozin group and 264 in the placebo group, Fig. 1). These patients were subsequently included in the safety analyses. Early discontinuation of the trial drug occurred in 114 patients (21.8%): 52 (20.0%) in the empagliflozin group and 62 patients (23.5%) in the placebo group. Eleven patients (2.1%) were lost to follow-up.

### Primary outcome

**Fig. 2:**
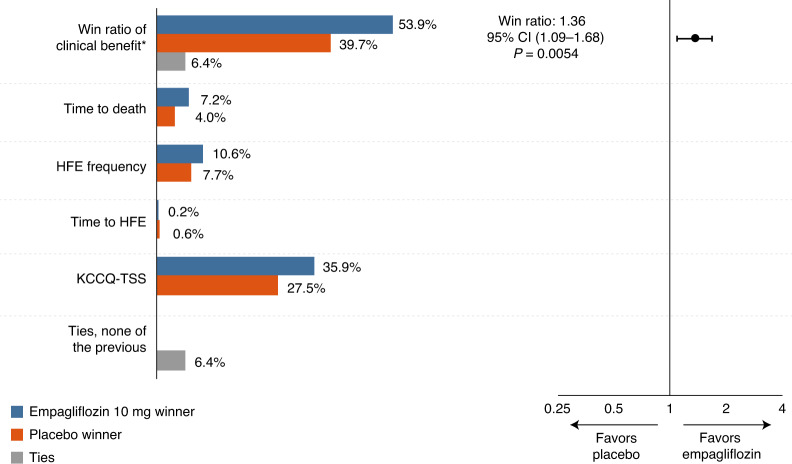
Primary efficacy outcome and components. The stratified win ratio was calculated using a non-parametric generalized pairwise comparison within heart failure status strata; data are presented as the point estimate and 95% CI with a two-sided *P* value. For the components of the win ratio, the percentages do not reflect randomized comparisons. Please refer to Table 2 for the overall number of events and KCCQ-TSS data. *Hierarchical composite of death, number of HFEs, time to first HFE and change from baseline in KCCQ-TSS after 90 days of treatment.

**Fig. 3:**
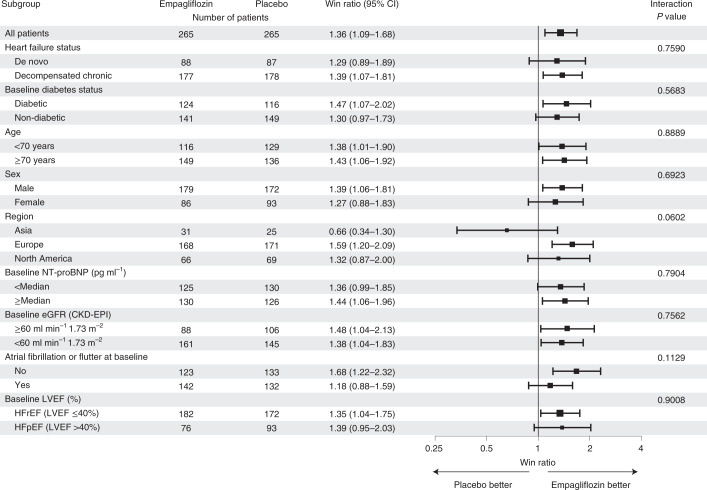
Primary efficacy outcome in all prespecified subgroups. Win ratios were calculated using a non-parametric generalized pairwise comparison within subgroup strata; data are presented as point estimates and 95% CIs with two-sided interaction *P* values. No adjustments for multiple testing were made. HFpEF, heart failure with preserved ejection fraction; HFrEF, heart failure with reduced ejection fraction.

A total of 33 patients (6.2%) died, 11 patients (4.2%) in the empagliflozin group and 22 (8.3%) in the placebo group. Sixty-seven patients (12.6%) had at least one HFE (empagliflozin, 28 patients, 10.6%; placebo, 39 patients, 14.7%). The adjusted mean change in Kansas City Cardiomyopathy Questionnaire Total Symptom Score (KCCQ-TSS) from baseline to 90 days was 36.2 (95% confidence interval (CI): 33.3–39.1) in the empagliflozin group and 31.7 (95% CI: 28.8–34.7) in the placebo group. Figure 2 shows the primary efficacy analysis of the hierarchical assessment of all-cause mortality, number and time to first HFEs, and change in KCCQ-TSS using the stratified win ratio. Empagliflozin was superior in 53.9% of paired comparisons and placebo was superior in 39.7%, whereas 6.4% of comparisons were tied, yielding a win ratio of 1.36 in favor of empagliflozin (95% CI: 1.09–1.68, *P* = 0.0054). Table 2 lists the proportion of wins and win ratios for all components of the primary outcome. The effect of empagliflozin on the primary efficacy outcome was generally consistent across prespecified subgroups, including acute heart failure status (de novo versus decompensated chronic heart failure), diabetes status, age, sex, geographic region, baseline N-terminal pro-brain natriuretic peptide (NT-proBNP), kidney function, atrial fibrillation status and LVEF subgroup (Fig. 3).

**Table 2:** Primary and secondary outcomes

	Empagliflozin (*n* = 265)	Placebo (*n* = 265)		*P* value
Primary endpoint			Win ratio (95% CI)^a^	
Primary endpoint (% wins)^b^	53.89	39.71	1.36 (1.09–1.68)	0.0054
Time to death (% wins)	7.15	4.01		
HFE frequency (% wins)^c^	10.59	7.65		
Time to first HFE (% wins)	0.24	0.57		
≥5 point difference in the KCCQ-TSS change from baseline to day 90 (% wins)^d^	35.91	27.48		
Percentage of ties	6.41	6.41		
Components described separately in the whole study population				
Deaths, *n* (%)	11 (4.2)	22 (8.3)		
Patients with HFE, *n* (%)	28 (10.6)	39 (14.7)		
Total HFEs, *n*	36	52		
Change from baseline in KCCQ-TSS at day 90^d^	See secondary endpoint			
Secondary endpoints			Hazard ratio (95% CI)	
CVD or HFE until end-of-trial visit, *n* (%), events per 100 patient years (95% CI)	34 (12.8), 55.01 (38.10–74.99)	49 (18.5), 80.45 (59.52–104.49)	0.69 (0.45–1.08)	
			Odds ratio (95% CI)	
KCCQ-TSS improvement ≥10 points at day 90, *n* (%)	220.1 (83.1)	202.1 (76.3)	1.522 (0.927–2.501)	
			Adjusted mean difference (95% CI)	
KCCQ-TSS change from baseline to day 90, adjusted mean (95% CI)	36.19 (33.28–39.09)	31.73 (28.80–34.67)	4.45 (0.32–8.59)	
Diuretic response (kg weight loss per mean daily loop diuretic dose)^e^, adjusted mean (95% CI)				
At day 15	−3.33 (−4.38 to −2.29)	−1.02 (−2.04 to 0.00)	−2.31 (−3.77 to −0.85)	
At day 30	−3.80 (−5.39 to −2.20)	−1.01 (−2.59 to 0.57)	−2.79 (−5.03 to −0.54)	
			Adjusted geometric mean ratio (95% CI)	
AUC of change from baseline in NT-proBNP at day 30, adjusted geometric mean (95% CI)^d^	24.07 (22.61–25.62)	26.77 (25.15–28.48)	0.90 (0.82–0.98)	
			Adjusted mean difference (95% CI)	
Percentage of days alive and out of hospital from study drug initiation until 30 days after initial hospital discharge, mean (s.d.)	81.37 (18.62)	80.90 (21.25)	0.47 (−2.97 to 3.91)	
Days alive and out of hospital from study drug initiation until 30 days after initial hospital discharge, mean (s.d.)	28.00 (6.15)	27.47 (6.63)	NA	
Percentage of days alive and out of hospital from study drug initiation until 90 days after randomization, mean (s.d.)	87.55 (19.54)	85.79 (22.76)	1.76 (−1.91 to 5.43)	
Days alive and out of hospital from study drug initiation until 90 days after randomization, mean (s.d.)	78.29 (20.17)	76.13 (22.85)	NA	
			Odds ratio (95% CI)	
Hospitalizations for heart failure until 30 days after initial hospital discharge, *n* (%)	14 (5.3)	12 (4.5)	1.179 (0.534–2.601)	
Occurrence of chronic dialysis or renal transplant or sustained reduction of ≥40% eGFR_CKD-EPIcr_, or sustained eGFR_CKD-EPIcr_ <15 ml min^−1^ 1.73 m^−^^2^ for patients with baseline eGFR ≥30 ml min^−1^ 1.73 m^−^^2^, sustained eGFR_CKD-EPIcr_ <10 ml min^−1^ 1.73 m^−^^2^ for patients with baseline eGFR <30 ml min^−1^ 1.73 m^−^^2^, *n* (%)	0	2 (0.8)	Not possible to fit a model	

AUC, area under the curve; CKD-EPIcr, Chronic Kidney Disease Epidemiology Collaboration equation using serum creatinine concentration; CVD, cardiovascular death; eGFR, estimated glomerular filtration rate; NA, not applicable.

The stratified win ratio for the primary endpoint was calculated using a non-parametric generalized pairwise comparison within heart failure status strata. For the secondary endpoints, the hazard ratio was calculated using a Cox proportional hazards model, the odds ratios were calculated using logistic regression models, the adjusted geometric mean ratio was calculated with analysis of covariance (ANCOVA), and the adjusted mean differences were calculated with either ANCOVA or mixed effects models for repeated measures, as appropriate. No adjustments for multiple testing were made. Data are given as point estimates and 95% CI, with two-sided *P* values, where appropriate. Full details are provided in Supplementary Note 2.

^a^Variance calculated using the asymptotic normal *U* statistics approach.

^b^Pairs are analyzed within strata for a stratified win ratio, applying weights that are analogous to a Mantel–Haenszel approach.

^c^Frequency based on events up to the earlier of the two censoring times.

^d^Based on multiple imputation with 100 iterations.

^e^Excluding patients not taking diuretics for more than 1 day during the time period; the units are kg per 40 mg i.v. furosemide (or 80 mg oral furosemide). The equivalent loop diuretic dose to a single dose of 40 mg furosemide is defined as 20 mg torasemide or 1 mg bumetanide.

### Secondary outcomes

Table 2 lists the prespecified secondary outcomes. The incidence of cardiovascular death or HFE until the end-of-trial visit was 12.8% in the empagliflozin group and 18.5% in the placebo group (hazard ratio, 0.69; 95% CI: 0.45–1.08). There was no significant difference in the proportion of patients with a KCCQ-TSS improvement of 10 points or greater at day 90 between the treatment groups. Patients in the empagliflozin group had a greater absolute change in KCCQ-TSS from baseline to day 90 (adjusted mean difference, 4.45 points; 95% CI: 0.32–8.59) than patients in the placebo group.

Patients in the empagliflozin group had a greater reduction in NT-proBNP concentration (as measured using the area under the curve of the change from baseline) at day 30 (adjusted geometric mean ratio, 0.90; 95% CI: 0.82–0.98) than patients in the placebo group. Other secondary endpoints are listed in Table 2.

### Safety analyses

A summary of adverse events is provided in Supplementary Table 2. Adverse events leading to discontinuation of empagliflozin or placebo occurred in 8.5% and 12.9% of patients, respectively. No ketoacidosis occurred in the empagliflozin or placebo groups. Rates of volume depletion were 12.7% in the empagliflozin group and 10.2% in the placebo group. Investigator-defined serious symptomatic hypotension occurred in 1.2% of the patients in the empagliflozin group and in 1.5% in the placebo group. Investigator-defined hypoglycemia occurred in 1.9% of patients treated with empagliflozin and in 1.5% of patients treated with placebo. Details of renal and urinary adverse events are given in Supplementary Table 3. Acute renal failure occurred in 7.7% of patients in the empagliflozin group and in 12.1% of patients in the placebo group. Urinary tract infection occurred in 4.2% of the patients in the empagliflozin group and in 6.4% in the placebo group. Adjusted mean changes in hematocrit, hemoglobin, alanine aminotransferase, aspartate aminotransferase, uric acid, and estimated glomerular filtration rate between baseline and day 90 are listed in Supplementary Table 4 and do not indicate any safety concerns for empagliflozin. The creatinine change between baseline and the last value on treatment was similar between the empagliflozin and placebo groups. There was a greater increase in hematocrit and hemoglobin in the empagliflozin group.

Regarding systolic blood pressure, the adjusted mean change from baseline to 90 days was 0.1 mmHg (95% CI: −2.5 to 2.7) in the empagliflozin group and 1.0 mmHg (95% CI: −1.6 to 3.6) in the placebo group. For diastolic blood pressure, the adjusted mean change from baseline to 90 days was −0.3 mmHg (95% CI: −1.8 to 1.3) in the empagliflozin group and −0.7 mmHg (95% CI: −2.3 to 0.8) in the placebo group.

## Discussion

Initiation of the SGLT2 inhibitor empagliflozin in patients hospitalized for acute heart failure resulted in a statistically significant and clinically meaningful benefit in the 90 days after randomization. Both a reduction in all-cause death and HFEs as well as an improvement in quality of life contributed to the increased number of wins in the empagliflozin group. We believe that the primary endpoint is meaningful because it allows the hierarchical assessment of benefit across three fundamental goals of care: improvement of survival, reduction of HFEs, and improvement of symptoms.

The effect was consistent across a broad spectrum of patients with reduced and preserved LVEF, acute de novo or decompensated chronic heart failure, and those with or without diabetes. Empagliflozin was well tolerated without safety concerns. The results of EMPULSE add to the accumulating evidence on the benefits of SGLT2 inhibitors in heart failure^9–14^.

EMPULSE is distinct from previous trials with SGLT2 inhibitors for several reasons. In particular, patients in EMPULSE were randomized early in the course of hospitalization for acute heart failure, at a median of 3 days after hospital admission. In the Effect of Sotagliflozin on Cardiovascular Events in Patients with Type 2 Diabetes Post Worsening Heart Failure (SOLOIST-WHF) trial, more than half of the patients were enrolled after hospital discharge and that trial included only patients with diabetes^12^. In addition, EMPULSE evaluated patients over the first 90 days after hospital admission, often considered the vulnerable phase of heart failure^15^. Also, EMPULSE included patients without a previous history of heart failure (that is, acute de novo), who were not yet treated for heart failure. The data support that adding empagliflozin to standard therapy was well tolerated and produced similar clinical benefit as in those with chronic decompensated heart failure. Thus, empagliflozin should be considered as an efficacious treatment in patients hospitalized for both de novo and decompensated acute heart failure.

The results of EMPULSE extend those of the pilot EMPA-RESPONSE-AHF trial (Randomized, Double Blind, Placebo Controlled, Multicenter Pilot Study on the Effects of Empagliflozin on Clinical Outcomes in Patients with Acute Decompensated Heart Failure), which suggested a clinical benefit of empagliflozin in patients hospitalized for acute heart failure^13^. Multiple large-scale drug trials in patients who were hospitalized for acute heart failure have failed to demonstrate compelling beneficial effects on their primary clinical outcome, although in several of these studies the therapy was given only for 24–48 hours and was not continued after hospital discharge^4–8^. PIONEER-HF (Comparison of Sacubitril/Valsartan Versus Enalapril on Effect on NT-proBNP in Patients Stabilized from an Acute Heart Failure Episode) had a similar design as EMPULSE, but primarily assessed the improvement in the concentration of NT-proBNP rather than clinical outcomes^16^. In addition, concerns have been raised about the safety of initiating chronic heart failure therapies early after a hospital admission for acute heart failure^17^. Because patients hospitalized for acute heart failure are often aggressively treated with diuretics and other vasoactive agents, it was previously unclear whether an SGLT2 inhibitor would increase the risk for worsening renal function, volume depletion and ketoacidosis. In the present study we show that empagliflozin was well tolerated when initiated in-hospital with fewer serious adverse events than placebo and with a clinical benefit that was readily apparent by 90 days. Reassuringly, no events of ketoacidosis were reported. Changes in blood pressure were minor and comparable between the groups. Empagliflozin significantly increased hemoglobin and hematocrit concentrations, which might be related to its diuretic effects. This is supported by a greater diuretic response both at day 15 and day 90 in the empagliflozin-treated patients.

The clinical benefit of empagliflozin was generally consistent across all prespecified subgroups, including patients with acute de novo and decompensated chronic heart failure, and those with or without type 2 diabetes. Notably, empagliflozin showed a clinical benefit in patients with both normal and reduced LVEF. The Empagliflozin Outcome Trial in Patients With Chronic Heart Failure with Reduced Ejection Fraction (EMPEROR-Reduced) and EMPEROR-Preserved recently showed that empagliflozin reduced the combined risk of cardiovascular death or hospitalization for heart failure in ambulant patients with chronic heart failure with reduced and preserved ejection fraction, respectively, regardless of diabetes status and ejection fraction, but these trials excluded hospitalized patients^9,11^. The results of EMPULSE therefore extend and complement those of EMPEROR-Reduced and EMPEROR-Preserved by focusing on patients hospitalized for acute heart failure across the range of ejection fraction.

This trial has some limitations. The short enrollment window and the requirement for patient stabilization might have excluded older, frailer and more severely diseased patients. The use of sacubitril–valsartan in EMPULSE, similar to earlier SGLT2 trials^9,10^, was modest but similar to contemporary background therapy in routine practice.

In conclusion, the results suggest that the initiation of empagliflozin as part of usual care in patients who are hospitalized for acute heart failure will result in a clinically meaningful benefit in 90 days without safety concerns.

## Methods

### Trial design and oversight

We conducted a multicenter, randomized, double-blind, 90 day superiority trial to evaluate the effect on clinical benefit, safety and tolerability of once daily oral EMPagliflozin 10 mg compared with placebo, initiated in patients hospitalized for acUte heart faiLure who have been StabilisEd (EMPULSE; ClinicalTrials.gov identifier NCT04157751). The Ethics Committee of each of the 118 sites in 15 countries approved the protocol and all patients gave written informed consent^18^. Boehringer Ingelheim and Eli Lilly sponsored the trial.

The executive committee of EMPULSE (Supplementary Note 1), consisting of academic members and representatives of Boehringer Ingelheim, designed the protocol and provided oversight of the trial’s conduct together with the trial sponsor. The sponsor performed statistical analyses of the trial according to a prespecified statistical analysis plan with oversight by the executive committee (Supplementary Note 1). Jonathan Blatchford is the statistician who coordinated all analyses. An independent data and safety monitoring committee reviewed the safety data.

### Patients

The trial design has been reported previously^18^. Participants were men or women aged at least 18 years (at least 21 years in Japan, being the age of legal consent) who were hospitalized with a primary diagnosis of acute heart failure with dyspnea on exertion or at rest, and at least two of the following: congestion on chest radiograph, rales on chest auscultation, clinically relevant edema (for example, at least 1+ on a 0–3+ scale), or an elevated jugular venous pressure. Patients were randomized after at least 24 h and no later than 5 days after admission, as early as possible after stabilization and while still in hospital. Patients were required to have a systolic blood pressure of at least 100 mmHg, no inotropic support for at least 24 h, no symptoms of hypotension, and in the 6 h prior to randomization no increase in the i.v. diuretic dose and no i.v. vasodilators including nitrates. Patients were required to have an NT-proBNP concentration of at least 1,600 pg ml^−1^ or a B-type natriuretic peptide (BNP) concentration of at least 400 pg ml^−1^. Patients in atrial fibrillation were required to have an NT-proBNP concentration of at least 2,400 pg ml^−1^ or a BNP concentration of at least 600 pg ml^−1^. Patients had to be treated with a minimum dose of 40 mg (20 mg for Japanese patients) i.v. furosemide or equivalent. Key exclusion criteria included cardiogenic shock; pulmonary embolism, cerebrovascular accident or acute myocardial infarction as the primary trigger for the current hospitalization or in the preceding 90 days before randomization; current or expected cardiac transplantation, left ventricular assist device, or inotropic support; an estimated glomerular filtration rate (eGFR) less than 20 ml min^−1^ per 1.73 m^2^ or requiring dialysis; and prior ketoacidosis. Key inclusion and exclusion criteria are listed in Supplementary Note 1^18^

### Trial visits and follow-up

Efficacy and safety parameters were assessed during follow-up visits at 3, 5, 15, 30 and 90 days after randomization. During the onsite visits at 15, 30 and 90 days, eGFR, natriuretic peptides, New York Heart Association class, and health status using the Kansas City Cardiomyopathy Questionnaire were assessed. Because of the ongoing coronavirus disease 2019 (COVID-19) pandemic, several adjustments to the study protocol were made, outlined in Supplementary Note 1. In brief, if patients were unable to come to the study site due to COVID-19 restrictions or safety concerns, a phone or home visit was allowed instead of an in-person site visit; if the collection of blood samples for the central laboratory was not possible, a local laboratory could be used and trial medication could be shipped if participants were unable to collect it. Overall, only two patients missed a visit due to COVID-19 disruption. In addition, 23 patients had a remote visit due to COVID-19 disruption. Compliance was assessed by tablet count at visits 3, 4 and 5.

### Primary and secondary outcomes

The primary outcome was clinical benefit at 90 days, defined as a hierarchical composite outcome of time to all-cause death, the number of HFEs, time to first HFE, and a 5 point or greater difference in change from baseline in KCCQ-TSS after 90 days of treatment. HFEs included heart failure hospitalizations, urgent heart failure visits and unplanned outpatient heart failure visits. An event was considered a HFE only if worsening signs and symptoms of heart failure were present and an intensification of therapy (defined as an increase of oral or i.v. diuretics, augmentation of a vasoactive agent, or starting a mechanical or surgical intervention) was performed. The complete definition is provided in the study protocol (Supplementary Note 2). Secondary outcomes included time to first occurrence of cardiovascular death or hospitalization for heart failure, change in KCCQ-TSS, diuretic response after 15 and 30 days of treatment, change in NT-proBNP concentration over 30 days of treatment, days alive and out of hospital, occurrence of a heart failure hospitalization until 30 days after initial hospital discharge and occurrence of chronic dialysis or renal transplant or significant and sustained reduction of eGFR (definitions are provided in the study protocol; see Supplementary Notes 1 and 2). Safety parameters included markers of volume depletion, hypotension and acute renal failure (Supplementary Notes 1 and 2).

### Statistical analysis

A sample size of 500 participants (250 per treatment arm) was estimated to provide a power of 87% at a one-sided alpha level of 0.025 under a set of assumptions previously published^18^ and listed in the study protocol (Supplementary Note 2). The primary analyses were performed according to the intention-to-treat principle and included all available data after randomization. The primary outcome analysis was performed using a stratified win ratio, which compares all patients randomized to empagliflozin with all patients randomized to placebo, in their heart failure status (acute de novo or decompensated chronic heart failure). Each comparison of two patients followed the hierarchy of comparing time to death, number of HFEs, time to HFE or a 5 point or greater difference in change from baseline in the KCCQ-TSS at day 90 until conclusion of a win or loss or otherwise concluding by a tie. We calculated the stratified win ratio^19^ as the number of wins in the empagliflozin group divided by the number of losses, which was then combined across both strata. A multiple imputation approach, according to whether patients were on treatment or off treatment, was used to impute missing data for the KCCQ-TSS. For more details on the win ratio and the imputation methods see Supplementary Note 1. For the secondary outcomes, a Cox proportional hazards model was used to analyze time to cardiovascular death or HFE. Comparison between treatment groups regarding improvement in KCCQ-TSS of 10 points or greater after 90 days of treatment was performed using a logistic regression model adjusting for heart failure status and baseline score. KCCQ-TSS at day 90 was evaluated using a mixed effects model for repeated measures adjusting for heart failure status and baseline score by visit interaction. The area under the curve of change from baseline in log-transformed NT-proBNP concentration, calculated using the linear trapezoidal rule, was assessed using an analysis of covariance model adjusting for heart failure status and the log of baseline NT-proBNP. Other secondary outcomes were analyzed using similar methods, as appropriate. The incidence of adverse events are shown descriptively. *P* values or confidence intervals are not adjusted for multiple comparisons.

All analyses were performed with SAS version 9.3 or higher (SAS Institute).

The Ethics Committee of each of the 118 sites in 15 countries approved the protocol and all patients gave written informed consent.

### Reporting Summary

Further information on research design is available in the Nature Research Reporting Summary linked to this article.

## Online content

Any methods, additional references, Nature Research reporting summaries, source data, extended data, supplementary information, acknowledgements, peer review information; details of author contributions and competing interests; and statements of data and code availability are available at 10.1038/s41591-021-01659-1.

## Supplementary information

Supplementary Information Supplementary Tables 1–4.

Reporting Summary

Supplementary Note 1

Supplementary Note 2

## Data Availability

To ensure independent interpretation of clinical study results, Boehringer Ingelheim grants all external authors access to relevant material, including participant-level clinical study data, as needed by them to fulfill their role and obligations as authors under the International Committee of Medical Journal Editors criteria. Clinical study documents and participant clinical study data are available to be shared on request after publication of the primary manuscript in a peer-reviewed journal, and if regulatory activities are complete and other criteria met as per the BI Policy on Transparency and Publication of Clinical Study Data (see https://www.mystudywindow.com). Bona fide, qualified scientific and medical researchers are eligible to request access to the clinical study data with corresponding documentation describing the structure and content of the datasets. Upon approval, and governed by a legal agreement, data are shared in a secure data-access system for a limited period of 1 year, which may be extended upon request. Prior to providing access, clinical study documents and data will be examined, and, if necessary, redacted and de-identified to protect the personal data of the study participants and personnel, and to respect the boundaries of the informed consent of the study participants. Researchers should use the https://vivli.org/ link to request access to study data and visit https://www.mystudywindow.com for further information.
