# Supplementary material for: The SGLT2 inhibitor empagliflozin in patients hospitalized for acute heart failure: a multinational randomized trial
Source: Nat Med. 2022 Feb 28;28(3):568–74. doi: 10.1038/s41591-021-01659-1 (PMC8938265; doi:10.1038/s41591-021-01659-1)
Supplement: Supplementary file 1 — Supplementary Tables 1–4. [file 41591_2021_1659_MOESM1_ESM.pdf]

nature portfolio

https://doi.org/10.1038/s41591-021-01659-1

Supplementary information

The SGLT2 inhibitor empagliflozin in patients hospitalized for acute heart failure: a multinational randomized trial

In the format provided by the authors and unedited

SUPPLEMENTARY INFORMATION FOR THE EMPULSE TRIAL

CONTENTS

SUPPLEMENTARY TABLE 1. REASONS FOR SCREENING FAILURE 2

SUPPLEMENTARY TABLE 2. SUMMARY OF ADVERSE EVENTS 3

SUPPLEMENTARY TABLE 3. DETAILS ON RENAL AND URINARY ADVERSE EVENTS................. 4

SUPPLEMENTARY TABLE 4. CHANGES IN HEMATOCRIT, HEMOGLOBIN, ALANINE AMINOTRANSFERASE (ALT), ASPARTATE TRANSAMINASE (AST), URIC ACID AND ESTIMATED GLOMERULAR FILTRATION RATE.. 5

SUPPLEMENTARY TABLE 1. REASONS FOR SCREENING FAILURE

Reason	Total N (%)
Not randomized	36 (100%)
Adverse event	1 (2.8%)
Inclusion / exclusion criteria not met*	30 (83.3%)
Conditions on NT−proBNP not met	7 (19.4%)
Patient not hemodynamically stabilized	5 (13.9%)
Primary diagnosis is acute HF not triggered by volume overload	2 (5.6%)
Missing	16 (44.4%)
Lost to follow-up	0
Consent withdrawn (not due to an adverse event)	1 (2.8%)
Other	4 (11.1%)

* Patients may have not met more than one in/exclusion criterion.

SUPPLEMENTARY TABLE 2. SUMMARY OF ADVERSE EVENTS

Category of AEs	Empagliflozin 10 mg N (%)	Placebo N (%)
Number of patients	260 (100.0)	264 (100.0)
Patients with any AEs	182 (70.0)	204 (77.3)
Severe AEs	39 (15.0)	54 (20.5)
Investigator defined drug-related AEs	30 (11.5)	27 (10.2)
AEs leading to discontinuation of study medication	22 (8.5)	34 (12.9)
Serious AEs	84 (32.3)	115 (43.6)
Results in death	9 (3.5)	17 (6.4)
Is life threatening	12 (4.6)	14 (5.3)
Persistent or significant disability/incapacity	0	1 (0.4)
Requires or prolongs hospitalization	64 (24.6)	87 (33.0)
Congenital anomaly or birth defect	0	0
Other medically important serious event	33 (12.7)	48 (18.2)
AEs of special interest		
Hepatic injury (narrow SMQ)	10 (3.8)	13 (4.9)
Acute renal failure (narrow SMQ)	20 (7.7)	32 (12.1)
Ketoacidosis (narrow SMQ)	0	0

Percentages calculated using total number of patients per treatment as the denominator.

A patient may be counted in more than one seriousness criterion.

SUPPLEMENTARY TABLE 3. DETAILS ON RENAL AND URINARY ADVERSE EVENTS

MedDRA PT	Empagliflozin 10 mg		Placebo	
	N (%)	Rate/100 pt-yrs	N (%)	Rate/100 pt-yrs
Number of patients	260 (100.0)		264 (100.0)	
Acute renal failure (narrow SMQ)[1]	20 (7.7)	34.69	32 (12.1)	55.69
Acute kidney injury	10 (3.8)	16.90	19 (7.2)	32.28
Renal impairment	9 (3.5)	15.38	11 (4.2)	18.58
Renal failure	2 (0.8)	3.34	2 (0.8)	3.33
Serious	12 (4.6)	20.37	23 (8.7)	39.35
Leading to discontinuation of study medication	6 (2.3)	10.02	4 (1.5)	6.64
Urinary tract infection	11 (4.2)	18.80	17 (6.4)	29.23
Cystitis	8 (3.1)	13.62	5 (1.9)	8.36
Urinary tract infection	3 (1.2)	5.01	6 (2.3)	10.10
Urinary tract infection bacterial	1 (0.4)	1.67	6 (2.3)	10.07
Escherichia urinary tract infection	1 (0.4)	1.67	0	-
Bacteriuria	0	-	1 (0.4)	1.66
Complicated[2]	1 (0.4)	1.67	2 (0.8)	3.33

[1] Using standard MedDRA query v24.0 definition for Acute renal Failure consist of 19 preferred terms including acute kidney injury, subacute kidney injury, anuria, oliguria, azotemia, renal failure, renal impairment, haemodialysis, haemofiltration, peritoneal dialysis

[2] Complicated UTI: Renal infections or Urosepsis or serious urinary tract infections.

SUPPLEMENTARY TABLE 4. CHANGES IN HEMATOCRIT, HEMOGLOBIN, ALANINE AMINOTRANSFERASE (ALT), ASPARTATE TRANSAMINASE (AST), URIC ACID AND ESTIMATED GLOMERULAR FILTRATION RATE

	Empagliflozin 10 mg	Placebo
Number of patients	260	264
Hematocrit [%]		
Number of analyzed patients	212	217
Baseline mean (SE)	40.32 (0.42)	40.87 (0.43)
Change from baseline at Day 90 adjusted mean (95% CI)	0.07 (-0.51, 0.64)	-1.87 (-2.46, -1.28)
Comparison vs placebo at Day 90 adjusted mean (95% CI)	1.94 (1.11, 2.76)	
Hemoglobin [g/dL]		
Number of analyzed patients	214	223
Baseline mean (SE)	13.21 (0.14)	13.40 (0.14)
Change from baseline at Day 90 adjusted mean (95% CI)	0.23 (0.04, 0.41)	-0.41 (-0.60, -0.22)
Comparison vs placebo at Day 90 adjusted mean (95% CI)	0.64 (0.37, 0.90)	
ALT [U/L]		
Number of analyzed patients	213	216
Baseline mean (SE)	28.8 (2.1)	28.4 (2.2)
Change from baseline at Day 90 adjusted mean (95% CI)	-7.5 (-13.1, -1.9)	-2.9 (-8.7, 2.9)
Comparison vs placebo at Day 90 adjusted mean (95% CI)	-4.7 (-12.7, 3.4)	
AST [U/L]		
Number of analyzed patients	210	211
Baseline mean (SE)	28.2 (1.8)	25.5 (1.1)
Change from baseline at Day 90 adjusted mean (95% CI)	-3.8 (-9.5, 2.0)	2.1 (-3.9, 8.2)
Comparison vs placebo at Day 90 adjusted mean (95% CI)	-5.9 (-14.3, 2.4)	
Uric acid [mg/dL]		
Number of analyzed patients	222	225
Baseline mean (SE)	8.62 (0.17)	8.58 (0.15)
Change from baseline at Day 90 adjusted mean (95% CI)	-1.96 (-2.24, -1.69)	-0.82 (-1.10, -0.54)
Comparison vs placebo at Day 90 adjusted mean (95% CI)	-1.15 (-1.54, -0.75)	
eGFR [mL/min/1.73m^2]		
Number of analyzed patients	220	225
Baseline mean (SE)	52.7 (1.3)	56.4 (1.4)
Change from baseline at Day 90 adjusted mean (95% CI)	1.9 (-0.2, 4.1)	1.1 (-1.2, 3.3)
Comparison vs placebo at Day 90 adjusted mean (95% CI)	0.9 (-2.2, 4.0)	
