# Supplementary material for: The SGLT2 inhibitor empagliflozin in patients hospitalized for acute heart failure: a multinational randomized trial
Source: Nat Med. 2022 Feb 28;28(3):568–74. doi: 10.1038/s41591-021-01659-1 (PMC8938265; doi:10.1038/s41591-021-01659-1)
Supplement: Supplementary file 2 — Reporting Summary [file 41591_2021_1659_MOESM2_ESM.pdf]

nature portfolio

Corresponding author(s): Prof. Dr. A.A. Voors

Last updated by author(s): Dec 3, 2021

Reporting Summary

Nature Portfolio wishes to improve the reproducibility of the work that we publish. This form provides structure for consistency and transparency in reporting. For further information on Nature Portfolio policies, see our Editorial Policies and the Editorial Policy Checklist.

Statistics

For all statistical analyses, confirm that the following items are present in the figure legend, table legend, main text, or Methods section.

n/a	Confirmed	
☐	☒	The exact sample size (*n*) for each experimental group/condition, given as a discrete number and unit of measurement
☒	☐	A statement on whether measurements were taken from distinct samples or whether the same sample was measured repeatedly
☐	☒	The statistical test(s) used AND whether they are one- or two-sided<br>*Only common tests should be described solely by name; describe more complex techniques in the Methods section.*
☐	☒	A description of all covariates tested
☐	☒	A description of any assumptions or corrections, such as tests of normality and adjustment for multiple comparisons
☐	☒	A full description of the statistical parameters including central tendency (e.g. means) or other basic estimates (e.g. regression coefficient) AND variation (e.g. standard deviation) or associated estimates of uncertainty (e.g. confidence intervals)
☐	☒	For null hypothesis testing, the test statistic (e.g. *F*, *t*, *r*) with confidence intervals, effect sizes, degrees of freedom and *P* value noted<br>*Give P values as exact values whenever suitable.*
☒	☐	For Bayesian analysis, information on the choice of priors and Markov chain Monte Carlo settings
☐	☒	For hierarchical and complex designs, identification of the appropriate level for tests and full reporting of outcomes
☒	☐	Estimates of effect sizes (e.g. Cohen's *d*, Pearson's *r*), indicating how they were calculated

Our web collection on statistics for biologists contains articles on many of the points above.

Software and code

Policy information about availability of computer code

Data collection	All clinical data were captured using the electronic data capture system RAVE
Data analysis	SAS software, version 9·3 or higher (SAS Institute).

For manuscripts utilizing custom algorithms or software that are central to the research but not yet described in published literature, software must be made available to editors and reviewers. We strongly encourage code deposition in a community repository (e.g. GitHub). See the Nature Portfolio guidelines for submitting code & software for further information.

Data

Policy information about availability of data

All manuscripts must include a data availability statement. This statement should provide the following information, where applicable:

- Accession codes, unique identifiers, or web links for publicly available datasets
- A description of any restrictions on data availability
- For clinical datasets or third party data, please ensure that the statement adheres to our policy

To ensure independent interpretation of clinical study results, Boehringer Ingelheim grants all external authors access to relevant material, including participant-level clinical study data, as needed by them to fulfill their role and obligations as authors under the ICMJE criteria.
Clinical study documents and participant clinical study data are available to be shared on request after publication of the primary manuscript in a peer-reviewed journal, and if regulatory activities are complete and other criteria met as per the BI Policy on Transparency and Publication of Clinical Study Data (see Medical & Clinical Trials | Clinical Research | MyStudyWindow). Bona fide, qualified scientific and medical researchers are eligible to request access to the clinical study data with corresponding documentation describing the structure and content of the datasets. Upon approval, and governed by a Legal Agreement, data are shared in a secured data-access system for a limited period of 1 year, which may be extended upon request. Prior to providing access, clinical study documents and data will be

examined, and, if necessary, redacted and de-identified, to protect the personal data of study participants and personnel, and to respect the boundaries of the informed consent of the study participants.
Researchers should use the https://vivli.org/ link to request access to study data and visit Medical & Clinical Trials | Clinical Research | MyStudyWindow for further information
On average it takes a few months to access data in the Vivli platform, but the timeline will vary depending on the number of data contributors, the number of studies, and your availability to respond to comments.

Field-specific reporting

Please select the one below that is the best fit for your research. If you are not sure, read the appropriate sections before making your selection.

☒ Life sciences ☐ Behavioural & social sciences ☐ Ecological, evolutionary & environmental sciences

For a reference copy of the document with all sections, see nature.com/documents/nr-reporting-summary-flat.pdf

Life sciences study design

All studies must disclose on these points even when the disclosure is negative.

Sample size	A sample size of 500 participants (250 per treatment arm) was estimated to provide a power of 87% at a one-sided alpha level of 0·025 under a set of assumptions previously published and listed in the study protocol.
Data exclusions	The primary analyses were performed according to the intention-to-treat principle and included all available data after randomisation. A total of 530 patients were included in the efficacy analyses using the intention-to-treat principle. Five hundred twenty-four patients received at least one dose of the trial drug (260 in the empagliflozin and 264 in the placebo group, Figure 1). These patients were subsequently included in the safety analyses. Early discontinuation of the trial drug occurred in 114 (21·8%) patients: 52 (20·0%) in the empagliflozin group and 62 patients (23·5%) in the placebo group. Eleven (2·1%) patients were lost to follow-up from the trial.
Replication	Replication is not relevant as this is a single study in which key events (e.g. death) happened only once.
Randomization	Each eligible patient was randomised to receive empagliflozin 10 mg, or matching placebo according to a randomisation plan in a 1:1 ratio, stratified according to heart failure status (de novo or decompensated chronic) at Visit 2a via Interactive Response Technology. Patient assignment to a treatment group was determined by a computer generated random sequence.
Blinding	Patients, investigators, central reviewers, and everyone involved in trial conduct or analysis or with any other interest in this double-blind trial were blinded with regard to the randomised treatment assignments until after database lock.

Reporting for specific materials, systems and methods

We require information from authors about some types of materials, experimental systems and methods used in many studies. Here, indicate whether each material, system or method listed is relevant to your study. If you are not sure if a list item applies to your research, read the appropriate section before selecting a response.

Materials & experimental systems

n/a	Involved in the study	
☒	☐	Antibodies
☒	☐	Eukaryotic cell lines
☒	☐	Palaeontology and archaeology
☒	☐	Animals and other organisms
☐	☒	Human research participants
☐	☒	Clinical data
☒	☐	Dual use research of concern

Methods

n/a	Involved in the study	
☒	☐	ChIP-seq
☒	☐	Flow cytometry
☒	☐	MRI-based neuroimaging

Human research participants

Policy information about studies involving human research participants

Population characteristics	Participants were men or women aged at least 18 years (at least 21years in Japan, being the age of legal consent) hospitalised with a primary diagnosis of acute heart failure with dyspnoea on exertion or at rest, and at least two of the following: congestion on chest radiograph, rales on chest auscultation, clinically relevant oedema (e.g. at least 1+ on a 0 to 3+ scale), or an elevated jugular venous pressure. Patients were randomised after at least 24 hours and no later than 5 days after admission, as early as possible after stabilisation and while still in hospital. Patients were required have a systolic blood pressure of at least 100mmHg, no inotropic support for at least 24hours, no symptoms of hypotension, and in the six hours prior to randomisation no increase in the intravenous diuretic dose and no intravenous vasodilators including nitrates. Patients were required to have a N-terminal pro-B-type natriuretic peptide (NT-proBNP) concentration of at least 1600pg/mL or a B-type natriuretic peptide (BNP) concentration of at least 400pg/mL. Patients in atrial fibrillation were required to have a

	NT-proBNP concentration of at least 2400pg/mL or a BNP concentration of at least 600pg/mL. Patients had to be treated with a minimum dose of 40 mg (20mg for Japanese patients) of intravenous furosemide or equivalent. The median age was 71 (interquartile range 61 to 78) years, 34% were women, and 78% were white. The median time from hospital admission to randomisation was 3 days .
Recruitment	Participants were recruited by investigators at the trial centers based on study eligibility criteria. Any self-selection bias or other bias would be a function of the characteristics of patients presenting during the recruitment period and the judgement of the investigators, but would be very unlikely to be imbalanced between treatment arms, given the multicentre, double-blind, randomized trial design.
Ethics oversight	All participating centres' ethics committees or relevant regulatory authorities approved the trial protocol

Note that full information on the approval of the study protocol must also be provided in the manuscript.

Clinical data

Policy information about clinical studies

All manuscripts should comply with the ICMJE guidelines for publication of clinical research and a completed CONSORT checklist must be included with all submissions.

Clinical trial registration: ClinicalTrials.gov identifier NCT04157751

Study protocol: Study protocol available from the sponsor

Data collection: May 18, 2020 to June 2, 2021 at 118 hospitals in 15 countries

Outcomes:

The primary outcome was clinical benefit at 90 days, defined as a hierarchical composite outcome of time to all-cause death, the number of heart failure events, time to first heart failure event, and a5-point or greater difference in change from baseline in Kansas City Cardiomyopathy Questionnaire Total Symptom Score after 90days of treatment. Heart failure events included heart failure hospitalisations, urgent heart failure visits and unplanned outpatient heart failure visits. An event was only considered a heart failure event if worsening signs and symptoms of heart failure were present and an intensification of therapy defined as an increase of oral or intravenous diuretics, augmentation of a vasoactive agent, or starting a mechanical or surgical intervention was performed. The complete definition is provided in the study protocol (Supplementary Appendix). Secondary outcomes included time to first occurrence of cardiovascular death or hospitalisation for heart failure, change in Kansas City Cardiomyopathy Questionnaire Total Symptom Score, diuretic response after 15 and 30 days of treatment, change in NT-proBNP concentration over 30 days of treatment, days alive and out of hospital, occurrence of a heart failure hospitalisation until 30 days after initial hospital discharge and occurrence of chronic dialysis or renal transplant or significant and sustained reduction of estimated glomerular filtration rate (definitions are provided in the study protocol; see Supplementary Appendix). Safety parameters included markers of volume depletion, hypotension and acute renal failure (Supplementary Appendix).

Assessments were conducted according to the protocol, as follows:

7.2.2 Primary endpoint analyses

The primary endpoint is net clinical benefit, a composite of death, number of HFEs, time to first HFE and change from baseline in KCCQ-CSS after 90 days of treatment. All patients randomised to empagliflozin are compared to all patients randomised to placebo.

For any two patients, the patient with the greater net clinical benefit is determined by assessing the following criteria sequentially, stopping when an advantage for either patient is shown:

1. Death within common follow-up time
- death is worse than no death
- earlier death is worse
- tied, if not possible to determine

2. Number of HFEs within common follow-up time
- more HFEs is worse
- tied, if same number of HFEs

3. Time to first HFE within common follow-up time
- earlier HFE is worse
- tied, if not possible to determine

4. KCCQ-CSS change from baseline at Day 90
- more positive change from baseline is better

Note, priority is therefore given to death over HFE, and both of these over changes in KCCQ- CSS. Below are some examples:

5. Death, e.g.:
- Patient A dies 30 days after randomisation (loses)
- Patient B dies 40 days after randomisation (wins)

6. If no winner based on death, number of HFEs within common follow-up time, e.g.:
- Patient A had two HFEs (loses)
- Patient B had one HFE (wins)

7. If no winner based on number of HFEs, time to first HFE, e.g.:

- Patient A had an HFE 30 days after randomisation (loses)
- Patient B had an HFE 50 days after randomisation (wins)

8. If no winner based on time to first HFE, KCCQ-CSS change from baseline at Day 90, e.g.:

- Patient A: KCCQ-CSS change from baseline at Day 90 is x (loses)
- Patient B: KCCQ-CSS change from baseline at Day 90 is y (y>x) (wins)

The implemented generalised pairwise comparisons approach compares all patients in one treatment group to all other patients within their strata in the other treatment group. The net benefit (Δ) is then calculated as the total number of wins in the empagliflozin group across all strata minus the total number of losses ad then divided by the total number of comparisons. This approach is analogous to Van Elteren's test [R98-1473].

Separate summaries for each component of this endpoint will also be presented. Sensitivity and subgroup analyses for the primary endpoint will be specified in the TSAP. The method of handling missing KCCQ-CSS values for this analysis is described in Section 7.3.

7.2.3 Secondary endpoint analyses

Secondary endpoints will not be tested in a hierarchical sequence, and no adjustment for multiple comparisons is planned.

Change from baseline in continuous endpoints, such as KCCQ-CSS, will be analysed usingrestricted maximum likelihood estimation based on a mixed-effect model for repeated measures (MMRM) analysis to obtain adjusted means for the treatment effects. This model will include discrete fixed effects for treatment group (empagliflozin or placebo) and HF status (de novo or decompensated chronic HF) at each visit and continuous fixed effects for baseline value at each visit. Missing data caused by patient withdrawal or other reasons will be handled implicitly by the MMRM approach. Area under the curve (AUC) of change from baseline in log-transformed NT-proBNP level over 30 days of treatment will be analysed by an analysis of covariance (ANCOVA). Based on literature reviews, NT-proBNP level is regarded as log-normally distributed, therefore values will be log-transformed prior to analysis [R19-3044]. The linear trapezoidal rule will be used to calculate the AUC after the log-transformation has been applied to each value.

Analysis of covariance (ANCOVA) with a discrete fixed effect for HF status (de novo or decompensated chronic HF) and a continuous fixed effect for baseline NT-proBNP level (log-transformed) will be used to compare treatment groups. The method of handling missing NT-proBNP levels for this analysis is described in Section 7.3.

Comparisons between treatment groups regarding the binary endpoint variable (improvement in KCCQ-CSS of ≥ 10 points after 90 days of treatment) will be performed using a logistic regression model adjusting for the binary covariate HF status (de novo or decompensated chronic HF). The likelihood-ratio test will be used to test for a difference between treatments. Adjusted odds ratios together with 1-sided 97.5% confidence limits will be used to quantify the effect of treatment, comparing empagliflozin to placebo as the reference. Time to event endpoints will be analysed using the Cox proportional hazards model [R07-4680] with HF status (de novo or decompensated chronic HF) as a covariate. Hazard ratios (HRs) and their associated one-sided 97.5% confidence limits will be estimated for evaluating the superiority of empagliflozin to placebo.

Other secondary endpoints will be summarised descriptively (including days alive and out of hospital).

Analysis of recurrent events will be described in the TSAP.
