# Supplementary material for: The SGLT2 inhibitor empagliflozin in patients hospitalized for acute heart failure: a multinational randomized trial
Source: Nat Med. 2022 Feb 28;28(3):568–74. doi: 10.1038/s41591-021-01659-1 (PMC8938265; doi:10.1038/s41591-021-01659-1)
Supplement: Supplementary file 3 — Supplementary Note 1 [file 41591_2021_1659_MOESM3_ESM.pdf]

SUPPLEMENTARY NOTE 1

CONTENTS

EXECUTIVE COMMITTEE OF EMPULSE TRIAL 2
DATA MONITORING COMMITTEE 2
NATIONAL LEAD COORDINATORS 3
KEY INCLUSION AND EXCLUSION CRITERIA 11
KEY INCLUSION CRITERIA 11
KEY EXCLUSION CRITERIA 11
MAJOR PROTOCOL-SPECIFIED EFFICACY ENDPOINTS 13
METHODOLOGY USED FOR THE PRIMARY ENDPOINT (WIN RATIO) 14

EXECUTIVE COMMITTEE OF EMPULSE TRIAL

Adriaan A Voors MD, (Chair) Professor of Cardiology, Department of Cardiology University of Groningen, University Medical Center Groningen, Groningen, the Netherlands
Piotr Ponikowski MD, (Co-Chair) Department of Heart Diseases, Medical University, Wroclaw, Poland
Christiane E Angermann MD, (Coordinating Investigator) Comprehensive Heart Failure Centre, University & University Hospital Würzburg, Würzburg, Germany
John R Teerlink MD, (Coordinating Investigator) Section of Cardiology, San Francisco Veterans Affairs Medical Center and School of Medicine, University of California San Francisco, San Francisco, USA
Mikhail Kosiborod MD, Saint Luke's Mid America Heart Institute, University of Missouri, Kansas City, USA and The George Institute for Global Health and the University of New South Wales, Sydney, NSW, Australia.
Sean P Collins MD, Department of Emergency Medicine, Vanderbilt University Medical Center, Nashville, USA
Jan Biegus MD, Department of Heart Diseases, Medical University, Wroclaw, Poland
Jasper Tromp MD, Duke-NUS Medical School and National Heart Centre Singapore, Singapore
João Pedro Ferreira MD, Université de Lorraine, Inserm, Centre d'Investigation Clinique Plurithématique, Nancy, France
Mitchell A Psotka MD, Inova Heart and Vascular Institute, Falls Church, USA
Michael E Nassif MD, Saint Luke's Mid America Heart Institute, University of Missouri, Kansas City, USA
Martina Brueckmann MD, PhD, Boehringer Ingelheim International, Ingelheim, Germany
Afshin Salsali MD, Cardiometabolic Medicine Boehringer Ingelheim Pharmaceuticals, Ridgefield, CT, USA

DATA MONITORING COMMITTEE
Francine K. Welty MD, PhD (DMC Chair), Harvard Medical School, Boston, MA, USA
Tim Clayton MSc (Member Statistician), London School of Hygiene and Tropical Medicine, London, UK
Klaus G. Parhofer MD (DMC Member), University of Munich, Munich, Germany
Barry Greenberg MD (DMC Member), UC San Diego Medical Center, La Jolla, CA, USA
Marvin A. Konstam MD (DMC Member), Tufts University School of Medicine, Tufts Medical Center, Boston, MA, USA
Kennedy R. Lees MD (DMC Member), Emeritus Professor, Stroke Opinions Limited, Troon, UK
Mike Palmer BSc. FSS PhD (Independent Statistician), N Zero 1 Ltd, Wilmslow, Cheshire, UK

NATIONAL LEAD COORDINATORS

Belgium: Stefan P Janssens MD, Department of Cardiovascular Sciences, Clinical Cardiology, Belgium University Hospital, KU Leuven

Canada: Shelley Zieroth MD, Section of Cardiology, Max Rady College of Medicine, University of Manitoba, Winnipeg

China: Changsheng Ma MD, Beijing Anzhen Hospital Capital Medical University Cardiology, Beijing

Czech Republic: Lenka Spinarova MD, 1st Department of Medicine, Masaryk University Hospital, Brno

Denmark: Morten Schou MD, Ph.D; Department of Cardiology, Gentofte University Hospital Copenhagen

Germany: P. Christian Schulze MD, University Hospital Jena, Jena

Germany: Uwe Zeymer MD, Klinikum Ludwigshafen, Ludwigshafen

Hungary: Robert G Kiss MD, Department of Cardiology, Military Hospital, Budapest

Italy: Maurizio Volterrani MD, Department of Cardiology, IRCCS San Raffaele Pisana, Rome

Japan: Yasushi Sakata MD, Department of Cardiovascular Medicine, Osaka University Graduate School of Medicine, Osaka

Netherlands: C. Jan Willem Borleffs MD, Haga Teaching Hospital, Den Hague

Norway: Henrik Schirmer MD, Department of Cardiology, Division of Medicine, Akershus University Hospital, Lørenskog

Poland: Jerzy K. Wranicz MD, Department of Electrocardiology, Medical University Central Clinical Hospital, Lodz

Spain: Josep Comin-Colet MD, Hospital Universitario de Bellvitge, Barcelona

Sweden: Michael Fu, MD, PhD, Section of Cardiology, Sahlgrenska University Hospital, University Gothenburg, Gothenburg

USA: Robert John Mentz MD, Duke Clinical Research Institute and Division of Cardiology, Duke University Medical Center, Durham

UAS: Sean Collins MD, Department of Emergency Medicine, Vanderbilt University Medical Center Vanderbilt University, Nashville

SPONSORS OF THE TRIAL

The sponsors of the trial were Boehringer Ingelheim and Eli Lilly and Company. Boehringer Ingelheim had the organizational oversight over the EMPULSE trial, which included trial conduct, supervision and monitoring of the enrolling study centers, data collection and storage as well as data storage and data analysis. The trial design was developed by the academic members of the executive Committee in co-

operation with representatives from Boehringer Ingelheim, who were also represented in the executive committee of the trial.

The executive committee developed and amended the protocol, case report forms, and statistical analysis plan; oversaw the recruitment of patients and the quality of follow-up; supervised the analysis of data; and the academic members provided an independent interpretation of the results. Jonathan Blatchford is the statistician who coordinated all analyses. The corresponding authors, who had unrestricted access to the data, prepared the drafts of the manuscript, which were then reviewed and edited by all authors, including representatives of Boehringer Ingelheim.

LIST OF PRINCIPAL INVESTIGATORS BY COUNTRY

Belgium

Stefan Janssens MD, UZ Leuven - Campus Gasthuisberg CardiologieHerestraat , Leuven

Wilfried Mullens MD, Ziekenhuis Oost-Limburg - Campus Sint-Jan Cardiology, Genk

Tom Sarens MD, AZ Sint-Blasius Kroonveldlaan 50, Dendermonde

Vincent Michiels MD, UZ Brussel Laarbeeklaan 101, Brussel

Pierre Troisfontaines MD, CHR de la Citadelle Cardiologie, Liège

Herbert De Raedt MD, Onze-Lieve-Vrouwziekenhuis - Campus Aalst, Aalst

Stéphane Carlier MD, CHU Ambroise Paré Service de cardiologie, Mons

Canada

Shelley Zieroth MD, St. Boniface General Hospital, Winnipeg

Elizabeth Swiggum MD, Royal Jubilee Hospital Research and Capacity Building, Victoria

Jay Udell MD, Toronto General Hospital, Toronto

China

Xiaoping Chen MD, West China Hospital of Sichuan University, Wuhou District, Chengdu City

Changsheng Ma MD, Beijing Anzhen Hospital Capital Medical University Cardiology, Chaoyang District, Beijing City

Yushi Wang MD, Jilin University First Hospital 1 Xinmin Street, Chaoyang District, Changchun City

Yan Wang MD, Xiamen Cardiovascular Hospital, Xiamen University, Huli District, Xiamen

Xinchun Yang MD, Beijing Chaoyang Hospital, Chaoyang District, Beijing

Zuyi Yuan MD, The First Affiliated Hospital of Xi 'an Jiaotong University, Yanta District, Xi 'an City

Czech Republic

Vaclav Durdil MD, University Hospital Motol Cardiology Dept., Prag

Lenka Spinarova MD, Univ.Hosp U Svate Anny, I.Internal Clinic-Cardiology, Brno

Marta Kaislerova MD, District Hospital Tabor Internal Department, Tabor

Petr Kala MD, University Hospital Brno, Department of Internal Cardiology Medicine, Brno

Denmark

M. Schou MD, Herlev and Gentofte Hospital Klinisk Biokemisk Afdeling, Herlev

C.D. Tuxen MD, Frederiksberg Hospital, Frederiksberg

A. Sjøl MD, Hvidovre Hospital, Hvidovre

M. Hollingdal MD, Viborg Regionhospital, Viborg

S. Vraa MD, Aalborg Universitetsshospital, Aalborg

Germany

Peter Lothar Schwimmbeck MD, PhD, Klinikum Leverkusen gGmbH, Akademisches Lehrkrankenhaus der Universität Köln, Leverkusen

Uwe Zeymer MD ,PhD, Klinikum der Stadt Ludwigshafen am Rhein gGmbH, Herzkatheterlabor, Klinische Studien, Ludwigshafen

P. Christian Schulze MD, PhD, Universitätsklinikum Jena, Klinik für Innere Medizin I Kardiologie, Internistische Intensivmedizin, Jena

Stefan Stoerk MD, PhD, Universitätsklinikum Würzburg, Medizinische Klinik und Poliklinik I, Würzburg

Harm Wienbergen MD, PhD Bremer Institut für Herz- und Kreislaufforschung (BIHKF) am Klinikum Links der Weser, Innere Medizin II, Bremen

Christoph Olivier MD, Universitäts-Herzzentrum Freiburg, Bad Krozingen GmbH Kardiologie und Angiologie I, Freiburg

Ingo Eitel MD, PhD Universitätsklinikum Schleswig-Holstein - Campus Lübeck Medizinische Klinik II / Kardiologie, Angiologie, Intensivmedizin, Lübeck

David Leistner MD, Charité - Universitätsmedizin Berlin, Medizinische Klinik für Kardiologie (CBF), Berlin

Birgit Aßmus MD, PhD, Universitätsklinikum Gießen und Marburg GmbH, Medizinische Klinik I, Innere Medizin/Kardiologie, Gießen

Marcus Mittag MD, Asklepios Klinik Langen-Seligenstadt GmbH, Medizinischen Klinik 1, Langen

Axel Linke MD, PhD Herzzentrum Dresden GmbH Universitätsklinik an der Technischen Universität Dresden, Klinik für Innere Medizin und Kardiologie, Dresden

Hungary

Kalman Toth MD, University of Pecs, Department of Cardiology, Pecs

Med Nagy Laszlo MD, Csongrad Country Dr Bugyi Istvan Hospital, Internal medicine, Szentes

Imre Zoltan Pozsonyi MD, Semmelweis University, 3rd Department of Internal Medicine, Budapest

Denes Pall MD, University Debrecen Hospital, Internal Medicine, Cardiology Department, Debrecen

Ebrahim Noori MD, Fejer County Saint George University Teaching Hospital, Internal Medicine Department, Szekesfehervar

Italy

Maurizio Volterrani MD, IRCCS San Raffaele, UO cardiologia Riabilitativa, Roma Lazio

Massimo Iacoviello MD, Az.Osp. Universitaria "Ospedali Riuniti U.O. di Cardiologia, FOGGIA

Massimo Piepoli MD, Osp. Guglielmo da Saliceto AUSL di Piacenza, Osp. Guglielmo da Saliceto, Piacenza

Salvatore De Rosa MD, Università degli Studi "Magna Grecia" - Campus "S. Venuta" CARDIOLOGIA, CATANZARO

Elio Gorga MD, ASST degli Spedali Civili di Brescia Cardiologia, BRESCIA

Piergiuseppe Agostoni, Centro Cardiologico Monzino-IRCCS, Milano

Simona D'Orazio MD, Ospedale della Val di Chiana Santa Margherita, Cortona (AR)

Fabrizio Oliva MD, ASST Grande Ospedale Metropolitano Niguarda, cardiologia – emodinamica, Milano

Gaetano De Ferrari MD, AO Città della Salute e della Scienza Presidio Molinette SC di Cardiologia,Torino

Gianfranco Sinagra MD, Azienda Sanitaria Universitaria Giuliano Isontina, CARDIOLOGIA,Trieste

Japan

Yasushi Sakata, MD, PhD, Osaka University Hospital, Cardiology, Osaka

Kazuki Fukui, MD, Kanagawa Cardiovascular and Respiratory Center, Cardiology, Kanagawa

Yasuo Okumura MD, Nihon University Itabashi Hospital, Cardiology, Tokyo

Koichiro Kuwahara MD, Shinshu University Hospital, Cardiology, Nagano

Naoki Sato MD, Kawaguchi Cardiovascular and Respiratory Hospital, Kawaguchi, Saitama,

Tomohito Ohtani MD, PhD, Osaka University Hospital Cardiology, Osaka

Masahiro Mori MD, Japan Community Health Care Organization Kyushu Hospital, Cardiology, Kitakyushu, Fukuoka

Tomomi Koizumi MD, Mito Medical Center, Cardiology, Ibaraki

Taro Shibasaki MD, Saitama Sekishikai Hospital, Cardiology, Sayama, Saitama

Akihiro Hayashida MD, The Sakakibara Heart Institute of Okayama, Cardiology, Okayama,

Netherlands

Prof. Adriaan Voors MD, University of Groningen, University Medical Center Groningen, Groningen

M. van Eck MD, Jeroen Bosch Ziekenhuis , Department of Cardiology, ’s Hertogenbosch

Lennaert Kleijn MD, TREANT Zorggroep, Department of Cardiology, Emmen

Tjeerd Römer MD, Alrijne Leiderdorp, Department of Cardiology, Leiderdorp

Anastazia Jerzewski MD, Gelre Ziekenhuizen Apeldoorn, Department of Cardiology, Apeldoorn

Reinhart Dorman MD, Bravis ziekenhuis, locatie Roosendaal, Department of Cardiology, Roosendaal

C. Jan Willem Borleffs MD, Haga Teaching Hospital, Den Hague

Jaco Houtgraaf MD, Diakonessenhuis Utrecht, Department of Cardiology, Utrecht

Eric Viergever MD, Groene Hart ziekenhuis, Department of Cardiology, Gouda

Eugene van Beek MD, Sint Jansdal Ziekenhuis, Department. of Cardiology, Harderwijk

Norway

Henrik Schirmer MD, Akershus Universitetssykehus HF, Hjertemedisinsk Avdeling, Medisinsk Divisjon, LØRENSKOG

Volker Pönitz MD, Helse Stavanger MD, Stavanger Universitetssykehus, Kardiologisk avdeling, STAVANGER

Johnsen Wold MD, Åse Katrine , Helse Førde HF, Førde Sentralsjukehus, Medisinsk poliklinikk, FØRDE

Geir Høgalmen MD, Sykehuset Innlandet HF, Avd. Lillehammer, Medical department, LILLEHAMMER

David Johansen MD, Universitetssykehuset Nord-Norge, Tromsø, HLK - Hjertemedisinsk Avdeling, TROMSØ

Poland

Grzegorz Piotrowski MD, Kopernik's Specialized Hospital, Cardiology Department, Lodz Jerzy K.

Wranicz MD, Cent.Clin.Hosp.Med.Univ.Lodz,Electrocard Central Clinical Hospital Medical University of Lodz, Lodz

Pawel Miekus MD, Saint Wincenty a Paulo Hosp., Cardiology Dept., Gdynia, Gdynia

Malgorzata Lelonek MD, Card.Cli.Mil.Med.Ac.Uni.Cli.Hosp. Cent.Vetera.Hosp.Lodz, Medical University of Lodz, Lodz

Spain

Domingo Pacual Figal MD, Hospital Universitario Virgen de la Arrixaca, Servicio de Cardiología,Murcia

Josep Comin-Colet MD, Hospital Universitari de Bellvitge, Servicio de Cardiología, Barcelona

Marta Cobo MD, Hospital Puerta de Hierro, Hospital Universitario Puerta de Hierro Majadahonda Servicio de Cardiología, Majadahonda

José Manuel García Pinilla MD, Hospital Universitario Virgen de la Victoria, Servicio de Cardiología, Malaga

Julio Eduardo Núñez MD, Hospital Clínico Universitario de Valencia, Servicio de Cardiología, Valencia

Antonio Reyes Domínguez MD, Hospital Nuestra Señora de Valme, Servicio de Medicina Interna, Sevilla

Román Freixa MD, Hospital Moises Broggi, Servicio de Cardiología, Barcelona

Sweden

Michael Fu MD, Sahlgrenska Universitetssjukhuset Östra, Kardiologsektionen/Medicinkliniken, Göteborg

N Bergh MD, Sahlgrenska Universitetssjukhuset Forskningsavdelningen, Göteborg

United States

William J. French MD, Lundquist Institute for Biomedical Innovation at Harbor-UCLA Medical Center, Torrance

Kirkwood F. Adams MD, The University of North Carolina at Chapel Hill, Clinical and Translational Research Center, Chapel Hill

Ravi Dhingra MD, University of Wisconsin Hospital and Clinics, Madison

Hal Andrew Skopicki MD, Stony Brook Medicine, Stony Brook,

Joseph L. Izzo MD, Erie County Medical Center, Department of Medicine, Buffalo,

Aasim Afzal MD, Center for Advanced Cardiac Care - Heart Failure Clinic, Plano,

Franchi Francesco MD, University of Florida Health Jacksonville, Jacksonville

Dilip B. Viswanath MD, Jefferson Washington Township Hospital, Washington Township

Mark D. Gelernt MD, Cardiovascular Associates of the Delaware Valley, Elmer

Robert Craig Long MD, University Of Mississippi Medical Center, Jackson,

Terrence X. O'Brien MD, Ralph H. Johnson Veterans Affairs Medical Center, Charleston

Christopher Vernin Chien MD, North Carolina Heart and Vascular - Rex Heart Failure Clinic, Raleigh

Marwa A. Sabe MD, Beth Israel Deaconess Medical Center, Boston,

Michael W Fong MD, University of Southern California Keck School of Medicine, Division of Cardiovascular Medicine, Los Angeles

Luanda Grazette MD, University of Southern California Keck School of Medicine, Division of Cardiovascular Medicine, Los Angeles

Jin Ho Han MD, Vanderbilt University Medical Center, Nashville,

David Alan Henderson MD, Cardiology Associates Research Company, Daytona Beach

Lauren Beth Cooper MD, Inova Fairfax Medical Campus, Falls Church

Naeem Khan Tahirkheli MD, South Oklahoma Heart Research Group, Oklahoma City

Modele O Ogunniyi MD, Grady Memorial Hospital, Atlanta,

David Gregory Brabham MD, Pharmatex Research, Amarillo,

Jignesh K. Patel MD, Cedars-Sinai Medical Center, Advanced Heart Disease Clinic, Los Angeles,

Dawn M. Lombardo MD, University of California Irvine Cardiovascular Center, Orange,

David Alan Baran MD, Sentara Norfolk General Hospital, Norfolk,

Tarun Watson Dasari MD, University of Oklahoma Medical Center, Oklahoma City

Snehal R Patel MD, Montefiore Medical Center, Medical Arts Pavilion, Bronx

Ronald Zolty MD, University of Nebraska Medical Center Physicians, Heart Center, Omaha,

Rita Anne Jermyn MD, The DeMatteis Center for Cardiac Research and Education, Greenvale,

Alan J. Bank MD, United Hospital, St. Paul,

Michael E. Nassif MD, Saint Luke's Hospital of Kansas City, Mid America Heart Institute, Kansas City

Alexander Adler MD, Methodist Medical Center, Methodist Research Administration Office, Glen Oak

KEY INCLUSION AND EXCLUSION CRITERIA

Key Inclusion criteria

1. ≥18 years currently hospitalized for the primary diagnosis of acute HF
2. Currently hospitalized for the primary diagnosis of acute heart failure (de novo or decompensated chronic HF), regardless of EF. Patients with a diagnosis of hospitalized heart failure must have the following HF signs and symptoms at the time of hospital admission:
   - Dyspnoea (exertional or at rest) and at least two of the following signs of fluid overload:
     i. Congestion on chest X-ray
     ii. Rales on chest auscultation
     iii. Clinically relevant oedema (e.g. ≥1+ on a 0 to 3+ scale)
     iv. Elevated jugular venous pressure
3. Evidence of left ventricular ejection fraction (LVEF, either reduced or preserved EF) as per local reading preferably measured during current hospitalization or in the 12 months prior to randomization
4. Patients must be randomized after at least 24 hours and no later than 5 days after admission, as early as possible after stabilization and while still in hospital
5. Patients must fulfil the following stabilization criteria (while in the hospital):
   - SBP ≥100mmHg and no symptoms of hypotension in the preceding 6 h
   - No increase in i.v. diuretic dose for 6 h prior to randomization
   - No i.v. vasodilators including nitrates within the last 6 h prior to randomization
   - No i.v. inotropic drugs for 24 h prior to randomization
6. Elevated NT-proBNP ≥1600 pg/mL or BNP ≥400 pg/mL (50% more for patients with AF) measured during index hospitalization, or in the 72 h prior to hospital admission. For patients treated with an angiotensin receptor neprilysin inhibitor (ARNI) in the previous 4 weeks prior to randomization, only NT-proBNP values should be used

7. HF episode leading to hospitalisation must have been treated with a minimum single dose of 40 mg of i.v. furosemide (or equivalent i.v. loop diuretic defined as 20 mg of torasemide or 1 mg of bumetanide)

Key Exclusion criteria

1. Cardiogenic shock
2. Current hospitalization for acute HF primarily triggered by pulmonary embolism, cerebrovascular accident, or acute myocardial infarction
3. Current hospitalization for acute heart failure not caused primarily by intravascular volume overload.
4. Interventions in the past 30 days prior to randomization or planned during the study:
   - Major cardiac surgery, or TAVI, or PCI, or MitraClip
   - All other surgeries that are considered major according to investigator judgement
   - Implantation of cardiac resynchronization therapy device
   - Cardiac mechanical support implantation
   - Carotid artery disease revascularization
5. Acute coronary syndrome/myocardial infarction, stroke or transient ischemic attack in the past 90 days prior to randomization

6. Heart transplant recipient, or listed for heart transplant with expectation to receive a transplant during the course of this trial (according to investigator judgement), or planned for palliative care for HF, or currently using left ventricular assist device (LVAD) or intra-aortic balloon pump (IABP) or any other type of mechanical circulatory support, or patients on mechanical ventilation, or patients with planned inotropic support in an outpatient setting
7. Haemodynamically severe uncorrected primary cardiac valvular disease planned for surgery or intervention during the course of the study
8. eGFR <20 mL/min/1.73m2 during hospitalization (latest local lab measurement before randomization) or patients requiring dialysis
9. Type 1 diabetes mellitus
10. History of ketoacidosis, including diabetic ketoacidosis

MAJOR PROTOCOL-SPECIFIED EFFICACY ENDPOINTS

Primary endpoint

The primary endpoint was clinical benefit at 90 days, defined as a hierarchical composite outcome of time to all-cause death, the number of HF events (HFE), time to first HFE and a ≥ 5-point difference in change from baseline in KCCQ total symptom score (KCCQ-TSS) after 90 days of treatment.

An HFE was:
- A hospitalization/ER visit OR
- An urgent care visit OR
- An Outpatient visit

With all of the following:
- The question "Was visit related to the primary diagnosis or worsening of heart failure in the opinion of the treating physician?" is answered yes
- At least one symptom of:
    - dyspnea
    - decreased exercise tolerance
    - fatigue
    - edema
    - other symptoms of worsened end-organ perfusion or volume overload
- At least two physical examination findings OR (one physical examination finding and at least one laboratory criterion).
    - Physical examinations are:
        - peripheral edema,
        - increased abdominal distention or ascites,
        - pulmonary rates/crackles/crepitations,
        - Increased jugular venous pressure and/or hepatojugular reflux,
        - S3 gallop,
        - Clinically significant or rapid weight gain thought to be related to fluid distention,
        - other
    - Laboratory criteria are:
        - Increased BNP or NT-proBNP,
        - radiological *or ultrasonographic* evidence of pulmonary congestions,
        - non-invasive diagnostic evidence of clinically significant elevated ventr. filling pressure or low cardiac output
        - invasive diagnostic evidence with right heart catheterization showing a pulmonary capillary wedge pressure >=18 mmHG, central venous pressure >=12 mmHg or cardiac index <2.2 L/min/m²
        - Other
- Intensification of therapy (significant augmentation of oral diuretics, IV diuretics, vasoactive agent, or mechanical or surgical intervention)

Exploratory endpoints (pre-specified)

All other endpoints other than primary endpoint are considered exploratory and included an improvement in KCCQ-TSS of ≥10 points after 90 days of treatment, Change from baseline in KCCQ-TSS after 90 days of treatment, change from baseline in log-transformed NT-proBNP AUC over 30 days, time to first occurrence of CV death or HFE, occurrence of HHF until 30 days after initial hospital discharge, days alive and out of

hospital from study drug initiation until 30 days after initial hospital discharge, days alive and out of hospital from study drug initiation until 90 days after randomization, and diuretic response during follow up (defined as kg of weight loss per daily unit of furosemide) after 15 days and 30 days. Safety parameters included markers of volume depletion, hypotension, and Occurrence of chronic dialysis or renal transplant or sustained reduction of ≥40% estimated glomerular filtration rate (eGFR) Chronic Kidney Disease Epidemiology Collaboration Equation ((CKD-EPI)cr), or o sustained eGFR (CKD-EPI)cr.

METHODOLOGY USED FOR THE PRIMARY ENDPOINT (WIN RATIO)

The primary endpoint was assessed using a stratified win ratio approach. The statistical model was a non-parametric generalized pairwise comparison within HF status strata.

For any two patients, a patient would win, i.e. achieve a better clinical outcome, as determined by assessing the following criteria sequentially, and stopping when an advantage for either patient was shown:

1. Death within common follow-up time
   - death is worse than no death
   - earlier death is worse
   - tied, if not possible to determine

2. Number of HFEs within common follow-up time
   - more HFEs is worse
   - tied, if same number of HFEs

3. Time to first HFE within common follow-up time
   - earlier HFE is worse
   - tied, if not possible to determine

4. KCCQ-TSS change from baseline at Day 90
   - more positive change from baseline is better
   - the threshold for the difference is >= 5 for a win
   - tied, if difference < 5

Note, priority was therefore given to death over HFE, and both of these over changes in KCCQ-TSS. Below are some examples:

1. Death, e.g.:
   - Patient A dies 30 days after randomization (loses)
   - Patient B dies 40 days after randomization (wins)

2. If no winner based on death, number of HFEs within common follow-up time, e.g.:
   - Patient A had two HFEs (loses)
   - Patient B had one HFE (wins)

3. If no winner based on number of HFEs, time to first HFE, e.g.:
   - Patient A had an HFE 30 days after randomization (loses)
   - Patient B had an HFE 50 days after randomization (wins)

4. If no winner based on time to first HFE, KCCQ-TSS change from baseline at Day 90, e.g.:

- Patient A: KCCQ-TSS change from baseline at Day 90 is 5 (loses)
- Patient B: KCCQ-TSS change from baseline at Day 90 is 11 (wins)

The implemented generalized pairwise comparisons approach compared all patients in one treatment group to all other patients within their strata in the other treatment group.

Applying weights that are analogous to a Mantel-Haenszel approach (1), the stratified win ratio is:

$$WR = \frac{\sum_{m=1}^{2} n_e^{(m)} / N^{(m)}}{\sum_{m=1}^{2} n_p^{(m)} / N^{(m)}}$$

where m is the stratum number (m = 1, 2), $n_e^{(m)}$ is the number of wins in the empagliflozin group in the mth stratum, $n_p^{(m)}$ is the number of wins in the placebo group in the mth stratum and $N^{(m)}$ is the total number of patients in the mth stratum.

The variance was calculated by the asymptotic normal U statistics approach (2)

Missing data for the KCCQ-TSS (as part of the stratified win ratio) was estimated using multiple imputation (MI), according to whether patients were on-treatment or off-treatment.

In brief: The intention was to impute missing data using available data. We intended to do so separately for on-treatment data and use the placebo course to impute off-treatment data (this is called a “jump-to-placebo” approach). In addition, baseline and previous KCCQ data, as well as HF status (de-novo vs chronic HF) was used.

In detail: Values measured after premature discontinuation of study drug were included in the analyses. All available on- and off-treatment data up to the planned measurement at Day 90 was included.

Different types of missing data were considered for the imputation as follows:

Missing data imputation (Assumption of missingness)	Non-monotone	Monotone
On-treatment data	MCMC-MI[1] (MAR[3])	SLR-MI[2] (MAR)
Off-treatment[4] data	Jump-to-Placebo (MNAR[5])	Jump-to-Placebo (MNAR)

[1] Markov Chain Monte Carlo – Multiple imputation (MCMC-MI)
[2] Sequential linear regression – Multiple imputation (SLR-MI)
[3] Missing at random (MAR)
[4] Any instance of a patient discontinuing treatment will lead to all future measurements being considered as off-treatment.
[5] Missing not at random (MNAR)

As the first step, non-monotone on-treatment missing data was imputed using Markov Chain Monte Carlo (MCMC) simulation and standard techniques; MI was performed separately by treatment with baseline value as a continuous variable and HF status as a binary variable. One hundred imputations were performed to ensure adequate efficiency and stability of the estimation for missing data.

For the monotone missing on-treatment data, a sequential linear regression MI (SLR-MI) approach was used. The MI was performed on a data set only including on-treatment data and once per imputation from the previous step. This procedure imputed values for all missing time points both on- and off-treatment, so imputations for off-treatment values were then deleted. The regression models were fitted separately by treatment and included the baseline value as a continuous covariate, HF status as a class covariate and the values at previous time points as separate continuous covariates.

To impute the missing off-treatment data, a residual-based approach was used to enact the 'jump-to-placebo'. Throughout this imputation, all observed off-treatment data was temporarily removed and added back at the end. A covariate-adjusted difference between the relevant treatment arm and placebo (i.e. 0 for those in the placebo arm) was calculated at each visit using ANCOVA (with HF status and treatment as categorical covariates and baseline value as a continuous covariate). Next, the relevant estimated treatment difference was deducted from all observed data in the active treatment group. Note that the adjustments were for the duration of the imputation only and the original values for the observed data were in the final imputed data set.

SLR-MI was then performed once per imputation upon this modified data but only including patients either with missing values and/or those in the placebo arm, ensuring imputations were performed using the placebo distribution. The models included baseline value as a continuous covariate, HF status as a class covariate and the values at previous time points as separate continuous covariates. All missing data therefore followed the distribution of the placebo arm, jumping from one arm's distribution to the placebo's arm at the point of missingness, while still accounting for past within-treatment performance (the residuals). After this imputation, all the original observed values (i.e. off-treatment values) were restored.

The practical implementation proceeded as follows:

1. Off-treatment data was removed from dataset

2. Non-monotone missing data was imputed under MAR to create a monotone pattern
    a. 100 imputations were performed by MCMC-MI, stratified by treatment, with the following parameters:
        i. Baseline value included as a continuous variable, HF status as a binary variable (de novo or decompensated chronic HF)
        ii. Multiple chains with 200 burn-in iterations per chain
        iii. Jeffrey's prior

3. SLR-MI was used to impute missing monotone on-treatment data:
    a. All data was imputed using SLR-MI, separately by treatment, with the following parameters:
        i. Baseline (continuous), HF status (de novo or decompensated HF) included as covariates
    b. All imputed data for patients at an off-treatment time point was deleted

4. On-treatment group means were calculated for each imputation at each visit
    a. ANCOVA used to calculate adjusted means with the following parameters:
        i. Baseline (continuous), HF status (de novo or decompensated chronic HF), and treatment included as covariates
        ii. Restricted Maximum Likelihood and Kenward-Rogers methods
        iii. Least Square Means used

5. Placebo group on-treatment means at each visit were subtracted from all group on-treatment means at the same visit, to give treatment differences
    a. Note that these were 0 for the placebo group

6. Individual data was ‘temporarily saved’ at this point, excluding available off-treatment data

7. Treatment differences were subtracted from the respective (by visit and treatment) data values
    a. Results were the ‘residuals’ after removal of treatment effect(s)
    b. This was referred to as the residual data

8. A subset of the residual data was created with all patients in the placebo group and all patients with missing values at first visit requiring imputation. Note that these missing values were all off-treatment.

9. Multiple imputation was performed on the first visit requiring imputation
    a. Linear regression models were used to derive the distributional parameters;
        i. One imputation per existing imputation
        ii. Residual subset used
        iii. Baseline (continuous), HF status (de novo or decompensated chronic HF), all previous visits (continuous) included as model covariates

10. Imputed off-treatment values from subset merged with residual data sets.

11. If visits that required imputation remained, step 8 was repeated, else it proceeded to the next step

12. Original data set, saved data sets and residual data sets were merged so that all missing values in the saved data set were filled with observed off-treatment measurements, where available, otherwise the values from corresponding residual data sets were used.

13. The stratified win ratio was calculated using these imputed data sets, by combining with the non-imputed death and HFE data. Then logs of the stratified win ratios were taken.

14. Estimates and variances were combined using Rubin’s rules to give a single estimate and variance for each parameter of interest across all imputations.

References for the Win-ratio

1. Dong G, Qiu J, Wang D, Vandemeulebroecke M. The stratified win ratio. Journal of Biopharmaceutical Statistics 2018;28:778–96

2. Dong G, Li D, Ballerstedt S, Vandemeulebroecke M. A generalized analytic solution to the win ratio to analyze a composite endpoint considering the clinical importance order among components. Pharm Stat. 2016. 15: 430-437
