# Supplementary material for: The SGLT2 inhibitor empagliflozin in patients hospitalized for acute heart failure: a multinational randomized trial
Source: Nat Med. 2022 Feb 28;28(3):568–74. doi: 10.1038/s41591-021-01659-1 (PMC8938265; doi:10.1038/s41591-021-01659-1)
Supplement: Supplementary file 4 — Supplementary Note 2 [file 41591_2021_1659_MOESM4_ESM.pdf]

This file contains the following items:

1. EMPULSE clinical trial protocol (CTP), version 1 p. 2
2. EMPULSE CTP, final version including a summary of changes p. 83

Clinical Trial Protocol

**Document Number:**		**c28603082-01**
**EudraCT No.**	**2019-002946-19**	
**BI Trial No.**	1245-0204	
**BI Investigational Medicinal Product(s)**	Jardiance®, empagliflozin	
**Title**	A multicentre, randomised, double-blind, 90-day superiority trial to evaluate the effect on clinical benefit, safety and tolerability of once daily oral **EMP**agliflozin 10 mg compared to placebo, initiated in patients hospitalised for ac**U**te heart fai**L**ure (de novo or decompensatied chronic HF) who have been **S**tabilis**E**d (**EMPULSE**)	
**Lay Title**	A study to test the effect of empagliflozin in patients who are in hospital for acute heart failure	
**Clinical Phase**	III	
**Clinical Trial Leader**	[redacted]	
**Coordinating Investigators**	[redacted]	
**Version and Date**	**Version:** 1.0	**Date:** 09 Oct 2019

Proprietary confidential information.
© 2019 Boehringer Ingelheim International GmbH or one or more of its affiliated companies. All rights reserved.
This document may not - in full or in part - be passed on, reproduced, published or otherwise used without prior written permission.

Proprietary confidential information © 2019 Boehringer Ingelheim International GmbH or one or more of its affiliated companies

CLINICAL TRIAL PROTOCOL SYNOPSIS

**Company name**	Boehringer Ingelheim
**Protocol date**	09 Oct 2019
**Revision date**	Not applicable
**BI trial number**	1245-0204
**Title of trial**	A multicentre, randomised, double-blind, 90-day superiority trial to evaluate the effect on clinical benefit, safety and tolerability of once daily oral **EMP**agliflozin 10 mg compared to placebo, initiated in patients hospitalised for ac**U**te heart fai**L**ure (de novo or decompensated chronic HF) who have been **S**tabilis**E**d (**EMPULSE**)
**Coordinating Investigators**	[redacted]
**Trial site(s)**	Multi-centre trial
**Clinical phase**	III
**Trial rationale**	Other trials with empagliflozin are performed to assess mortality and morbidity, functional capacity and safety of empagliflozin in patients with chronic heart failure under stable conditions (i.e. after hospital discharge).<br>Due to its mode of action (MOA), empagliflozin is expected to potentially alleviate congestive symptoms seen in patients shortly after initial stabilisation of acute cardiac decompensation helping to improve heart failure (HF)-related outcomes within several weeks after discharge from hospital.<br>In-hospital initiation of different therapies is one of the best predictors of long-term adherence to medications and long-term improved prognosis.
**Trial objective(s)**	The main objective of this study is to assess whether in-hospital administration of empagliflozin results in improvement in HF-related outcomes (clinical benefit including death, heart failure events (HFE)

Proprietary confidential information © 2019 Boehringer Ingelheim International GmbH or one or more of its affiliated companies

	and the burden of symptoms and physical limitations as assessed by the Kansas City Cardiomyopathy Questionnaire (KCCQ) clinical summary score) in patients hospitalised for acute heart failure (de novo or decompensated chronic HF) and after initial stabilisation. Secondary objectives are to further assess whether it is safe to start empagliflozin in patients admitted to hospital in this setting.
**Trial endpoints**	Primary Endpoint:<br>• Net clinical benefit, a composite of death, number of heart failure events (HFEs) (including hospitalisations for heart failure (HHFs), urgent heart failure visits and unplanned outpatient visits), time to first HFE and change from baseline in Kansas City Cardiomyopathy Questionnaire - Clinical Summary Score (KCCQ-CSS) after 90 days of treatment.<br>Secondary Endpoints:<br>• Proportion of patients with a clinically meaningful improvement in KCCQ-CSS of ≥ 10 points after 90 days of treatment<br>• Change from baseline in KCCQ CSS after 90 days of treatment<br>• Change from baseline in log-transformed N-Terminal Pro-Brain Natriuretic Peptide (NT-proBNP) level over 30 days of treatment (area under the curve (AUC)).<br>• Days alive and out of hospital from study drug initiation until 30 days after initial hospital discharge<br>• Days alive and out of hospital from study drug initiation until 90 days after randomisation<br>• Time to first occurrence of cardiovascular (CV) death or HFE until end of trial visit<br>• Occurrence of HHF until 30 days after initial hospital discharge<br>• Occurrence of chronic dialysis or renal transplant or sustained reduction of ≥40% estimated glomerular filtration rate (eGFR) Chronic Kidney Disease Epidemiology Collaboration Equation ((CKD-EPI)cr), or<br>o sustained eGFR (CKD-EPI)cr <15 mL/min/1.73 m^2 for patients with baseline eGFR ≥30 mL/min/1.73 m^2<br>o sustained eGFR (CKD-EPI)cr <10 mL/min/1.73 m^2 for patients with baseline eGFR <30 mL/min/1.73 m^2<br>• Diuretic effect as assessed by weight loss per mean daily loop diuretic dose after 15 days of treatment<br>• Diuretic effect as assessed by weight loss per mean daily loop diuretic dose after 30 days of treatment
**Trial design**	Randomised, double-blind, parallel-group, placebo controlled, multinational and multicentre study
**Total number of patients randomised**	Approximately 500
**Number of patients on each treatment**	Approximately 250
**Diagnosis**	Patients admitted to hospital for treatment of acute HF (de novo or decompensated chronic HF). Patients should be randomised after at

Proprietary confidential information © 2019 Boehringer Ingelheim International GmbH or one or more of its affiliated companies

	least 24 hours and no later than 5 days after hospital admission. Randomisation should occur as soon as the patient is stabilised (see inclusion criterion 7).
**Main in- and exclusion criteria**	Main Inclusion Criteria:<br>• Currently hospitalised for the primary diagnosis of acute heart failure (de novo or decompensated chronic HF), regardless of ejection fraction (EF). Patients with a diagnosis of hospitalised heart failure must have HF symptoms at the time of hospital admission<br>• Evidence of left ventricular ejection fraction (LVEF, either reduced or preserved EF) as per local reading preferably measured during current hospitalisation or in the 12 months prior to randomisation<br>• Patients must be randomised after at least 24 hours and no later than 5 days after admission, as early as possible after stabilisation and while still in hospital<br>• Patients must fulfil the following stabilisation criteria (while in the hospital):<br>- SBP ≥100mm Hg and no symptoms of hypotension in the preceding 6 hours,<br>- no increase in i.v. diuretic dose for 6 hours prior to randomisation,<br>- no i.v. vasodilators including nitrates within the last 6 hours prior to randomisation<br>- no i.v. inotropic drugs for 24 hours prior to randomisation.<br>• Elevated NT-proBNP ≥ 1600pg/mL or BNP ≥400 pg/mL according to the local lab, for patients without atrial fibrillation (AF); or elevated NT-proBNP ≥ 2400pg/mL or BNP ≥600 pg/mL for patients with AF, measured during the current hospitalisation or in the 72 hours prior to hospital admission,. For patients treated with an angiotensin receptor neprilysin inhibitor (ARNI) in the previous 4 weeks prior to randomisation, only NT-proBNP values should be used<br>• HF episode leading to hospitalisation must have been treated with a minimum dose of 40 mg of i.v. furosemide (or equivalent i.v. loop diuretic defined as 20 mg of torasemide or 1 mg of bumetanide)<br><br>Main Exclusion Criteria:<br>• Cardiogenic shock<br>• Current hospitalisation for acute heart failure primarily triggered by pulmonary embolism, cerebrovascular accident, or acute myocardial infarction (AMI)<br>• Current hospitalisation for acute heart failure not caused primarily by intravascular volume overload;<br>• Below interventions in the past 30 days prior to randomisation or planned during the study:

Proprietary confidential information © 2019 Boehringer Ingelheim International GmbH or one or more of its affiliated companies

	- Major cardiac surgery, or TAVI (Transcatheter Aortic Valve Implantation), or PCI, or Mitraclip<br>- All other surgeries that are considered major according to investigator judgement<br>- Implantation of cardiac resynchronisation therapy (CRT) device<br>- cardiac mechanical support implantation<br>- Carotid artery disease revascularisation (stent or surgery)<br>• Acute coronary syndrome / myocardial infarction, stroke or transient ischemic attack (TIA) in the past 90 days prior to randomisation<br>• Heart transplant recipient, or listed for heart transplant with expectation to receive a transplant during the course of this trial (according to investigator judgement), or planned for palliative care for HF, or currently using left ventricular assist device (LVAD) or intra-aortic balloon pump (IABP) or any other type of mechanical circulatory support, or patients on mechanical ventilation, or patients with planned inotropic support in an out-patient setting<br>• Haemodynamically significant (severe) uncorrected primary cardiac valvular disease planned for surgery or intervention during the course of the study (note: secondary mitral regurgitation or tricuspid regurgitation due to dilated cardiomyopathy is not excluded unless planned for surgery or intervention during the course of the study)<br>• Impaired renal function, defined as eGFR < 20 mL/min/1.73 m^2 as measured during hospitalisation (latest local lab measurement before randomisation) or requiring dialysis<br>• Type 1 Diabetes Mellitus (T1DM)<br>• History of ketoacidosis, including diabetic ketoacidosis (DKA)
**Test product(s)**	Empagliflozin
**dose**	10 mg q.d.
**mode of administration**	Oral (p.o.)
**Comparator product(s)**	Placebo
**dose**	Not applicable
**mode of administration**	Oral (p.o.)
**Duration of treatment**	90 days
**Statistical methods**	The primary endpoint is the net clinical benefit (defined in Section 7.2.2). The statistical model will be a non-parametric generalised pairwise comparison within HF status strata. The variance of the net clinical benefit will be calculated using the asymptotic normal approach.

Proprietary confidential information © 2019 Boehringer Ingelheim International GmbH or one or more of its affiliated companies

FLOW CHART

**Trial Periods**	**Screen**	**Randomised Treatment**							**Follow-up**
**Visit**	**1**	**2 (in hospital)**			**3**	**4**	**5 (EoT)**	**Early Discon-tinuation Visit[3]**	**6 (FUP) (Phone Call)**
		**a**	**b[2]**	**c[2]**					
**Days**	-4 to 1	1[1]	3	5	15	30	90		EoT + 7 days
Time window for visits (days)		None	±1	±1	±3	±3	±7		+ 7
Informed consent[4]	X								
Demographics	X								
Medical history	X								
Physical examination	X						X	X	
Review of in-/exclusion criteria	X	X							
Randomisation (IRT)		X							
Weight		X			X	X	X	X	
Height		X							
Vital signs[5]		X	X	X	X	X	X	X	
Safety lab: blood tests[6]		X	X	X	X	X	X	X	
Safety lab: urinalysis including ketones[6]		X			X	X	X	X	
Urine Pregnancy test[7]	X						X[12]	X	
HbA1c[6]		X					X	X	
eGFR (CKD-EPI)cr formula[6]		X	X	X	X	X	X	X	
NT-proBNP (central lab)[6,8]		X	X	X	X	X	X	X	
12 lead-ECG[9]		X					X[12]	X	
KCCQ		X			X	X	X	X	
Clinical Congestion Score		X	X	X	X		X	X	
NYHA Classification		X			X	X	X	X	
HCRU		X			X	X	X	X	
All AEs/SAEs/AESIs[10]	X	X	X	X	X	X	X	X	X
Concomitant therapy		X	X	X	X	X	X	X	
Dispense trial medication		X				X			
Compliance check					X	X	X	X	
Return medication						X	X	X	
Completion of patient participation (End of Study (EOS))							(X)[11]		X

1 Day of Randomisation / Day of first intake of randomised medication. All Visit 2a assessments must be performed before the first dose is taken in hospital. Assessments from Visit 2a will be used as baseline values.

2 Visit 2b and 2c will only be performed, if the patient is still in hospital.

3 Patients who discontinue trial treatment prematurely should undergo the Early Discontinuation Visit as soon as possible and the Follow-up (FUP) Visit 7 days thereafter. Patients should be followed up until Visit 5 (day 90) according to their planned visit schedule.

4 Written informed consent must be obtained before any other assessment is performed.

Proprietary confidential information © 2019 Boehringer Ingelheim International GmbH or one or more of its affiliated companies

[5] Blood pressure and heart rate will be measured with the patient seated and rested for at least 5 minutes. Vital signs at Visit 2a need to be checked before first trial medication intake.

[6] Samples for central lab (including NT-proBNP) at Visit 2a need to be taken before first trial medication intake.

[7] Women of childbearing potential only.

[8] For patient's eligibility, local lab should be used, including a locally performed BNP or NTproBNP result taken during the current hospitalisation or in the 72 hours prior hospital admission. At the time the local BNP or NT-proBNP sample is collected, the baseline rhythm (e.g. sinus rhythm, AF) of the patient must be clarified using ECG or other measures (for example continuous heart monitoring) and documented.

[9] In addition to the scheduled ECGs, ECGs should be done and documented at any time of clinical event (e.g. acute event of arrhythmia, tachy- or bradycardia, angina, or MI).

[10] After the individual patient's end of the trial the investigator should only report any occurrence of cancer, related SAEs and related AESIs of which the investigator may become aware of and only via the SAE form, please see Section 5.2.7.2.1.

[11] Patients who prematurely discontinue medication are followed up according to protocol, and their visit schedule will end at their scheduled Visit 5.

[12] Urinary pregnancy test and ECG do not need to be repeated if patient prematurely discontinued trial medication and these assessments were done at the Early Discontinuation Visit.

Proprietary confidential information © 2019 Boehringer Ingelheim International GmbH or one or more of its affiliated companies

TABLE OF CONTENTS

TITLE PAGE 1

CLINICAL TRIAL PROTOCOL SYNOPSIS 2

FLOW CHART 6

TABLE OF CONTENTS 8

ABBREVIATIONS 11

1. INTRODUCTION 15

1.1 MEDICAL BACKGROUND 15
1.2 DRUG PROFILE 16
1.3 RATIONALE FOR PERFORMING THE TRIAL 17
1.4 BENEFIT - RISK ASSESSMENT 19
1.4.1 Benefits 19
1.4.2 Risks 20
1.4.3 Discussion 24

2. TRIAL OBJECTIVES AND ENDPOINTS 25

2.1 MAIN OBJECTIVES, PRIMARY AND SECONDARY ENDPOINTS 25
2.1.1 Main objectives 25
2.1.2 Primary endpoint(s) 25
2.1.3 Secondary endpoint(s) 25
2.2 FURTHER OBJECTIVES AND FURTHER ENDPOINTS 26
2.2.1 Further objectives 26
2.2.2 Further endpoints 26

3. DESCRIPTION OF DESIGN AND TRIAL POPULATION 28

3.1 OVERALL TRIAL DESIGN 28
3.2 DISCUSSION OF TRIAL DESIGN, INCLUDING THE CHOICE OF CONTROL GROUP(S) 29
3.3 SELECTION OF TRIAL POPULATION 30
3.3.1 Main diagnosis for trial entry 31
3.3.2 Inclusion criteria 31
3.3.3 Exclusion criteria 32
3.3.4 Withdrawal of patients from treatment or assessments 35
3.3.4.1 Discontinuation of trial treatment 35
3.3.4.2 Withdrawal of consent to trial participation 36
3.3.4.3 Discontinuation of the trial by the sponsor 36

4. TREATMENTS 38

4.1 INVESTIGATIONAL TREATMENTS 38
4.1.1 Identity of the Investigational Medicinal Products 38
4.1.2 Selection of doses in the trial and dose modifications 38
4.1.3 Method of assigning patients to treatment groups 39
4.1.4 Drug assignment and administration of doses for each patient 39
4.1.5 Blinding and procedures for unblinding 40

Proprietary confidential information © 2019 Boehringer Ingelheim International GmbH or one or more of its affiliated companies

4.1.5.1 Blinding 40
4.1.5.2 Unblinding and breaking the code 40
4.1.6 Packaging, labelling, and re-supply 41
4.1.7 Storage conditions 41
4.1.8 Drug accountability 41
4.2 OTHER TREATMENTS, EMERGENCY PROCEDURES, RESTRICTIONS 42
4.2.1 Other treatments and emergency procedures 42
4.2.2 Restrictions 43
4.2.2.1 Restrictions regarding concomitant treatment 43
4.2.2.2 Restrictions on diet and life style 43
4.2.2.3 Contraception requirements 43
4.3 TREATMENT COMPLIANCE 43
5. ASSESSMENTS 45
5.1 ASSESSMENT OF EFFICACY 45
5.1.1 Net Clinical Benefit 45
5.1.2 NT-proBNP and BNP 45
5.1.3 Clinical Congestion Score 45
5.1.4 New York Heart Association classification 46
5.2 ASSESSMENT OF SAFETY 47
5.2.1 Physical examination 47
5.2.2 Vital signs 47
5.2.3 Body weight and height 47
5.2.4 Safety laboratory parameters 48
5.2.4.1 Renal Function 49
5.2.4.2 Pregnancy Testing 50
5.2.4.3 Criteria for hypoglycaemic events 50
5.2.4.4 Urinary tract infection and genital infections 50
5.2.5 Electrocardiogram 50
5.2.6 Other safety parameters 50
5.2.7 Assessment of adverse events 51
5.2.7.1 Definitions of AEs 51
5.2.7.1.1 Adverse event 51
5.2.7.1.2 Serious adverse event 51
5.2.7.1.3 AEs considered “Always Serious” 51
5.2.7.1.4 Adverse events of special interest 52
5.2.7.1.5 Intensity (severity) of AEs 53
5.2.7.1.6 Causal relationship of AEs 53
5.2.7.2 Adverse event collection and reporting 54
5.2.7.2.1 AE Collection 54
5.2.7.2.2 AE reporting to the sponsor and timelines 54
5.2.7.2.3 Pregnancy 55
5.3 DRUG CONCENTRATION MEASUREMENTS AND PHARMACOKINETICS 55
5.4 ASSESSMENT OF BIOMARKER(S) 55
5.5 BIOBANKING 55

Proprietary confidential information © 2019 Boehringer Ingelheim International GmbH or one or more of its affiliated companies

5.6 OTHER ASSESSMENTS....55
5.6.1 Health Care Resource Utilisation (HCRU)....55
5.7 APPROPRIATENESS OF MEASUREMENTS....56
6. INVESTIGATIONAL PLAN....57
6.1 VISIT SCHEDULE....57
6.2 DETAILS OF TRIAL PROCEDURES AT SELECTED VISITS....57
6.2.1 Screening and run-in period(s)....57
6.2.2 Treatment period(s)....58
6.2.3 Follow-up period and trial completion....59
7. STATISTICAL METHODS AND DETERMINATION OF SAMPLE SIZE....60
7.1 NULL AND ALTERNATIVE HYPOTHESES....60
7.2 PLANNED ANALYSES....60
7.2.1 General considerations....60
7.2.2 Primary endpoint analyses....61
7.2.3 Secondary endpoint analyses....62
7.2.4 Further endpoint analyses....63
7.2.5 Safety analyses....63
7.2.6 Interim Analyses....64
7.3 HANDLING OF MISSING DATA....64
7.4 RANDOMISATION....64
7.5 DETERMINATION OF SAMPLE SIZE....65
8. INFORMED CONSENT, TRIAL RECORDS, DATA PROTECTION, PUBLICATION POLICY, AND ADMINISTRATIVE STRUCTURE....66
8.1 TRIAL APPROVAL, PATIENT INFORMATION, INFORMED CONSENT....66
8.2 DATA QUALITY ASSURANCE....67
8.3 RECORDS....67
8.3.1 Source documents....67
8.3.2 Direct access to source data and documents....68
8.3.3 Storage period of records....68
8.4 EXPEDITED REPORTING OF ADVERSE EVENTS....68
8.5 STATEMENT OF CONFIDENTIALITY AND PATIENT PRIVACY....69
8.6 TRIAL MILESTONES....69
8.7 ADMINISTRATIVE STRUCTURE OF THE TRIAL....69
9. REFERENCES....72
9.1 PUBLISHED REFERENCES....72
9.2 UNPUBLISHED REFERENCES....75
10. APPENDICES....76
10.1 KCCQ (KANSAS CITY CARDIOMYOPATHY QUESTIONNAIRE)....76
10.2 INCLUSION OF ILLITERATE PATIENTS - KCCQ....78
11. DESCRIPTION OF GLOBAL AMENDMENT(S)....79

Proprietary confidential information © 2019 Boehringer Ingelheim International GmbH or one or more of its affiliated companies

ABBREVIATIONS

AE	Adverse Event
AESI	Adverse Event of Special Interest
AF	Atrial Fibrillation
ALCOA	Attributable, Legible, Contemporaneous, Original, Accurate
ALT	Alanine Aminotransferase
AMI	Acute myocardial infarction
ANCOVA	Analysis of Covariance
ARNI	Angiotensin Receptor Neprilysin Inhibitor
AST	Aspartate Aminotransferase
AUC	Area under the Curve
BI	Boehringer Ingelheim
BNP	Brain Natriuretic Peptide
BUN	Blood Urea Nitrogen
CA	Competent Authority
CEC	Clinical Event Committee
CHF	Chronic Heart Failure
CK	Creatine Kinase
CKD-EPIcr	Chronic Kidney Disease Epidemiology Collaboration Equation
COPD	Chronic Obstructive Pulmonary Disease
CRA	Clinical Research Associate
CRF	Case Report Form, paper or electronic (sometimes referred to as “eCRF”)
CRO	Contract Research Organisation
CRT	Cardiac resynchronisation therapy
CT	Computed Tomography
CT Leader	Clinical Trial Leader
CT Manager	Clinical Trial Manager
CTP	Clinical Trial Protocol
CV	Cardiovascular
DILI	Drug Induced Liver Injury
DKA	Diabetic Ketoacidosis
DM	Diabetes Mellitus

Proprietary confidential information © 2019 Boehringer Ingelheim International GmbH or one or more of its affiliated companies

DMC	Data Monitoring Committee
DBP	Diastolic Blood Pressure
EC	Ethics Committee
ECG	Electrocardiogram
eCRF	Electronic Case Report Form
eDC	Electronic Data Capture
EF	Ejection Fraction
EOS	End of Study
eGFR	Estimated Glomerular Filtration Rate
EMPA-REG	Empagliflozin – Reducing Excess Glucose
EoT	End of Treatment
EU	European Union
EudraCT	European Clinical Trials Database
ExCom	Executive Committee
FDA	Food and Drug Administration
FUP	Follow-up
GCP	Good Clinical Practice
GI	Gastrointestinal
GMP	Good Manufacturing Practice
HA	Health Authority
Hb	Haemoglobin
HbA1c	Glycosylated haemoglobin
HCRU	Health Care Resource Utilisation
HDL	High Density Lipoprotein
HF	Heart Failure
HFE	Heart Failure Event
HFmrEF	Heart Failure with mid-range Ejection Fraction
HFpEF	Heart Failure with Preserved Ejection Fraction
HFrEF	Heart Failure with Reduced Ejection Fraction
HHF	Hospitalisation for Heart Failure
HR	Hazard Ratio
i.v.	intravenous
IABP	Intra-Aortic Balloon Pump

Proprietary confidential information © 2019 Boehringer Ingelheim International GmbH or one or more of its affiliated companies

IB	Investigator's Brochure
ICD	Implantable Cardioverter Defibrillator
ICF	Informed Consent Form
ICH	International Council on Harmonisation
IEC	Independent Ethics Committee
IMP	Investigational Medicinal Product
IRB	Institutional Review Board
IRT	Interactive Response Technology
ISF	Investigator Site File
ITT	Intention To Treat
JVP	Jugular Venous Pressure
KCCQ	Kansas City Cardiomyopathy Questionnaire
KCCQ-CSS	Kansas City Cardiomyopathy Questionnaire - Clinical Summary Score
KCCQ-TSS	Kansas City Cardiomyopathy Questionnaire - Total Symptom Score
LDL	Low-Density Lipoprotein
LPLT	Last Patient Last Treatment
LVAD	Left Ventricular Assist Device
LVEF	Left Ventricular Ejection Fraction
MACE	Major Adverse Cardiovascular Events
MedDRA	Medical Dictionary for Drug Regulatory Activities
MMRM	Mixed Model Repeated Measure
MOA	Mode of Action
MRI	Magnetic Resonance Imaging
NCC	National Coordinator Committee
NT-proBNP	N-Terminal Pro-Brain Natriuretic Peptide
NYHA	New York Heart Association
OPU	Operative Unit
PCI	Percutaneous coronary intervention
PPS	Per Protocol Set
PRO	Patient Reported Outcome
PV	Pharmacovigilance
q.d.	quaque die (once a day)
RA	Regulatory Authority

Proprietary confidential information © 2019 Boehringer Ingelheim International GmbH or one or more of its affiliated companies

RBC	Red Blood Cells / Erythrocytes
REP	Residual Effect Period
RS	Randomised Set
SAE	Serious Adverse Event
SBP	Systolic Blood Pressure
SGLT-1	Sodium Glucose Co-Transporter 1
SGLT-2	Sodium Glucose Co-Transporter 2
SMQ	Standardised MedDRA Query
SOP	Standard Operating Procedure
SUSAR	Suspected Unexpected Serious Adverse Reactions
T1DM	Type 1 Diabetes Mellitus
T2DM	Type 2 Diabetes Mellitus
TAVI	Transcatheter Aortic Valve Implantation
TIA	Transient Ischemic Attack
TMF	Trial Master File
TS	Treated Set
TSAP	Trial Statistical Analysis Plan
TSS	Total Symptom Score
ULN	Upper Level of Normal
UTI	Urinary Tract Infections
VT	Ventricular Tachycardia
WBC	White Blood Cells / Leukocytes
WHO	World Health Organisation
WOCBP	Woman of childbearing potential
y-GT	Gamma-Glutamyl Transferase

Proprietary confidential information © 2019 Boehringer Ingelheim International GmbH or one or more of its affiliated companies

1. INTRODUCTION

1.1 MEDICAL BACKGROUND

Heart failure (HF) is one of the most prevalent chronic diseases associated with high mortality and morbidity. Over 22 million people around the world suffer from chronic heart failure (CHF), of which more than 5.5 million live in the US. Patients with HF are at high risk of mortality and morbidity, 50% die within 5 years of their diagnosis of HF and a large number are re-hospitalised for exacerbation of HF symptoms [P16-03952, P16-05920]. Heart failure is the most frequent cause of hospital admission among patients 65 years or older [P16-03952], and there are over 1 million hospitalised patients each year with HF as a primary diagnosis [R19-2918].

The cost burden of treating patients with HF is estimated to be $32 billion a year, and expected to rise to $70 billion a year by 2030. Approximately 80% of the cost is related to hospital admission [R19-2917].

Two main types of HF have been defined mainly based on the left ventricular ejection fraction (LVEF) and also other structural changes in the heart. They consist of heart failure with reduced EF (HFrEF) ≤40% and heart failure with preserved EF (HFpEF) >40%. Relative prevalence of HFrEF among HF patients is approximately 50% [R16-1528]. European Society of Cardiology in their 2016 guideline introduced a third type of heart failure with LVEF ranging between 40-49% named heart failure with mid-range ejection fraction (HFmrEF). The exact characteristics of these patients, response to therapy or prognosis is yet to be determined. In this trial, HF patients with full spectrum of EF are allowed to participate.

Outcomes for patients after a hospitalisation for HF (HHF) remain poor. Among patients with HF, 40-50% demonstrate worsening of HF symptoms, and 30-40% are re-hospitalised within 6 months after discharge from hospital [R19-2916, R19-2915]. After HHF, the one-year mortality rate is high and essentially not different between patients with preserved or reduced LVEF [R16-2217], underscoring a high unmet medical need in this population.

Most hospital admissions for acute decompensation of HF are due to volume overload, evident by weight gain or information gathered from haemodynamic monitors or insertable devices [R19-2914]. In addition, despite apparent symptom control at the time of discharge from hospital, up to 40% of patients still have residual congestion [R19-2913].

The current treatment strategy for patients hospitalised for acute cardiac decompensation is mainly focused around maintaining previously established guideline directed medical therapy, blood pressure stabilisation, optimising volume status, in HFrEF pts initiating life-saving therapies, and initiating beta-blockers if indicated [P13-06404]. Based on available data, in well-selected patients who are haemodynamically stable during hospitalisation for HF, guideline-recommended HF-related medications can be safely initiated [R19-2924], including a recent trial of sacubitril/valsartan, which demonstrated safe and efficacious use in stabilised patients with acute decompensated heart failure with low EF before discharge [R19-2932].

Proprietary confidential information © 2019 Boehringer Ingelheim International GmbH or one or more of its affiliated companies

In-hospital initiation of different therapies is one of the best predictors of long-term adherence to medications [R19-2911, R19-2919, R19-2921] and long-term improvement in prognosis [R19-2924].

Empagliflozin is an orally available inhibitor of the renal dependent sodium glucose cotransporter 2 (SGLT-2), promoting urinary glucose excretion. Empagliflozin is indicated for reduction of blood glucose in patients with Type 2 Diabetes Mellitus (T2DM), and for cardiovascular (CV) death risk reduction in patients with T2DM and established CV disease. Empagliflozin also reduces blood pressure, arterial stiffness and measures of myocardial workload, likely through various mechanisms, as well as improving other CV risk factors (e.g. uric acid, visceral fat mass, albuminuria; [P15-00589, P15-09541].

In 2010, BI initiated the EMPA-REG (Empagliflozin – Reducing Excess Glucose) OUTCOME trial to explore the CV benefit of the drug as well as to establish the CV safety of empagliflozin [P15-09840]. This trial was completed in 2015 and showed that empagliflozin, when given in addition to standard of care treatment in high CV risk patients with T2DM, reduces the risk of 3-point major adverse cardiovascular events (MACE) by 14%, which was mostly driven by a 38% reduction in CV death. Furthermore, this trial demonstrated a relative reduction in the prespecified and adjudicated composite outcome of CV death or HHF by 34% and HHF by 35%.

Consistent with the main results of the EMPA-REG OUTCOME trial, in approximately 10% of the trial population who had investigator-reported heart failure at baseline (with subtype of heart failure not characterized), empagliflozin showed reduction in CV death, HHF, and the composite of HHF or CV death [P16-01253].

In the EMPA-REG OUTCOME trial, empagliflozin was associated with a lower risk of post-acute heart failure rehospitalisation and mortality [P19-01985]. These results indicate that treatment with empagliflozin may play a role in reducing the risk in the post-acute HF period, with a significant early impact on HF hospital readmissions and mortality.

Currently, two large clinical outcome trials are ongoing in patients with chronic HFpEF (EMPEROR-preserved) or HFrEF (EMPEROR-reduced) to evaluate the effect of empagliflozin for the reduction of cardiovascular death and heart failure hospitalisation.

In addition, two clinical trials are ongoing in patients with chronic HFpEF (EMPERIAL-preserved) or HFrEF (EMPERIAL-reduced) to evaluate the effect of empagliflozin on exercise ability using the 6 minute walk test.

It should be noted, that with the exception of smaller investigator-initiated case studies, no prior clinical trial has assessed the effect of empagliflozin in patients hospitalized with HF.

1.2 DRUG PROFILE

Empagliflozin is an orally available, potent, and selective inhibitor of the renal SGLT-2. Its selective inhibition reduces renal reabsorption of sodium and glucose. This leads to both increased urinary sodium and glucose excretion. While urinary sodium excretion returns to

Proprietary confidential information © 2019 Boehringer Ingelheim International GmbH or one or more of its affiliated companies

near normal within a few days of empagliflozin administration, the effect on urinary glucose continues for as long as the medication is used.

Empagliflozin has been developed for the treatment of T2DM, and has received marketing approval in various regions including for example the European Union, Latin American countries, USA and Japan where it is marketed under the brand name Jardiance®.

For a more detailed description of the drug profile please refer to the current Investigator's Brochure (IB) [c01678844] and local prescribing information for empagliflozin.

The Residual Effect Period (REP) of empagliflozin is 7 days. This is the period after the last dose with measurable drug levels and/or pharmacodynamic effects still likely to be present.

1.3 RATIONALE FOR PERFORMING THE TRIAL

Outcomes for patients after a HHF remain poor but in-hospital initiation of different therapies is one of the best predictors of long-term adherence to medications [R19-2911, R19-2919, R19-2920] and long-term improved prognosis [R19-2924], see Section 1.1 for further details.

For empagliflozin, the mode of action (MOA) suggests a potential for improving patients` fluid retention and congestion in the context of cardiac decompensation, which is the rational for performing this study:

- The glucosuria mediated osmotic diuresis, as well as natriuresis, is thought to result in long-lasting haemodynamic changes, less extracellular volume, increase of haemoglobin, shift in fuel supply from glucose to fat oxidation and towards more energy-efficient ketones, possible reduction of vascular wall stress, decrease of ventricular load and improving cardiac function [P15-00589, P15-09541]. While empagliflozin lowers blood pressure, this change is not associated with an increase in heart rate unlike what has been observed for vasodilators [P14-01668].

- The described effects start as early as with the first dose of empagliflozin taken or within the first few weeks of its administration [P16-01830], which would be in line with the observed early effects on reduction in HHF as shown in the EMPA-REG OUTCOME trial. See Figure 1.3: 1.

Proprietary confidential information © 2019 Boehringer Ingelheim International GmbH or one or more of its affiliated companies

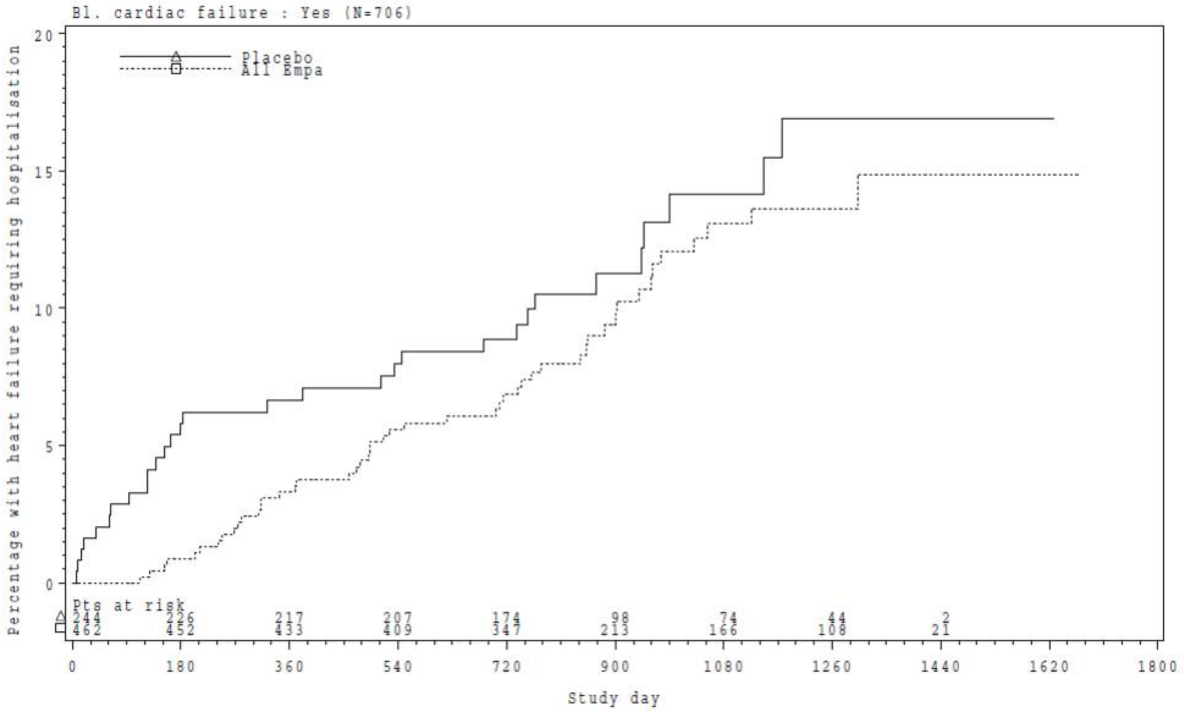

Figure 1.3: 1 Kaplan-Meier estimates of time to first heart failure requiring hospitalisation in patients with baseline cardiac failure [narrow standardized MedDRA query (SMQ)], pooled empagliflozin doses versus placebo-treated set. X-axis: study day [c02695839-01].

- Patients with HFpEF are thought to benefit from combined beneficial effects of empagliflozin (see above). This benefit is expected based on the increased prevalence of obesity, hypertension, and anaemia in patients with HFpEF [R17-1562], as these concomitant conditions may affect heart failure (obesity: increased peripheral resistance; hypertension: increased cardiac workload and oxygen consumption; anaemia: reduced cardiac oxygen supply).

- Although HFpEF and HFrEF are considered as different disease entities, patients with HFrEF are thought to profit from the same combined effects of empagliflozin, with potentially different weighting of the individual beneficial effects.

- Presently, there are two large clinical outcome trials ongoing with empagliflozin, the EMPEROR-reduced (NCT03057977, EudraCT 2017-004073-14) and EMPEROR-preserved trials (NCT03057951, EudraCT 2017-004072-59), investigating the long-term CV mortality and morbidity benefits in patients with chronic HFrEF and HFpEF. Even if these trials are proven positive for long-term clinical morbidity and mortality, it will not be known if in-hospital use of empagliflozin is safe in patients hospitalized for acute heart failure, since hospitalised patients were excluded from the EMPEROR trials. Due to its fast MOA, empagliflozin is expected to potentially alleviate congestive symptoms and residual fluid retention seen in patients with acute heart failure helping to stabilise HF-related symptoms within the first few days and weeks after discharge from hospital.

Proprietary confidential information © 2019 Boehringer Ingelheim International GmbH or one or more of its affiliated companies

As early treatment initiation is important but not covered by the ongoing clinical outcome trials in chronic HF, this trial is performed to assess HF-related outcomes (effect on heart failure events (HFEs), death and Kansas City Cardiomyopathy Questionnaire - Clinical Summary Score (KCCQ-CSS)), and safety and tolerability of early initiation of treatment with empagliflozin in patients hospitalised for acute HF after stabilisation but still in hospital.

1.4 BENEFIT - RISK ASSESSMENT

Empagliflozin is currently indicated for reduction of blood glucose in patients with T2DM, and for CV death risk reduction in patients with T2DM and established CV disease.

The safety profile of empagliflozin has been well established in over 15000 patients with T2DM treated in clinical studies (of which more than 10000 were treated with empagliflozin) with maximum treatment duration of 4 years. Empagliflozin was tested in over 4600 patients with T2DM and high CV risk for median treatment duration of 2.6 years [P15-09840]. In addition, approximately 550 healthy volunteers were exposed to empagliflozin (up to 800 mg single dose and up to 50 mg multiple dosing). Based on the mode of action of empagliflozin, which is independent of insulin and potential concomitant T2DM, it is not expected that the safety profile in patients without T2DM would be different to that in patients with T2DM [P17-04479].

Because of the mode of action, blockade of the SGLT2 transporter by empagliflozin leads to glucosuria in patients with and without diabetes, although with less average daily glucose excretion in non-diabetic patients. Therefore, it is considered likely that the tolerability of empagliflozin in non-diabetic patients will be as favourable as in those with T2DM.

Available data from completed and ongoing trials do not indicate safety concerns for non-diabetic CHF patients, other than those already described for patients with T2DM.

1.4.1 Benefits

A detailed rationale for the use of empagliflozin in HF can be found in Section 1.1 and 1.3.

Patients with HF are thought to benefit from combined effects of empagliflozin such as persistent osmodiuresis, reduction in blood pressure, weight loss, increase in haematocrit, and changes in cardiometabolic parameters as early as the first few weeks of empagliflozin administrations. Due to the combination of positive effects, empagliflozin is expected to address the unmet medical need of reducing congestion in this patient population.

It has been shown in healthy volunteers that dosing with empagliflozin results in glycosuria approximating 2/3 the average glucosuria in patients with T2DM. This is similar to the amount of glucose lost in T2DM subjects with moderate renal impairment [P13-04190]. Because no difference in CV benefit was detected for patients with renal impairment vs. the overall population in the EMPA-REG OUTCOME study, it is hypothesised that the amount of glucosuria is not the main factor for obtaining CV effects with empagliflozin. In the same study, empagliflozin improved the main outcome of CV death and HHF with a similar magnitude in patients with low or high levels of glycosylated haemoglobin (HbA1c) at baseline, and independent of the amount of HbA1c change throughout the trial. In addition, a

Proprietary confidential information © 2019 Boehringer Ingelheim International GmbH or one or more of its affiliated companies

mechanistic study in non-diabetic subjects [P16-01830] provide supporting evidence that the benefit of empagliflozin in treating HF patients should also be expected in patients without diabetes. Taking all the data into consideration, the risk reduction for HF outcome appears to be independent of the glycaemic status at baseline or the degree of glycaemic control throughout the trial or the amount of glucosuria, which suggests the benefits could be achieved in both diabetic and non-diabetic populations. [P15-09840, P18-10152]

1.4.2 Risks

Table 1.4.2: 1: Known and potential risks of clinical relevance for the study population

Known or potential risks of clinical relevance for this trial	Summary of data, rationale for the risk	Mitigation strategy
Investigational Medicinal Product: Empagliflozin		
Hypoglycaemia	The risk of hypoglycaemia was increased only when empagliflozin was used concomitantly with insulin and/or sulfonylurea. The risk of hypoglycaemia of patients without diabetes mellitus (DM) is considered very low. In a mechanistic study [c11963611-01] subjects without DM had an increase of endogenous glucose production in response to glycosuria due to empagliflozin.	Patients with type 1 diabetes mellitus (T1DM) are excluded from the study.<br>Guidance for the investigator provided in the IB.
Volume depletion	Empagliflozin may selectively reduce interstitial volume with minimal change in intravascular volume.<br>Polyuria and consequent dehydration and hypotension were identified as risks in patients treated with empagliflozin, especially patients with known CV disease, history of hypotension, taking diuretics, other antihypertensive drugs, or elderly patients aged 75 years and older. [P19-02151]	Hospitalised patients will be randomised after diuretics stabilisation.<br>Guidance for the investigator provided in the IB.<br>Information and recommendations for the patients provided in the Informed Consent Form (ICF).
Diabetic ketoacidosis (DKA)	Rare cases of DKA, including fatal cases, were reported in patients treated with SGLT2-inhibitors. In patients treated with SGLT2-inhibitors DKA may occur with lower than usual glucose values. The risk of DKA is increased in patients	Patients with T1DM are excluded from the study.<br>Guidance for the investigator provided in the IB. Training will be provided. Information and recommendations for the patients provided in the ICF.

Proprietary confidential information © 2019 Boehringer Ingelheim International GmbH or one or more of its affiliated companies

Known or potential risks of clinical relevance for this trial	Summary of data, rationale for the risk	Mitigation strategy
Investigational Medicinal Product: Empagliflozin		
	with lower than needed insulin intake, T1DM, low carbohydrate intake, acute illness, major trauma, operation, severe dehydration or alcohol use.	DKA is an adverse even of special interest (AESI). Cases reported as DKA or metabolic acidosis are adjudicated. (see Sections 4.2.1 and 8.7; details are also described in the adjudication charter)
Complicated urinary tract infections (UTI)	Cases of complicated UTI, including pyelonephritis and urosepsis were reported in patients treated with empagliflozin.	Guidance for the investigator provided in the IB. Information and recommendations for the patients provided in the ICF.
Necrotizing fasciitis of perineum (Fournier's gangrene)	Rare cases of Fournier's gangrene, including fatal cases, were reported in patients treated with SGLT2-inhibitors.	Guidance for the investigator provided in the IB. Information and recommendations for the patients provided in the ICF.
Hypersensitivity	The risks of allergic skin reactions (e.g. rash, urticaria) and angioedema were identified for empagliflozin based on post-marketing experience. As with all drugs, the risk of severe and unexpected allergic reactions cannot be excluded.	Patients with hypersensitivity to empagliflozin are excluded from trial participation.
Drug-induced liver injury (DILI)	No risk of DILI was identified for empagliflozin. However, DILI generally can be severe and lead to fatal outcome or need of liver transplant. Therefore, careful monitoring and assessment of patients for potential DILI is needed.	Parameters for potential liver injury are included in the safety laboratory. Cases of liver impairment are defined as an AESI. Severe cases of liver impairment are adjudicated. (see Sections 5.2.4, 5.2.7 and 8.7; details are also described in the adjudication charter)

Proprietary confidential information © 2019 Boehringer Ingelheim International GmbH or one or more of its affiliated companies

Known or potential risks of clinical relevance for this trial	Summary of data, rationale for the risk	Mitigation strategy
Investigational Medicinal Product: Empagliflozin		
Renal safety	In clinical trials in patients with DM, the incidence of renal impairment was similar to placebo. An initial decrease of estimated glomerular filtration rate (eGFR) was seen in patients treated with empagliflozin, which improved during continuous treatment or discontinuation of empagliflozin.<br>Cases of renal impairment, including requiring dialysis, were reported in patients using SGLT2-inhibitors. Due to the renal mode of action and risk of volume depletion, the renal safety should be monitored.	Parameters for potential renal impairment are included in the safety laboratory.<br>Cases of renal impairment are defined as an AESI (see Sections 5.2.4 and 5.2.7.1.4).
Women of childbearing potential (WOCBP)	The safety of empagliflozin in pregnant women was not established.	In accordance with international regulatory guidelines, WOCBP are excluded from trial participation unless they agree to use highly effective contraceptive method.<br>All WOCBP undergo pregnancy testing before being randomised.

<u>Known and potential risks in healthy volunteers and patients with T2DM:</u>

For the main risks, please refer to Table 1.4.2: 1

In clinical studies, empagliflozin was well tolerated in both healthy volunteers and patients with T2DM including patients with high CV risk. The frequency of overall adverse events (AEs), AEs leading to discontinuation and serious adverse events (SAEs) were comparable to placebo [c01678844].

In general there was a small increase in frequency of urinary tract infection (UTI) compared to placebo. There was an increase in frequency of genital infections with the use of empagliflozin. There was a small increase in total cholesterol, low-density lipoprotein (LDL) cholesterol and high-density lipoprotein (HDL) cholesterol and no significant changes in triglycerides. No clinically relevant changes in electrolytes were observed with empagliflozin [c01678844].

Proprietary confidential information © 2019 Boehringer Ingelheim International GmbH or one or more of its affiliated companies

Known and potential risks in patients with CHF and without T2DM:

In the empagliflozin HF program including two ongoing large EMPEROR outcome trials, and two EMPERIAL exercise capacity trials (with more than 7500 patients being followed-up as of May 2019), the frequencies of overall adverse events (AE), severe AE, serious AE, and AE leading to treatment discontinuation reported in patients without DM were similar or lower than the ones reported in patients with DM and consistent with the known safety reporting described in the Investigator Brochure. The frequency of AE of hypoglycaemia in patients without DM was lower than in patients with DM.

As of July 2019 based on blinded data review there has been no severe hypoglycaemia (i.e. requiring assistance) or serious hypoglycaemic events and no case of ketoacidosis reported in patients without DM and no other safety concerns were raised in this population.

Adverse events related to volume depletion, hypotension, urinary tract infections and genital infections in patients without DM were reported with similar frequency as in patients with DM.

Many patients with HF have renal impairment, and to ensure that the trial results reflect this population, patients with estimated glomerular filtration rate (eGFR) ≥ 20 ml/min/1.73m2 can be included. In the EMPA-REG Outcome trial, the cardiovascular benefits for empagliflozin were not driven by its pharmacological effect of lowering blood glucose and were consistently noted in patients with different degrees of renal impairment, including 540 patients with eGFR between > 30 and < 45 ml/min/1.73m^2. In previous trials in patients with T2DM, the safety profile in moderate and severe renal impairment was comparable to the overall trial population [P17-10453].

In the ongoing EMPEROR trials there have been more than 1300 patients with eGFR between ≥ 30 and < 45 ml/min/1.73m^2 with no safety issue identified to date. As part of the periodic Data Monitoring Committee (DMC) review, the safety in more than 400 patients with eGFR less than 30 ml/min/1.73m^2 has also been specifically evaluated and as of July 2019 there have been no safety issues.

In addition, a dapagliflozin outcome trial in over 4700 patients with HFrEF showed a significant risk reduction in the composite primary outcome of CV death and HHF with a similar safety profile compared to placebo. In this trial, patients without DM (nearly 60%) also showed a positive trend towards improvement of CV death and HHF and a safety profile similar to patients with DM. There was no increased risk of hypoglycaemia or ketoacidosis in patients without DM [R19-3125].

Renal safety will be closely monitored throughout the trial (see Section 5.2.4 and Flow Chart).

In addition an independent Data Monitoring Committee (DMC) is implemented who is following up on patients` safety in an unblinded manner. Please refer to Section 8.7 for details.

Proprietary confidential information © 2019 Boehringer Ingelheim International GmbH or one or more of its affiliated companies

1.4.3 Discussion

The overall tolerability and safety profile, as outlined in this Section 1.4 and the evidence as summarized in the current Investigator's Brochure (IB), supports chronic safe administration of empagliflozin 10 mg in human studies, specifically in the heart failure population intended for inclusion into this trial.

Patients will be treated according to standard of care and trial medication will be given in addition to standard therapy. It should also be noted that empagliflozin has not been approved for the treatment of HF. Given the short duration of the trial, the lack of an indication of empagliflozin for the treatment of heart failure and the gap in knowledge in heart failure patients in the acute setting, it is justified to include patients with type 2 diabetes mellitus in this trial, who may receive other antidiabetic therapies for controlling their glucose levels. Therefore, the placebo controlled trial design does not constitute a risk for the population in this study.

Due to the combination of positive effects, empagliflozin is expected to address the unmet medical need of reducing congestion in the patient population under investigation in this trial.

Overall the potential benefits, coupled with an acceptable safety profile support the initiation of the trial.

Proprietary confidential information © 2019 Boehringer Ingelheim International GmbH or one or more of its affiliated companies

2. TRIAL OBJECTIVES AND ENDPOINTS

2.1 MAIN OBJECTIVES, PRIMARY AND SECONDARY ENDPOINTS

2.1.1 Main objectives

The main objective of this study is to assess whether in-hospital administration of empagliflozin results in improvements in HF-related clinical events and patient-reported outcomes (death, HFE and KCCQ-CSS as a measure of health status (symptoms and physical limitations)) in patients hospitalised for acute heart failure (de novo or decompensated chronic HF) and after initial stabilisation.

Secondary objectives are to further assess whether it is safe to start empagliflozin in patients admitted to hospital in this setting. See Section 2.1.3.

This trial is part of an investigational clinical trial program of empagliflozin in patients with HF.

2.1.2 Primary endpoint(s)

The primary endpoint is the net clinical benefit, a composite of death, number of heart failure events (HFE) (including hospitalisations for heart failure (HHFs), urgent heart failure visits and unplanned outpatient visits), time to first HFE and change from baseline in KCCQ-CSS after 90 days of treatment.

2.1.3 Secondary endpoint(s)

Secondary endpoints to assess safety and efficacy in this setting are:

- Proportion of patients with an improvement in KCCQ-CSS of ≥ 10 points after 90 days of treatment.
- Change from baseline in KCCQ-CSS after 90 days of treatment.
- Change from baseline in log-transformed N-Terminal Pro-Brain Natriuretic Peptide (NT-proBNP) level over 30 days of treatment (area under the curve (AUC)).
- Days alive and out of hospital from study drug initiation until 30 days after initial hospital discharge.
- Days alive and out of hospital from study drug initiation until 90 days after randomisation.
- Time to first occurrence of CV death or HFE until end of trial visit.
- Occurrence of HHF until 30 days after initial hospital discharge.
- Occurrence of chronic dialysis or renal transplant or sustained[1] reduction of ≥40% eGFR Chronic Kidney Disease Epidemiology Collaboration Equation ((CKD-EPI)cr), or

[1] An eGFR (CKD-EPI)cr reduction is considered sustained, if it is determined by two or more consecutive post-baseline central laboratory measurements separated by at least 30 days (first to last of the consecutive eGFR values). Chronic dialysis is

Proprietary confidential information © 2019 Boehringer Ingelheim International GmbH or one or more of its affiliated companies

  - sustained eGFR (CKD-EPI)cr <15 mL/min/1.73 m^2 for patients with baseline eGFR ≥30 mL/min/1.73 m^2
  - sustained eGFR (CKD-EPI)cr <10 mL/min/1.73 m^2 for patients with baseline eGFR <30 mL/min/1.73 m^2
- Diuretic effect as assessed by weight loss per mean daily loop diuretic dose after 15 days of treatment.
- Diuretic effect as assessed by weight loss per mean daily loop diuretic dose after 30 days of treatment.

2.2 FURTHER OBJECTIVES AND FURTHER ENDPOINTS

2.2.1 Further objectives

Further objectives are to evaluate additional and complimentary details on efficacy and safety.

2.2.2 Further endpoints

- Proportion of patients with an improvement in KCCQ-CSS of ≥ 5 points after 30 days of treatment.
- Proportion of patients with an improvement in KCCQ-CSS of ≥ 10 points after 30 days of treatment.
- Proportion of patients with an improvement in KCCQ-CSS of ≥ 20 points after 30 days of treatment.
- Proportion of patients with an improvement in KCCQ-CSS of ≥ 5 points after 90 days of treatment.
- Proportion of patients with an improvement in KCCQ-CSS of ≥ 20 points after 90 days of treatment.
- Change from baseline in KCCQ CSS after 15 days of treatment.
- Change from baseline in KCCQ-CSS after 30 days of treatment.
- Mean of changes from baseline in KCCQ-CSS at Days 15, 30 and 90.
- Change from baseline in KCCQ overall summary score, Total Symptom Score (TSS), and individual domains after 15, 30 and 90 days of treatment.
- Change from baseline in log-transformed NT-proBNP level over 15 days of treatment (AUC).
- Change from baseline in log-transformed NT-proBNP level over 90 days of treatment (AUC).
- Proportion of patients with a reduction of NT-proBNP ≥ 30% after 90 days of treatment.
- Time to occurrence of CV death until end of trial visit.
- Time to first occurrence of HFE until end of trial visit.
- Time to occurrence of all-cause mortality until end of trial visit.
- Time to first occurrence of HFE until end of trial visit.
- Occurrence of CV death, HFE until 30 days after initial hospital discharge.

---

regarded as chronic if the frequency of dialysis is twice or more per week for at least 90 days.

Proprietary confidential information © 2019 Boehringer Ingelheim International GmbH or one or more of its affiliated companies

- Occurrence of HFE until 30 days after randomisation.
- Occurrence of all-cause mortality until 30 days after initial hospital discharge.
- Occurrence of acute renal failure (based on narrow SMQ).
- Change from baseline in eGFR (CKD-EPI)cr after 15, 30 and 90 days of treatment.
- Occurrence of dialysis or renal transplant or sustained[2] reduction of ≥40% eGFR (CKD-EPI)cr, or
    - sustained eGFR (CKD-EPI)cr <15 mL/min/1.73 m^2 for patients with baseline eGFR ≥30 mL/min/1.73 m^2.
    - sustained eGFR (CKD-EPI)cr <10 mL/min/1.73 m^2 for patients with baseline eGFR <30 mL/min/1.73 m^2.
- Occurrence of first acute kidney injury (based on preferred term).
- Occurrence of symptomatic hypotension until 90 days after randomisation.
- Percentage of patients on medication (adherence to medication) over 90 days.
- Change from baseline in body weight after 15, 30 and 90 days of treatment.
- Diuretic effect as assessed by weight loss per mean daily loop diuretic dose after 90 days of treatment.
- Change from baseline in haematocrit after 15, 30 and 90 days of treatment.
- Change from baseline in New York Heart Association (NYHA) class after 90 days of treatment.
- Occurrence of hypoglycaemic events.
- Occurrence of volume depletion.
- Change from baseline in Systolic Blood Pressure (SBP) after 15, 30 and 90 days of treatment.
- Change from baseline in Diastolic Blood Pressure (DBP) after 15, 30 and 90 days of treatment.
- Change from baseline in Clinical Congestion Score after 15 and 90 days of treatment.

[2] An eGFR (CKD-EPI)cr reduction is considered sustained, if it is determined by two or more consecutive post-baseline central laboratory measurements separated by at least 14 days (first to last of the consecutive eGFR values).

Proprietary confidential information © 2019 Boehringer Ingelheim International GmbH or one or more of its affiliated companies

3. DESCRIPTION OF DESIGN AND TRIAL POPULATION

3.1 OVERALL TRIAL DESIGN

This is a randomised, double-blind, parallel-group, placebo controlled, multinational and multicentre study.

A total of approximately 500 male and female patients admitted to the hospital for acute HF will (de novo or decompensated chronic HF) be randomised (1:1) in the study in about 13 countries.
The pre-screening period will start when patients are admitted. Screening (Visit 1) and randomisation (Visit 2a) can happen on the same day. Randomisation should occur after at least 24 hours and no later than 5 days after admission and before discharge when patients fulfil criteria to indicate clinical stability including a stable dose of diuretics. Visit 2 consists of two further visits (Visit 2b and 2c) that will be performed only if the patient is still in hospital. All patients will be treated on top of standard of care. Patients will receive the study drug for 90 days. A follow-up phone call will be performed 7 days after the End of Treatment Visit.

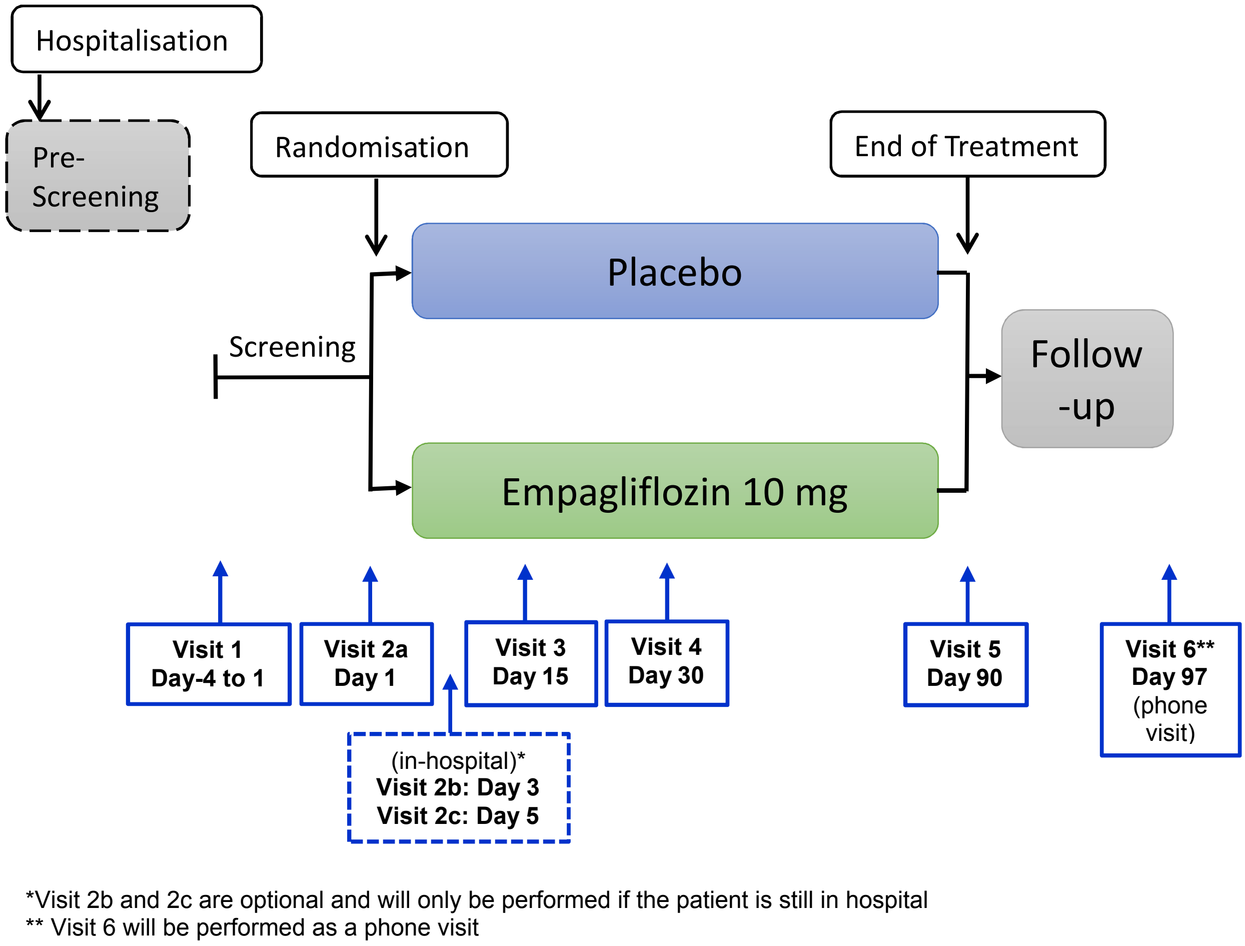

Figure 3.1: 1 Trial design

Proprietary confidential information © 2019 Boehringer Ingelheim International GmbH or one or more of its affiliated companies

The primary endpoint will be assessed at Visit 5.

Patients who prematurely stop medication should have an End of Treatment Visit but be followed up until their planned Visit 5 (Day 90) according to the visit schedule.

3.2 DISCUSSION OF TRIAL DESIGN, INCLUDING THE CHOICE OF CONTROL GROUP(S)

Hospitalisation for HF identifies patients at increased risk of death and re-hospitalisation following discharge. In the EMPEROR trials (see Section 1.1) patients hospitalised for HF were excluded.
Since the long-term mortality and morbidity of empagliflozin in patient with chronic HF will be assessed in EMPEROR trials, the main objective of this study is to assess whether in-hospital administration of empagliflozin leads to an improvement in the clinical benefit (a composite of death, number of HFEs, time to first HFE and change from baseline in KCCQ-CSS) in patients hospitalised for acute heart failure (de novo or decompensated chronic) and after initial stabilisation. Secondary objectives are to further assess whether it is safe to start empagliflozin in patients already admitted to hospital in this setting.

Choice of endpoint:

Patient-reported outcomes (PRO) can provide information on a range of patients' health status. Improving patient's symptoms, physical limitations and quality of life is one of the key goals of managing HF, and is endorsed by practice guidelines and the regulators. Health authorities, including US FDA, and major payers such as the Center for Medicare and Medicaid are increasingly interested in PROs [R19-3040, R19-3126]. To better evaluate the effect of empagliflozin on improving health status (symptoms, and physical limitations), the Kansas City Cardiomyopathy Questionnaire (KCCQ) will be used in this trial. The KCCQ is one of the most validated, clinically responsive and widely used HF PRO instruments [R17-2687].

In addition, in DAPA-HF, an outcome trial in over 4700 patients with HFrEF, dapagliflozin showed significant improvement in change in Kansas City Cardiomyopathy Questionnaire - Total Symptom Score (KCCQ-TSS) from baseline at 8 months. A similar improvement in KCCQ overall summary score was also detected in DEFINE-HF, a study of approximately 260 patients evaluating the effect of dapagliflozin compared to placebo on biomarkers, symptoms, and functional status in patients with HFrEF (both with and without DM) [R19-3125, R19-3124]. KCCQ is a self-administered questionnaire designed and validated to evaluate physical limitations, symptoms (frequency, severity, and changes over time), social limitations, self-efficacy, and quality of life in patients with HF (refer to Appendix 10.1).

In this trial, the KCCQ-CSS (which includes symptom burden and physical limitations domains) will be assessed as part of a composite clinical benefit endpoint including mortality and HF events (see Section 7.2.2).

General safety including parameters relevant to the empagliflozin MOA i.e. hypotension and renal function will be the focus of the safety assessment in this trial. Although it is likely that clinical events like death and hospitalisations will occur in this trial, this study is limited in its

Proprietary confidential information © 2019 Boehringer Ingelheim International GmbH or one or more of its affiliated companies

statistical power to conclusively assess the effect on morbidity and mortality outcomes with the planned sample size.

Control group:

Due to its mode of action, empagliflozin has the potential to be efficacious in treating patients' congestion with HF. However, the effect of empagliflozin in patients with acute heart failure (de novo or decompensated chronic) after stabilisation in hospital has not yet been systematically assessed in a randomised clinical trial. Currently, two large clinical outcome trials are ongoing in patients with HFpEF (EMPEROR-preserved) or HFrEF (EMPEROR-reduced) to evaluate empagliflozin for the reduction of cardiovascular death and heart failure hospitalisation in patients outside the hospital setting.

The placebo-controlled design is considered ethically acceptable on the basis of appropriate criteria for patient selection and discontinuation, the ability to change background therapy to maintain, or obtain sufficient levels of haemodynamic and glycaemic control (in patients with T2DM) as defined in relevant local and regional guidelines for optimised standard of care.

Patients should be receiving appropriate care as defined by their physician or practitioner for all cardiovascular conditions according to the prevailing guidelines. In HFrEF patients, this includes, but is not limited to (if indicated and not contraindicated) acetylsalicylic acid, statins, a diuretic, an inhibitor of the renin-angiotensin system with or without neprilysin inhibitor, a beta-blocker and a mineralocorticoid receptor antagonist, each to be given at clinically appropriate doses, and the use of implantable devices like pacemakers or implantable cardioverter defibrillators (ICDs, or cardiac resynchronisation therapies (CRTs)). This therapy should be selected in the context of local or regional guidelines for primary or secondary CV disease prevention.

Duration:

Due to the potential early effect of empagliflozin (see Section 1.1 for mode of action and EMPA-REG-OUTCOME results) empagliflozin is expected to exert its effect within a few days to weeks of administration.

The primary endpoint is net clinical benefit, a composite of death, number of HFEs, time to first HFE and change from baseline in KCCQ-CSS at Day 90.

Thus, a 90 day time point is considered suitable for the assessment of the primary endpoints and evaluation of empagliflozin safety.

3.3 SELECTION OF TRIAL POPULATION

A total number of approximately 500 patients are planned to be randomised in about 13 countries and approximately 125 sites.

Proprietary confidential information © 2019 Boehringer Ingelheim International GmbH or one or more of its affiliated companies

The study is planned to be conducted in the US/North America and in Europe and may be expanded to additional countries (e.g. in Asia, South America or Eastern Europe) and more sites based on patient availability.

Screening of patients for this trial is competitive, i.e. screening for the trial will stop at all sites at the same time once a sufficient number of patients has been screened. Investigators will be notified about screening completion and will then not be allowed to screen additional patients for this trial.

A log of all patients enrolled into the trial (i.e. who have signed informed consent) will be maintained in the Investigator Site File (ISF) irrespective of whether they have been treated with investigational drug or not.
If a patient is enrolled in error (does not meet all inclusion criteria or meets one or more exclusion criteria on the day of enrolment), the sponsor should be contacted immediately.

3.3.1 Main diagnosis for trial entry

Patients admitted to the hospital for treatment of acute HF (de novo or decompensated chronic HF). Patients should be randomised after at least 24 hours and no later than 5 days after hospital admission. Randomisation should occur as soon as the patient is stabilised (see inclusion criterion 7).

Please refer to Section 8.3.1 (Source Documents) for the documentation requirements pertaining to the in- and exclusion criteria.

3.3.2 Inclusion criteria

1. Of full age of consent (according to local legislation, at least ≥ 18 years) at screening.

2. Signed and dated written informed consent in accordance with ICH-GCP and local legislation prior to admission to the trial.

3. Male or female patients. Women of childbearing potential (WOCBP)[3] must be ready and able to use highly effective methods of birth control per ICH M3 (R2) that result in a low failure rate of less than 1% per year when used consistently and correctly. A list of contraception methods meeting these criteria is provided in the patient information.

4. Currently hospitalised for the primary diagnosis of acute heart failure (de novo or decompensated chronic HF), regardless of EF. Patients with a diagnosis of hospitalised heart failure must have the following HF signs and symptoms at the time of hospital admission:

---

[3] A woman is considered of childbearing potential, i.e. fertile, following menarche and until becoming post-menopausal unless permanently sterile.
Permanent sterilisation methods include hysterectomy, bilateral salpingectomy and bilateral oophorectomy.
Tubal ligation is NOT a method of permanent sterilisation. A woman who underwent tubal ligation is still considered as WOCBP. However tubal ligation is considered as a method of highly effective birth control.
A postmenopausal state is defined as no menses for 12 months without an alternative medical cause.

Proprietary confidential information © 2019 Boehringer Ingelheim International GmbH or one or more of its affiliated companies

a. Persistent dyspnoea at rest or with minimal exertion, and,
b. Signs of fluid overload, at least two of the following must apply:
    i. Congestion on chest X-ray,
    ii. Rales on chest auscultation,
    iii. Clinical relevant oedema (e.g. ≥1+ on a 0 to 3+ scale), indicating indentation of skin with mild digital pressure that requires 10 or more seconds to resolve in any dependent area including extremities or sacral region,
    iv. Elevated jugular venous pressure (JVP).

5. Evidence of LVEF (either reduced or preserved EF, preferably quantitative) as per local reading (by e.g. echocardiography, radionuclide ventriculography, invasive angiography, MRI or CT) preferably measured during current hospitalisation. A historical LVEF assessment may be used if there is no in-hospital measurement and if it was measured within 12 months prior to randomisation provided there was no major intervening disease affecting EF like MI. The LVEF should preferably be documented in a local report prior to randomisation. If no such report is available, LVEF, valve status and relevant pathologic cardiac findings from echocardiography or other local readings should be mentioned in the source documents.

6. Patients must be randomised after at least 24 hours and no later than 5 days after admission, as early as possible after stabilisation and while they are still hospitalised.

7. Patients must fulfil the following stabilisation criteria (while in the hospital):
    - Systolic BP ≥100mm Hg and no symptoms of hypotension in the preceding 6 hours,
    - no increase in i.v. diuretic dose for 6 hours prior to randomisation,
    - no i.v. vasodilators including nitrates within the last 6 hours prior to randomisation
    - no i.v. inotropic drugs for 24 hours prior to randomisation.

8. Elevated NT-proBNP ≥ 1600 pg/mL or BNP ≥400 pg/mL according to the local lab for patients without atrial fibrillation (AF); or elevated NT-proBNP ≥ 2400 pg/mL or BNP ≥600 pg/mL for patients with AF, measured during the current hospitalisation or in the 72 hours prior to hospital admission. For patients treated with an angiotensin receptor neprilysin inhibitor (ARNI) in the previous 4 weeks prior to randomisation, only NT-proBNP values should be used.

9. HF episode leading to hospitalisation must have been treated with a minimum dose of 40 mg of i.v. furosemide (or equivalent i.v. loop diuretic defined as 20 mg of torasemide or 1 mg of bumetanide).

3.3.3 Exclusion criteria

1. Cardiogenic shock.

Proprietary confidential information © 2019 Boehringer Ingelheim International GmbH or one or more of its affiliated companies

2. Current hospitalisation for acute heart failure primarily triggered by pulmonary embolism, cerebrovascular accident, or acute myocardial infarction (AMI)[4] diagnosed using electrocardiogram (ECG), and/or cardiac imaging and/or coronary angiography.

3. Current hospitalisation for acute heart failure not caused primarily by intravascular volume overload; for example triggered by significant arrhythmia (e.g., sustained ventricular tachycardia, or atrial fibrillation/flutter with sustained ventricular response >130 beats per minute, or bradycardia with sustained ventricular arrhythmia <45 beats per minute), infection/sepsis, severe anaemia, or acute exacerbation of chronic obstructive pulmonary disease (COPD).

4. Below interventions in the past 30 days prior to randomisation or planned during the study:

   - Major cardiac surgery (for example coronary artery bypass graft or valve replacement), or Transcatheter Aortic Valve Implantation (TAVI), or percutaneous coronary intervention (PCI), or Mitraclip
   - All other surgeries that are considered major according to investigator judgement,
   - Implantation of a cardiac resynchronisation therapy device (CRT) (pacemakers or implantable cardioverter defibrillators (ICD) without resynchonization function are allowed),
   - cardiac mechanical support implantation
   - Carotid artery disease revascularisation (stent or surgery)

5. Acute coronary syndromes / myocardial infarction (increase in cardiac enzymes in combination with symptoms of ischaemia or newly developed ischaemic ECG changes), stroke or TIA in the past 90 days prior to randomisation.

6. Symptomatic ventricular tachycardia (VT) with syncope in patients without an ICD in the past 90 days prior to randomisation.

7. Current use or prior use of a SGLT-2 inhibitor or combined SGLT-1 and 2 inhibitor in the past 90 days prior to randomisation. Discontinuation of a SGLT-2 inhibitor or combined SGLT-1 and 2 inhibitor for the purposes of study enrolment is not permitted.

8. Heart transplant recipient, or listed for heart transplant with expectation to receive a transplant during the course of this trial (according to investigator judgement), or planned for palliative care for HF, or currently using or plan for left ventricular assist device (LVAD) or intra-aortic balloon pump (IABP) or any other type of mechanical circulatory support, or patients on mechanical ventilation, or patients with planned inotropic support in an out-patient setting.

9. Haemodynamically significant (severe) uncorrected primary cardiac valvular disease planned for surgery or intervention during the course of the study (note: secondary mitral

[4] *Please note: Troponin elevations are frequently seen in patients with acute heart failure and therefore are not sufficient for a diagnosis of AMI. In order to exclude an AMI, concomitant objective evidence of AMI based on typical symptoms, ECG, and/or cardiac imaging and/or coronary angiography should be available.*

Proprietary confidential information © 2019 Boehringer Ingelheim International GmbH or one or more of its affiliated companies

regurgitation or tricuspid regurgitation due to dilated cardiomyopathy is not excluded unless planned for surgery or intervention during the course of the study).

10. Cardiomyopathy due to infiltrative diseases (e.g. amyloidosis), accumulation diseases (e.g. haemochromatosis, Fabry disease), muscular dystrophies, cardiomyopathy with reversible causes (e.g. stress cardiomyopathy), hypertrophic obstructive cardiomyopathy, complex (according to investigator`s judgement) congenital heart disease, or known pericardial constriction.

11. Heart failure caused by peripartum cardiomyopathy or Tako-Tsubo cardiomyopathy diagnosed within the past 6 months, or active myocarditis, or uncorrected thyroid disease, or other acute structural heart disease as cause for acute cardiac decompensation (e.g. acute mitral cord rupture as cause for hospital admission for heart failure).

12. Symptomatic bradycardia or third degree heart block without a pacemaker.

13. SBP $\geq$ 180 mmHg at randomisation (confirmed with second measurement after 5 minutes).

14. Atrial fibrillation or atrial flutter with a documented resting heart rate > 110 bpm at Visit 1.

15. Impaired renal function, defined as eGFR < 20 mL/min/1.73 m^2 as measured during hospitalisation (latest local lab measurement before randomisation) or requiring dialysis.

16. Type 1 Diabetes Mellitus (T1DM).

17. Indication of liver disease, defined by serum levels of either alanine aminotransferase (ALT) (SGPT), aspartate aminotransferase (AST) (SGOT), or alkaline phosphatase above 3 x upper limit of normal (ULN) as determined at Visit 1 or history of cirrhosis with evidence of portal hypertension such as varices.

18. Haemoglobin < 9 g/dl at Visit 1.

19. History of ketoacidosis, including DKA.

20. Gastrointestinal (GI) surgery or GI disorder that could interfere with trial medication absorption in the investigator’s opinion.

21. Any documented active or suspected malignancy or history of malignancy within 2 years prior to screening, except appropriately treated basal cell carcinoma of the skin or in situ carcinoma of uterine cervix or low risk prostate cancer (biopsy Gleason score of $\leq$ 6 and clinical stage T1c or T2a).

22. Presence of any disease other than heart failure with life expectancy less than 1 year.

23. Decision for palliative care in HF (informed patient decision to adhere to limited heart failure treatments only).

Proprietary confidential information © 2019 Boehringer Ingelheim International GmbH or one or more of its affiliated companies

24. Patients who must or wish to continue the intake of restricted medications (see Section 4.2.2) or any drug considered likely to interfere with the safe conduct of the trial

25. Current or previous randomisation in another empagliflozin heart failure trial (i.e. studies 1245.110, 1245.121, 1245-0167, 1245-0168) or currently enrolled in another investigational device or drug trial, or less than 30 days since ending another investigational device or drug trial(s), or receiving other investigational treatment(s). Patients participating in a purely observational trial will not be excluded.

26. Known allergy or hypersensitivity to empagliflozin or any excipient of the Investigational Medicinal Product (IMP), or other SGLT-2 inhibitors.

27. Chronic alcohol or drug abuse or any condition that, in the investigator's opinion, makes them an unreliable trial patient or unlikely to complete the trial.

28. Women who are pregnant, nursing, or who plan to become pregnant while in the trial.

29. Any other clinical condition that would jeopardise patients safety while participating in this trial, or may prevent the patient from adhering to the trial protocol.

3.3.4 Withdrawal of patients from treatment or assessments

Patients may discontinue trial treatment or withdraw consent to trial participation as a whole ("withdrawal of consent") with very different implications; please see Sections 3.3.4.1 and 3.3.4.2 below.

An excessive withdrawal rate can have a severe negative impact on the scientific value of the trial. The "Intention To Treat (ITT)" analysis requires that all randomised patients be followed until trial end even if the trial medication was temporarily interrupted, discontinued or never started.

Every effort should be made to keep the patients in the trial: if possible on treatment, or at least to collect important trial data.

Measures to control the withdrawal rate include careful patient selection, appropriate explanation of the trial requirements and procedures prior to trial enrolment, as well as the explanation of the consequences of withdrawal.

The decision to discontinue trial treatment or withdraw consent to trial participation and the reason must be documented in the patient files and Case Report Form (CRF). If applicable, consider the requirements for Adverse Event collection reporting (please see Sections 5.2.7.2.1 and 5.2.7.2).

3.3.4.1 Discontinuation of trial treatment

An individual patient will discontinue trial treatment if:

- The patient wants to discontinue trial treatment, without the need to justify the decision.

Proprietary confidential information © 2019 Boehringer Ingelheim International GmbH or one or more of its affiliated companies

- The patient has repeatedly shown to be non-compliant with important trial procedures and, in the opinion of both, the investigator and sponsor representative, is not willing or able to adhere to the trial requirements in the future.
- The patient needs to take concomitant medication that interferes with the investigational medicinal product (see Section 4.2.2.1) for restricted medication
- The patient can no longer receive trial treatment for medical reasons (such as surgery, adverse events, other diseases, or pregnancy).In case of a temporary reason for treatment

discontinuation, trial treatment should be restarted if medically justified, please see Section 4.1.4.

Given the patient's agreement, the patient will undergo the procedures for the Early Discontinuation Visit as soon as possible after treatment discontinuation and the Follow-up Visit as outlined in the Flow Chart and Section 6.2.3.
In addition, if the patient discontinues early, every effort should be made for the patient to attend the regularly scheduled study visits and have all study procedures performed except those pertaining to drug intake. However if this is not possible, then the remaining visits should be conducted by phone. In the rare case that even this is not possible, at least vital status and information about HFEs should be retrieved and documented at the day of the scheduled Visit 5 (Day 90).

If a patient becomes pregnant during the trial, the trial medication will be stopped, the patient will be followed up during the trial and until birth or termination of the pregnancy (see further details in Section 5.2.7.2).

If new efficacy/safety information becomes available, Boehringer Ingelheim will review the benefit-risk-assessment and, if needed, pause or discontinue the trial treatment for all patients or take any other appropriate action to guarantee the safety of the trial patients.

3.3.4.2 Withdrawal of consent to trial participation

Patients may withdraw their consent to trial participation at any time without the need to justify the decision.

This will however mean that no further information may be collected for the purpose of the trial and negative implications for the scientific value may be the consequence. Furthermore it may mean that further patient follow up on safety cannot occur.

If a patient wants to withdraw consent, the investigator must be involved in the discussion with the patient and explain the difference between trial treatment discontinuation and withdrawal of consent to trial participation, as well as explain the options for continued follow-up after trial treatment discontinuation, please see Section 3.3.4.1 above.

3.3.4.3 Discontinuation of the trial by the sponsor

Boehringer Ingelheim reserves the right to discontinue the trial overall or at a particular trial site at any time for the following reasons:

1. Failure to meet expected enrolment goals overall or at a particular trial site.

Proprietary confidential information © 2019 Boehringer Ingelheim International GmbH or one or more of its affiliated companies

2. Emergence of any efficacy/safety information invalidating the earlier positive benefit-risk-assessment that could significantly affect the continuation of the trial.
3. Deviations from GCP, the trial protocol, or the contract impairing the appropriate conduct of the trial.

Further follow up of patients affected will occur as described in Section 3.3.4.1.
The investigator / the trial site will be reimbursed for reasonable expenses incurred in case of trial termination (except in case of the third reason).

Proprietary confidential information © 2019 Boehringer Ingelheim International GmbH or one or more of its affiliated companies

4. TREATMENTS

4.1 INVESTIGATIONAL TREATMENTS

The trial medication will be provided by Boehringer Ingelheim Pharma GmbH & Co.KG.

4.1.1 Identity of the Investigational Medicinal Products

The characteristics of test products are below:

Table 4.1.1: 1 Test product 1

Substance:	Empagliflozin
Pharmaceutical formulation:	Film-coated tablet
Source:	Boehringer Ingelheim GmbH & Co.KG
Unit strength:	10 mg
Posology	1 tablet once daily
Route of administration:	Oral

Table 4.1.1: 2 Test product 2

Substance:	Placebo to Empagliflozin 10 mg
Pharmaceutical formulation:	Film-coated tablet
Source:	Boehringer Ingelheim GmbH & Co.KG
Unit strength:	-
Posology	1 tablet once daily
Route of administration:	Oral

4.1.2 Selection of doses in the trial and dose modifications

Empagliflozin 10 mg and 25 mg are approved for the treatment of T2DM.

Proprietary confidential information © 2019 Boehringer Ingelheim International GmbH or one or more of its affiliated companies

Empagliflozin exerts its effect by promoting glucosuria and consequent haemodynamic changes associated with diuresis, improvement in arterial stiffness, blood pressure lowering effect with no increase in heart rate and reduction in heart rate multiplied by pressure product, an index of myocardial oxygen consumption. These modes of actions support the scientific rationale of testing empagliflozin in patients with HF.

In the EMPA-REG-OUTCOME trial both doses were administered to patients with T2DM and showed to be equally effective in reducing CV death, HHF, and composite of HHF or CV death in patients with HF at baseline.

In a subgroup analysis, empagliflozin improved the main outcome of CV death and HHF with a similar magnitude in patients with low or high levels of HbA1c at baseline and irrespective of the dose of empagliflozin [P18-10152]. This indicates the risk reduction for this outcome is independent of the degree of glycaemic control at baseline, suggesting that these benefits can be achieved with the 10 mg dose similar to the 25 mg dose in the non-diabetic population. The mechanism of action is supported by studies in healthy volunteers where both doses were associated with a glucosuria of about 50g per day.

Given the lower exposure with empagliflozin 10 mg and similar general safety, and similar observed CV effects for both doses, empagliflozin 10 mg once daily has been selected as the dose in this trial.

For further details see current version of the IB [c01678844].

4.1.3 Method of assigning patients to treatment groups

After the assessment of all inclusion and exclusion criteria, each eligible patient will be randomised to receive empagliflozin 10 mg, or matching placebo according to a randomisation plan in a 1:1 ratio, stratified according to HF status (de novo or decompensated chronic) at Visit 2a via Interactive Response Technology (IRT). Note that the medication number is different from the patient number (the latter is generated during screening via the IRT System).

To facilitate the use of the IRT, the Investigator or delegate will receive a manual including all necessary instructions for using the system. A copy of the manual will be available in the ISF.

Patient assignment to a treatment group will be determined by a computer generated random sequence. Access to the randomisation code will be controlled and documented - for further details please refer to Section 4.1.5.1. and 4.1.5.2.

Using this procedure, relevant parties will be blinded to the treatment group assignment.

4.1.4 Drug assignment and administration of doses for each patient

Patients who qualify will be randomised to one of the treatment groups described in Section 4.1.1. Trial medication will be dispensed by the pharmacist or the investigator in a double-blind manner.

Proprietary confidential information © 2019 Boehringer Ingelheim International GmbH or one or more of its affiliated companies

IRT will be used to allocate trial medication to patients. At Visit 2a patients will be assigned one medication kit for a 30 days treatment period and 5 days of reserve. At Visit 4 patients will be assigned two medication kits for 30 days treatment period and 5 days of reserve each.

Treatment starts on the day of Visit 2a and ends on the day of Visit 5 (or early discontinuation visit). For further details regarding packaging please refer to Section 4.1.6.

From the start of the treatment period patients will be instructed to take the trial medication once daily with a glass of water. Empagliflozin can be taken with or without food.

To ensure a dose interval of about 24 hours, the medication should be taken in the morning at approximately the same time every day including the days of study visits. If a dose is missed by more than 12 hours, that dose should be skipped and the next dose should be taken as scheduled. No double doses should be taken.

In case trial medication was discontinued for a temporary reason or by the patient without a medical indication, trial treatment can be resumed at any time until Visit 5 (EoT). Trial medication should be restarted if and as soon as medically justified (see also Section 3.3.4.1 and 4.2.1).

4.1.5 Blinding and procedures for unblinding

4.1.5.1 Blinding

Patients, investigators, central reviewers, and everyone involved in trial conduct or analysis or with any other interest in this double-blind trial will remain blinded with regard to the randomised treatment assignments until after database lock.

The access to the randomisation code will be restricted until its release for analysis.

The DMC will be provided with unblinded data in order to allow them to review efficacy and safety and to fulfil their tasks as outlined in the data monitoring committee charter. An independent team, not otherwise involved in the conduct of the trial, will provide the unblinded results to the DMC.

4.1.5.2 Unblinding and breaking the code

Emergency unblinding will be available to the investigator via IRT. It must only be used in an emergency situation when the identity of the trial drug must be known to the investigator in order to provide appropriate medical treatment or otherwise assure safety of trial participants. The reason for unblinding must be documented in the source documents and/or appropriate CRF page.

Due to the requirements to report Suspected Unexpected Serious Adverse Reactions (SUSARs), it may be necessary for a representative from BI's Pharmacovigilance group to access the randomisation code for individual patients during trial conduct. The access to the code will only be given to authorised Pharmacovigilance (PV) representatives for processing in the PV database system and not be shared further.

Proprietary confidential information © 2019 Boehringer Ingelheim International GmbH or one or more of its affiliated companies

4.1.6 Packaging, labelling, and re-supply

The investigational medicinal products will be provided by BI or a designated Contract Research Organisation (CRO). They will be packaged and labelled in accordance with the principles of Good Manufacturing Practice (GMP). Re-supply to the sites will be managed via an IRT system, which will also monitor expiry dates of supplies available at the sites.

For details of packaging and the description of the label, refer to the ISF.

4.1.7 Storage conditions

Drug supplies will be kept in their original packaging and in a secure limited access storage area according to the recommended storage conditions on the medication label. A temperature log must be maintained for documentation.

If the storage conditions are found to be outside the specified range, the Clinical Research Associate (CRA) or the Clinical Trial Manager (CT Manager), as provided in the list of contacts, must be contacted immediately.
If the storage conditions are found to be outside the specified range, the process outlined in the ISF should be followed.

4.1.8 Drug accountability

The investigator or designee will receive the investigational drugs delivered by the sponsor when the following requirements are fulfilled:

- Approval of the clinical trial protocol by the Institutional Review Board (IRB) / ethics committee (EC),
- Availability of a signed and dated clinical trial contract between the sponsor and the investigational site,
- Approval/notification of the regulatory authority, e.g. competent authority,
- Availability of the curriculum vitae of the Principal Investigator,
- Availability of a signed and dated clinical trial protocol,
- Availability of the proof of a medical license for the Principal Investigator (if applicable),
- Availability of FDA Form 1572 (if applicable).

Investigational drugs are not allowed to be used outside the context of this protocol. They must not be forwarded to other investigators or clinics. Patients should be instructed to return unused investigational drug.

The investigator or designee must maintain records of the product's delivery to the trial site, the inventory at the site, the use by each patient, and the return to the sponsor or warehouse / drug distribution centre or alternative disposal of unused products. If applicable, the sponsor or warehouse / drug distribution centre will maintain records of the disposal.

These records will include dates, quantities, batch / serial numbers, expiry ('use- by') dates, and the unique code numbers assigned to the investigational medicinal product and trial patients. The investigator or designee will maintain records that document adequately that the

Proprietary confidential information © 2019 Boehringer Ingelheim International GmbH or one or more of its affiliated companies

patients were provided the doses specified by the Clinical Trial Protocol (CTP) and reconcile all investigational medicinal products received from the sponsor. At the time of return to the sponsor <and/or> appointed CRO, the investigator or designee must verify that all unused or partially used drug supplies have been returned by the clinical trial patient and that no remaining supplies are in the investigator's possession.

4.2 OTHER TREATMENTS, EMERGENCY PROCEDURES, RESTRICTIONS

4.2.1 Other treatments and emergency procedures

The use of medication for the treatment of HF will be at the discretion of the Investigator and should be in accordance with local/international guidelines.

All concomitant (additional) medications and other therapies should be recorded on the appropriate pages of the electronic Case Report Form (eCRF).

Concomitant antidiabetic medications should be adjusted individually as clinically indicated by the patient's usual diabetes care provider. Additional guidance is provided in the IB. Restrictions of antidiabetic background therapy are described in Section 4.2.2.

Patients without a diagnosis of DM experiencing repeated or severe symptoms such as nervousness, sweating, intense hunger, trembling, weakness and palpitations should contact the Investigator or other healthcare professional, as these symptoms might be suggestive of hypoglycaemia. In the case of hypoglycaemia, in patients with or without DM, that may put the patient at risk (e.g. repeated symptomatic hypoglycaemia or severe hypoglycaemia), appropriate care should be provided at the discretion of the Investigator.

Special attention must be paid to the prevention of ketoacidosis. All patients must be made aware of this risk and need to be instructed to contact the Investigator or other healthcare professional in case of symptoms of metabolic acidosis, ketoacidosis and DKA.

Cases of DKA have been reported in patients treated with empagliflozin, including fatal cases. In a number of reported cases, the presentation of the condition was atypical with only moderately increased blood glucose values; below 14 mmol/l (250 mg/dl).

The risk of DKA must be considered in the event of non-specific symptoms such as nausea, vomiting, anorexia, abdominal pain, excessive thirst, difficulty breathing, confusion, unusual fatigue or sleepiness.

Patients should be assessed and treated for ketoacidosis immediately according to local guidelines if these symptoms occur, regardless of blood glucose level. If ketoacidosis is suspected, the trial medication should be discontinued, the patient should be evaluated, and prompt treatment should be initiated.

Patients who may be at higher risk of ketoacidosis while taking empagliflozin include patients on a very low carbohydrate diet (as the combination may further increase ketone body production), patients with an acute illness, pancreatic disorders suggesting insulin deficiency (e.g. Type 1 diabetes mellitus (T1DM), history of pancreatitis or pancreatic

Proprietary confidential information © 2019 Boehringer Ingelheim International GmbH or one or more of its affiliated companies

surgery), insulin dose reduction (including insulin pump failure), alcohol abuse, severe dehydration, and patients with a history of ketoacidosis Empagliflozin should be used with caution in these patients. In patients requiring insulin, caution should be taken when the dose of insulin is reduced.

In clinical situations known to predispose to ketoacidosis (e.g. prolonged fasting due to acute illness or surgery), the Investigator should consider monitoring for ketoacidosis and temporarily discontinue the trial medication.

There are no trial specific emergency procedures to be followed.

In patients with heart failure who receive empagliflozin, elderly patients with heart failure and in case of conditions that may lead to fluid loss (e.g. gastrointestinal illness), careful monitoring of volume status (e.g. physical examination, laboratory tests including haematocrit), blood pressure measurement and electrolytes is recommended since empagliflozin may potentially lead to hypotension. Temporary interruption of the study drug should be considered until the fluid loss is corrected.

Temporary interruption of empagliflozin should be considered in patients with complicated urinary tract infections.

4.2.2 Restrictions

4.2.2.1 Restrictions regarding concomitant treatment

The use of any SGLT-2 inhibitors or combined SGLT-1 and 2 inhibitors except the blinded trial medication is prohibited during the course of the trial. This also includes the 7 days period between the End of Treatment (EoT) and the Follow-up Visit.

If any restricted treatment is given during the conduct of the trial, the trial medication can be discontinued temporarily, or if needed permanently.

If the patient is in need of any additional treatment during this period, this may be given at the discretion of the Investigator. The patient can still remain on trial medication.

4.2.2.2 Restrictions on diet and life style

None.

4.2.2.3 Contraception requirements

Women of childbearing potential must use highly effective methods of birth control per ICH M3 (R2) that result in a low failure rate of less than 1% per year when used consistently and correctly. A list of contraception methods meeting these criteria is provided in the patient information.

4.3 TREATMENT COMPLIANCE

Patients are requested to bring all remaining trial medication including empty package material with them when attending visits 3, 4 and 5 (EoT).

Proprietary confidential information © 2019 Boehringer Ingelheim International GmbH or one or more of its affiliated companies

Based on tablet counts, treatment compliance will be calculated as shown in the formula below. Compliance will be verified by the CRA authorised by the sponsor.

$$\text{Treatment compliance (\%)} = \frac{\text{Number of tablets actually taken} \times 100}{\text{Number of tablets which should have been taken as directed by the investigator}}$$

If the number of doses taken is not between 80-120%, site staff will explain to the patient the importance of treatment compliance. However, randomised patients will not be discontinued for poor compliance without prior discussion with the monitor or designee.

Patients who are not compliant with their medication should again be carefully interviewed and again re-informed about the purpose and the conduct of the trial.

Proprietary confidential information © 2019 Boehringer Ingelheim International GmbH or one or more of its affiliated companies

5. ASSESSMENTS

5.1 ASSESSMENT OF EFFICACY

5.1.1 Net Clinical Benefit

Clinical benefit is a composite of death, HFEs (including HHFs, urgent heart failure visits and unplanned outpatient visits) and KCCQ-CSS.

Investigators will evaluate patients at each study visit and assess for any interim hospitalisation, urgent heart failure visit or unplanned outpatient visit and record the patient response on the dedicated CRFs. Death and HFEs will be categorised by the investigator according to pre-specified criteria [R19-3245] (more details can be found in the ISF). Attempts should be made to obtain supporting medical records for these healthcare interactions.

KCCQ is a commonly used 23 item self-administered questionnaire designed to evaluate physical limitations, symptoms (frequency, severity, and changes over time), social limitations, self-efficacy, and quality of life in patients with HF (refer to Appendix 10.1).

The questionnaire takes less than 15 minutes to complete and will be assessed according to the Flow Chart. Please refer to Section 6.2.2 for order of completion of patient reported outcome measures.

The Investigator (or designated site-personnel) should ensure that the patient has access to a quiet area at the site where he/she can be left alone to record her/his response in the questionnaire. In instances where a patient cannot give or decide upon a response, no response should be recorded. The Investigator (or designated site-personnel) should check that all items have been completed by the patient, but the response to each item should not be scrutinised. Instructions to patients are included in the questionnaire. The respective procedure for illiterate patients (if included) is described in the Appendix 10.2.

5.1.2 NT-proBNP and BNP

NT-proBNP or BNP measurements available from standard of care (local laboratory) will be used in all patients to determine eligibility.
NT-proBNP will be obtained at all visits from Visit 2 until Visit 5 (see Flow Chart) using central laboratory to investigate a potential effect of the trial medication.

5.1.3 Clinical Congestion Score

Patient's congestion will be assessed using a clinician-based outcome assessment of 6 different signs and symptoms: dyspnoea, orthopnoea, fatigue, jugular venous pressure (JVP) (as assessed by the investigator), rales, and oedema. Each category will be assessed through a four-measure questionnaire which will be further converted to a standardized 4-point scale ranging from 0 to 3 as shown in Table 5.1.3: 1.

Proprietary confidential information © 2019 Boehringer Ingelheim International GmbH or one or more of its affiliated companies

Table 5.1.3: 1 Clinical congestion score

**Signs/Symptoms**	**0**	**1**	**2**	**3**
Dyspnoea	None	Seldom	Frequent	Continuous
Orthopnoea	None	Seldom	Frequent	Continuous
Fatigue	None	Seldom	Frequent	Continuous
JVP (cm H2O) (Jugular Venous Pressure[5])	≤6	6< JVP < 10	10≤ JVP <15	≥15
Rales	None	Bases	From base to <50%	From base to >50%
Oedema	Absent/ trace	Slight	Moderate	Marked

The Clinical Congestion Score will be completed according to the Flow Chart. Please refer to Section 6.2.2 for preferred order of completion of assessments.

5.1.4 New York Heart Association classification

The NYHA functional classification will be used to classify the severity of the patients' heart failure. The investigator should place the patients in one of the four categories based on how limited their physical activity are.

Table 5.1.4: 1 New York Heart Association classification

**Class**	**Patient symptoms**
I	No limitation of physical activity. Ordinary physical activity does not cause undue fatigue, palpitation, dyspnoea (shortness of breath)
II	Slight limitation of physical activity. Comfortable at rest. Ordinary physical activity results in fatigue, palpitation, dyspnoea (shortness of breath)
III	Marked limitation of physical activity. Comfortable at rest. Less than ordinary activity causes fatigue, palpitation, or dyspnoea
IV	Unable to carry on any physical activity without discomfort. Symptoms of heart failure at rest. If any physical activity is undertaken, discomfort increases

The classification of patient's physical activity according to NYHA will be performed at Visits 2a, 3, 4, 5 and at early discontinuation.

[5] Jugular venous pressure should be estimated by adding 5 cm H_2O to the vertical distance of the peak jugular venous pulsation from the sternal angle

Proprietary confidential information © 2019 Boehringer Ingelheim International GmbH or one or more of its affiliated companies

5.2 ASSESSMENT OF SAFETY

5.2.1 Physical examination

A complete physical examination will be performed at the time points specified in the Flow Chart. Complete physical examination will include general appearance as well as evaluation of organ systems including an assessment of the cardiovascular system.

The results must be included in the source documents available at the site.

5.2.2 Vital signs

Vital signs will be evaluated at the time points specified in the Flow Chart, prior to blood sampling. This includes systolic and diastolic blood pressure and pulse rate (electronically or by palpation count for 1 minute) in a seated position after 5 minutes of rest.

The preferred method for blood pressure measurement is by a standard mercury sphygmomanometer. If a standard mercury sphygmomanometer is not available, alternative devices recommended by website www.dableducational.org may be used or devices approved for use by the appropriate national agency/ies.

At visit 2a, after the patient has rested quietly in the seated position for five minutes, blood pressure should be taken in both arms. If the pressures differ by more than 10 mmHg (as for example in the presence of a subclavian steal syndrome), the pressure from the arm with the higher pressure (systolic or diastolic) should be entered in the eCRF and this arm should be used for subsequent measurements. The same method and, if possible, the same device should be used throughout the trial for a patient.

Blood pressure measurements should be recorded to the nearest 2 mmHg only when measured with a manual sphygmomanometer; when digital devices are used the value from the device should be rounded to the nearest 1 mmHg.

For eCRF entry, decimal places should be rounded to integers (e.g. a DBP of 94.5 would be rounded to 95 mmHg and a SBP of 109.4 would be rounded to 109 mmHg).

The results must be included in the source documents available at the site.

5.2.3 Body weight and height

Measurements of body weight and height will be performed at the time points specified in the Flow Chart.

Body weight should be measured as follows:

- after the urine sampling (weight after bladder voiding),
- shoes and coat/jackets should be taken off, and
- pockets should be emptied of heavy objects (i.e. keys, coins etc.).

Proprietary confidential information © 2019 Boehringer Ingelheim International GmbH or one or more of its affiliated companies

5.2.4 Safety laboratory parameters

Safety laboratory parameters needed for assessing patients eligibility including NT-proBNP/BNP will be assessed based on local lab.

Safety laboratory parameters to be assessed during the trial are listed in Table 5.2.4: 1. For the sampling time points please see the Flow Chart.

All analyses will be performed by a central laboratory, the respective reference ranges will be provided in the ISF. Patients do not have to be fasted for the blood sampling for the safety laboratory.

Instructions regarding sample collection, sample handling/ processing and sample shipping are provided in the Laboratory Manual in the ISF.
The central laboratory will send reports to the investigator. It is the responsibility of the investigator to evaluate the laboratory reports. Clinically relevant abnormal findings as judged by the investigator will be reported as adverse events (please refer to Section 5.2.7).

In case the criteria for hepatic injury are fulfilled, a number of additional measures will be performed (please see Section 5.2.7.1 and the DILI Checklist provided in the ISF and electronic data capture (eDC) system. The amount of blood taken from the patient concerned will be increased due to this additional sampling.
The central laboratory will transfer the results of the analysis to the sponsor.

Proprietary confidential information © 2019 Boehringer Ingelheim International GmbH or one or more of its affiliated companies

Table 5.2.4: 1 Safety laboratory tests

Haematology
Hematocrit<br>Haemoglobin (Hb)<br>Reticulocyte Count (reflex test if Hb outside normal range)<br>Red Blood Cells (RBC) / Erythrocytes<br>WBC / Leukocytes<br>Platelet Count / Thrombocytes
Clinical chemistry
Albumin<br>Alkaline phosphatase<br>γ-GT (gamma-glutamyl transferase) reflex test triggered by elevated alkaline phosphatase on two sequential measures<br>ALT (alanine transaminase, SGPT)<br>AST (aspartate transaminase, SGOT)<br>Bicarbonate<br>Bilirubin total, fractionated if increased<br>Chloride<br>Creatinine<br>Creatine kinase (CK)<br>High sensitivity Troponin I (reflex tests, if CK is elevated)<br>Glucose<br>Potassium<br>Protein total<br>Sodium<br>Urea (blood urea nitrogen (BUN)<br>Uric acid
Urine
Urinalysis: dipstick test including for example (but not limited to) ketones semi-quantitative measurement, white blood cells, blood<br>(glucose measurement in urine will not be performed)

5.2.4.1 Renal Function

The estimated glomerular filtration rate (eGFR) will be derived from serum creatinine values, age, sex and race based on the CKD-EPIcr equation [R12-1392].

Proprietary confidential information © 2019 Boehringer Ingelheim International GmbH or one or more of its affiliated companies

5.2.4.2 Pregnancy Testing

Pregnancy testing (urine) will be performed in female patients of child bearing potential according to the time points indicated in the Flow Chart. Pregnancy kits will be provided by the Central Laboratory. For reporting of pregnancy event refer to Section 5.2.7.2.

5.2.4.3 Criteria for hypoglycaemic events

All symptomatic hypoglycaemia events, or severe hypoglycaemias (e.g. if the patient required assistance of another person), or any hypoglycaemia episode with glucose values < 54 mg/dl (< 3.0 mmol/l), or if the investigator considered the event to be an AE should be documented as an AE "hypoglycaemic event". In non-diabetic or pre-diabetic patients, the investigator should consider and rule out other alternative causes for such symptoms and can perform blood glucose levels to confirm the diagnosis of hypoglycaemia.

5.2.4.4 Urinary tract infection and genital infections

Patients having a history of chronic/recurrent urinary tract infections (UTI) or genital infections, or an acute episode of UTI or genital infection at screening will be identified and this condition has to be documented as medical history or baseline condition in the eCRF, respectively.

For documentation of symptomatic acute UTI during trial conduct, a urine culture sample has to be taken and sent to central lab for confirmation of the diagnosis.

5.2.5 Electrocardiogram

12-lead ECGs will be performed at Visits 2a and 5 (EoT) as indicated in the Flow Chart. The investigator or a designee will evaluate whether the ECG is normal or abnormal and assess clinical relevance. ECGs may be repeated for quality reasons and a repeated recording used for analysis.

Additional ECGs may be recorded for safety reasons. Dated and signed printouts of ECG with findings should be documented in patient's medical record.

Clinically relevant abnormal findings will be reported either as baseline condition (if identified at the screening visit) or otherwise as AEs and will be followed up and/or treated as medically appropriate.

5.2.6 Other safety parameters

There are no additional safety parameters in the trial.

Proprietary confidential information © 2019 Boehringer Ingelheim International GmbH or one or more of its affiliated companies

5.2.7 Assessment of adverse events

5.2.7.1 Definitions of AEs

5.2.7.1.1 Adverse event

An AE is defined as any untoward medical occurrence in a patient or clinical investigation subject administered a medicinal product and which does not necessarily have to have a causal relationship with this treatment.

An AE can therefore be any unfavourable and unintended sign (including an abnormal laboratory finding), symptom, or disease temporally associated with the use of a medicinal product, whether or not considered related to the medicinal product.

The following should also be recorded as an AE in the CRF and BI SAE form (if applicable):

- Worsening of the underlying disease or of other pre-existing conditions.
- Changes in vital signs, ECG, physical examination and laboratory test results, if they are judged clinically relevant by the investigator.

If such abnormalities already exist prior to trial inclusion, they will be considered as baseline conditions and should be collected in the eCRF only.

5.2.7.1.2 Serious adverse event

A serious adverse event (SAE) is defined as any AE, which fulfils at least one of the following criteria:

- results in death,
- is life-threatening, which refers to an event in which the patient was at risk of death at the time of the event; it does not refer to an event that hypothetically might have caused death if more severe,
- requires inpatient hospitalisation or prolongation of existing hospitalisation,
- results in persistent or significant disability or incapacity,
- is a congenital anomaly / birth defect,
- is deemed serious for any other reason if it is an important medical event when based on appropriate medical judgement which may jeopardise the patient and may require medical or surgical intervention to prevent one of the other outcomes listed in the above definitions. Examples of such events are intensive treatment in an emergency room or at home for allergic bronchospasm, blood dyscrasias or convulsions that do not result in hospitalisation or development of dependency or abuse.

5.2.7.1.3 AEs considered "Always Serious"

In accordance with the European Medicines Agency initiative on Important Medical Events, Boehringer Ingelheim has set up a list of AEs, which by their nature, can always be considered to be "serious" even though they may not have met the criteria of an SAE as defined above.

Proprietary confidential information © 2019 Boehringer Ingelheim International GmbH or one or more of its affiliated companies

The latest list of "Always Serious AEs" can be found in the eDC system. A copy of the latest list of "Always Serious AEs" will be provided upon request. These events should always be reported as SAEs as described in Section 5.2.7.2.

Cancers of new histology and exacerbations of existing cancer must be classified as a serious event regardless of the time since discontinuation of the drug and must be reported as described in 5.2.7.2, subsections "AE Collection" and "AE reporting to sponsor and timelines".

5.2.7.1.4 Adverse events of special interest

The term adverse events of special interest (AESI) relates to any specific AE that has been identified at the project level as being of particular concern for prospective safety monitoring and safety assessment within this trial, e.g. the potential for AEs based on knowledge from other compounds in the same class. AESIs need to be reported to the sponsor's Pharmacovigilance Department within the same timeframe that applies to SAEs, please see Section 5.2.7.2.2.

The following are considered as AESIs:

Hepatic injury

A hepatic injury is defined by the following alterations of hepatic laboratory parameters:

- an elevation of AST (Aspartate Aminotransferase) and/or ALT (Alanine Aminotransferase) ≥3 fold ULN combined with an elevation of total bilirubin ≥2 fold ULN measured in the same blood draw sample, or
- Aminotransferase (ALT, and/or AST) elevations ≥5 fold ULN.

These lab findings constitute a hepatic injury alert and the patients showing these lab abnormalities need to be followed up according to the eDC system.

In case of clinical symptoms of hepatic injury (icterus, unexplained encephalopathy, unexplained coagulopathy, right upper quadrant abdominal pain, etc.) without lab results (ALT, AST, total bilirubin) available, the investigator should make sure these parameters are analysed, if necessary in an unscheduled blood test. Should the results meet the criteria of hepatic injury alert, the procedures described in the DILI checklist should be followed.

Decreased renal function

Decreased renal function is defined by a creatinine value showing a ≥ 2 fold increase from baseline and is above the Upper Limit of Normal (ULN).

For the AESI "decreased renal function" patients need to be followed up appropriately based on local clinical guidance.

Proprietary confidential information © 2019 Boehringer Ingelheim International GmbH or one or more of its affiliated companies

Ketoacidosis

If metabolic acidosis, ketoacidosis and DKA is suspected further investigations should be done according to the medical judgment and the clinical course until a diagnosis is made and/or the patient is recovered.

Due to its mechanism of action, empagliflozin may potentially modify the clinical presentation of ketoacidosis which may occur at lower plasma glucose levels in patients with DM and potentially also in non-diabetic patient population. The diagnosis of ketoacidosis in these patients can be based on arterial pH ≤7.30, serum bicarbonate levels <15 mmol/L and measurement of serum beta-hydroxybutrate levels. Other diagnostic criteria which can support the diagnosis of ketoacidosis are urine ketones and anion gap >10 mmol/L.

Investigators should note that not all criteria mentioned above need to apply for the diagnosis of ketoacidosis, and clinical judgment should also be taken into consideration.

5.2.7.1.5 Intensity (severity) of AEs

The intensity (severity) of the AE should be judged based on the following:

Mild: Awareness of sign(s) or symptom(s) that is/are easily tolerated.
Moderate: Sufficient discomfort to cause interference with usual activity.
Severe: Incapacitating or causing inability to work or to perform usual activities.

5.2.7.1.6 Causal relationship of AEs

Medical judgement should be used to determine whether there is a reasonable possibility of a causal relationship between the adverse event and the given study treatment, considering all relevant factors, including pattern of reaction, temporal relationship, de-challenge or re-challenge, confounding factors such as concomitant medication, concomitant diseases and relevant history.

Arguments that may suggest that there is a reasonable possibility of a causal relationship could be:

- The event is consistent with the known pharmacology of the drug.
- The event is known to be caused by or attributed to the drug class.
- A plausible time to onset of the event relative to the time of drug exposure.
- Evidence that the event is reproducible when the drug is re-introduced.
- No medically sound alternative aetiologies that could explain the event (e.g. pre-existing or concomitant diseases, or co-medications).
- The event is typically drug-related and infrequent in the general population not exposed to drugs (e.g. Stevens-Johnson syndrome).
- An indication of dose-response (i.e. greater effect size if the dose is increased, smaller effect size if dose is reduced).

Arguments that may suggest that there is no reasonable possibility of a causal relationship could be:

Proprietary confidential information © 2019 Boehringer Ingelheim International GmbH or one or more of its affiliated companies

- No plausible time to onset of the event relative to the time of drug exposure is evident (e.g. pre-treatment cases, diagnosis of cancer or chronic disease within days / weeks of drug administration; an allergic reaction weeks after discontinuation of the drug concerned).
- Continuation of the event despite the withdrawal of the medication, taking into account the pharmacological properties of the compound (e.g. after 5 half-lives). Of note, this criterion may not be applicable to events whose time course is prolonged despite removing the original trigger.
- Additional arguments amongst those stated before, like alternative explanation (e.g. situations where other drugs or underlying diseases appear to provide a more likely explanation for the observed event than the drug concerned).
- Disappearance of the event even though the trial drug treatment continues or remains unchanged.

5.2.7.2 Adverse event collection and reporting

5.2.7.2.1 AE Collection

The investigator shall maintain and keep detailed records of all AEs in the patient files.

The following must be collected and documented on the appropriate CRF(s) by the investigator:

- From signing the informed consent onwards until the individual patient's end of trial participation (End of Study (EOS)):
  all AEs (serious and non-serious) and all AESIs.
- After the individual patient's end of trial:
  the investigator does not need to actively monitor the patient for new AEs but should only report any occurrence of cancer and trial treatment related SAEs and trial treatment related AESIs of which the investigator may become aware of by any means of communication, e.g. phone call. Those AEs should be reported on the BI SAE form (see Section 5.2.7.2.2), but not on the CRF.

For some types of AEs additional information will be collected in the eCRF due to the nature of the event and mechanisms of action of the trial medication. These listed AEs are distinct from AESI. The list of types of AEs for which additional information will be collected may change during the trial based on potential new knowledge about the safety profile of empagliflozin:

- Hypoglycaemic event
- Bone fracture
- Hypotension

5.2.7.2.2 AE reporting to the sponsor and timelines

The investigator must report SAEs, AESIs, and non-serious AEs which are relevant for the reported SAE or AESI, on the BI SAE form via fax immediately (within 24 hours) to the sponsor's unique entry point (country specific contact details will be provided in the ISF). The same timeline applies if follow-up information becomes available. In specific occasions,

Proprietary confidential information © 2019 Boehringer Ingelheim International GmbH or one or more of its affiliated companies

the investigator could inform the sponsor upfront via telephone. This does not replace the requirement to complete and fax the BI SAE form.

With receipt of any further information to these events, a follow-up SAE form has to be provided. For follow-up information the same rules and timeline apply as for initial information. All (S)AEs, including those persisting after individual patient's end of trial must be followed up until they have resolved, have been assessed as "chronic" or "stable" , or no further information can be obtained.

5.2.7.2.3 Pregnancy

In rare cases, pregnancy might occur in a clinical trial. Once a patient has been enrolled in the clinical trial and has taken trial medication, the investigator must report any drug exposure during pregnancy in a trial participant immediately (within 24 hours) by means of Part A of the Pregnancy Monitoring Form to the sponsor's unique entry point.

The outcome of the pregnancy associated with the drug exposure during pregnancy must be followed up and reported to the sponsor's unique entry point on the Pregnancy Monitoring Form for Clinical Studies (Part B).
The ISF will contain the Pregnancy Monitoring Form for Clinical Studies (Part A and B).

As pregnancy itself is not to be reported as an AE, in the absence of an accompanying SAE and/or AESI, only the Pregnancy Monitoring Form for Clinical Studies and not the SAE form is to be completed. If there is an SAE and/or AESI associated with the pregnancy an SAE form must be completed in addition.

5.3 DRUG CONCENTRATION MEASUREMENTS AND PHARMACOKINETICS

This study will not analyse pharmacokinetic or pharmacodynamics parameters.

5.4 ASSESSMENT OF BIOMARKER(S)

Established biomarkers of efficacy and safety are described and discussed in Section 5.1 and 5.2.

This study will not analyse exploratory biomarkers.

5.5 BIOBANKING

Not applicable.

5.6 OTHER ASSESSMENTS

5.6.1 Health Care Resource Utilisation (HCRU)

HCRU will be captured via interview with the patient and verified against medical records where available and entered in the eCRF at the visits specified in the Flowchart. Information on utilization of following resources will be collected:

Proprietary confidential information © 2019 Boehringer Ingelheim International GmbH or one or more of its affiliated companies

- All-cause hospital admissions (first and recurrent).
- Hospital admission due to worsening of heart failure (first and recurrent).
- All-cause emergency room visits (first and recurrent).
- Emergency room visits due to worsening of heart failure (requiring i.v. diuretic therapy) (first and recurrent).
- Any unscheduled outpatient visits (first and recurrent).
- Unscheduled outpatient visits related to heart failure (first and recurrent).
- Length of hospital stay(s) (initial and recurrent).
- Length of ICU stay during hospital stay (initial and recurrent).
- Intensification of diuretic therapy (adding a new diuretic, increase of dose).

5.7 APPROPRIATENESS OF MEASUREMENTS

All measurements performed during this trial are standard measurements and will be performed in order to monitor safety aspects, and to determine empagliflozin efficacy and safety in an appropriate way.

For more details on NT-proBNP, please refer to Section 3.2.

The scheduled measurements are appropriate to see drug induced changes in vital signs, standard laboratory values, the biomarker NT-proBNP, and in ECG. The primary and secondary endpoints are accepted for evaluation of efficacy, safety and tolerability of an oral HF drug and they are widely used in respective pivotal phase III HF studies.

Health related quality of life questionnaires (specifically the KCCQ) are a necessary part for this phase III trial in order to collect data on patient related outcomes (as part of the primary endpoint).

Therefore, the appropriateness of all measurements applied in this trial is given.

Proprietary confidential information © 2019 Boehringer Ingelheim International GmbH or one or more of its affiliated companies

6. INVESTIGATIONAL PLAN

6.1 VISIT SCHEDULE

All patients are to adhere to the visit schedule as specified in the Flow Chart. If any visit has to be rescheduled, subsequent visits should follow the original visit date schedule. The trial medication packs contain sufficient medication to allow for the allowed time windows.

All trial visits should take place at approximately the same time of day and preferably before noon.

6.2 DETAILS OF TRIAL PROCEDURES AT SELECTED VISITS

Please refer to the Flow Chart and Section 5 for details of the procedures performed at each visit.

6.2.1 Screening and run-in period(s)

Due to the short time window when patients can be randomised (between 24 hours after hospitalisation and no later than 5 days), potential study patients should be identified as soon as possible and their improvement followed up accordingly (pre-screening). Potential study patients should be approached to assess their interest in volunteering as a participant in this trial as soon as possible.

Screening Period

The investigations for the main diagnosis for inclusion are to be completed and documented per standard of care as a prerequisite to consideration for study participation. Patients will be included in the study based on local BNP or NT-proBNP and other available laboratory measurements.

Following informed consent, the patient will undergo Visit 1/screening assessments as indicated in the Flow Chart. The assessments must all fall within the acceptable screening visit window but do not need to be performed on the same day. The patient should be registered in IRT as a screened patient.

Re-screening within the same hospitalisation and current episode of acute decompensation is not allowed. Nevertheless, if a patient who failed screening before has another episode of acute decompensation that leads to a new hospitalisation, patient can be screened again and considered for the study.

Medical History:

Medical History will be documented using pre-specified categories as given in the eDC system. For detailed requirements please see Section 8.3.1.

Baseline Conditions

Ongoing conditions/diagnosis from the Medical History questionnaire will be documented as Baseline Conditions.

Proprietary confidential information © 2019 Boehringer Ingelheim International GmbH or one or more of its affiliated companies

If the patient meets the entry criteria, Visit 2a should occur as soon as possible once it has been confirmed that the patient is eligible to continue. If the patient does not meet the entry criteria, (i.e. fail to meet one or more of the inclusion criteria, and/or meet one or more of the exclusion criteria) following Visit 1 procedures, they should be registered as a screen failure in IRT.

6.2.2 Treatment period(s)

After a final check of all in- and exclusion criteria, patients eligible will be randomised at Visit 2a using IRT. Randomisation can occur at the same day as the screening visit. All Visit 2a assessments must be performed before the first dose is taken in hospital. Assessments from Visit 2a will be used as baseline values.

Patients will undergo visit assessments as indicated in the Flow Chart.

Patients will have further visits with a reduced number of assessments two and four days thereafter if they are still hospitalised (Visits 2b and 2c at Day 3 and 5).

The patients will return to the clinic for regularly scheduled Visits 3, 4 and 5 (EoT) on days 15, 30 and 90 respectively after randomisation as specified in the Flow Chart. These on-site visits will assess the occurrence of safety and efficacy endpoints, trial medication compliance (Visit 3, 4 and 5), concomitant therapy or intervention. The assessments at these visits can be performed post-dose. Patients should be instructed to take their medication on the morning of their visits at home like all days. Visits should be routinely scheduled at approximately the same time of day for each visit.

All other medications the patient is receiving should be taken as instructed by the physician. At any time during the treatment period, the HF background therapy is allowed to be adjusted and optimised according to local and international guidelines.
If any additional therapy is considered necessary for the patient's welfare during the treatment period it may be given at the discretion of the Investigator (see also restrictions in Section 4.2.2).

Patient reported outcome measures (KCCQ) should preferably be done first and laboratory samples should be taken at the end of each visit. All other measures should be performed in between KCCQ and laboratory sampling.

Allocation of medication kit number(s) will be managed through the IRT. Patients will be assigned medication at Visit 2a, starting treatment in hospital and will receive their trial mediation kit at discharge to take home. This kit contains sufficient medication until Visit 4. At this visit patients should return the medication and compliance check will be done. At Visit 4 new medication kits will be dispensed. Visit 5 (EoT) is the last day of treatment and the patient should return all remaining medication during this visit.

Early permanent trial medication discontinuation is only justified when clear persistent contraindications arise, or when the patient requests to stop trial medication. See Section 3.3.4.1 for details on how to handle trial medication discontinuations. An Early Discontinuation Visit should be performed with the procedures indicated in the Flow Chart.

Proprietary confidential information © 2019 Boehringer Ingelheim International GmbH or one or more of its affiliated companies

6.2.3 Follow-up period and trial completion

A Follow-up Visit will be performed 7 days (+7 day window) after the last dose of trial medication. The assessments to be performed at the Follow-up Visit are indicated in the Flow Chart. The Follow-up Visit marks the completion of the study for the individual patient who completed the study on trial medication.

As a standard, Follow-up Visit should be performed as phone call. If considered necessary e.g. for safety reasons requiring personal follow-up, the visit can be done as a site visit.

See Section 3.3.4.1 for procedures to be followed in case a patient prematurely discontinues trial treatment.

For patients who early discontinued trial medication but followed up according to the visit schedule, Visit 5 (EoT) marks the completion of the study for the individual patient.

Proprietary confidential information © 2019 Boehringer Ingelheim International GmbH or one or more of its affiliated companies

7. STATISTICAL METHODS AND DETERMINATION OF SAMPLE SIZE

The eligible patients for this trial will be randomised to one of two treatment groups (empagliflozin or placebo) in a 1:1 ratio, stratified according to HF status (de novo or decompensated chronic HF).

The primary endpoint is net clinical benefit (defined in Section 7.2.2) [R19-3043]. The statistical model will be a non-parametric generalised pairwise comparison within HF status strata. The variance of the net clinical benefit will be calculated using the asymptotic normal approach [R19-3046].

The primary model will be conducted on all randomised patients.

7.1 NULL AND ALTERNATIVE HYPOTHESES

For the primary endpoint, superiority of empagliflozin vs. placebo will be evaluated with one-sided tests, at a significance level of 0.025, in the following structure:

Null hypothesis: There is no difference between the effect of placebo and the effect of empagliflozin or the effect of placebo is greater.

Alternative hypothesis: The effect of empagliflozin is greater than the effect of placebo.

Secondary and further endpoints will be evaluated in an exploratory manner.

7.2 PLANNED ANALYSES

7.2.1 General considerations

The efficacy analysis will be based on the randomised set (RS), including all randomised patients. Analyses will be performed according to the intention-to-treat principle, with the use of all available data (on and off-treatment) through the trial period. This equates to a treatment-policy style estimand. Sensitivity analyses investigating hypothetical estimands (e.g. on-treatment data only) may be specified in the Trial Statistical Analysis Plan (TSAP). If a Per Protocol Set (PPS) is required, this will also be specified in the TSAP. Important protocol deviations will be described in the TSAP.

The safety analysis will be based on the treated set (TS), which consists of all patients treated with at least one dose of trial medication.

For both efficacy and safety analyses, treatment will be evaluated as randomised.

For all endpoints, baseline will be defined as the last available measurement before start of trial medication.

Proprietary confidential information © 2019 Boehringer Ingelheim International GmbH or one or more of its affiliated companies

7.2.2 Primary endpoint analyses

The primary endpoint is net clinical benefit, a composite of death, number of HFEs, time to first HFE and change from baseline in KCCQ-CSS after 90 days of treatment.
All patients randomised to empagliflozin are compared to all patients randomised to placebo.

For any two patients, the patient with the greater net clinical benefit is determined by assessing the following criteria sequentially, stopping when an advantage for either patient is shown:

1. Death within common follow-up time
   - death is worse than no death
   - earlier death is worse
   - tied, if not possible to determine

2. Number of HFEs within common follow-up time
   - more HFEs is worse
   - tied, if same number of HFEs

3. Time to first HFE within common follow-up time
   - earlier HFE is worse
   - tied, if not possible to determine

4. KCCQ-CSS change from baseline at Day 90
   - more positive change from baseline is better

Note, priority is therefore given to death over HFE, and both of these over changes in KCCQ-CSS. Below are some examples:

5. Death, e.g.:
   - Patient A dies 30 days after randomisation (loses)
   - Patient B dies 40 days after randomisation (wins)

6. If no winner based on death, number of HFEs within common follow-up time, e.g.:
   - Patient A had two HFEs (loses)
   - Patient B had one HFE (wins)

7. If no winner based on number of HFEs, time to first HFE, e.g.:
   - Patient A had an HFE 30 days after randomisation (loses)
   - Patient B had an HFE 50 days after randomisation (wins)

8. If no winner based on time to first HFE, KCCQ-CSS change from baseline at Day 90, e.g.:
   - Patient A: KCCQ-CSS change from baseline at Day 90 is x (loses)
   - Patient B: KCCQ-CSS change from baseline at Day 90 is y (y>x) (wins)

Proprietary confidential information © 2019 Boehringer Ingelheim International GmbH or one or more of its affiliated companies

The implemented generalised pairwise comparisons approach compares all patients in one treatment group to all other patients within their strata in the other treatment group. The net benefit (Δ) is then calculated as the total number of wins in the empagliflozin group across all strata minus the total number of losses and then divided by the total number of comparisons.

$$\Delta = \frac{1}{n \times m} \sum_{i=1}^{n} \sum_{j=1}^{m} U_{ij}$$

Where U_{ij} is 1 if the empagliflozin patient wins, -1 if the placebo patient wins and 0 if there is a tie.

n is the number of patients in the empagliflozin group, m is the number of patients in the placebo group.

The variance is calculated by the asymptotic normal distribution of Δ as follows:

$$Var\ (\Delta) = \frac{n+m}{(n \times m)^2} \left(\sum_{i=1}^{n} \sum_{j=1}^{m} \sum_{j'=1}^{m} U_{ij} U_{ij'} + \sum_{j=1}^{m} \sum_{i=1}^{n} \sum_{i'=1}^{n} U_{ij} U_{i'j} \right)$$

This approach is analogous to Van Elteren's test [R98-1473].

Separate summaries for each component of this endpoint will also be presented.

Sensitivity and subgroup analyses for the primary endpoint will be specified in the TSAP.

The method of handling missing KCCQ-CSS values for this analysis is described in Section 7.3.

7.2.3 Secondary endpoint analyses

Secondary endpoints will not be tested in a hierarchical sequence, and no adjustment for multiple comparisons is planned.

Change from baseline in continuous endpoints, such as KCCQ-CSS, will be analysed using restricted maximum likelihood estimation based on a mixed-effect model for repeated measures (MMRM) analysis to obtain adjusted means for the treatment effects. This model will include discrete fixed effects for treatment group (empagliflozin or placebo) and HF status (de novo or decompensated chronic HF) at each visit and continuous fixed effects for baseline value at each visit. Missing data caused by patient withdrawal or other reasons will be handled implicitly by the MMRM approach.

Area under the curve (AUC) of change from baseline in log-transformed NT-proBNP level over 30 days of treatment will be analysed by an analysis of covariance (ANCOVA). Based on literature reviews, NT-proBNP level is regarded as log-normally distributed, therefore values will be log-transformed prior to analysis [R19-3044]. The linear trapezoidal rule will be used to calculate the AUC after the log-transformation has been applied to each value.

Proprietary confidential information © 2019 Boehringer Ingelheim International GmbH or one or more of its affiliated companies

Analysis of covariance (ANCOVA) with a discrete fixed effect for HF status (de novo or decompensated chronic HF) and a continuous fixed effect for baseline NT-proBNP level (log-transformed) will be used to compare treatment groups. The method of handling missing NT-proBNP levels for this analysis is described in Section 7.3.

Comparisons between treatment groups regarding the binary endpoint variable (improvement in KCCQ-CSS of ≥ 10 points after 90 days of treatment) will be performed using a logistic regression model adjusting for the binary covariate HF status (de novo or decompensated chronic HF). The likelihood-ratio test will be used to test for a difference between treatments. Adjusted odds ratios together with 1-sided 97.5% confidence limits will be used to quantify the effect of treatment, comparing empagliflozin to placebo as the reference.

Time to event endpoints will be analysed using the Cox proportional hazards model [R07-4680] with HF status (de novo or decompensated chronic HF) as a covariate. Hazard ratios (HRs) and their associated one-sided 97.5% confidence limits will be estimated for evaluating the superiority of empagliflozin to placebo.

Other secondary endpoints will be summarised descriptively (including days alive and out of hospital).

Analysis of recurrent events will be described in the TSAP.

7.2.4 Further endpoint analyses

Change from baseline in continuous endpoints will be analysed using the same methods as for secondary endpoints.

Comparisons between treatment groups regarding the binary endpoint variables will follow the same approach as for secondary endpoints.

All other further endpoints will be summarised descriptively.

7.2.5 Safety analyses

Adverse events will be coded using the Medical Dictionary for Drug Regulatory Activities (MedDRA). Standard BI summary tables and listings will be produced. All adverse events with an onset between start of treatment and end of the residual effect period (REP), a period of 7 days after the last dose of trial medication, will be assigned to the on-treatment period for evaluation.

All treated patients will be included in the safety analysis. In general, safety analyses will be descriptive in nature and will be based on BI standards. No hypothesis testing is planned.

Statistical analysis and reporting of adverse events will concentrate on treatment-emergent adverse events, i.e. all adverse events occurring between start of treatment and end of the residual effect period. Adverse events that start before first drug intake and deteriorate under treatment will also be considered as 'treatment-emergent'.

Proprietary confidential information © 2019 Boehringer Ingelheim International GmbH or one or more of its affiliated companies

Frequency, severity, and causal relationship of adverse events will be tabulated by system organ class and preferred term after coding according to the current version of the Medical Dictionary for Drug Regulatory Activities (MedDRA) at the database lock.

Laboratory data will be analysed both quantitatively as well as qualitatively. The latter will be done via comparison of laboratory data to their reference ranges. Values outside the reference range as well as values defined as clinically relevant will be summarised. Treatment groups will be compared descriptively with regard to distribution parameters as well as with regard to frequency and percentage of patients with abnormal values or clinically relevant abnormal values.

Vital signs, physical examinations, or other safety-relevant data observed at screening, baseline, during the course of the trial and at the end-of-trial evaluation will be assessed with regard to possible changes compared to findings before start of treatment.

Reasons for discontinuation and use of post-baseline concomitant medications will be tabulated.

The details of the analysis will be specified in the TSAP.

7.2.6 Interim Analyses

No interim analysis is planned but a Data Monitoring Committee (DMC) will be in place with tasks as described in Section 8.7.

7.3 HANDLING OF MISSING DATA

Missing data for KCCQ-CSS and NT-proBNP will be estimated using multiple imputation, according to whether patients are on-treatment or off-treatment.

Further details on multiple imputation and rules for handling missing data for other secondary and further endpoints will be specified in the TSAP.

7.4 RANDOMISATION

The trial will be performed as a double-blind design with respect to placebo and empagliflozin. Patients will be randomised to the trial treatments in a 1:1 ratio. Patients will be stratified by HF status (de novo or decompensated chronic HF).

Patients will be randomised in blocks to double-blind treatment via an IRT system.

Approximately equal numbers of patients will be randomised to each treatment group.

BI will arrange for the randomisation and the packaging and labelling of trial medication. The randomisation list will be generated using a validated system, which involves a pseudo-random number generator so that the resulting treatment will be both reproducible and non-predictable. The block size will be documented in the Clinical Trial Report. Access to the codes will be controlled and documented.

Proprietary confidential information © 2019 Boehringer Ingelheim International GmbH or one or more of its affiliated companies

7.5 DETERMINATION OF SAMPLE SIZE

Powers for superiority in net clinical benefit were estimated by simulation using a shared frailty approach. Initially a frailty value was simulated for each patient using a gamma distribution. Deaths were assumed to have constant hazard for any given patient dependent upon the frailty term, leading to a marginal Lomax distribution. HFEs were assumed to have a Poisson distribution within patients, again depending on the frailty parameter, leading to a marginal negative binomial distribution. The number of HFEs per patient was nominally capped at 7 per patient over the 90 day period. For KCCQ-CSS, the frailty terms were directly transformed into a capped normal distribution (values below 0 or above 100 set to 0 and 100 respectively). High frailties corresponded to low KCCQ-CSS scores and vice versa. The use of a shared frailty parameter ensures correlation between components of the net clinical benefit endpoint so that patients with poor outcomes in one component are also more likely to have a poor outcome in the others. Ten thousand simulations were used for each scenario. Calculations of net clinical benefit were performed using the generalised pairwise comparisons method for net benefit using the asymptotic variance approach and algorithm for net clinical benefit as described in Section 7.2.2. Simulations and calculations were performed using R statistical software Version 3.5.1.

A sample size of 500 randomised patients in total (250 per treatment group) would provide 87.2% power to detect a difference between the empagliflozin treatment group and placebo treatment group, in net clinical benefit, at a one-sided significance level of 0.025, assuming the following:

Proportion of patients who die within 90 days = 5%

Hazard ratio for death component = 0.8

Proportion of patients with at least one HFE within 90 days = 15%

Hazard ratio for HFE component = 0.7

KCCQ-CSS baseline adjusted standard deviation = 20

KCCQ-CSS treatment effect = 6

Table 7.5: 1 Power estimates for various combinations of KCCQ-CSS treatment effects and death/HFE hazard ratios with 500 patients in total

HR for death	HR for HFE	KCCQ-CSS treatment effect			
		4	5	6	7
0.7	0.7	67.4%	80.1%	89.1%	94.5%
0.8	0.7	64.0%	77.3%	**87.2%**	93.3%
0.8	0.8	55.2%	69.1%	81.1%	89.4%

Proprietary confidential information © 2019 Boehringer Ingelheim International GmbH or one or more of its affiliated companies

8. INFORMED CONSENT, TRIAL RECORDS, DATA PROTECTION, PUBLICATION POLICY, AND ADMINISTRATIVE STRUCTURE

The trial will be carried out in compliance with the protocol, the ethical principles laid down in the Declaration of Helsinki, in accordance with the ICH Harmonized Guideline for Good Clinical Practice (GCP), relevant BI Standard Operating Procedures (SOPs), the EU directive 2001/20/EC / EU regulation 536/2014 and other relevant regulations. Investigators and site staff must adhere to these principles. Deviation from the protocol, the principles of ICH GCP or applicable regulations as will be treated as “protocol deviation”.
Standard medical care (prophylactic, diagnostic and therapeutic procedures) remains the responsibility of the treating physician of the patient.

The investigator will inform the sponsor immediately of any urgent safety measures taken to protect the trial patients against any immediate hazard, as well as of any serious breaches of the protocol or of ICH GCP.

The Boehringer Ingelheim transparency and publication policy can be found on the following web page: trials.boehringer-ingelheim.com. The rights of the investigator and of the sponsor with regard to publication of the results of this trial are described in the investigator contract. As a rule, no trial results should be published prior to finalisation of the Clinical Trial Report.

The certificate of insurance cover is made available to the investigator and the patients, and is stored in the ISF.

8.1 TRIAL APPROVAL, PATIENT INFORMATION, INFORMED CONSENT

This trial will be initiated only after all required legal documentation has been reviewed and approved by the respective Institutional Review Board (IRB / Independent Ethics Committee (IEC and competent authority (CA) according to national and international regulations. The same applies for the implementation of changes introduced by amendments.

Prior to patient participation in the trial, written informed consent must be obtained from each patient (or the patient’s legally accepted representative) according to ICH-GCP and to the regulatory and legal requirements of the participating country. Each signature must be personally dated by each signatory and the informed consent and any additional patient-information form retained by the investigator as part of the trial records. A signed copy of the informed consent and any additional patient information must be given to each patient or the patient’s legally accepted representative.”

The patient must be given sufficient time to consider participation in the trial. The investigator or delegate obtains written consent of the patient’s own free will with the informed consent form after confirming that the patient understands the contents. The investigator or his delegate must sign (or place a seal on) and date the informed consent form. If a trial collaborator has given a supplementary explanation, the trial collaborator also signs (or places a seal on) and dates the informed consent.

Proprietary confidential information © 2019 Boehringer Ingelheim International GmbH or one or more of its affiliated companies

Re-consenting may become necessary when new relevant information becomes available and should be conducted according to the sponsor's instructions.

The consent and re-consenting process should be properly documented in the source documentation.

8.2 DATA QUALITY ASSURANCE

A risk-based approach is used for trial quality management. It is initiated by the assessment of critical data and processes for trial subject protection and reliability of the results as well as identification and assessment of associated risks. An Integrated Quality and Risk Management Plan documents the rationale and strategies for risk management during trial conduct including monitoring approaches, vendor management and other processes focusing on areas of greatest risk.

Continuous risk review and assessment may lead to adjustments in trial conduct, trial design or monitoring approaches.

A quality assurance audit/inspection of this trial may be conducted by the sponsor, sponsor's designees, or by IRB / IEC or by regulatory authorities. The quality assurance auditor will have access to all medical records, the investigator's trial-related files and correspondence, and the informed consent documentation of this clinical trial.

8.3 RECORDS

CRFs for individual patients will be provided by the sponsor. See Section 4.1.5.2 for rules about emergency code breaks. For drug accountability, refer to Section 4.1.8.

8.3.1 Source documents

In accordance with regulatory requirements, the investigator should prepare and maintain adequate and accurate source documents and trial records that include all observations and other data pertinent to the investigation on each trial patient. Source data as well as reported data should follow the "Attributable, Legible, Contemporaneous, Original, Accurate (ALCOA) principles" and be attributable, legible, contemporaneous, original and accurate. Changes to the data should be traceable (audit trail).

Data reported on the CRF must be consistent with the source data or the discrepancies must be explained.

The current medical history of the patient may not be sufficient to confirm eligibility for the trial and the investigator may need to request previous medical histories and evidence of any diagnostic tests. In this case, the investigator must make at least one documented attempt to retrieve previous medical records. If this fails, a verbal history from the patient, documented in their medical records, would be acceptable.

If the patient is not compliant with the protocol, any corrective action e.g. re-training must be documented in the patient file.

Proprietary confidential information © 2019 Boehringer Ingelheim International GmbH or one or more of its affiliated companies

For the CRF, data must be derived from source documents, for example:

- Patient identification: gender, year of birth (in accordance with local laws and regulations).
- Patient participation in the trial (substance, trial number, patient number, date patient was informed).
- Dates of patient's visits, including dispensing of trial medication.
- Medical history (including trial indication and concomitant diseases, if applicable).
- Medication history.
- Adverse events and outcome events (onset date (mandatory), and end date (if available)).
- Serious adverse events (onset date (mandatory), and end date (if available)).
- Concomitant therapy (start date, changes).
- Originals or copies of laboratory results and other imaging or testing results, with proper documented medical evaluation (in validated electronic format, if available).
- Supporting documents and medical records for assessment of death and HFEs.
- Completion of patient's participation in the trial" (end date; in case of premature discontinuation document the reason for it).
- Prior to allocation of a patient to a treatment into a clinical trial, there must be documented evidence in the source data (e.g. medical records) that the trial participant meets all inclusion criteria and does not meet any exclusion criteria. The absence of records (either medical records, verbal documented feedback of the patient or testing conducted specific for a protocol) to support inclusion/exclusion criteria does not make the patient eligible for the clinical trial.

8.3.2 Direct access to source data and documents

The investigator /institution will allow site trial-related monitoring, audits, IRB / IEC review and regulatory inspections. Direct access must be provided to the CRF and all source documents/data, including progress notes, copies of laboratory and medical test results, which must be available at all times for review by the CRA, auditor and regulatory inspector (e.g. FDA). They may review all CRFs and informed consents. The accuracy of the data will be verified by direct comparison with the source documents described in Section 8.3.1. The sponsor will also monitor compliance with the protocol and GCP.

8.3.3 Storage period of records

Trial site(s):
The trial site(s) must retain the source and essential documents (including ISF) according to contract or the local requirements valid at the time of the end of the trial (whatever is longer).

Sponsor:
The sponsor must retain the essential documents according to the sponsor's SOPs.

8.4 EXPEDITED REPORTING OF ADVERSE EVENTS

BI is responsible to fulfil their legal and regulatory reporting obligation in accordance with regulatory requirements.

Proprietary confidential information © 2019 Boehringer Ingelheim International GmbH or one or more of its affiliated companies

8.5 STATEMENT OF CONFIDENTIALITY AND PATIENT PRIVACY

Data protection and data security measures are implemented for the collection, storage and processing of patient data in accordance with the principles 7 and 12 of the World Health Organisation (WHO) GCP handbook.
Individual patient data obtained as a result of this trial is considered confidential and disclosure to third parties is prohibited with the following exceptions:
Personalised treatment data may be given to the patient's personal physician or to other appropriate medical personnel responsible for the patient's welfare. Data generated at the site as a result of the trial need to be available for inspection on request by the participating physicians, the sponsor's representatives, by the IRB / IEC and the regulatory authorities.

8.6 TRIAL MILESTONES

The **start of the trial** is defined as the date when the first patient in the whole trial signs informed consent.

The end of the trial is defined as the date of the last visit of the last patient in the whole trial ("Last Patient Completed"). The "**Last Patient Last Treatment"** (LPLT) date is defined as the date on which the last patient in the whole trial is administered the last dose of trial treatment (as scheduled per protocol or prematurely). Individual investigators will be notified of SUSARs occurring with the trial medication until 30 days after LPLT at their site.

Early termination of the trial is defined as the premature termination of the trial due to any reason before the end of the trial as specified in this protocol.

Temporary halt of the trial is defined as any unplanned interruption of the trial by the sponsor with the intention to resume it.

Suspension of the trial is defined as an interruption of the trial based on a Health Authority request.

The IEC / competent authority in each participating EU member state will be notified about the trial milestones according to the respective laws.

A final report of the clinical trial data will be written only after all patients have completed the trial in all countries (EU or non-EU) to incorporate and consider all data in the report.

The sponsor will submit to the EU database a summary of the final trial results within one year from the end of a clinical trial as a whole, regardless of the country of the last patient (EU or non-EU).

8.7 ADMINISTRATIVE STRUCTURE OF THE TRIAL

The trial is sponsored by Boehringer Ingelheim (BI).

Two Coordinating Investigators are responsible to coordinate investigators at the different sites participating in this trial. Tasks and responsibilities are defined in a contract.

Proprietary confidential information © 2019 Boehringer Ingelheim International GmbH or one or more of its affiliated companies

An Executive Committee (ExCom) consisting of independent experts (including the Coordinating Investigators of this trial) and sponsor representatives will be established to support the sponsor in designing the trials and successful execution. The composition of the ExCom will be documented in the Trial Master File (TMF). The tasks and responsibilities will be agreed in contracts between the ExCom members and the Sponsor and also summarised in an ExCom charter. The Executive Committee will assess the baseline characteristics of the patients in an ongoing blinded manner and if needed, may take appropriate steps, which may include restrictions to enrolment for certain subpopulations.

A National Coordinator Committee (NCC) will be established and will consist of the leading expert(s) in each of the participating countries. The NCs will support the Sponsor in the successful execution of the trial. The NCC will have an advisory function in the trial. The tasks and responsibilities will be agreed in contracts between the NCC member and the Sponsor.

A Data Monitoring Committee (DMC) will be established. Members of the DMC are independent of BI, they are physicians experienced in the treatment of the disease under investigation and a statistician. The DMC will evaluate safety data. While DMC members may be unblinded, measures are in place to ensure the blinding for everyone else involved in the trial. Regular DMC meetings will be held at specified intervals. The DMC will recommend continuation, modification or termination of the trial as detailed in the DMC charter. DMC recommendations as well as the final BI decision will be reported to the appropriate Regulatory Authorities (RAs)/ Health Authority (Has), IRBs/ECs, and to investigators as requested by local law. The tasks and responsibilities of the DMC are specified in a charter.

An independent external committee (Clinical Event Committee (CEC)) will be established to adjudicate certain hepatic events and ketoacidosis.

Hepatic External adjudication for hepatic events

Certain hepatic events will be adjudicated by external independent experts for severity and causal relationship with the trial medication; both in a blinded fashion. Events to be reviewed will be defined in a charter.

Events may either be defined by abnormal laboratory values and/or relevant adverse events or both.

For qualifying events, relevant source documents generated from any medical evaluations of these events will be requested including for example laboratory values, histological analysis, reports from ultrasound, computed tomography (CT), magnetic resonance imaging (MRI), scintigraphy, hospital discharge letters, and medical reports from other physicians. All evaluations will be performed in a blinded fashion. The assessments will be analysed based on empagliflozin data combined from multiple trials (i.e. on project level).

Proprietary confidential information © 2019 Boehringer Ingelheim International GmbH or one or more of its affiliated companies

Adjudication of ketoacidosis

Events suspected to be metabolic acidosis, ketoacidosis and DKA will be adjudicated by independent external experts in a blinded fashion. Events to be reviewed will be defined in a charter.

Relevant documentation on the participating (Principal) Investigators (e.g. their curricula vitae) will be filed in the ISF. The investigators will have access to the BI clinical trial portal (Clinergize) to facilitate document exchange.

BI has appointed a Clinical Trial Leader (CT Leader), responsible for coordinating all required activities, in order to

- manage the trial in accordance with applicable regulations and internal SOPs,
- direct the clinical trial team in the preparation, conduct, and reporting of the trial,
- ensure appropriate training and information of CT Managers, CRAs, and investigators of participating countries.

The organisation of the trial in the participating countries will be performed by the respective local or regional BI-organisation (Operating Unit, OPU) in accordance with applicable regulations and BI SOPs, or by a Contract Research Organisation (CRO) with which the responsibilities and tasks will have been agreed and a written contract filed before initiation of the clinical trial.

Data Management and Statistical Evaluation will be done by BI according to BI SOPs.

Tasks and functions assigned in order to organise, manage, and evaluate the trial are defined according to BI SOPs. A list of responsible persons and relevant local information can be found in the ISF.
A central laboratory service and an IRT vendor will be used in this trial. Details will be provided in the IRT Manual and Central Laboratory Manual, available in the ISF.

Proprietary confidential information © 2019 Boehringer Ingelheim International GmbH or one or more of its affiliated companies

9. REFERENCES

9.1 PUBLISHED REFERENCES

P13-04190 Seman L, Macha S, Nehmiz G, Simons G, Ren B, Pinnetti S, et.al. Empagliflozin (BI 10773), a potent and selective SGLT2 inhibitor, induces dose dependent glucosuria in healthy subjects. Clin Pharmacol Drug Dev. 2013. 2(2): 152-161.

P13-06404 Yancy CW, et.al. 2013 ACCF/AHA Guideline for the Management of Heart Failure. Circulation. 2013. 128: e240-e327.

P14-01668 Cherney DZI, Perkins BA, Soleymanlou N, Har R, Fagan N, Johansen OE, et.al. The effect of empagliflozin on arterial stiffness and heart rate variability in subjects with uncomplicated type 1 diabetes mellitus. Cardiovasc Diabetol. 2014. 13(28).

P15-00589 Inzucchi SE, Zinman B, Wanner C, Ferrari R, Fitchett D, Hantel S, et.al. SGLT-2 inhibitors and cardiovascular risk: proposed pathways and review of ongoing outcome trials. Diabetes Vasc Dis Res. 2015. 12(2): 90-100.

P15-09541 Chilton R, Tikkanen I, Cannon CP, Crowe S, Woerle HJ, Broedl UC, et.al. Effects of empagliflozin on blood pressure and markers of arterial stiffness and vascular resistance in patients with type 2 diabetes. Diabetes Obes Metab. 2015. 17(12): 1180-1193.

P15-09840 Zinman B, Wanner C, Lachin JM, Fitchett D, Bluhmki E, Hantel S, et.al. Empagliflozin, cardiovascular outcomes, and mortality in type 2 diabetes. New Engl J Med. 2015. 373(22): 2117-2128.

P16-01253 Fitchett D, Zinman B, Wanner C, Lachin JM, Hantel S, Salsali A, et.al. Heart failure outcomes with empagliflozin in patients with type 2 diabetes at high cardiovascular risk: results of the EMPA-REG OUTCOME trial. Eur Heart J. 2016. 37(19): 1526-1534.

P16-01830 Ferrannini E, Baldi S, Frascerra S, Astiarraga B, Heise T, Bizzotto R, et.al. Shift to fatty substrate utilization in response to sodium-glucose cotransporter 2 inhibition in subjects without diabetes and patients with type 2 diabetes. Diabetes. 2016. 65(5): 1190-1195.

P16-03952 Mozaffarian D, et.al. American Heart Association Statistics Committee and Stroke Statistics Subcommittee. Heart disease and stroke statistics - 2016 update: a report from the American Heart Association. Circulation. 2016. 133(4): e38-e360.

P16-05920 Ponikowski P, Voors AA, Anker SD, Bueno H, Cleland JGF, Coats AJS, et.al. 2016 ESC Guidelines for the diagnosis and treatment of acute and chronic heart failure: the Task Force for the Diagnosis and Treatment of Acute and Chronic Heart Failure of the European Society of Cardiology (ESC), developed with the special contribution of the Heart Failure Association (HFA) of the ESC. Eur Heart J. 2016. 37: 2129-2200.

P17-04479 Al-Jobori H, Daniele G, Cersosimo E, Triplitt C, Mehta R, Norton L, et.al. Empagliflozin and kinetics of renal glucose transport in healthy individuals and individuals with type 2 diabetes. Diabetes. 2017. 66 (7): 1999-2006.

Proprietary confidential information © 2019 Boehringer Ingelheim International GmbH or one or more of its affiliated companies

P17-10453 Wanner C, Lachin JM, Inzucchi SE, Fitchett D, Mattheus M, George J, et.al. Empagliflozin and clinical outcomes in patients with type 2 diabetes mellitus, established cardiovascular disease and chronic kidney disease. Circulation. 2018. 137(2): 119-129.

R16-1528 Owan TE, Redfield MM. Epidemiology of diastolic heart failure. Progress in Cardiovascular Diseases. 2005. 47(5): 320-332.

R16-2217 Cheng RK, Cox M, Neely ML, Heidenreich PA, Bhatt DL, Eapen ZJ, et.al. Outcomes in patients with heart failure with preserved, borderline, and reduced ejection fraction in the Medicare population. Am Heart J. 2014. 168(5): 721 - 730.e3.

R17-1562 Bishu K, Redfield MM. Acute heart failure with preserved ejection fraction: unique patient characteristics and targets for therapy. Curr Heart Fail Rep. 2013. 10(3): 190-197.

R17-2687 Spertus J, Peterson E, Conard MW, Heidenreich PA, Krumholz HM, Jones P, et.al. Cardiovascular Outcomes Research Consortium Monitoring clinical changes in patients with heart failure: a comparison of methods. American Heart Journal. 2005. 150(4): 707-715.

P18-10152 Inzucchi SE, Kosiborod M, Fitchett D, Wanner C, Hehnke U, Kaspers S, et.al. Improvement in cardiovascular outcomes with empagliflozin is independent of glycemic control. Circulation. 2018. 138(17): 1904-1907.

P19-01985 Savarese G, Sattar N, Januzzi J, Verma S, Lund LH, Fitchett D, et.al. Empagliflozin is associated with a lower risk of post-acute heart failure rehospitalization and mortality. Insights from the EMPA-REG OUTCOME trial. Circulation. 2019. 139(11): 1458-1460.

P19-02151 Verma S, McMurray J.J.V. SGLT2 inhibitors and mechanisms of cardiovascular benefit: a state-of-the-art review. Diabetologia. 2018. 61: 2108-2117.

R07-4680 Collett.D, Modelling Survival Data in Medical Research. A CRC Press Company. 2003.

R12-1392 Levey AS, Stevens LA, Schmid CH, Zhang Y, Castro AF, Feldman HI, et.al. Disease Epidemiology Collaboration (CKD-EPI) A new equation to estimate glomerular filtration rate. Ann Intern Med. 2009. 150(9):604-612.

R19-2911 Gattis WA, O'Connor CM, Gallup DS, Hasselblad V, Gheorghiade M., et.al. Predischarge initiation of carvedilol in patients hospitalised for decompensated heart failure: results of the Initiation Management Predischarge: Process for Assessment of Carvedilol Therapy in Heart Failure (IMPACT-HF) trial. J Am Coll Cardiol. 2004. 43(9): 1534–41.

R19-2913 Logeart D, Thabut G, Jourdain P, Chavelas C, et al. Predischarge B-type natriuretic peptide assay for identifying patients at high risk of re-admission after decompensated heart failure. J Am Coll Cardiol. 2004. 43(4): 635-641.

R19-2914 Zile MR, Bennett TB, et.al. Transition from Chronic Compensated to Acute Decompensated Heart Failure: Pathophysiological insight from continuous monitoring of intracardiac Pressures. Circulation. 2008. 118. 1433-1441.

R19-2915 Gheorghiade M, Vaduganathan M, et al. Rehospitalisation for heart failure: problems and perspectives. J Am Coll Cardiol. 2013. 61(4): 391-403.

Proprietary confidential information © 2019 Boehringer Ingelheim International GmbH or one or more of its affiliated companies

R19-2916	Torre-Amione G, Milo-Cotter O, Kaluski E, Perchenet L, Kobrin I, Frey A, et.al. Early worsening heart failure in patients admitted for acute heart failure: time course, hemodynamic predictors, and outcome. J Card Fail. 2009. 15(8): 639–644.
R19-2917	Heidenreich PA, et al. Forecasting the Impact of Heart Failure in the United States: A Policy Statement From the American Heart Association. Circ Heart Fail. 2013. 6: 606-619.
R19-2918	Blecker S, Paul M, Taksler G, Ogedegbe G, Katz S. Heart failure associated hospitalisations in the United States. J Am Coll Cardiol. 2013. 61(12): 1259-1267.
R19-2919	Butler J, Arbogast PG, Daugherty J, Jain MK, Ray WA, Griffin MR. Outpatient utilization of angiotensin-converting enzyme inhibitors among heart failure patients after hospital discharge. J Am Coll Cardiol. 2004. 43(11): 2036-2043.
R19-2920	Fonarow GC, Gawlinski A, Moughrabi S, Tillisch JH. Improved treatment of coronary heart disease by implementation of a Cardiac Hospitalisation Atherosclerosis Management Program (CHAMP). Am J Cardiol. 2001. 87(7): 819-822.
R19-2921	Grodin JL, Liebo MJ, et.al. Prognostic Implications of Changes in Amino-Terminal Pro-B-Type Natriuretic Peptide in Acute Decompensated Heart Failure: Insights from ASCEND-HF. Journal of Cardiac Failure. 2019. 00(00): 1-9.
R19-2924	Aditi A, Bhagat, et.al. Initiation, Continuation, Switching, and Withdrawal of Heart Failure Medical Therapies During Hospitalisation. JACC:HF. 2019. 7(1): 1-12.
R19-2932	Velazquez EJ, Morrow DA, DeVore AD, Duffy CI, Ambrosy AP, McCague K, et.al. PIONEER-HF Investigators. Angiotensin-neprilysin inhibition in acute decompensated heart failure. N Engl J Med. 2019. 380(6): 539-548.
R19-3040	Heidenreich PA. Patient-Reported Outcomes: The Future of Heart Failure Care. JACC HF. 2019. 7: 1-3.
R19-3043	Buyse M. Generalized pairwise comparisons for prioritized outcomes in the two-sample problem. Statistics in Medicine. 2010. 29(30): 3245-3257.
R19-3044	Schou M, Gustafsson F, Kjaer A, Hildebrandt PM. Long-term clinical variation of NT-proBNP in stable chronic heart failure patients. European Heart Journal. 2007. 28(2): 177-182.
R19-3046	Buyse M. Generalized Pairwise Comparisons. Wiley StatsRef: Statistics Reference Online. 2019. 99: 1-9.
R19-3124	Nassif ME, Windsor S, Tang F, Khariton Y, Husain M, Inzucchi S, et.al. Dapagliflozin Effects on Biomarkers, Symptoms, and Functional Status in Patients With Heart Failure With Reduced Ejection Fraction: The DEFINE-HF Trial. Circulation. 2019. 40(140): 1-14.
R19-3125	McMurray JJV, Solomon SD, Inzucchi L, et.al. Dapagliflozin with Patients in Heart Failure and Reduced Ejection Fraction. New England Journal of Medicine. 2019. 1-13.

Proprietary confidential information © 2019 Boehringer Ingelheim International GmbH or one or more of its affiliated companies

R19-3126 U.S. Department of Health and Human Services, Food and Drug Administration, Center for Drug Evaluation and Research, Center for Biologics Evaluation and Research. Treatment for Heart Failure: Endpoints for Drug Development Guidance for Industry. June 2019.

R98-1473 Van Elterens. On the combination of independent two sample tests of Wilcoxon. Bull Inst Int Stat. 1960. 37(3): 351-361.

R19-3245 Hicks KA, Mahaffey KW, Mehran R, Nissen SE, Wiviott SD, et.al. 2017 Cardiovascular and Stroke Endpoint Definitions for Clinical Trials. Journal of the American College of Cardiology. 2018. 71(9): 1021-34.

9.2 UNPUBLISHED REFERENCES

c02695839-01 Devins T, Espadero RM, Frampton H, Koeppen M, Mattheus M, Joseph D. Clinical Trial Report: Empagliflozin (BI 10773). The EMPA-REG OUTCOME® Trial 1245.25.

c01678844 Investigators Brochure. Empagliflozin. Current Version.

c11963611-01 Lewis-D'Agostino D, Fagan N, Wu J, Stanley J. Clinical Trial Report: Jardiance® (empagliflozin). An open-label, mechanistic study to examine the effect of oral empagliflozin (25 mg q.d.) on kinetics of renal glucose reabsorption in patients with type 2 diabetes mellitus and healthy controls. Trial 1245.66.

Proprietary confidential information © 2019 Boehringer Ingelheim International GmbH or one or more of its affiliated companies

10. APPENDICES

10.1 KCCQ (KANSAS CITY CARDIOMYOPATHY QUESTIONNAIRE)

THE KANSAS CITY CARDIOMYOPATHY QUESTIONNAIRE:

The following questions refer to your **heart failure** and how it may affect your life. Please read and complete the following questions. There are no right or wrong answers. Please mark the answer that best applies to you.

1. **Heart failure** affects different people in different ways. Some feel shortness of breath while others feel fatigue. Please indicate how much you are limited by **heart failure** (shortness of breath or fatigue) in your ability to do the following activities over the past 2 weeks.

Place an X in one box on each line

Activity	Extremely Limited	Quite a bit Limited	Moderately Limited	Slightly Limited	Not at all Limited	Limited for other reasons or did not do the activity
Dressing yourself	☐	☐	☐	☐	☐	☐
Showering/Bathing	☐	☐	☐	☐	☐	☐
Walking 1 block on level ground	☐	☐	☐	☐	☐	☐
Doing yardwork, housework or carrying groceries	☐	☐	☐	☐	☐	☐
Climbing a flight of stairs without stopping	☐	☐	☐	☐	☐	☐
Hurrying or jogging (as if to catch a bus)	☐	☐	☐	☐	☐	☐

2. Compared with 2 weeks ago, have your symptoms of **heart failure** (shortness of breath, fatigue or ankle swelling) changed?
My symptoms of **heart failure** have become . . .

Much worse	Slightly worse	Not changed	Slightly better	Much better	I've had no symptoms over the last 2 weeks
☐	☐	☐	☐	☐	☐

3. Over the past 2 weeks, how many times did you have **swelling** in your feet, ankles or legs when you woke up in the morning?

Every morning	3 or more times a week, but not every day	1–2 times a week	Less than once a week	Never over the past 2 weeks
☐	☐	☐	☐	☐

4. Over the past 2 weeks, how much has **swelling** in your feet, ankles or legs bothered you?
It has been . . .

**Extremely** bothersome	**Quite a bit** bothersome	**Moderately** bothersome	**Slightly** bothersome	**Not at all** bothersome	I've had **no swelling**
☐	☐	☐	☐	☐	☐

5. Over the past 2 weeks, on average, how many times has **fatigue** limited your ability to do what you want?

All of the time	Several times per day	At least once a day	3 or more times per week but not every day	1–2 times per week	Less than once a week	Never over the past 2 weeks
☐	☐	☐	☐	☐	☐	☐

6. Over the past 2 weeks, how much has your **fatigue** bothered you?
It has been . . .

**Extremely** bothersome	**Quite a bit** bothersome	**Moderately** bothersome	**Slightly** bothersome	**Not at all** bothersome	I've had **no fatigue**
☐	☐	☐	☐	☐	☐

Proprietary confidential information © 2019 Boehringer Ingelheim International GmbH or one or more of its affiliated companies

7. Over the past 2 weeks, on average, how many times has **shortness of breath** limited your ability to do what you wanted?

All of the time	Several times per day	At least once a day	3 or more times per week but not every day	1–2 times per week	Less than once a week	Never over the past 2 weeks
❑	❑	❑	❑	❑	❑	❑

8. Over the past 2 weeks, how much has your **shortness of breath** bothered you?
It has been . . .

**Extremely** bothersome	**Quite a bit** bothersome	**Moderately** bothersome	**Slightly** bothersome	**Not at all** bothersome	I've had **no shortness of breath**
❑	❑	❑	❑	❑	❑

9. Over the past 2 weeks, on average, how many times have you been forced to sleep sitting up in a chair or with at least 3 pillows to prop you up because of **shortness of breath**?

Every night	3 or more times a week, but not every day	1–2 times a week	Less than once a week	Never over the past 2 weeks
❑	❑	❑	❑	❑

10. **Heart failure** symptoms can worsen for a number of reasons. How sure are you that you know what to do, or whom to call, if your **heart failure** gets worse?

**Not at all** sure	**Not very** sure	**Somewhat** sure	**Mostly** sure	**Completely** sure
❑	❑	❑	❑	❑

11. How well do you understand what things you are able to do to keep your **heart failure** symptoms from getting worse? (for example, weighing yourself, eating a low salt diet, etc.)

Do not understand at all	Do not understand very well	Somewhat understand	Mostly understand	Completely understand
❑	❑	❑	❑	❑

12. Over the past 2 weeks, how much has your **heart failure** limited your enjoyment of life?

It has **extremely** limited my enjoyment of life	It has limited my enjoyment of life **quite a bit**	It has **moderately** limited my enjoyment of life	It has **slightly** limited my enjoyment of life	It has **not limited** my enjoyment of life at all
❑	❑	❑	❑	❑

13. If you had to spend the rest of your life with your **heart failure** the way it is right now, how would you feel about this?

Not at all satisfied	Mostly dissatisfied	Somewhat satisfied	Mostly satisfied	Completely satisfied
❑	❑	❑	❑	❑

14. Over the past 2 weeks, how often have you felt discouraged or down in the dumps because of your **heart failure**?

I felt that way **all of the time**	I felt that way **most of the time**	I **occasionally** felt that way	I **rarely** felt that way	I **never** felt that way
❑	❑	❑	❑	❑

15. How much does your **heart failure** affect your lifestyle? Please indicate how your **heart failure** may have limited your participation in the following activities over the past 2 weeks.

Please place an **X** in one box on each line

**Activity**	**Severely** limited	Limited **quite a bit**	**Moderately** limited	**Slightly** limited	**Did not** limit at all	Does not apply or did not do for other reasons
Hobbies, recreational activities	❑	❑	❑	❑	❑	❑
Working or doing household chores	❑	❑	❑	❑	❑	❑
Visiting family or friends out of your home	❑	❑	❑	❑	❑	❑
Intimate relationships with loved ones	❑	❑	❑	❑	❑	❑

Proprietary confidential information © 2019 Boehringer Ingelheim International GmbH or one or more of its affiliated companies

10.2 INCLUSION OF ILLITERATE PATIENTS - KCCQ

In the event of recruiting an illiterate patient, the following process should be followed with respect to completion of the self-reported PRO:

- At each visit where the administration of the Patient Reported Outcome form is required, the trial coordinator or designated site personnel will read each of the items on the questionnaire to the patient, word for word, and without any accompanying explanation.

- The questionnaires will be provided to patients in the language or local dialect that is understood by the patient using the different language versions of the questionnaire that are part of the eCRF for the trial.

- The patient will choose the most appropriate response to the question, and indicate the response on the questionnaire by him/herself. If this is not possible, the trial coordinator or designated site personnel or patient's caregiver will indicate the response on the questionnaire based on the patient's feedback.

In the same way as for all other patients, the completion of the questionnaires should be performed in a quiet area where the patient can consider his/her responses.

Proprietary confidential information © 2019 Boehringer Ingelheim International GmbH or one or more of its affiliated companies

11. DESCRIPTION OF GLOBAL AMENDMENT(S)

This is the original protocol.

APPROVAL / SIGNATURE PAGE

Document Number: c28603082 **Technical Version Number:1.0**

Document Name: clinical-trial-protocol-version-01

Title: A multicentre, randomised, double-blind, 90-day superiority trial to evaluate the effect on clinical benefit, safety and tolerability of once daily oral EMPagliflozin 10 mg compared to placebo, initiated in patients hospitalised for acUte heart faiLure (de novo or decompensatied chronic HF) who have been StabilisEd (EMPULSE).

Signatures (obtained electronically)

Meaning of Signature	Signed by	Date Signed
Author-Clinical Trial Leader	[redacted]	09 Oct 2019 15:36 CEST
Approval-Team Member Medicine		09 Oct 2019 16:34 CEST
Author-Trial Statistician		10 Oct 2019 10:11 CEST
Approval-Therapeutic Area Head		11 Oct 2019 10:45 CEST
Verification-Paper Signature Completion		16 Oct 2019 08:35 CEST

(Continued) Signatures (obtained electronically)

Meaning of Signature	Signed by	Date Signed

Clinical Trial Protocol

	**Document Number:**	**c28603082-02**
**EudraCT No.**	**2019-002946-19**	
**BI Trial No.**	1245-0204	
**BI Investigational Medicinal Product(s)**	Jardiance®, empagliflozin	
**Title**	A multicentre, randomised, double-blind, 90-day superiority trial to evaluate the effect on clinical benefit, safety and tolerability of once daily oral **EMP**agliflozin 10 mg compared to placebo, initiated in patients hospitalised for ac**U**te heart fai**L**ure (de novo or decompensated chronic HF) who have been **S**tabilis**E**d (**EMPULSE**)	
**Lay Title**	A study to test the effect of empagliflozin in patients who are in hospital for acute heart failure.	
**Clinical Phase**	III	
**Clinical Trial Leader**	[redacted]	
**Coordinating Investigators**	[redacted]	
**Status**	Final Protocol (Revised Protocol (based on global amendment 1))	
**Version and Date**	**Version:** 2.0	**Date:** 04 Jun 2020

Proprietary confidential information.
© 2020 Boehringer Ingelheim International GmbH or one or more of its affiliated companies. All rights reserved.
This document may not - in full or in part - be passed on, reproduced, published or otherwise used without prior written permission.

001-MCS-40-106_RD-03 (17.0) / Saved on 18 Mar 2019

Proprietary confidential information © 2020 Boehringer Ingelheim International GmbH or one or more of its affiliated companies

CLINICAL TRIAL PROTOCOL SYNOPSIS

**Company name**	Boehringer Ingelheim
**Protocol date**	09 Oct 2019
**Revision date**	04 Jun 2020
**BI trial number**	1245-0204
**Title of trial**	A multicentre, randomised, double-blind, 90-day superiority trial to evaluate the effect on clinical benefit, safety and tolerability of once daily oral **EMP**agliflozin 10 mg compared to placebo, initiated in patients hospitalised for ac**U**te heart fai**L**ure (de novo or decompensated chronic HF) who have been **S**tabilis**E**d (**EMPULSE**)
**Coordinating Investigators**	[redacted]
**Trial site(s)**	Multi-centre trial
**Clinical phase**	III
**Trial rationale**	Other trials with empagliflozin are performed to assess mortality and morbidity, functional capacity and safety of empagliflozin in patients with chronic heart failure under stable conditions (i.e. after hospital discharge).<br>Due to its mode of action (MOA), empagliflozin is expected to potentially alleviate congestive symptoms seen in patients shortly after initial stabilisation of acute cardiac decompensation helping to improve heart failure (HF)-related outcomes within several weeks after discharge from hospital.<br>In-hospital initiation of different therapies is one of the best predictors of long-term adherence to medications and long-term improved prognosis.
**Trial objective(s)**	The main objective of this study is to assess whether in-hospital administration of empagliflozin results in improvement in HF-related outcomes (clinical benefit including death, heart failure events (HFE) and the burden of symptoms as assessed by the Kansas City Cardiomyopathy Questionnaire (KCCQ) total symptom score) in patients hospitalised for acute heart failure (de novo or decompensated

Proprietary confidential information © 2020 Boehringer Ingelheim International GmbH or one or more of its affiliated companies

	chronic HF) and after initial stabilisation.<br>Secondary objectives are to further assess whether it is safe to start empagliflozin in patients admitted to hospital in this setting.
**Trial endpoints**	Primary Endpoint:<br>• Clinical benefit, a composite of death, number of heart failure events (HFEs) (including hospitalisations for heart failure (HHFs), urgent heart failure visits and unplanned outpatient visits), time to first HFE and change from baseline in Kansas City Cardiomyopathy Questionnaire - Total Symptom Score (KCCQ-TSS) after 90 days of treatment assessed by the win ratio.<br>Secondary Endpoints:<br>• Improvement in KCCQ-TSS of ≥ 10 points after 90 days of treatment<br>• Change from baseline in KCCQ-TSS after 90 days of treatment<br>• Change from baseline in log-transformed N-Terminal Pro-Brain Natriuretic Peptide (NT-proBNP) level over 30 days of treatment (area under the curve (AUC)).<br>• Days alive and out of hospital from study drug initiation until 30 days after initial hospital discharge<br>• Days alive and out of hospital from study drug initiation until 90 days after randomisation<br>• Time to first occurrence of cardiovascular (CV) death or HFE until end of trial visit<br>• Occurrence of HHF until 30 days after initial hospital discharge<br>• Occurrence of chronic dialysis or renal transplant or sustained reduction of ≥40% estimated glomerular filtration rate (eGFR) Chronic Kidney Disease Epidemiology Collaboration Equation ((CKD-EPI)cr), or<br>o sustained eGFR (CKD-EPI)cr <15 mL/min/1.73 m^2 for patients with baseline eGFR ≥30 mL/min/1.73 m^2<br>o sustained eGFR (CKD-EPI)cr <10 mL/min/1.73 m^2 for patients with baseline eGFR <30 mL/min/1.73 m^2<br>• Diuretic effect as assessed by weight loss per mean daily loop diuretic dose after 15 days of treatment<br>• Diuretic effect as assessed by weight loss per mean daily loop diuretic dose after 30 days of treatment
**Trial design**	Randomised, double-blind, parallel-group, placebo controlled, multinational and multicentre study
**Total number of patients randomised**	Approximately 500
**Number of patients on each treatment**	Approximately 250
**Diagnosis**	Patients admitted to hospital for treatment of acute HF (de novo or decompensated chronic HF). Patients should be randomised after at least 24 hours and no later than 5 days after hospital admission. Randomisation should occur as soon as the patient is stabilised (see inclusion criterion 7).

Proprietary confidential information © 2020 Boehringer Ingelheim International GmbH or one or more of its affiliated companies

**Main in- and exclusion criteria**	Main Inclusion Criteria:<br>• Currently hospitalised for the primary diagnosis of acute heart failure (de novo or decompensated chronic HF), regardless of ejection fraction (EF). Patients with a diagnosis of hospitalised heart failure must have HF symptoms at the time of hospital admission<br>• Evidence of left ventricular ejection fraction (LVEF, either reduced or preserved EF) as per local reading preferably measured during current hospitalisation or in the 12 months prior to randomisation<br>• Patients must be randomised after at least 24 hours and no later than 5 days after admission, as early as possible after stabilisation and while still in hospital<br>• Patients must fulfil the following stabilisation criteria (while in the hospital):<br>- SBP ≥100mm Hg and no symptoms of hypotension in the preceding 6 hours,<br>- no increase in i.v. diuretic dose for 6 hours prior to randomisation,<br>- no i.v. vasodilators including nitrates within the last 6 hours prior to randomisation<br>- no i.v. inotropic drugs for 24 hours prior to randomisation.<br>• Elevated NT-proBNP ≥ 1600pg/mL or BNP ≥400 pg/mL according to the local lab, for patients without atrial fibrillation (AF); or elevated NT-proBNP ≥ 2400pg/mL or BNP ≥600 pg/mL for patients with AF, measured during the current hospitalisation or in the 72 hours prior to hospital admission,. For patients treated with an angiotensin receptor neprilysin inhibitor (ARNI) in the previous 4 weeks prior to randomisation, only NT-proBNP values should be used<br>• HF episode leading to hospitalisation must have been treated with a minimum dose of 40 mg of i.v. furosemide (or equivalent i.v. loop diuretic defined as 20 mg of torasemide or 1 mg of bumetanide)<br><br>Main Exclusion Criteria:<br>• Cardiogenic shock<br>• Current hospitalisation for acute heart failure primarily triggered by pulmonary embolism, cerebrovascular accident, or acute myocardial infarction (AMI)<br>• Current hospitalisation for acute heart failure not caused primarily by intravascular volume overload;<br>• Below interventions in the past 30 days prior to randomisation or planned during the study:<br>- Major cardiac surgery, or TAVI (Transcatheter Aortic Valve Implantation), or PCI, or Mitraclip<br>- All other surgeries that are considered major according to

Proprietary confidential information © 2020 Boehringer Ingelheim International GmbH or one or more of its affiliated companies

	investigator judgement<br>- Implantation of cardiac resynchronisation therapy (CRT) device<br>- cardiac mechanical support implantation<br>- Carotid artery disease revascularisation (stent or surgery)<br>• Acute coronary syndrome / myocardial infarction, stroke or transient ischemic attack (TIA) in the past 90 days prior to randomisation<br>• Heart transplant recipient, or listed for heart transplant with expectation to receive a transplant during the course of this trial (according to investigator judgement), or planned for palliative care for HF, or currently using left ventricular assist device (LVAD) or intra-aortic balloon pump (IABP) or any other type of mechanical circulatory support, or patients on mechanical ventilation, or patients with planned inotropic support in an out-patient setting<br>• Haemodynamically significant (severe) uncorrected primary cardiac valvular disease planned for surgery or intervention during the course of the study (note: secondary mitral regurgitation or tricuspid regurgitation due to dilated cardiomyopathy is not excluded unless planned for surgery or intervention during the course of the study)<br>• Impaired renal function, defined as eGFR < 20 mL/min/1.73 m^2 as measured during hospitalisation (latest local lab measurement before randomisation) or requiring dialysis<br>• Type 1 Diabetes Mellitus (T1DM)<br>• History of ketoacidosis, including diabetic ketoacidosis (DKA)
**Test product(s)**	Empagliflozin
**dose**	10 mg q.d.
**mode of administration**	Oral (p.o.)
**Comparator product(s)**	Placebo
**dose**	Not applicable
**mode of administration**	Oral (p.o.)
**Duration of treatment**	90 days
**Statistical methods**	The primary endpoint is clinical benefit (as defined in Section 7.2.2) assessed by the win ratio. The statistical model will be a non-parametric generalised pairwise comparison within HF status strata. The variance of the win ratio will be calculated using the asymptotic normal U statistics approach.

Proprietary confidential information © 2020 Boehringer Ingelheim International GmbH or one or more of its affiliated companies

FLOW CHART

**Trial Periods**	**Screen**	**Randomised Treatment**							**Follow-up**
**Visit**	**1**	**2 (in hospital)**			**3**	**4**	**5 (EoT)**	**Early Discon-tinuation Visit[3]**	**6 (FUP) (Phone Call)**
		**a**	**b[2]**	**c[2]**					
**Days**	-4 to 1	1[1]	3	5	15	30	90		EoT + 7 days
Time window for visits (days)		None	±1	±1	±3	±3	±7		+ 7
Informed consent[4]	X								
Demographics	X								
Medical history	X								
Physical examination	X						X	X	
Review of in-/exclusion criteria[8]	X	X							
Randomisation (IRT)		X							
Weight		X			X	X	X	X	
Height		X							
Vital signs[5]		X	X	X	X	X	X	X	
Safety lab: blood tests[6]		X	X	X	X	X	X	X	
Safety lab: urinalysis including ketones[6]		X			X	X	X	X	
Urine Pregnancy test[7]	X						X[12]	X	
HbA1c[6]		X					X	X	
eGFR (CKD-EPI)cr formula[6]		X	X	X	X	X	X	X	
NT-proBNP (central lab)[6,8]		X	X	X	X	X	X	X	
12 lead-ECG[9]		X					X[12]	X	
KCCQ		X			X	X	X	X	
Patient Global Impression of Severity of HF Symptoms		X			X	X	X	X	
Clinical Congestion Score		X	X	X	X		X	X	
NYHA Classification		X			X	X	X	X	
HCRU		X			X	X	X	X	
All AEs/SAEs/AESIs[10]	X	X	X	X	X	X	X	X	X
Concomitant therapy		X	X	X	X	X	X	X	
Dispense trial medication		X				X			
Compliance check					X	X	X	X	
Return medication						X	X	X	
Completion of patient participation (End of Study (EOS))							(X)[11]		X

1 Day of Randomisation / Day of first intake of randomised medication. All Visit 2a assessments must be performed before the first dose is taken in hospital. Assessments from Visit 2a will be used as baseline values.

2 Visit 2b and 2c will only be performed, if the patient is still in hospital.

Proprietary confidential information © 2020 Boehringer Ingelheim International GmbH or one or more of its affiliated companies

[3] Patients who discontinue trial treatment prematurely should undergo the Early Discontinuation Visit as soon as possible and the Follow-up (FUP) Visit 7 days thereafter. Patients should be followed up until Visit 5 (day 90) according to their planned visit schedule.

[4] Written informed consent must be obtained before any other assessment is performed.

[5] Blood pressure and heart rate will be measured with the patient seated and rested for at least 5 minutes. Vital signs at Visit 2a need to be checked before first trial medication intake.

[6] Samples for central lab (including NT-proBNP) at Visit 2a need to be taken before first trial medication intake.

[7] Women of childbearing potential only.

[8] For patient's eligibility, local lab should be used, including a locally performed BNP or NTproBNP result taken during the current hospitalisation or in the 72 hours prior hospital admission. At the time the local BNP or NT-proBNP sample is collected, the baseline rhythm (e.g. sinus rhythm, AF) of the patient must be clarified using ECG or other measures (for example continuous heart monitoring) and documented.

[9] In addition to the scheduled ECGs, ECGs should be done and documented at any time of clinical event (e.g. acute event of arrhythmia, tachy- or bradycardia, angina, or MI).

[10] After the individual patient's end of the trial the investigator should only report any occurrence of cancer, related SAEs and related AESIs of which the investigator may become aware of and only via the SAE form, please see Section 5.2.7.2.1.

[11] Patients who prematurely discontinue medication are followed up according to protocol, and their visit schedule will end at their scheduled Visit 5.

[12] Urinary pregnancy test and ECG do not need to be repeated if patient prematurely discontinued trial medication and these assessments were done at the Early Discontinuation Visit.

For potential modifications of trial conduct in case of restrictions due to COVID-19, please refer to Sections 4.1.4, 6.1, 8.1 and 10.5.

Proprietary confidential information © 2020 Boehringer Ingelheim International GmbH or one or more of its affiliated companies

TABLE OF CONTENTS

TITLE PAGE 1

CLINICAL TRIAL PROTOCOL SYNOPSIS 2

FLOW CHART 6

TABLE OF CONTENTS 8

ABBREVIATIONS 12

1. INTRODUCTION 16

1.1 MEDICAL BACKGROUND 16
1.2 DRUG PROFILE 17
1.3 RATIONALE FOR PERFORMING THE TRIAL 18
1.4 BENEFIT - RISK ASSESSMENT 20
1.4.1 Benefits 20
1.4.2 Risks 21
1.4.3 Discussion 25

2. TRIAL OBJECTIVES AND ENDPOINTS 26

2.1 MAIN OBJECTIVES, PRIMARY AND SECONDARY ENDPOINTS 26
2.1.1 Main objectives 26
2.1.2 Primary endpoint(s) 26
2.1.3 Secondary endpoint(s) 26
2.2 FURTHER OBJECTIVES AND FURTHER ENDPOINTS 27
2.2.1 Further objectives 27
2.2.2 Further endpoints 27

3. DESCRIPTION OF DESIGN AND TRIAL POPULATION 29

3.1 OVERALL TRIAL DESIGN 29
3.2 DISCUSSION OF TRIAL DESIGN, INCLUDING THE CHOICE OF CONTROL GROUP(S) 30
3.3 SELECTION OF TRIAL POPULATION 31
3.3.1 Main diagnosis for trial entry 32
3.3.2 Inclusion criteria 32
3.3.3 Exclusion criteria 34
3.3.4 Withdrawal of patients from treatment or assessments 36
3.3.4.1 Discontinuation of trial treatment 37
3.3.4.2 Withdrawal of consent to trial participation 37
3.3.4.3 Discontinuation of the trial by the sponsor 38

4. TREATMENTS 39

4.1 INVESTIGATIONAL TREATMENTS 39
4.1.1 Identity of the Investigational Medicinal Products 39
4.1.2 Selection of doses in the trial and dose modifications 40
4.1.3 Method of assigning patients to treatment groups 40
4.1.4 Drug assignment and administration of doses for each patient 41
4.1.5 Blinding and procedures for unblinding 41

Proprietary confidential information © 2020 Boehringer Ingelheim International GmbH or one or more of its affiliated companies

4.1.5.1 Blinding ... 41
4.1.5.2 Unblinding and breaking the code ... 41
4.1.6 Packaging, labelling, and re-supply ... 42
4.1.7 Storage conditions ... 42
4.1.8 Drug accountability ... 42
4.2 OTHER TREATMENTS, EMERGENCY PROCEDURES, RESTRICTIONS ... 43
4.2.1 Other treatments and emergency procedures ... 43
4.2.2 Restrictions ... 44
4.2.2.1 Restrictions regarding concomitant treatment ... 44
4.2.2.2 Restrictions on diet and life style ... 45
4.2.2.3 Contraception requirements ... 45
4.3 TREATMENT COMPLIANCE ... 45

5. ASSESSMENTS ... 46

5.1 ASSESSMENT OF EFFICACY ... 46
5.1.1 Clinical Benefit ... 46
5.1.2 NT-proBNP and BNP ... 46
5.1.3 Clinical Congestion Score ... 46
5.1.4 New York Heart Association classification ... 47
5.1.5 Patient Global Impression of Severity of Heart Failure Symptoms ... 48
5.2 ASSESSMENT OF SAFETY ... 48
5.2.1 Physical examination ... 48
5.2.2 Vital signs ... 48
5.2.3 Body weight and height ... 49
5.2.4 Safety laboratory parameters ... 49
5.2.4.1 Renal Function ... 50
5.2.4.2 Pregnancy Testing ... 51
5.2.4.3 Criteria for hypoglycaemic events ... 51
5.2.4.4 Urinary tract infection and genital infections ... 51
5.2.5 Electrocardiogram ... 51
5.2.6 Other safety parameters ... 51
5.2.7 Assessment of adverse events ... 52
5.2.7.1 Definitions of AEs ... 52
5.2.7.1.1 Adverse event ... 52
5.2.7.1.2 Serious adverse event ... 52
5.2.7.1.3 AEs considered “Always Serious” ... 52
5.2.7.1.4 Adverse events of special interest ... 53
5.2.7.1.5 Intensity (severity) of AEs ... 54
5.2.7.1.6 Causal relationship of AEs ... 54
5.2.7.2 Adverse event collection and reporting ... 55
5.2.7.2.1 AE Collection ... 55
5.2.7.2.2 AE reporting to the sponsor and timelines ... 55
5.2.7.2.3 Pregnancy ... 56
5.3 DRUG CONCENTRATION MEASUREMENTS AND PHARMACOKINETICS ... 56
5.4 ASSESSMENT OF BIOMARKER(S) ... 56

Proprietary confidential information © 2020 Boehringer Ingelheim International GmbH or one or more of its affiliated companies

5.5 BIOBANKING ... 56
5.6 OTHER ASSESSMENTS ... 57
5.6.1 Health Care Resource Utilisation (HCRU) ... 57
5.7 APPROPRIATENESS OF MEASUREMENTS ... 57
6. INVESTIGATIONAL PLAN ... 58
6.1 VISIT SCHEDULE ... 58
6.2 DETAILS OF TRIAL PROCEDURES AT SELECTED VISITS ... 58
6.2.1 Screening and run-in period(s) ... 58
6.2.2 Treatment period(s) ... 59
6.2.3 Follow-up period and trial completion ... 60
7. STATISTICAL METHODS AND DETERMINATION OF SAMPLE SIZE ... 61
7.1 NULL AND ALTERNATIVE HYPOTHESES ... 61
7.2 PLANNED ANALYSES ... 61
7.2.1 General considerations ... 61
7.2.2 Primary endpoint analyses ... 62
7.2.3 Secondary endpoint analyses ... 63
7.2.4 Further endpoint analyses ... 64
7.2.5 Safety analyses ... 64
7.2.6 Interim Analyses ... 65
7.3 HANDLING OF MISSING DATA ... 65
7.4 RANDOMISATION ... 65
7.5 DETERMINATION OF SAMPLE SIZE ... 65
8. INFORMED CONSENT, TRIAL RECORDS, DATA PROTECTION, PUBLICATION POLICY, AND ADMINISTRATIVE STRUCTURE ... 67
8.1 TRIAL APPROVAL, PATIENT INFORMATION, INFORMED CONSENT ... 67
8.2 DATA QUALITY ASSURANCE ... 68
8.3 RECORDS ... 68
8.3.1 Source documents ... 68
8.3.2 Direct access to source data and documents ... 69
8.3.3 Storage period of records ... 69
8.4 EXPEDITED REPORTING OF ADVERSE EVENTS ... 70
8.5 STATEMENT OF CONFIDENTIALITY AND PATIENT PRIVACY ... 70
8.6 TRIAL MILESTONES ... 70
8.7 ADMINISTRATIVE STRUCTURE OF THE TRIAL ... 71
9. REFERENCES ... 73
9.1 PUBLISHED REFERENCES ... 73
9.2 UNPUBLISHED REFERENCES ... 76
10. APPENDICES ... 77
10.1 KCCQ (KANSAS CITY CARDIOMYOPATHY QUESTIONNAIRE) ... 77
10.2 INCLUSION OF ILLITERATE PATIENTS - KCCQ ... 79

Proprietary confidential information © 2020 Boehringer Ingelheim International GmbH or one or more of its affiliated companies

10.3 PATIENT GLOBAL IMPRESSION OF SEVERITY OF HEART FAILURE SYMPTOMS: SHORTNESS OF BREATH, FATIGUE AND SWELLING 79
10.4 ACCEPTED FORMS OF CONTRACEPTION FOR PATIENTS IN ITALY 79
10.5 POTENTIAL MODIFICATION OF TRIAL CONDUCT IN CASE OF RESTRICTIONS DUE TO COVID-19 79
11. DESCRIPTION OF GLOBAL AMENDMENT(S) 82
11.1 GLOBAL AMENDMENT 1 82

Proprietary confidential information © 2020 Boehringer Ingelheim International GmbH or one or more of its affiliated companies

ABBREVIATIONS

AE	Adverse Event
AESI	Adverse Event of Special Interest
AF	Atrial Fibrillation
ALCOA	Attributable, Legible, Contemporaneous, Original, Accurate
ALT	Alanine Aminotransferase
AMI	Acute myocardial infarction
ANCOVA	Analysis of Covariance
ARNI	Angiotensin Receptor Neprilysin Inhibitor
AST	Aspartate Aminotransferase
AUC	Area under the Curve
BI	Boehringer Ingelheim
BNP	Brain Natriuretic Peptide
BUN	Blood Urea Nitrogen
CA	Competent Authority
CEC	Clinical Event Committee
CHF	Chronic Heart Failure
CK	Creatine Kinase
CKD-EPIcr	Chronic Kidney Disease Epidemiology Collaboration Equation
COPD	Chronic Obstructive Pulmonary Disease
CRA	Clinical Research Associate
CRF	Case Report Form, paper or electronic (sometimes referred to as “eCRF”)
CRO	Contract Research Organisation
CRT	Cardiac resynchronisation therapy
CT	Computed Tomography
CT Leader	Clinical Trial Leader
CT Manager	Clinical Trial Manager
CTP	Clinical Trial Protocol
CV	Cardiovascular
DILI	Drug Induced Liver Injury
DKA	Diabetic Ketoacidosis
DM	Diabetes Mellitus

Proprietary confidential information © 2020 Boehringer Ingelheim International GmbH or one or more of its affiliated companies

DMC	Data Monitoring Committee
DBP	Diastolic Blood Pressure
EC	Ethics Committee
ECG	Electrocardiogram
eCRF	Electronic Case Report Form
eDC	Electronic Data Capture
EF	Ejection Fraction
EOS	End of Study
eGFR	Estimated Glomerular Filtration Rate
EMPA-REG	Empagliflozin – Reducing Excess Glucose
EoT	End of Treatment
EU	European Union
EudraCT	European Clinical Trials Database
ExCom	Executive Committee
FDA	Food and Drug Administration
FUP	Follow-up
γ-GT	Gamma-Glutamyl Transferase
GCP	Good Clinical Practice
GI	Gastrointestinal
GMP	Good Manufacturing Practice
HA	Health Authority
Hb	Haemoglobin
HbA1c	Glycosylated haemoglobin
HCRU	Health Care Resource Utilisation
HDL	High Density Lipoprotein
HF	Heart Failure
HFE	Heart Failure Event
HFmrEF	Heart Failure with mid-range Ejection Fraction
HFpEF	Heart Failure with Preserved Ejection Fraction
HFrEF	Heart Failure with Reduced Ejection Fraction
HHF	Hospitalisation for Heart Failure
HR	Hazard Ratio
i.v.	intravenous

Proprietary confidential information © 2020 Boehringer Ingelheim International GmbH or one or more of its affiliated companies

IABP	Intra-Aortic Balloon Pump
IB	Investigator's Brochure
ICD	Implantable Cardioverter Defibrillator
ICF	Informed Consent Form
ICH	International Council on Harmonisation
IEC	Independent Ethics Committee
IMP	Investigational Medicinal Product
IRB	Institutional Review Board
IRT	Interactive Response Technology
ISF	Investigator Site File
ITT	Intention To Treat
JVP	Jugular Venous Pressure
KCCQ	Kansas City Cardiomyopathy Questionnaire
KCCQ-CSS	Kansas City Cardiomyopathy Questionnaire - Clinical Summary Score
KCCQ-TSS	Kansas City Cardiomyopathy Questionnaire - Total Symptom Score
LDL	Low-Density Lipoprotein
LPLT	Last Patient Last Treatment
LVAD	Left Ventricular Assist Device
LVEF	Left Ventricular Ejection Fraction
MACE	Major Adverse Cardiovascular Events
MedDRA	Medical Dictionary for Drug Regulatory Activities
MMRM	Mixed Model Repeated Measure
MOA	Mode of Action
MRI	Magnetic Resonance Imaging
NCC	National Coordinator Committee
NT-proBNP	N-Terminal Pro-Brain Natriuretic Peptide
NYHA	New York Heart Association
OPU	Operative Unit
PCI	Percutaneous coronary intervention
PGI-S	Patient Global Impression of Severity of Heart Failure Symptoms
PPS	Per Protocol Set
PRO	Patient Reported Outcome
PV	Pharmacovigilance

Proprietary confidential information © 2020 Boehringer Ingelheim International GmbH or one or more of its affiliated companies

q.d.	quaque die (once a day)
RA	Regulatory Authority
RBC	Red Blood Cells / Erythrocytes
REP	Residual Effect Period
RS	Randomised Set
SAE	Serious Adverse Event
SBP	Systolic Blood Pressure
SGLT-1	Sodium Glucose Co-Transporter 1
SGLT-2	Sodium Glucose Co-Transporter 2
SMQ	Standardised MedDRA Query
SOP	Standard Operating Procedure
SUSAR	Suspected Unexpected Serious Adverse Reactions
T1DM	Type 1 Diabetes Mellitus
T2DM	Type 2 Diabetes Mellitus
TAVI	Transcatheter Aortic Valve Implantation
TIA	Transient Ischemic Attack
TMF	Trial Master File
TS	Treated Set
TSAP	Trial Statistical Analysis Plan
TSS	Total Symptom Score
ULN	Upper Level of Normal
UTI	Urinary Tract Infections
VT	Ventricular Tachycardia
WBC	White Blood Cells / Leukocytes
WHO	World Health Organisation
WOCBP	Woman of childbearing potential

Proprietary confidential information © 2020 Boehringer Ingelheim International GmbH or one or more of its affiliated companies

1. INTRODUCTION

1.1 MEDICAL BACKGROUND

Heart failure (HF) is one of the most prevalent chronic diseases associated with high mortality and morbidity. Over 22 million people around the world suffer from chronic heart failure (CHF), of which more than 5.5 million live in the US. Patients with HF are at high risk of mortality and morbidity, 50% die within 5 years of their diagnosis of HF and a large number are re-hospitalised for exacerbation of HF symptoms [P16-03952, P16-05920]. Heart failure is the most frequent cause of hospital admission among patients 65 years or older [P16-03952], and there are over 1 million hospitalised patients each year with HF as a primary diagnosis [R19-2918].

The cost burden of treating patients with HF is estimated to be $32 billion a year, and expected to rise to $70 billion a year by 2030. Approximately 80% of the cost is related to hospital admission [R19-2917].

Two main types of HF have been defined mainly based on the left ventricular ejection fraction (LVEF) and also other structural changes in the heart. They consist of heart failure with reduced EF (HFrEF) ≤40% and heart failure with preserved EF (HFpEF) >40%. Relative prevalence of HFrEF among HF patients is approximately 50% [R16-1528]. European Society of Cardiology in their 2016 guideline introduced a third type of heart failure with LVEF ranging between 40-49% named heart failure with mid-range ejection fraction (HFmrEF). The exact characteristics of these patients, response to therapy or prognosis is yet to be determined. In this trial, HF patients with full spectrum of EF are allowed to participate.

Outcomes for patients after a hospitalisation for HF (HHF) remain poor. Among patients with HF, 40-50% demonstrate worsening of HF symptoms, and 30-40% are re-hospitalised within 6 months after discharge from hospital [R19-2916, R19-2915]. After HHF, the one-year mortality rate is high and essentially not different between patients with preserved or reduced LVEF [R16-2217], underscoring a high unmet medical need in this population.

Most hospital admissions for acute decompensation of HF are due to volume overload, evident by weight gain or information gathered from haemodynamic monitors or insertable devices [R19-2914]. In addition, despite apparent symptom control at the time of discharge from hospital, up to 40% of patients still have residual congestion [R19-2913].

The current treatment strategy for patients hospitalised for acute cardiac decompensation is mainly focused around maintaining previously established guideline directed medical therapy, blood pressure stabilisation, optimising volume status, in HFrEF pts initiating life-saving therapies, and initiating beta-blockers if indicated [P13-06404]. Based on available data, in well-selected patients who are haemodynamically stable during hospitalisation for HF, guideline-recommended HF-related medications can be safely initiated [R19-2924], including a recent trial of sacubitril/valsartan, which demonstrated safe and efficacious use in stabilised patients with acute decompensated heart failure with low EF before discharge [R19-2932].

Proprietary confidential information © 2020 Boehringer Ingelheim International GmbH or one or more of its affiliated companies

In-hospital initiation of different therapies is one of the best predictors of long-term adherence to medications [R19-2911, R19-2919, R19-2921] and long-term improvement in prognosis [R19-2924].

Empagliflozin is an orally available inhibitor of the renal dependent sodium glucose cotransporter 2 (SGLT-2), promoting urinary glucose excretion. Empagliflozin is indicated for reduction of blood glucose in patients with Type 2 Diabetes Mellitus (T2DM), and for cardiovascular (CV) death risk reduction in patients with T2DM and established CV disease. Empagliflozin also reduces blood pressure, arterial stiffness and measures of myocardial workload, likely through various mechanisms, as well as improving other CV risk factors (e.g. uric acid, visceral fat mass, albuminuria; [P15-00589, P15-09541].

In 2010, BI initiated the EMPA-REG (Empagliflozin – Reducing Excess Glucose) OUTCOME trial to explore the CV benefit of the drug as well as to establish the CV safety of empagliflozin [P15-09840]. This trial was completed in 2015 and showed that empagliflozin, when given in addition to standard of care treatment in high CV risk patients with T2DM, reduces the risk of 3-point major adverse cardiovascular events (MACE) by 14%, which was mostly driven by a 38% reduction in CV death. Furthermore, this trial demonstrated a relative reduction in the prespecified and adjudicated composite outcome of CV death or HHF by 34% and HHF by 35%.

Consistent with the main results of the EMPA-REG OUTCOME trial, in approximately 10% of the trial population who had investigator-reported heart failure at baseline (with subtype of heart failure not characterized), empagliflozin showed reduction in CV death, HHF, and the composite of HHF or CV death [P16-01253].

In the EMPA-REG OUTCOME trial, empagliflozin was associated with a lower risk of post-acute heart failure rehospitalisation and mortality [P19-01985]. These results indicate that treatment with empagliflozin may play a role in reducing the risk in the post-acute HF period, with a significant early impact on HF hospital readmissions and mortality.

Currently, two large clinical outcome trials are ongoing in patients with chronic HFpEF (EMPEROR-preserved) or HFrEF (EMPEROR-reduced) to evaluate the effect of empagliflozin for the reduction of cardiovascular death and heart failure hospitalisation.

In addition, two clinical trials in patients with chronic HFpEF (EMPERIAL-preserved [c26554599-01]) or HFrEF (EMPERIAL-reduced [c26554767-01]) to evaluate the effect of empagliflozin on exercise ability using the 6 minute walk test are completed.

It should be noted, that with the exception of smaller investigator-initiated case studies, no prior clinical trial has assessed the effect of empagliflozin in patients hospitalized with HF.

1.2 DRUG PROFILE

Empagliflozin is an orally available, potent, and selective inhibitor of the renal SGLT-2. Its selective inhibition reduces renal reabsorption of sodium and glucose. This leads to both increased urinary sodium and glucose excretion. While urinary sodium excretion returns to

Proprietary confidential information © 2020 Boehringer Ingelheim International GmbH or one or more of its affiliated companies

near normal within a few days of empagliflozin administration, the effect on urinary glucose continues for as long as the medication is used.

Empagliflozin has been developed for the treatment of T2DM, and has received marketing approval in various regions including for example the European Union, Latin American countries, USA and Japan where it is marketed under the brand name Jardiance®.

For a more detailed description of the drug profile please refer to the current Investigator's Brochure (IB) [c01678844] and local prescribing information for empagliflozin.

The Residual Effect Period (REP) of empagliflozin is 7 days. This is the period after the last dose with measurable drug levels and/or pharmacodynamic effects still likely to be present.

1.3 RATIONALE FOR PERFORMING THE TRIAL

Outcomes for patients after a HHF remain poor but in-hospital initiation of different therapies is one of the best predictors of long-term adherence to medications [R19-2911, R19-2919, R19-2920] and long-term improved prognosis [R19-2924], see Section 1.1 for further details.

For empagliflozin, the mode of action (MOA) suggests a potential for improving patients` fluid retention and congestion in the context of cardiac decompensation, which is the rational for performing this study:

- The glucosuria mediated osmotic diuresis, as well as natriuresis, is thought to result in long-lasting haemodynamic changes, less extracellular volume, increase of haemoglobin, shift in fuel supply from glucose to fat oxidation and towards more energy-efficient ketones, possible reduction of vascular wall stress, decrease of ventricular load and improving cardiac function [P15-00589, P15-09541]. While empagliflozin lowers blood pressure, this change is not associated with an increase in heart rate unlike what has been observed for vasodilators [P14-01668].

- The described effects start as early as with the first dose of empagliflozin taken or within the first few weeks of its administration [P16-01830], which would be in line with the observed early effects on reduction in HHF as shown in the EMPA-REG OUTCOME trial. See Figure 1.3: 1.

Proprietary confidential information © 2020 Boehringer Ingelheim International GmbH or one or more of its affiliated companies

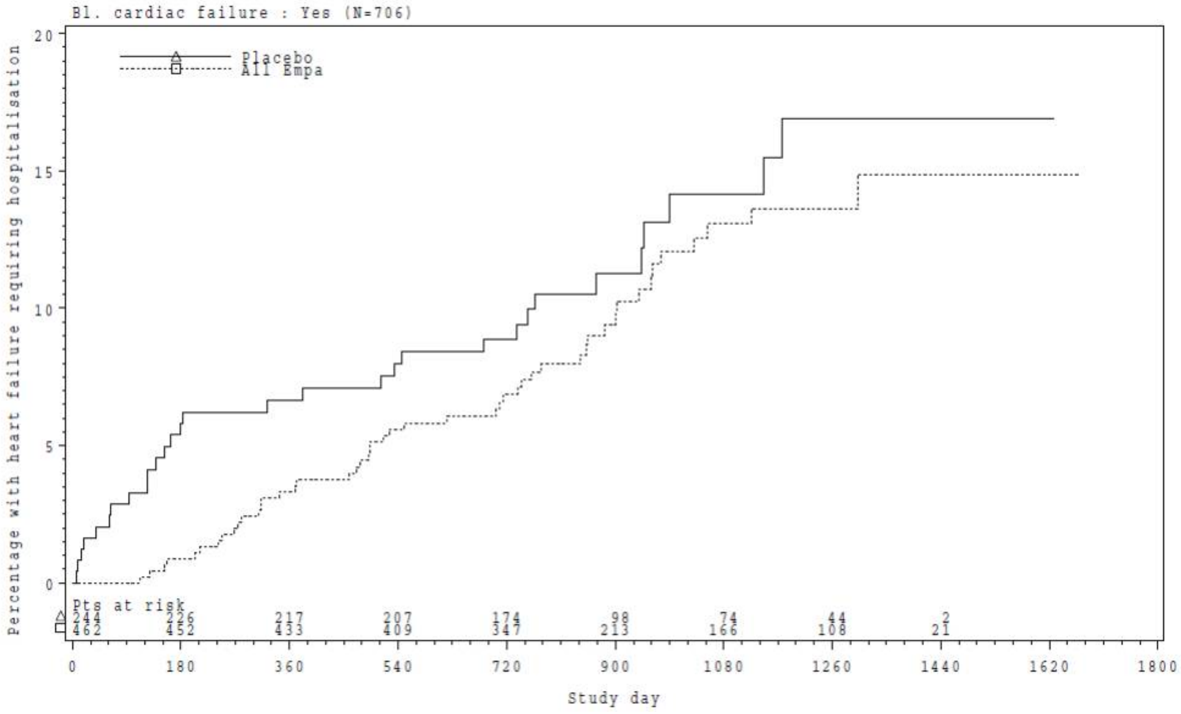

Figure 1.3: 1 Kaplan-Meier estimates of time to first heart failure requiring hospitalisation in patients with baseline cardiac failure [narrow standardized MedDRA query (SMQ)], pooled empagliflozin doses versus placebo-treated set. X-axis: study day [c02695839-01].

- Patients with HFpEF are thought to benefit from combined beneficial effects of empagliflozin (see above). This benefit is expected based on the increased prevalence of obesity, hypertension, and anaemia in patients with HFpEF [R17-1562], as these concomitant conditions may affect heart failure (obesity: increased peripheral resistance; hypertension: increased cardiac workload and oxygen consumption; anaemia: reduced cardiac oxygen supply).

- Although HFpEF and HFrEF are considered as different disease entities, patients with HFrEF are thought to profit from the same combined effects of empagliflozin, with potentially different weighting of the individual beneficial effects.

- Presently, there are two large clinical outcome trials ongoing with empagliflozin, the EMPEROR-reduced (NCT03057977, EudraCT 2017-004073-14) and EMPEROR-preserved trials (NCT03057951, EudraCT 2017-004072-59), investigating the long-term CV mortality and morbidity benefits in patients with chronic HFrEF and HFpEF. Even if these trials are proven positive for long-term clinical morbidity and mortality, it will not be known if in-hospital use of empagliflozin is safe in patients hospitalised for acute heart failure, since hospitalised patients were excluded from the EMPEROR trials. Due to its fast MOA, empagliflozin is expected to potentially alleviate congestive symptoms and residual fluid retention seen in patients with acute heart failure helping to stabilise HF-related symptoms within the first few days and weeks after discharge from hospital.

Proprietary confidential information © 2020 Boehringer Ingelheim International GmbH or one or more of its affiliated companies

As early treatment initiation is important but not covered by the ongoing clinical outcome trials in chronic HF, this trial is performed to assess HF-related outcomes (effect on heart failure events (HFEs), death and Kansas City Cardiomyopathy Questionnaire – Total Symptom Score (KCCQ-TSS)), and safety and tolerability of early initiation of treatment with empagliflozin in patients hospitalised for acute HF after stabilisation but still in hospital.

1.4 BENEFIT - RISK ASSESSMENT

Empagliflozin is currently indicated for reduction of blood glucose in patients with T2DM, and for CV death risk reduction in patients with T2DM and established CV disease.

The safety profile of empagliflozin has been well established in over 15000 patients with T2DM treated in clinical studies (of which more than 10000 were treated with empagliflozin) with maximum treatment duration of 4 years. Empagliflozin was tested in over 4600 patients with T2DM and high CV risk for median treatment duration of 2.6 years [P15-09840]. In addition, approximately 550 healthy volunteers were exposed to empagliflozin (up to 800 mg single dose and up to 50 mg multiple dosing). Based on the mode of action of empagliflozin, which is independent of insulin and potential concomitant T2DM, it is not expected that the safety profile in patients without T2DM would be different to that in patients with T2DM [P17-04479].

Because of the mode of action, blockade of the SGLT2 transporter by empagliflozin leads to glucosuria in patients with and without diabetes, although with less average daily glucose excretion in non-diabetic patients. Therefore, it is considered likely that the tolerability of empagliflozin in non-diabetic patients will be as favourable as in those with T2DM.

Available data from completed and ongoing trials do not indicate safety concerns for non-diabetic CHF patients, other than those already described for patients with T2DM.

1.4.1 Benefits

A detailed rationale for the use of empagliflozin in HF can be found in Section 1.1 and 1.3.

Patients with HF are thought to benefit from combined effects of empagliflozin such as persistent osmodiuresis, reduction in blood pressure, weight loss, increase in haematocrit, and changes in cardiometabolic parameters as early as the first few weeks of empagliflozin administrations. Due to the combination of positive effects, empagliflozin is expected to address the unmet medical need of reducing congestion in this patient population.

It has been shown in healthy volunteers that dosing with empagliflozin results in glycosuria approximating 2/3 the average glucosuria in patients with T2DM. This is similar to the amount of glucose lost in T2DM subjects with moderate renal impairment [P13-04190]. Because no difference in CV benefit was detected for patients with renal impairment vs. the overall population in the EMPA-REG OUTCOME study, it is hypothesised that the amount of glucosuria is not the main factor for obtaining CV effects with empagliflozin. In the same study, empagliflozin improved the main outcome of CV death and HHF with a similar magnitude in patients with low or high levels of glycosylated haemoglobin (HbA1c) at baseline, and independent of the amount of HbA1c change throughout the trial. In addition, a

Proprietary confidential information © 2020 Boehringer Ingelheim International GmbH or one or more of its affiliated companies

mechanistic study in non-diabetic subjects [P16-01830] provide supporting evidence that the benefit of empagliflozin in treating HF patients should also be expected in patients without diabetes. Taking all the data into consideration, the risk reduction for HF outcome appears to be independent of the glycaemic status at baseline or the degree of glycaemic control throughout the trial or the amount of glucosuria, which suggests the benefits could be achieved in both diabetic and non-diabetic populations. [P15-09840, P18-10152]

1.4.2 Risks

Table 1.4.2: 1: Known and potential risks of clinical relevance for the study population

Known or potential risks of clinical relevance for this trial	Summary of data, rationale for the risk	Mitigation strategy
Investigational Medicinal Product: Empagliflozin		
Hypoglycaemia	The risk of hypoglycaemia was increased only when empagliflozin was used concomitantly with insulin and/or sulfonylurea. The risk of hypoglycaemia of patients without diabetes mellitus (DM) is considered very low. In a mechanistic study [c11963611-01] subjects without DM had an increase of endogenous glucose production in response to glycosuria due to empagliflozin.	Patients with type 1 diabetes mellitus (T1DM) are excluded from the study.<br>Guidance for the investigator provided in the IB.
Volume depletion	Empagliflozin may selectively reduce interstitial volume with minimal change in intravascular volume.<br>Polyuria and consequent dehydration and hypotension were identified as risks in patients treated with empagliflozin, especially patients with known CV disease, history of hypotension, taking diuretics, other antihypertensive drugs, or elderly patients aged 75 years and older. [P19-02151]	Hospitalised patients will be randomised after diuretics stabilisation.<br>Guidance for the investigator provided in the IB.<br>Information and recommendations for the patients provided in the Informed Consent Form (ICF).

Proprietary confidential information © 2020 Boehringer Ingelheim International GmbH or one or more of its affiliated companies

Table 1.4.2: 1: Known and potential risks of clinical relevance for the study population (cont.)

Known or potential risks of clinical relevance for this trial	Summary of data, rationale for the risk	Mitigation strategy
Investigational Medicinal Product: Empagliflozin		
Diabetic ketoacidosis (DKA)	Rare cases of DKA, including fatal cases, were reported in patients treated with SGLT2-inhibitors. In patients treated with SGLT2-inhibitors DKA may occur with lower than usual glucose values. The risk of DKA is increased in patients with lower than needed insulin intake, T1DM, low carbohydrate intake, acute illness, major trauma, operation, severe dehydration or alcohol use.	Patients with T1DM are excluded from the study. Guidance for the investigator provided in the IB. Training will be provided. Information and recommendations for the patients provided in the ICF. DKA is an adverse even of special interest (AESI). Cases reported as DKA or metabolic acidosis are adjudicated. (see Sections 4.2.1 and 8.7; details are also described in the adjudication charter)
Complicated urinary tract infections (UTI)	Cases of complicated UTI, including pyelonephritis and urosepsis were reported in patients treated with empagliflozin.	Guidance for the investigator provided in the IB. Information and recommendations for the patients provided in the ICF.
Necrotizing fasciitis of perineum (Fournier's gangrene)	Rare cases of Fournier's gangrene, including fatal cases, were reported in patients treated with SGLT2-inhibitors.	Guidance for the investigator provided in the IB. Information and recommendations for the patients provided in the ICF.
Hypersensitivity	The risks of allergic skin reactions (e.g. rash, urticaria) and angioedema were identified for empagliflozin based on post-marketing experience. As with all drugs, the risk of severe and unexpected allergic reactions cannot be excluded.	Patients with hypersensitivity to empagliflozin are excluded from trial participation.

Proprietary confidential information © 2020 Boehringer Ingelheim International GmbH or one or more of its affiliated companies

Table 1.4.2: 1: Known and potential risks of clinical relevance for the study population (cont.)

Known or potential risks of clinical relevance for this trial	Summary of data, rationale for the risk	Mitigation strategy
Investigational Medicinal Product: Empagliflozin		
Drug-induced liver injury (DILI)	No risk of DILI was identified for empagliflozin. However, DILI generally can be severe and lead to fatal outcome or need of liver transplant. Therefore, careful monitoring and assessment of patients for potential DILI is needed.	Parameters for potential liver injury are included in the safety laboratory.<br>Cases of liver impairment are defined as an AESI. Severe cases of liver impairment are adjudicated. (see Sections 5.2.4, 5.2.7 and 8.7; details are also described in the adjudication charter)
Renal safety	In clinical trials in patients with DM, the incidence of renal impairment was similar to placebo. An initial decrease of estimated glomerular filtration rate (eGFR) was seen in patients treated with empagliflozin, which improved during continuous treatment or discontinuation of empagliflozin.<br>Cases of renal impairment, including requiring dialysis, were reported in patients using SGLT2-inhibitors. Due to the renal mode of action and risk of volume depletion, the renal safety should be monitored.	Parameters for potential renal impairment are included in the safety laboratory.<br>Cases of renal impairment are defined as an AESI (see Sections 5.2.4 and 5.2.7.1.4).
Women of childbearing potential (WOCBP)	The safety of empagliflozin in pregnant women was not established.	In accordance with international regulatory guidelines, WOCBP are excluded from trial participation unless they agree to use highly effective contraceptive method.<br>All WOCBP undergo pregnancy testing before being randomised.

Proprietary confidential information © 2020 Boehringer Ingelheim International GmbH or one or more of its affiliated companies

Known and potential risks in healthy volunteers and patients with T2DM:

For the main risks, please refer to Table 1.4.2: 1

In clinical studies, empagliflozin was well tolerated in both healthy volunteers and patients with T2DM including patients with high CV risk. The frequency of overall adverse events (AEs), AEs leading to discontinuation and serious adverse events (SAEs) were comparable to placebo [c01678844].

In general there was a small increase in frequency of urinary tract infection (UTI) compared to placebo. There was an increase in frequency of genital infections with the use of empagliflozin. There was a small increase in total cholesterol, low-density lipoprotein (LDL) cholesterol and high-density lipoprotein (HDL) cholesterol and no significant changes in triglycerides. No clinically relevant changes in electrolytes were observed with empagliflozin [c01678844].

Known and potential risks in patients with CHF and without T2DM:

In the empagliflozin HF program including two ongoing large EMPEROR outcome trials, and two EMPERIAL exercise capacity trials (with more than 7500 patients being followed-up as of May 2019), the frequencies of overall adverse events (AE), severe AE, serious AE, and AE leading to treatment discontinuation reported in patients without DM were similar or lower than the ones reported in patients with DM and consistent with the known safety reporting described in the Investigator Brochure. The frequency of AE of hypoglycaemia in patients without DM was lower than in patients with DM.

As of July 2019 based on blinded data review there has been no severe hypoglycaemia (i.e. requiring assistance) or serious hypoglycaemic events and no case of ketoacidosis reported in patients without DM and no other safety concerns were raised in this population.

Adverse events related to volume depletion, hypotension, urinary tract infections and genital infections in patients without DM were reported with similar frequency as in patients with DM.

Many patients with HF have renal impairment, and to ensure that the trial results reflect this population, patients with estimated glomerular filtration rate (eGFR) ≥ 20 ml/min/1.73m2 can be included. In the EMPA-REG Outcome trial, the cardiovascular benefits for empagliflozin were not driven by its pharmacological effect of lowering blood glucose and were consistently noted in patients with different degrees of renal impairment, including 540 patients with eGFR between > 30 and < 45 ml/min/1.73m^2. In previous trials in patients with T2DM, the safety profile in moderate and severe renal impairment was comparable to the overall trial population [P17-10453].

In the ongoing EMPEROR trials there have been more than 1300 patients with eGFR between ≥ 30 and < 45 ml/min/1.73m^2 with no safety issue identified to date. As part of the periodic Data Monitoring Committee (DMC) review, the safety in more than 400 patients with eGFR less than 30 ml/min/1.73m^2 has also been specifically evaluated and as of July 2019 there have been no safety issues.

Proprietary confidential information © 2020 Boehringer Ingelheim International GmbH or one or more of its affiliated companies

In addition, a dapagliflozin outcome trial in over 4700 patients with HFrEF showed a significant risk reduction in the composite primary outcome of CV death and HHF with a similar safety profile compared to placebo. In this trial, patients without DM (nearly 60%) also showed a positive trend towards improvement of CV death and HHF and a safety profile similar to patients with DM. There was no increased risk of hypoglycaemia or ketoacidosis in patients without DM [R19-3125].

Renal safety will be closely monitored throughout the trial (see Section 5.2.4; Flow Chart).

In addition an independent Data Monitoring Committee (DMC) is implemented who is following up on patients` safety in an unblinded manner. Please refer to Section 8.7 for details.

Risk evaluation in relation with COVID-19

Patients with chronic heart diseases are at higher risk for severe illness from COVID-19. Therefore, in case of local high risk of COVID-19 infection, physical visits to the sites should be avoided as much as possible. In the event of restriction to visit the investigator site, certain procedures can be done remotely, and local labs can be used instead of central lab. These changes are meant to keep the integrity of the trial and they will not affect the benefit-risk of empagliflozin.

There is no indication that empagliflozin may increase the risk of COVID-19 infection. As with any acute illness, empagliflozin during COVID-19 infection has the potential to increase the risk of ketoacidosis. The risk of ketoacidosis in case of acute illness is adequately addressed in the IB. The study drug should be discontinued in case of severe COVID-19 disease.

1.4.3 Discussion

The overall tolerability and safety profile, as outlined in this Section 1.4 and the evidence as summarized in the current Investigator's Brochure (IB), supports chronic safe administration of empagliflozin 10 mg in human studies, specifically in the heart failure population intended for inclusion into this trial.

Patients will be treated according to standard of care and trial medication will be given in addition to standard therapy. It should also be noted that empagliflozin has not been approved for the treatment of HF. Given the short duration of the trial, the lack of an indication of empagliflozin for the treatment of heart failure and the gap in knowledge in heart failure patients in the acute setting, it is justified to include patients with type 2 diabetes mellitus in this trial, who may receive other antidiabetic therapies for controlling their glucose levels. Therefore, the placebo controlled trial design does not constitute a risk for the population in this study.

Due to the combination of positive effects, empagliflozin is expected to address the unmet medical need of reducing congestion in the patient population under investigation in this trial.

Overall the potential benefits, coupled with an acceptable safety profile support the initiation of the trial.

Proprietary confidential information © 2020 Boehringer Ingelheim International GmbH or one or more of its affiliated companies

2. TRIAL OBJECTIVES AND ENDPOINTS

2.1 MAIN OBJECTIVES, PRIMARY AND SECONDARY ENDPOINTS

2.1.1 Main objectives

The main objective of this study is to assess whether in-hospital administration of empagliflozin results in improvements in HF-related clinical events and patient-reported outcomes (death, HFE and KCCQ-TSS as a measure of health status (symptoms)) in patients hospitalised for acute heart failure (de novo or decompensated chronic HF) and after initial stabilisation.

Secondary objectives are to further assess whether it is safe to start empagliflozin in patients admitted to hospital in this setting. See Section 2.1.3.

This trial is part of an investigational clinical trial program of empagliflozin in patients with HF.

2.1.2 Primary endpoint(s)

The primary endpoint is clinical benefit, a composite of death, number of heart failure events (HFE) (including hospitalisations for heart failure (HHFs), urgent heart failure visits and unplanned outpatient visits), time to first HFE and change from baseline in KCCQ-TSS after 90 days of treatment assessed by the win ratio.

2.1.3 Secondary endpoint(s)

Secondary endpoints to assess safety and efficacy in this setting are:

- Improvement in KCCQ-TSS of ≥ 10 points after 90 days of treatment.
- Change from baseline in KCCQ-TSS after 90 days of treatment.
- Change from baseline in log-transformed N-Terminal Pro-Brain Natriuretic Peptide (NT-proBNP) level over 30 days of treatment (area under the curve (AUC)).
- Days alive and out of hospital from study drug initiation until 30 days after initial hospital discharge.
- Days alive and out of hospital from study drug initiation until 90 days after randomisation.
- Time to first occurrence of CV death or HFE until end of trial visit.
- Occurrence of HHF until 30 days after initial hospital discharge.

Proprietary confidential information © 2020 Boehringer Ingelheim International GmbH or one or more of its affiliated companies

- Occurrence of chronic dialysis or renal transplant or sustained[1] reduction of ≥40% eGFR Chronic Kidney Disease Epidemiology Collaboration Equation ((CKD-EPI)cr), or
    - sustained eGFR (CKD-EPI)cr <15 mL/min/1.73 m^2 for patients with baseline eGFR ≥30 mL/min/1.73 m^2
    - sustained eGFR (CKD-EPI)cr <10 mL/min/1.73 m^2 for patients with baseline eGFR <30 mL/min/1.73 m^2
- Diuretic effect as assessed by weight loss per mean daily loop diuretic dose after 15 days of treatment.
- Diuretic effect as assessed by weight loss per mean daily loop diuretic dose after 30 days of treatment.

2.2 FURTHER OBJECTIVES AND FURTHER ENDPOINTS

2.2.1 Further objectives

Further objectives are to evaluate additional and complimentary details on efficacy and safety.

2.2.2 Further endpoints

- Improvement in KCCQ-TSS of ≥ 5 points after 30 days of treatment.
- Improvement in KCCQ-TSS of ≥ 10 points after 30 days of treatment.
- Improvement in KCCQ-TSS of ≥ 20 points after 30 days of treatment.
- Improvement in KCCQ-TSS of ≥ 5 points after 90 days of treatment.
- Improvement in KCCQ-TSS of ≥ 20 points after 90 days of treatment.
- Change from baseline in KCCQ-TSS after 15 days of treatment.
- Change from baseline in KCCQ-TSS after 30 days of treatment.
- Mean of changes from baseline in KCCQ-TSS at Days 15, 30 and 90.
- Change from baseline in KCCQ overall summary score, Clinical Summary Score (CSS), and individual domains after 15, 30 and 90 days of treatment.
- Change from baseline in log-transformed NT-proBNP level over 15 days of treatment (AUC).
- Change from baseline in log-transformed NT-proBNP level over 90 days of treatment (AUC).
- Reduction of NT-proBNP ≥ 30% after 90 days of treatment.
- Time to occurrence of CV death until end of trial visit.
- Time to first occurrence of HFE until end of trial visit.
- Time to occurrence of all-cause mortality until end of trial visit.
- Time to first occurrence of HFE until end of trial visit.
- Occurrence of CV death, HFE until 30 days after initial hospital discharge.

[1] An eGFR (CKD-EPI)cr reduction is considered sustained, if it is determined by two or more consecutive post-baseline central laboratory measurements separated by at least 30 days (first to last of the consecutive eGFR values). Chronic dialysis is regarded as chronic if the frequency of dialysis is twice or more per week for at least 90 days.

Proprietary confidential information © 2020 Boehringer Ingelheim International GmbH or one or more of its affiliated companies

- Occurrence of HFE until 30 days after randomisation.
- Occurrence of all-cause mortality until 30 days after initial hospital discharge.
- Occurrence of acute renal failure (based on narrow SMQ).
- Change from baseline in eGFR (CKD-EPI)cr after 15, 30 and 90 days of treatment.
- Occurrence of dialysis or renal transplant or sustained[2] reduction of ≥40% eGFR (CKD-EPI)cr, or
    - sustained eGFR (CKD-EPI)cr <15 mL/min/1.73 m^2 for patients with baseline eGFR ≥30 mL/min/1.73 m^2.
    - sustained eGFR (CKD-EPI)cr <10 mL/min/1.73 m^2 for patients with baseline eGFR <30 mL/min/1.73 m^2.
- Occurrence of first acute kidney injury (based on preferred term).
- Occurrence of symptomatic hypotension until 90 days after randomisation.
- Adherence to medication over 90 days.
- Change from baseline in body weight after 15, 30 and 90 days of treatment.
- Diuretic effect as assessed by weight loss per mean daily loop diuretic dose after 90 days of treatment.
- Change from baseline in haematocrit after 15, 30 and 90 days of treatment.
- Change from baseline in New York Heart Association (NYHA) class after 90 days of treatment.
- Occurrence of hypoglycaemic events.
- Occurrence of volume depletion.
- Change from baseline in Systolic Blood Pressure (SBP) after 15, 30 and 90 days of treatment.
- Change from baseline in Diastolic Blood Pressure (DBP) after 15, 30 and 90 days of treatment.
- Change from baseline in Clinical Congestion Score after 15 and 90 days of treatment.
- Change from baseline in Patient Global Impression of Severity of Heart Failure Symptoms after 90 days of treatment.

[2] An eGFR (CKD-EPI)cr reduction is considered sustained, if it is determined by two or more consecutive post-baseline central laboratory measurements separated by at least 14 days (first to last of the consecutive eGFR values).

Proprietary confidential information © 2020 Boehringer Ingelheim International GmbH or one or more of its affiliated companies

3. DESCRIPTION OF DESIGN AND TRIAL POPULATION

3.1 OVERALL TRIAL DESIGN

This is a randomised, double-blind, parallel-group, placebo controlled, multinational and multicentre study.

A total of approximately 500 male and female patients admitted to the hospital for acute HF will (de novo or decompensated chronic HF) be randomised (1:1) in the study in about 13 countries.
The pre-screening period will start when patients are admitted. Screening (Visit 1) and randomisation (Visit 2a) can happen on the same day. Randomisation should occur after at least 24 hours and no later than 5 days after admission and before discharge when patients fulfil criteria to indicate clinical stability including a stable dose of diuretics. Visit 2 consists of two further visits (Visit 2b and 2c) that will be performed only if the patient is still in hospital. All patients will be treated on top of standard of care. Patients will receive the study drug for 90 days. A follow-up phone call will be performed 7 days after the End of Treatment Visit.

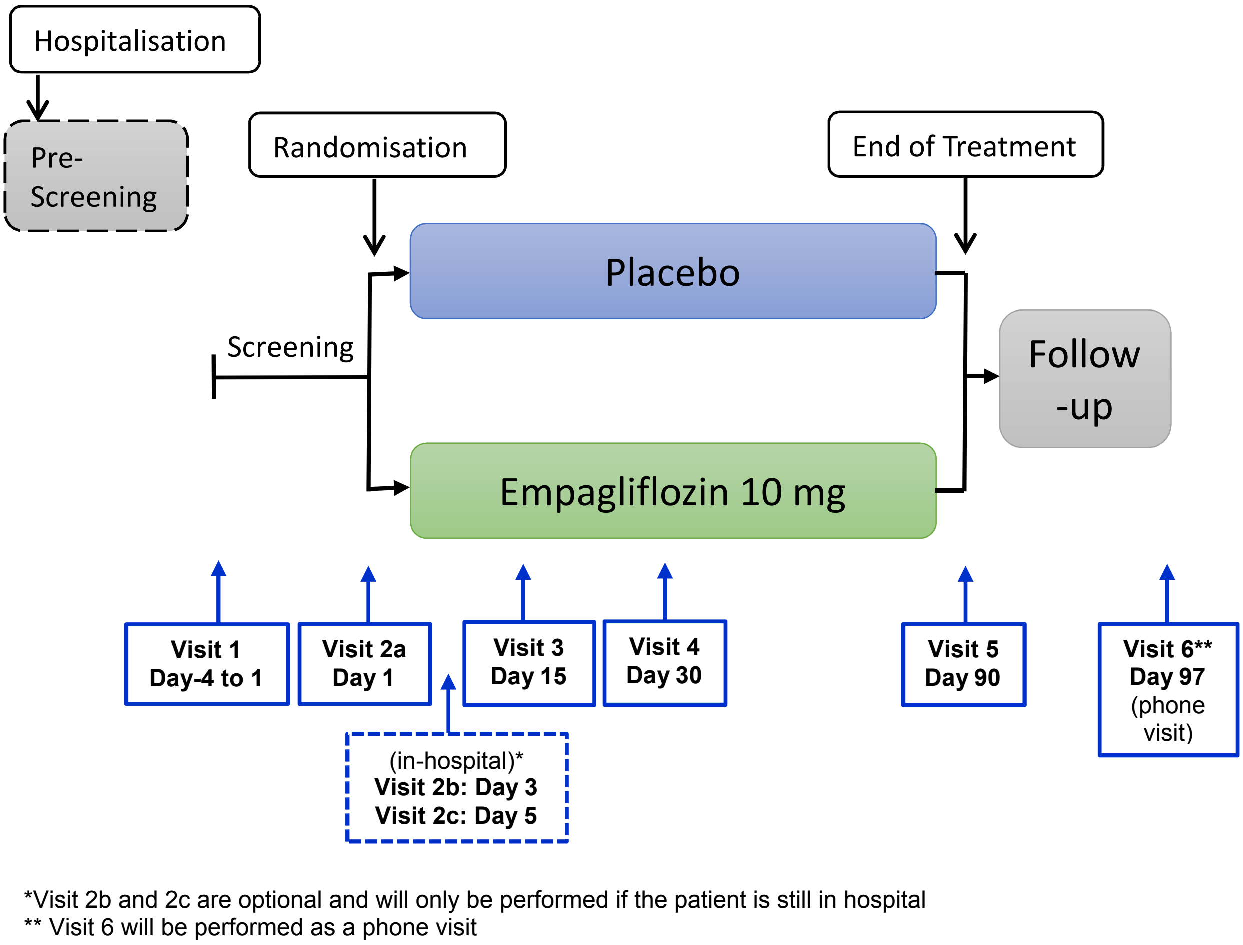

Figure 3.1: 1 Trial design

Proprietary confidential information © 2020 Boehringer Ingelheim International GmbH or one or more of its affiliated companies

The primary endpoint will be assessed at Visit 5.

Patients who prematurely stop medication should have an End of Treatment Visit but be followed up until their planned Visit 5 (Day 90) according to the visit schedule.

3.2 DISCUSSION OF TRIAL DESIGN, INCLUDING THE CHOICE OF CONTROL GROUP(S)

Hospitalisation for HF identifies patients at increased risk of death and re-hospitalisation following discharge. In the EMPEROR trials (see Section 1.1) patients hospitalised for HF were excluded.
Since the long-term mortality and morbidity of empagliflozin in patient with chronic HF will be assessed in EMPEROR trials, the main objective of this study is to assess whether in-hospital administration of empagliflozin leads to an improvement in clinical benefit (a composite of death, number of HFEs, time to first HFE and change from baseline in KCCQ-TSS) in patients hospitalised for acute heart failure (de novo or decompensated chronic) and after initial stabilisation assessed by the win ratio. Secondary objectives are to further assess whether it is safe to start empagliflozin in patients already admitted to hospital in this setting.

Choice of endpoint:

Patient-reported outcomes (PRO) can provide information on a range of patients' health status. Improving patient's symptoms, physical limitations and quality of life is one of the key goals of managing HF, and is endorsed by practice guidelines and the regulators. Health authorities, including US FDA, and major payers such as the Center for Medicare and Medicaid are increasingly interested in PROs [R19-3040, R19-3126]. To better evaluate the effect of empagliflozin on improving health status (symptoms, and physical limitations), the Kansas City Cardiomyopathy Questionnaire (KCCQ) will be used in this trial. The KCCQ is one of the most validated, clinically responsive and widely used HF PRO instruments [R17-2687].

In addition, in DAPA-HF, an outcome trial in over 4700 patients with HFrEF, dapagliflozin showed significant improvement in change in Kansas City Cardiomyopathy Questionnaire - Total Symptom Score (KCCQ-TSS) from baseline at 8 months. A similar improvement in KCCQ overall summary score and KCCQ-TSS was also detected in DEFINE-HF, a study of approximately 260 patients evaluating the effect of dapagliflozin compared to placebo on biomarkers, symptoms, and functional status in patients with HFrEF (both with and without DM) [R19-3125, R19-3124]. KCCQ is a self-administered questionnaire designed and validated to evaluate physical limitations, symptoms (frequency, severity, and changes over time), social limitations, self-efficacy, and quality of life in patients with HF (refer to Appendix 10.1).

In this trial, the KCCQ-TSS will be assessed by the win ratio as part of a composite clinical benefit endpoint including mortality and HF events (see Section 7.2.2).

General safety including parameters relevant to the empagliflozin MOA i.e. hypotension and renal function will be the focus of the safety assessment in this trial. Although it is likely that clinical events like death and hospitalisations will occur in this trial, this study is limited in its

Proprietary confidential information © 2020 Boehringer Ingelheim International GmbH or one or more of its affiliated companies

statistical power to conclusively assess the effect on morbidity and mortality outcomes with the planned sample size.

Control group:

Due to its mode of action, empagliflozin has the potential to be efficacious in treating patients' congestion with HF. However, the effect of empagliflozin in patients with acute heart failure (de novo or decompensated chronic) after stabilisation in hospital has not yet been systematically assessed in a randomised clinical trial. Currently, two large clinical outcome trials are ongoing in patients with HFpEF (EMPEROR-preserved) or HFrEF (EMPEROR-reduced) to evaluate empagliflozin for the reduction of cardiovascular death and heart failure hospitalisation in patients outside the hospital setting.

The placebo-controlled design is considered ethically acceptable on the basis of appropriate criteria for patient selection and discontinuation, the ability to change background therapy to maintain, or obtain sufficient levels of haemodynamic and glycaemic control (in patients with T2DM) as defined in relevant local and regional guidelines for optimised standard of care.

Patients should be receiving appropriate care as defined by their physician or practitioner for all cardiovascular conditions according to the prevailing guidelines. In HFrEF patients, this includes, but is not limited to (if indicated and not contraindicated) acetylsalicylic acid, statins, a diuretic, an inhibitor of the renin-angiotensin system with or without neprilysin inhibitor, a beta-blocker and a mineralocorticoid receptor antagonist, each to be given at clinically appropriate doses, and the use of implantable devices like pacemakers or implantable cardioverter defibrillators (ICDs, or cardiac resynchronisation therapies (CRTs)). This therapy should be selected in the context of local or regional guidelines for primary or secondary CV disease prevention.

Duration:

Due to the potential early effect of empagliflozin (see Section 1.1 for mode of action and EMPA-REG-OUTCOME results) empagliflozin is expected to exert its effect within a few days to weeks of administration.

The primary endpoint is clinical benefit, a composite of death, number of HFEs, time to first HFE and change from baseline in KCCQ-TSS at Day 90 assessed by the win ratio.

Thus, a 90 day time point is considered suitable for the assessment of the primary endpoint and evaluation of empagliflozin safety.

3.3 SELECTION OF TRIAL POPULATION

A total number of approximately 500 patients are planned to be randomised in about 13 countries and approximately 125 sites.

Proprietary confidential information © 2020 Boehringer Ingelheim International GmbH or one or more of its affiliated companies

The study is planned to be conducted in the US/North America and in Europe and may be expanded to additional countries (e.g. in Asia, South America or Eastern Europe) and more sites based on patient availability.

Screening of patients for this trial is competitive, i.e. screening for the trial will stop at all sites at the same time once a sufficient number of patients has been screened. Investigators will be notified about screening completion and will then not be allowed to screen additional patients for this trial.

A log of all patients enrolled into the trial (i.e. who have signed informed consent) will be maintained in the Investigator Site File (ISF) irrespective of whether they have been treated with investigational drug or not.
If a patient is enrolled in error (does not meet all inclusion criteria or meets one or more exclusion criteria on the day of enrolment), the sponsor should be contacted immediately.

3.3.1 Main diagnosis for trial entry

Patients admitted to the hospital for treatment of acute HF (de novo or decompensated chronic HF). Patients should be randomised after at least 24 hours and no later than 5 days after hospital admission. Randomisation should occur as soon as the patient is stabilised (see inclusion criterion 7).

Please refer to Section 8.3.1 (Source Documents) for the documentation requirements pertaining to the in- and exclusion criteria.

3.3.2 Inclusion criteria

1. Of full age of consent (according to local legislation, at least ≥ 18 years) at screening.

2. Signed and dated written informed consent in accordance with ICH-GCP and local legislation prior to admission to the trial.

3. Male or female patients. Women of childbearing potential (WOCBP)[3] must be ready and able to use highly effective methods of birth control per ICH M3 (R2) that result in a low failure rate of less than 1% per year when used consistently and correctly. A list of contraception methods meeting these criteria is provided in the patient information.

4. Currently hospitalised for the primary diagnosis of acute heart failure (de novo or decompensated chronic HF), regardless of EF. Patients with a diagnosis of hospitalised heart failure must have the following HF signs and symptoms at the time of hospital admission:

---

[3] A woman is considered of childbearing potential, i.e. fertile, following menarche and until becoming post-menopausal unless permanently sterile.
Permanent sterilisation methods include hysterectomy, bilateral salpingectomy and bilateral oophorectomy.
Tubal ligation is NOT a method of permanent sterilisation. A woman who underwent tubal ligation is still considered as WOCBP. However tubal ligation is considered as a method of highly effective birth control.
A postmenopausal state is defined as no menses for 12 months without an alternative medical cause.

Proprietary confidential information © 2020 Boehringer Ingelheim International GmbH or one or more of its affiliated companies

a. Persistent dyspnoea at rest or with minimal exertion, and,
b. Signs of fluid overload, at least two of the following must apply:
    i. Congestion on chest X-ray,
    ii. Rales on chest auscultation,
    iii. Clinical relevant oedema (e.g. ≥1+ on a 0 to 3+ scale), indicating indentation of skin with mild digital pressure that requires 10 or more seconds to resolve in any dependent area including extremities or sacral region,
    iv. Elevated jugular venous pressure (JVP).

5. Evidence of LVEF (either reduced or preserved EF, preferably quantitative) as per local reading (by e.g. echocardiography, radionuclide ventriculography, invasive angiography, MRI or CT) preferably measured during current hospitalisation. A historical LVEF assessment may be used if there is no in-hospital measurement and if it was measured within 12 months prior to randomisation provided there was no major intervening disease affecting EF like MI. The LVEF should preferably be documented in a local report prior to randomisation. If no such report is available, LVEF, valve status and relevant pathologic cardiac findings from echocardiography or other local readings should be mentioned in the source documents.

6. Patients must be randomised after at least 24 hours and no later than 5 days after admission, as early as possible after stabilisation and while they are still hospitalised.

7. Patients must fulfil the following stabilisation criteria (while in the hospital):
    - Systolic BP ≥100mm Hg and no symptoms of hypotension in the preceding 6 hours,
    - no increase in i.v. diuretic dose for 6 hours prior to randomisation,
    - no i.v. vasodilators including nitrates within the last 6 hours prior to randomisation
    - no i.v. inotropic drugs for 24 hours prior to randomisation.

8. Elevated NT-proBNP ≥ 1600 pg/mL or BNP ≥400 pg/mL according to the local lab for patients without atrial fibrillation (AF); or elevated NT-proBNP ≥ 2400 pg/mL or BNP ≥600 pg/mL for patients with AF, measured during the current hospitalisation or in the 72 hours prior to hospital admission. For patients treated with an angiotensin receptor neprilysin inhibitor (ARNI) in the previous 4 weeks prior to randomisation, only NT-proBNP values should be used.

9. HF episode leading to hospitalisation must have been treated with a minimum single dose of 40 mg of i.v. furosemide (or equivalent i.v. loop diuretic defined as 20 mg of torasemide or 1 mg of bumetanide).

Proprietary confidential information © 2020 Boehringer Ingelheim International GmbH or one or more of its affiliated companies

3.3.3 Exclusion criteria

1. Cardiogenic shock.

2. Current hospitalisation for acute heart failure primarily triggered by pulmonary embolism, cerebrovascular accident, or acute myocardial infarction (AMI)[4] diagnosed using electrocardiogram (ECG), and/or cardiac imaging and/or coronary angiography.

3. Current hospitalisation for acute heart failure not caused primarily by intravascular volume overload; for example triggered by significant arrhythmia (e.g., sustained ventricular tachycardia, or atrial fibrillation/flutter with sustained ventricular response >130 beats per minute, or bradycardia with sustained ventricular arrhythmia <45 beats per minute), infection/sepsis, severe anaemia, or acute exacerbation of chronic obstructive pulmonary disease (COPD).

4. Below interventions in the past 30 days prior to randomisation or planned during the study:

   - Major cardiac surgery (for example coronary artery bypass graft or valve replacement), or Transcatheter Aortic Valve Implantation (TAVI), or percutaneous coronary intervention (PCI), or Mitraclip
   - All other surgeries that are considered major according to investigator judgement,
   - Implantation of a cardiac resynchronisation therapy device (CRT) (pacemakers or implantable cardioverter defibrillators (ICD) without resynchonization function are allowed),
   - cardiac mechanical support implantation
   - Carotid artery disease revascularisation (stent or surgery)

5. Acute coronary syndromes / myocardial infarction (increase in cardiac enzymes in combination with symptoms of ischaemia or newly developed ischaemic ECG changes), stroke or TIA in the past 90 days prior to randomisation.

6. Symptomatic ventricular tachycardia (VT) with syncope in patients without an ICD in the past 90 days prior to randomisation.

7. Current use or prior use of a SGLT-2 inhibitor or combined SGLT-1 and 2 inhibitor in the past 90 days prior to randomisation. Discontinuation of a SGLT-2 inhibitor or combined SGLT-1 and 2 inhibitor for the purposes of study enrolment is not permitted.

8. Heart transplant recipient, or listed for heart transplant with expectation to receive a transplant during the course of this trial (according to investigator judgement), or planned for palliative care for HF, or currently using or plan for left ventricular assist device (LVAD) or intra-aortic balloon pump (IABP) or any other type of mechanical

[4] *Please note: Troponin elevations are frequently seen in patients with acute heart failure and therefore are not sufficient for a diagnosis of AMI. In order to exclude an AMI, concomitant objective evidence of AMI based on typical symptoms, ECG, and/or cardiac imaging and/or coronary angiography should be available.*

Proprietary confidential information © 2020 Boehringer Ingelheim International GmbH or one or more of its affiliated companies

circulatory support, or patients on mechanical ventilation, or patients with planned inotropic support in an out-patient setting.

9. Haemodynamically significant (severe) uncorrected primary cardiac valvular disease planned for surgery or intervention during the course of the study (note: secondary mitral regurgitation or tricuspid regurgitation due to dilated cardiomyopathy is not excluded unless planned for surgery or intervention during the course of the study).

10. Cardiomyopathy due to infiltrative diseases (e.g. amyloidosis), accumulation diseases (e.g. haemochromatosis, Fabry disease), muscular dystrophies, cardiomyopathy with reversible causes (e.g. stress cardiomyopathy), hypertrophic obstructive cardiomyopathy, complex (according to investigator`s judgement) congenital heart disease, or known pericardial constriction.

11. Heart failure caused by peripartum cardiomyopathy or Tako-Tsubo cardiomyopathy diagnosed within the past 6 months, or active myocarditis, or uncorrected thyroid disease, or other acute structural heart disease as cause for acute cardiac decompensation (e.g. acute mitral cord rupture as cause for hospital admission for heart failure).

12. Symptomatic bradycardia or third degree heart block without a pacemaker.

13. SBP $\geq$ 180 mmHg at randomisation (confirmed with second measurement after 5 minutes).

14. Atrial fibrillation or atrial flutter with a documented resting heart rate > 110 bpm in ECG performed before randomisation (Visit 2a).

15. Impaired renal function, defined as eGFR < 20 mL/min/1.73 m^2 as measured during hospitalisation (latest local lab measurement before randomisation) or requiring dialysis.

16. Type 1 Diabetes Mellitus (T1DM).

17. Indication of liver disease, defined by serum levels of either alanine aminotransferase (ALT) (SGPT), aspartate aminotransferase (AST) (SGOT), or alkaline phosphatase above 3 x upper limit of normal (ULN) as measured during hospitalisation (latest local lab measurement before randomisation) or history of cirrhosis with evidence of portal hypertension such as varices.

18. Haemoglobin < 9 g/dl as measured during hospitalisation (latest local lab measurement before randomisation).

19. History of ketoacidosis, including DKA.

20. Gastrointestinal (GI) surgery or GI disorder that could interfere with trial medication absorption in the investigator’s opinion.

21. Any documented active or suspected malignancy or history of malignancy within 2 years prior to screening, except appropriately treated basal cell carcinoma of the skin or in situ

Proprietary confidential information © 2020 Boehringer Ingelheim International GmbH or one or more of its affiliated companies

carcinoma of uterine cervix or low risk prostate cancer (biopsy Gleason score of ≤ 6 and clinical stage T1c or T2a).

22. Presence of any disease other than heart failure with life expectancy less than 1 year.

23. Decision for palliative care in HF (informed patient decision to adhere to limited heart failure treatments only).

24. Patients who must or wish to continue the intake of restricted medications (see Section 4.2.2) or any drug considered likely to interfere with the safe conduct of the trial

25. Current or previous randomisation in another empagliflozin heart failure trial (i.e. studies 1245.110, 1245.121, 1245-0167, 1245-0168) or currently enrolled in another investigational device or drug trial, or less than 30 days since ending another investigational device or drug trial(s), or receiving other investigational treatment(s). Patients participating in a purely observational trial will not be excluded.

26. Known allergy or hypersensitivity to empagliflozin or any excipient of the Investigational Medicinal Product (IMP), or other SGLT-2 inhibitors.

27. Chronic alcohol or drug abuse or any condition that, in the investigator's opinion, makes them an unreliable trial patient or unlikely to complete the trial.

28. Women who are pregnant, nursing, or who plan to become pregnant while in the trial.

29. Any other clinical condition that would jeopardise patients safety while participating in this trial, or may prevent the patient from adhering to the trial protocol.

3.3.4 Withdrawal of patients from treatment or assessments

Patients may discontinue trial treatment or withdraw consent to trial participation as a whole ("withdrawal of consent") with very different implications; please see Sections 3.3.4.1 and 3.3.4.2 below.

An excessive withdrawal rate can have a severe negative impact on the scientific value of the trial. The "Intention To Treat (ITT)" analysis requires that all randomised patients be followed until trial end even if the trial medication was temporarily interrupted, discontinued or never started.

Every effort should be made to keep the patients in the trial: if possible on treatment, or at least to collect important trial data.

Measures to control the withdrawal rate include careful patient selection, appropriate explanation of the trial requirements and procedures prior to trial enrolment, as well as the explanation of the consequences of withdrawal.

The decision to discontinue trial treatment or withdraw consent to trial participation and the reason must be documented in the patient files and Case Report Form (CRF). If applicable,

Proprietary confidential information © 2020 Boehringer Ingelheim International GmbH or one or more of its affiliated companies

consider the requirements for Adverse Event collection reporting (please see Sections 5.2.7.2.1 and 5.2.7.2).

3.3.4.1 Discontinuation of trial treatment

An individual patient will discontinue trial treatment if:

- The patient wants to discontinue trial treatment, without the need to justify the decision.
- The patient has repeatedly shown to be non-compliant with important trial procedures and, in the opinion of both, the investigator and sponsor representative, is not willing or able to adhere to the trial requirements in the future.
- The patient needs to take concomitant medication that interferes with the investigational medicinal product (see Section 4.2.2.1) for restricted medication
- The patient can no longer receive trial treatment for medical reasons (such as surgery, adverse events, other diseases, or pregnancy).In case of a temporary reason for treatment discontinuation, trial treatment should be restarted if medically justified, please see Section 4.1.4.

Given the patient's agreement, the patient will undergo the procedures for the Early Discontinuation Visit as soon as possible after treatment discontinuation and the Follow-up Visit as outlined in the Flow Chart and Section 6.2.3.
In addition, if the patient discontinues early, every effort should be made for the patient to attend the regularly scheduled study visits and have all study procedures performed except those pertaining to drug intake. However if this is not possible, then the remaining visits should be conducted by phone. In the rare case that even this is not possible, at least vital status and information about HFEs should be retrieved and documented at the day of the scheduled Visit 5 (Day 90).

If a patient becomes pregnant during the trial, the trial medication will be stopped, the patient will be followed up during the trial and until birth or termination of the pregnancy (see further details in Section 5.2.7.2).

If new efficacy/safety information becomes available, Boehringer Ingelheim will review the benefit-risk-assessment and, if needed, pause or discontinue the trial treatment for all patients or take any other appropriate action to guarantee the safety of the trial patients.

3.3.4.2 Withdrawal of consent to trial participation

Patients may withdraw their consent to trial participation at any time without the need to justify the decision.

This will however mean that no further information may be collected for the purpose of the trial and negative implications for the scientific value may be the consequence. Furthermore it may mean that further patient follow up on safety cannot occur.

If a patient wants to withdraw consent, the investigator must be involved in the discussion with the patient and explain the difference between trial treatment discontinuation and withdrawal of consent to trial participation, as well as explain the options for continued follow-up after trial treatment discontinuation, please see Section 3.3.4.1 above.

Proprietary confidential information © 2020 Boehringer Ingelheim International GmbH or one or more of its affiliated companies

3.3.4.3 Discontinuation of the trial by the sponsor

Boehringer Ingelheim reserves the right to discontinue the trial overall or at a particular trial site at any time for the following reasons:

1. Failure to meet expected enrolment goals overall or at a particular trial site.
2. Emergence of any efficacy/safety information invalidating the earlier positive benefit-risk-assessment that could significantly affect the continuation of the trial.
3. Deviations from GCP, the trial protocol, or the contract impairing the appropriate conduct of the trial.

Further follow up of patients affected will occur as described in Section 3.3.4.1.
The investigator / the trial site will be reimbursed for reasonable expenses incurred in case of trial termination (except in case of the third reason).

Proprietary confidential information © 2020 Boehringer Ingelheim International GmbH or one or more of its affiliated companies

4. TREATMENTS

4.1 INVESTIGATIONAL TREATMENTS

The trial medication will be provided by Boehringer Ingelheim Pharma GmbH & Co.KG.

4.1.1 Identity of the Investigational Medicinal Products

The characteristics of test products are below:

Table 4.1.1: 1 Test product 1

Substance:	Empagliflozin
Pharmaceutical formulation:	Film-coated tablet
Source:	Boehringer Ingelheim GmbH & Co.KG
Unit strength:	10 mg
Posology	1 tablet once daily
Route of administration:	Oral

Table 4.1.1: 2 Test product 2

Substance:	Placebo to Empagliflozin 10 mg
Pharmaceutical formulation:	Film-coated tablet
Source:	Boehringer Ingelheim GmbH & Co.KG
Unit strength:	-
Posology	1 tablet once daily
Route of administration:	Oral

Proprietary confidential information © 2020 Boehringer Ingelheim International GmbH or one or more of its affiliated companies

4.1.2 Selection of doses in the trial and dose modifications

Empagliflozin 10 mg and 25 mg are approved for the treatment of T2DM.

Empagliflozin exerts its effect by promoting glucosuria and consequent haemodynamic changes associated with diuresis, improvement in arterial stiffness, blood pressure lowering effect with no increase in heart rate and reduction in heart rate multiplied by pressure product, an index of myocardial oxygen consumption. These modes of actions support the scientific rationale of testing empagliflozin in patients with HF.

In the EMPA-REG-OUTCOME trial both doses were administered to patients with T2DM and showed to be equally effective in reducing CV death, HHF, and composite of HHF or CV death in patients with HF at baseline.

In a subgroup analysis, empagliflozin improved the main outcome of CV death and HHF with a similar magnitude in patients with low or high levels of HbA1c at baseline and irrespective of the dose of empagliflozin [P18-10152]. This indicates the risk reduction for this outcome is independent of the degree of glycaemic control at baseline, suggesting that these benefits can be achieved with the 10 mg dose similar to the 25 mg dose in the non-diabetic population. The mechanism of action is supported by studies in healthy volunteers where both doses were associated with a glucosuria of about 50g per day.

Given the lower exposure with empagliflozin 10 mg and similar general safety, and similar observed CV effects for both doses, empagliflozin 10 mg once daily has been selected as the dose in this trial.

For further details see current version of the IB [c01678844].

4.1.3 Method of assigning patients to treatment groups

After the assessment of all inclusion and exclusion criteria, each eligible patient will be randomised to receive empagliflozin 10 mg, or matching placebo according to a randomisation plan in a 1:1 ratio, stratified according to HF status (de novo or decompensated chronic) at Visit 2a via Interactive Response Technology (IRT). Note that the medication number is different from the patient number (the latter is generated during screening via the IRT System).

To facilitate the use of the IRT, the Investigator or delegate will receive a manual including all necessary instructions for using the system. A copy of the manual will be available in the ISF.

Patient assignment to a treatment group will be determined by a computer generated random sequence. Access to the randomisation code will be controlled and documented - for further details please refer to Section 4.1.5.1. and 4.1.5.2.

Using this procedure, relevant parties will be blinded to the treatment group assignment.

Proprietary confidential information © 2020 Boehringer Ingelheim International GmbH or one or more of its affiliated companies

4.1.4 Drug assignment and administration of doses for each patient

Patients who qualify will be randomised to one of the treatment groups described in Section 4.1.1. Trial medication will be dispensed by the pharmacist or the investigator in a double-blind manner.

IRT will be used to allocate trial medication to patients. At Visit 2a patients will be assigned one medication kit for a 30 days treatment period and 5 days of reserve. At Visit 4 patients will be assigned two medication kits for 30 days treatment period and 5 days of reserve each.

During the COVID-19 pandemic, there might be situations that would not allow a patient to come to the site for the study visit. If the investigator judges it as favourable and safe to continue trial medication, trial medication might be shipped from the site to the patient (for more details see Sections 6.1, 8.1 and 10.5).

Treatment starts on the day of Visit 2a and ends on the day of Visit 5 (or early discontinuation visit). For further details regarding packaging please refer to Section 4.1.6.

From the start of the treatment period patients will be instructed to take the trial medication once daily with a glass of water. Empagliflozin can be taken with or without food.

To ensure a dose interval of about 24 hours, the medication should be taken in the morning at approximately the same time every day including the days of study visits. If a dose is missed by more than 12 hours, that dose should be skipped and the next dose should be taken as scheduled. No double doses should be taken.

In case trial medication was discontinued for a temporary reason or by the patient without a medical indication, trial treatment can be resumed at any time until Visit 5 (EoT). Trial medication should be restarted if and as soon as medically justified (see also Section 3.3.4.1 and 4.2.1).

4.1.5 Blinding and procedures for unblinding

4.1.5.1 Blinding

Patients, investigators, central reviewers, and everyone involved in trial conduct or analysis or with any other interest in this double-blind trial will remain blinded with regard to the randomised treatment assignments until after database lock.

The access to the randomisation code will be restricted until its release for analysis.

The DMC will be provided with unblinded data in order to allow them to review efficacy and safety and to fulfil their tasks as outlined in the data monitoring committee charter. An independent team, not otherwise involved in the conduct of the trial, will provide the unblinded results to the DMC.

4.1.5.2 Unblinding and breaking the code

Emergency unblinding will be available to the investigator via IRT. It must only be used in an emergency situation when the identity of the trial drug must be known to the investigator

Proprietary confidential information © 2020 Boehringer Ingelheim International GmbH or one or more of its affiliated companies

in order to provide appropriate medical treatment or otherwise assure safety of trial participants. The reason for unblinding must be documented in the source documents and/or appropriate CRF page.

Due to the requirements to report Suspected Unexpected Serious Adverse Reactions (SUSARs), it may be necessary for a representative from BI's Pharmacovigilance group to access the randomisation code for individual patients during trial conduct. The access to the code will only be given to authorised Pharmacovigilance (PV) representatives for processing in the PV database system and not be shared further.

4.1.6 Packaging, labelling, and re-supply

The investigational medicinal products will be provided by BI or a designated Contract Research Organisation (CRO). They will be packaged and labelled in accordance with the principles of Good Manufacturing Practice (GMP). Re-supply to the sites will be managed via an IRT system, which will also monitor expiry dates of supplies available at the sites.

For details of packaging and the description of the label, refer to the ISF.

4.1.7 Storage conditions

Drug supplies will be kept in their original packaging and in a secure limited access storage area according to the recommended storage conditions on the medication label. A temperature log must be maintained for documentation.

If the storage conditions are found to be outside the specified range, the Clinical Research Associate (CRA) or the Clinical Trial Manager (CT Manager), as provided in the list of contacts, must be contacted immediately.
If the storage conditions are found to be outside the specified range, the process outlined in the ISF should be followed.

4.1.8 Drug accountability

The investigator or designee will receive the investigational drugs delivered by the sponsor when the following requirements are fulfilled:

- Approval of the clinical trial protocol by the Institutional Review Board (IRB) / ethics committee (EC),
- Availability of a signed and dated clinical trial contract between the sponsor and the investigational site,
- Approval/notification of the regulatory authority, e.g. competent authority,
- Availability of the curriculum vitae of the Principal Investigator,
- Availability of a signed and dated clinical trial protocol,
- Availability of the proof of a medical license for the Principal Investigator (if applicable),
- Availability of FDA Form 1572 (if applicable).

Proprietary confidential information © 2020 Boehringer Ingelheim International GmbH or one or more of its affiliated companies

Investigational drugs are not allowed to be used outside the context of this protocol. They must not be forwarded to other investigators or clinics. Patients should be instructed to return unused investigational drug.

The investigator or designee must maintain records of the product's delivery to the trial site, the inventory at the site, the use by each patient, and the return to the sponsor or warehouse / drug distribution centre or alternative disposal of unused products. If applicable, the sponsor or warehouse / drug distribution centre will maintain records of the disposal.

These records will include dates, quantities, batch / serial numbers, expiry ('use- by') dates, and the unique code numbers assigned to the investigational medicinal product and trial patients. The investigator or designee will maintain records that document adequately that the patients were provided the doses specified by the Clinical Trial Protocol (CTP) and reconcile all investigational medicinal products received from the sponsor. At the time of return to the sponsor <and/or> appointed CRO, the investigator or designee must verify that all unused or partially used drug supplies have been returned by the clinical trial patient and that no remaining supplies are in the investigator's possession.

4.2 OTHER TREATMENTS, EMERGENCY PROCEDURES, RESTRICTIONS

4.2.1 Other treatments and emergency procedures

The use of medication for the treatment of HF will be at the discretion of the Investigator and should be in accordance with local/international guidelines.

All concomitant (additional) medications and other therapies should be recorded on the appropriate pages of the electronic Case Report Form (eCRF).

Concomitant antidiabetic medications should be adjusted individually as clinically indicated by the patient's usual diabetes care provider. Additional guidance is provided in the IB. Restrictions of antidiabetic background therapy are described in Section 4.2.2.

Patients without a diagnosis of DM experiencing repeated or severe symptoms such as nervousness, sweating, intense hunger, trembling, weakness and palpitations should contact the Investigator or other healthcare professional, as these symptoms might be suggestive of hypoglycaemia. In the case of hypoglycaemia, in patients with or without DM, that may put the patient at risk (e.g. repeated symptomatic hypoglycaemia or severe hypoglycaemia), appropriate care should be provided at the discretion of the Investigator.

Special attention must be paid to the prevention of ketoacidosis. All patients must be made aware of this risk and need to be instructed to contact the Investigator or other healthcare professional in case of symptoms of metabolic acidosis, ketoacidosis and DKA.

Cases of DKA have been reported in patients treated with empagliflozin, including fatal cases. In a number of reported cases, the presentation of the condition was atypical with only moderately increased blood glucose values; below 14 mmol/l (250 mg/dl).

Proprietary confidential information © 2020 Boehringer Ingelheim International GmbH or one or more of its affiliated companies

The risk of DKA must be considered in the event of non-specific symptoms such as nausea, vomiting, anorexia, abdominal pain, excessive thirst, difficulty breathing, confusion, unusual fatigue or sleepiness.

Patients should be assessed and treated for ketoacidosis immediately according to local guidelines if these symptoms occur, regardless of blood glucose level. If ketoacidosis is suspected, the trial medication should be discontinued, the patient should be evaluated, and prompt treatment should be initiated.

Patients who may be at higher risk of ketoacidosis while taking empagliflozin include patients on a very low carbohydrate diet (as the combination may further increase ketone body production), patients with an acute illness, pancreatic disorders suggesting insulin deficiency (e.g. Type 1 diabetes mellitus (T1DM), history of pancreatitis or pancreatic surgery), insulin dose reduction (including insulin pump failure), alcohol abuse, severe dehydration, and patients with a history of ketoacidosis Empagliflozin should be used with caution in these patients. In patients requiring insulin, caution should be taken when the dose of insulin is reduced.

In clinical situations known to predispose to ketoacidosis (e.g. prolonged fasting due to acute illness or surgery), the Investigator should consider monitoring for ketoacidosis and temporarily discontinue the trial medication.

There are no trial specific emergency procedures to be followed.

In patients with heart failure who receive empagliflozin, elderly patients with heart failure and in case of conditions that may lead to fluid loss (e.g. gastrointestinal illness), careful monitoring of volume status (e.g. physical examination, laboratory tests including haematocrit), blood pressure measurement and electrolytes is recommended since empagliflozin may potentially lead to hypotension. Temporary interruption of the study drug should be considered until the fluid loss is corrected.

Temporary interruption of empagliflozin should be considered in patients with complicated urinary tract infections.

4.2.2 Restrictions

4.2.2.1 Restrictions regarding concomitant treatment

The use of any SGLT-2 inhibitors or combined SGLT-1 and 2 inhibitors except the blinded trial medication is prohibited during the course of the trial. This also includes the 7 days period between the End of Treatment (EoT) and the Follow-up Visit.

If any restricted treatment is given during the conduct of the trial, the trial medication can be discontinued temporarily, or if needed permanently.

If the patient is in need of any additional treatment during this period, this may be given at the discretion of the Investigator. The patient can still remain on trial medication.

Proprietary confidential information © 2020 Boehringer Ingelheim International GmbH or one or more of its affiliated companies

4.2.2.2 Restrictions on diet and life style

None.

4.2.2.3 Contraception requirements

Women of childbearing potential must use highly effective methods of birth control per ICH M3 (R2) that result in a low failure rate of less than 1% per year when used consistently and correctly. A list of contraception methods meeting these criteria is provided in the patient information.

4.3 TREATMENT COMPLIANCE

Patients are requested to bring all remaining trial medication including empty package material with them when attending visits 3, 4 and 5 (EoT).

Based on tablet counts, treatment compliance will be calculated as shown in the formula below. Compliance will be verified by the CRA authorised by the sponsor.

$$\text{Treatment compliance (\%)} = \frac{\text{Number of tablets actually taken} \times 100}{\text{Number of tablets which should have been taken as directed by the investigator}}$$

If the number of doses taken is not between 80-120%, site staff will explain to the patient the importance of treatment compliance. However, randomised patients will not be discontinued for poor compliance without prior discussion with the monitor or designee.

Patients who are not compliant with their medication should again be carefully interviewed and again re-informed about the purpose and the conduct of the trial.

Proprietary confidential information © 2020 Boehringer Ingelheim International GmbH or one or more of its affiliated companies

5. ASSESSMENTS

5.1 ASSESSMENT OF EFFICACY

5.1.1 Clinical Benefit

Clinical benefit is a composite of death, HFEs (including HHFs, urgent heart failure visits and unplanned outpatient visits) and KCCQ-TSS.

Investigators will evaluate patients at each study visit and assess for any interim hospitalisation, urgent heart failure visit or unplanned outpatient visit and record the patient response on the dedicated CRFs. Death and HFEs will be categorised by the investigator according to pre-specified criteria [R19-3245] (more details can be found in the ISF). Attempts should be made to obtain supporting medical records for these healthcare interactions.

KCCQ is a commonly used 23 item self-administered questionnaire designed to evaluate physical limitations, symptoms (frequency, severity, and changes over time), social limitations, self-efficacy, and quality of life in patients with HF (refer to Appendix 10.1).

The questionnaire takes less than 15 minutes to complete and will be assessed according to the Flow Chart. Please refer to Section 6.2.2 for order of completion of patient reported outcome measures.

The Investigator (or designated site-personnel) should ensure that the patient has access to a quiet area at the site where he/she can be left alone to record her/his response in the questionnaire. In instances where a patient cannot give or decide upon a response, no response should be recorded. The Investigator (or designated site-personnel) should check that all items have been completed by the patient, but the response to each item should not be scrutinised. Instructions to patients are included in the questionnaire. The respective procedure for illiterate patients (if included) is described in the Appendix 10.2.

5.1.2 NT-proBNP and BNP

NT-proBNP or BNP measurements available from standard of care (local laboratory) will be used in all patients to determine eligibility.
NT-proBNP will be obtained at all visits from Visit 2 until Visit 5 (see Flow Chart) using central laboratory to investigate a potential effect of the trial medication.

5.1.3 Clinical Congestion Score

Patient's congestion will be assessed using a clinician-based outcome assessment of 6 different signs and symptoms: dyspnoea, orthopnoea, fatigue, jugular venous pressure (JVP) (as assessed by the investigator), rales, and oedema. Each category will be assessed through a four-measure questionnaire which will be further converted to a standardized 4-point scale ranging from 0 to 3 as shown in Table 5.1.3: 1.

Proprietary confidential information © 2020 Boehringer Ingelheim International GmbH or one or more of its affiliated companies

Table 5.1.3: 1 Clinical congestion score

**Signs/Symptoms**	**0**	**1**	**2**	**3**
Dyspnoea	None	Seldom	Frequent	Continuous
Orthopnoea	None	Seldom	Frequent	Continuous
Fatigue	None	Seldom	Frequent	Continuous
JVP (cm H2O) (Jugular Venous Pressure[5])	≤6	6< JVP < 10	10≤ JVP <15	≥15
Rales	None	Bases	From base to <50%	From base to >50%
Oedema	Absent/ trace	Slight	Moderate	Marked

The Clinical Congestion Score will be completed according to the Flow Chart. Please refer to Section 6.2.2 for preferred order of completion of assessments.

5.1.4 New York Heart Association classification

The NYHA functional classification will be used to classify the severity of the patients' heart failure. The investigator should place the patients in one of the four categories based on how limited their physical activity are.

Table 5.1.4: 1 New York Heart Association classification

**Class**	**Patient symptoms**
I	No limitation of physical activity. Ordinary physical activity does not cause undue fatigue, palpitation, dyspnoea (shortness of breath)
II	Slight limitation of physical activity. Comfortable at rest. Ordinary physical activity results in fatigue, palpitation, dyspnoea (shortness of breath)
III	Marked limitation of physical activity. Comfortable at rest. Less than ordinary activity causes fatigue, palpitation, or dyspnoea
IV	Unable to carry on any physical activity without discomfort. Symptoms of heart failure at rest. If any physical activity is undertaken, discomfort increases

The classification of patient's physical activity according to NYHA will be performed at Visits 2a, 3, 4, 5 and at early discontinuation.

[5] Jugular venous pressure should be estimated by adding 5 cm H_2O to the vertical distance of the peak jugular venous pulsation from the sternal angle

Proprietary confidential information © 2020 Boehringer Ingelheim International GmbH or one or more of its affiliated companies

5.1.5 Patient Global Impression of Severity of Heart Failure Symptoms

Patient Global Impression of Severity of Heart Failure Symptoms (PGI-S) is a 1-item questionnaire to assess patient's impression of symptoms severity, specifically: shortness of breath, fatigue and swelling.

The PGI-S asks the patient to choose one response that best describes how his/her Heart Failure Symptoms, specifically: shortness of breath, fatigue and swelling are now on a 5-point scale:

- Not at all (1)
- Mild (2)
- Moderate (3)
- Severe (4)
- Very severe (5)

Please refer to Section 6.2.2 for order of completion of patient reported outcome measure and Appendix 10.3.

5.2 ASSESSMENT OF SAFETY

5.2.1 Physical examination

A complete physical examination will be performed at the time points specified in the Flow Chart. Complete physical examination will include general appearance as well as evaluation of organ systems including an assessment of the cardiovascular system.

The results must be included in the source documents available at the site.

5.2.2 Vital signs

Vital signs will be evaluated at the time points specified in the Flow Chart, prior to blood sampling. This includes systolic and diastolic blood pressure and pulse rate (electronically or by palpation count for 1 minute) in a seated position after 5 minutes of rest.

The preferred method for blood pressure measurement is by a standard mercury sphygmomanometer. If a standard mercury sphygmomanometer is not available, alternative devices recommended by website www.dableducational.org may be used or devices approved for use by the appropriate national agency/ies.

At visit 2a, after the patient has rested quietly in the seated position for five minutes, blood pressure should be taken in both arms. If the pressures differ by more than 10 mmHg (as for example in the presence of a subclavian steal syndrome), the pressure from the arm with the higher pressure (systolic or diastolic) should be entered in the eCRF and this arm should be used for subsequent measurements. The same method and, if possible, the same device should be used throughout the trial for a patient.

Proprietary confidential information © 2020 Boehringer Ingelheim International GmbH or one or more of its affiliated companies

Blood pressure measurements should be recorded to the nearest 2 mmHg only when measured with a manual sphygmomanometer; when digital devices are used the value from the device should be rounded to the nearest 1 mmHg.

For eCRF entry, decimal places should be rounded to integers (e.g. a DBP of 94.5 would be rounded to 95 mmHg and a SBP of 109.4 would be rounded to 109 mmHg).

The results must be included in the source documents available at the site.

5.2.3 Body weight and height

Measurements of body weight and height will be performed at the time points specified in the Flow Chart.

Body weight should be measured as follows:

- after the urine sampling (weight after bladder voiding),
- shoes and coat/jackets should be taken off, and
- pockets should be emptied of heavy objects (i.e. keys, coins etc.).

5.2.4 Safety laboratory parameters

Safety laboratory parameters needed for assessing patients eligibility including NT-proBNP/BNP will be assessed based on local lab.

Safety laboratory parameters to be assessed during the trial are listed in Table 5.2.4: 1. For the sampling time points please see the Flow Chart.

All analyses will be performed by a central laboratory, the respective reference ranges will be provided in the ISF. Patients do not have to be fasted for the blood sampling for the safety laboratory.

Instructions regarding sample collection, sample handling/ processing and sample shipping are provided in the Laboratory Manual in the ISF.
The central laboratory will send reports to the investigator. It is the responsibility of the investigator to evaluate the laboratory reports. Clinically relevant abnormal findings as judged by the investigator will be reported as adverse events (please refer to Section 5.2.7).

In case the criteria for hepatic injury are fulfilled, a number of additional measures will be performed (please see Section 5.2.7.1 and the DILI Checklist provided in the ISF and electronic data capture (eDC) system. The amount of blood taken from the patient concerned will be increased due to this additional sampling.
The central laboratory will transfer the results of the analysis to the sponsor.

Proprietary confidential information © 2020 Boehringer Ingelheim International GmbH or one or more of its affiliated companies

Table 5.2.4: 1 Safety laboratory tests

Haematology
Hematocrit<br>Haemoglobin (Hb)<br>Reticulocyte Count (reflex test if Hb outside normal range)<br>Red Blood Cells (RBC) / Erythrocytes<br>WBC / Leukocytes<br>Platelet Count / Thrombocytes
Clinical chemistry
Albumin<br>Alkaline phosphatase<br>γ-GT (gamma-glutamyl transferase) reflex test triggered by elevated alkaline phosphatase on two sequential measures<br>ALT (alanine transaminase, SGPT)<br>AST (aspartate transaminase, SGOT)<br>Bicarbonate<br>Bilirubin total, fractionated if increased<br>Chloride<br>Creatinine<br>Creatine kinase (CK)<br>High sensitivity Troponin I (reflex tests, if CK is elevated)<br>Glucose<br>Potassium<br>Protein total<br>Sodium<br>Urea (blood urea nitrogen (BUN)<br>Uric acid
Urine
Urinalysis: test including for example (but not limited to) ketones semi-quantitative measurement, white blood cells, blood<br>(glucose measurement in urine will not be performed)

5.2.4.1 Renal Function

The estimated glomerular filtration rate (eGFR) will be derived from serum creatinine values, age, sex and race based on the CKD-EPIcr equation [R12-1392].

Proprietary confidential information © 2020 Boehringer Ingelheim International GmbH or one or more of its affiliated companies

5.2.4.2 Pregnancy Testing

Pregnancy testing (urine) will be performed in female patients of child bearing potential according to the time points indicated in the Flow Chart. Pregnancy kits will be provided by the Central Laboratory. For reporting of pregnancy event refer to Section 5.2.7.2.

5.2.4.3 Criteria for hypoglycaemic events

All symptomatic hypoglycaemia events, or severe hypoglycaemias (e.g. if the patient required assistance of another person), or any hypoglycaemia episode with glucose values < 54 mg/dl (< 3.0 mmol/l), or if the investigator considered the event to be an AE should be documented as an AE "hypoglycaemic event". In non-diabetic or pre-diabetic patients, the investigator should consider and rule out other alternative causes for such symptoms and can perform blood glucose levels to confirm the diagnosis of hypoglycaemia.

5.2.4.4 Urinary tract infection and genital infections

Patients having a history of chronic/recurrent urinary tract infections (UTI) or genital infections, or an acute episode of UTI or genital infection at screening will be identified and this condition has to be documented as medical history or baseline condition in the eCRF, respectively.

For documentation of symptomatic acute UTI during trial conduct, a urine culture sample has to be taken and sent to central lab for confirmation of the diagnosis.

5.2.5 Electrocardiogram

12-lead ECGs will be performed at Visits 2a and 5 (EoT) as indicated in the Flow Chart. The investigator or a designee will evaluate whether the ECG is normal or abnormal and assess clinical relevance. ECGs may be repeated for quality reasons and a repeated recording used for analysis.

Additional ECGs may be recorded for safety reasons. Dated and signed printouts of ECG with findings should be documented in patient's medical record.

Clinically relevant abnormal findings will be reported either as baseline condition (if identified at the screening visit) or otherwise as AEs and will be followed up and/or treated as medically appropriate.

5.2.6 Other safety parameters

There are no additional safety parameters in the trial.

Proprietary confidential information © 2020 Boehringer Ingelheim International GmbH or one or more of its affiliated companies

5.2.7 Assessment of adverse events

5.2.7.1 Definitions of AEs

5.2.7.1.1 Adverse event

An AE is defined as any untoward medical occurrence in a patient or clinical investigation subject administered a medicinal product and which does not necessarily have to have a causal relationship with this treatment.

An AE can therefore be any unfavourable and unintended sign (including an abnormal laboratory finding), symptom, or disease temporally associated with the use of a medicinal product, whether or not considered related to the medicinal product.

The following should also be recorded as an AE in the CRF and BI SAE form (if applicable):

- Worsening of the underlying disease or of other pre-existing conditions.
- Changes in vital signs, ECG, physical examination and laboratory test results, if they are judged clinically relevant by the investigator.

If such abnormalities already exist prior to trial inclusion, they will be considered as baseline conditions and should be collected in the eCRF only.

5.2.7.1.2 Serious adverse event

A serious adverse event (SAE) is defined as any AE, which fulfils at least one of the following criteria:

- results in death,
- is life-threatening, which refers to an event in which the patient was at risk of death at the time of the event; it does not refer to an event that hypothetically might have caused death if more severe,
- requires inpatient hospitalisation or prolongation of existing hospitalisation,
- results in persistent or significant disability or incapacity,
- is a congenital anomaly / birth defect,
- is deemed serious for any other reason if it is an important medical event when based on appropriate medical judgement which may jeopardise the patient and may require medical or surgical intervention to prevent one of the other outcomes listed in the above definitions. Examples of such events are intensive treatment in an emergency room or at home for allergic bronchospasm, blood dyscrasias or convulsions that do not result in hospitalisation or development of dependency or abuse.

5.2.7.1.3 AEs considered "Always Serious"

In accordance with the European Medicines Agency initiative on Important Medical Events, Boehringer Ingelheim has set up a list of AEs, which by their nature, can always be considered to be "serious" even though they may not have met the criteria of an SAE as defined above.

Proprietary confidential information © 2020 Boehringer Ingelheim International GmbH or one or more of its affiliated companies

The latest list of "Always Serious AEs" can be found in the eDC system. A copy of the latest list of "Always Serious AEs" will be provided upon request. These events should always be reported as SAEs as described in Section 5.2.7.2.

Cancers of new histology and exacerbations of existing cancer must be classified as a serious event regardless of the time since discontinuation of the drug and must be reported as described in 5.2.7.2, subsections "AE Collection" and "AE reporting to sponsor and timelines".

5.2.7.1.4 Adverse events of special interest

The term adverse events of special interest (AESI) relates to any specific AE that has been identified at the project level as being of particular concern for prospective safety monitoring and safety assessment within this trial, e.g. the potential for AEs based on knowledge from other compounds in the same class. AESIs need to be reported to the sponsor's Pharmacovigilance Department within the same timeframe that applies to SAEs, please see Section 5.2.7.2.2.

The following are considered as AESIs:

<u>Hepatic injury</u>

A hepatic injury is defined by the following alterations of hepatic laboratory parameters:

- an elevation of AST (Aspartate Aminotransferase) and/or ALT (Alanine Aminotransferase) ≥3 fold ULN combined with an elevation of total bilirubin ≥2 fold ULN measured in the same blood draw sample, or
- Aminotransferase (ALT, and/or AST) elevations ≥5 fold ULN.

These lab findings constitute a hepatic injury alert and the patients showing these lab abnormalities need to be followed up according to the eDC system.

In case of clinical symptoms of hepatic injury (icterus, unexplained encephalopathy, unexplained coagulopathy, right upper quadrant abdominal pain, etc.) without lab results (ALT, AST, total bilirubin) available, the investigator should make sure these parameters are analysed, if necessary in an unscheduled blood test. Should the results meet the criteria of hepatic injury alert, the procedures described in the DILI checklist should be followed.

<u>Decreased renal function</u>

Decreased renal function is defined by a creatinine value showing a ≥ 2 fold increase from baseline and is above the Upper Limit of Normal (ULN).

For the AESI "decreased renal function" patients need to be followed up appropriately based on local clinical guidance.

Proprietary confidential information © 2020 Boehringer Ingelheim International GmbH or one or more of its affiliated companies

Ketoacidosis

If metabolic acidosis, ketoacidosis and DKA is suspected further investigations should be done according to the medical judgment and the clinical course until a diagnosis is made and/or the patient is recovered.

Due to its mechanism of action, empagliflozin may potentially modify the clinical presentation of ketoacidosis which may occur at lower plasma glucose levels in patients with DM and potentially also in non-diabetic patient population. The diagnosis of ketoacidosis in these patients can be based on arterial pH ≤7.30, serum bicarbonate levels <15 mmol/L and measurement of serum beta-hydroxybutrate levels. Other diagnostic criteria which can support the diagnosis of ketoacidosis are urine ketones and anion gap >10 mmol/L.

Investigators should note that not all criteria mentioned above need to apply for the diagnosis of ketoacidosis, and clinical judgment should also be taken into consideration.

5.2.7.1.5 Intensity (severity) of AEs

The intensity (severity) of the AE should be judged based on the following:

Mild:	Awareness of sign(s) or symptom(s) that is/are easily tolerated.
Moderate:	Sufficient discomfort to cause interference with usual activity.
Severe:	Incapacitating or causing inability to work or to perform usual activities.

5.2.7.1.6 Causal relationship of AEs

Medical judgement should be used to determine whether there is a reasonable possibility of a causal relationship between the adverse event and the given study treatment, considering all relevant factors, including pattern of reaction, temporal relationship, de-challenge or re-challenge, confounding factors such as concomitant medication, concomitant diseases and relevant history.

Arguments that may suggest that there is a reasonable possibility of a causal relationship could be:

- The event is consistent with the known pharmacology of the drug.
- The event is known to be caused by or attributed to the drug class.
- A plausible time to onset of the event relative to the time of drug exposure.
- Evidence that the event is reproducible when the drug is re-introduced.
- No medically sound alternative aetiologies that could explain the event (e.g. pre-existing or concomitant diseases, or co-medications).
- The event is typically drug-related and infrequent in the general population not exposed to drugs (e.g. Stevens-Johnson syndrome).
- An indication of dose-response (i.e. greater effect size if the dose is increased, smaller effect size if dose is reduced).

Proprietary confidential information © 2020 Boehringer Ingelheim International GmbH or one or more of its affiliated companies

Arguments that may suggest that there is no reasonable possibility of a causal relationship could be:

- No plausible time to onset of the event relative to the time of drug exposure is evident (e.g. pre-treatment cases, diagnosis of cancer or chronic disease within days / weeks of drug administration; an allergic reaction weeks after discontinuation of the drug concerned).
- Continuation of the event despite the withdrawal of the medication, taking into account the pharmacological properties of the compound (e.g. after 5 half-lives). Of note, this criterion may not be applicable to events whose time course is prolonged despite removing the original trigger.
- Additional arguments amongst those stated before, like alternative explanation (e.g. situations where other drugs or underlying diseases appear to provide a more likely explanation for the observed event than the drug concerned).
- Disappearance of the event even though the trial drug treatment continues or remains unchanged.

5.2.7.2 Adverse event collection and reporting

5.2.7.2.1 AE Collection

The investigator shall maintain and keep detailed records of all AEs in the patient files.

The following must be collected and documented on the appropriate CRF(s) by the investigator:

- From signing the informed consent onwards until the individual patient's end of trial participation (End of Study (EOS)):
  all AEs (serious and non-serious) and all AESIs.
- After the individual patient's end of trial:
  the investigator does not need to actively monitor the patient for new AEs but should only report any occurrence of cancer and trial treatment related SAEs and trial treatment related AESIs of which the investigator may become aware of by any means of communication, e.g. phone call. Those AEs should be reported on the BI SAE form (see Section 5.2.7.2.2), but not on the CRF.

For some types of AEs additional information will be collected in the eCRF due to the nature of the event and mechanisms of action of the trial medication. These listed AEs are distinct from AESI. The list of types of AEs for which additional information will be collected may change during the trial based on potential new knowledge about the safety profile of empagliflozin:

- Hypoglycaemic event
- Bone fracture
- Hypotension

5.2.7.2.2 AE reporting to the sponsor and timelines

The investigator must report SAEs, AESIs, and non-serious AEs which are relevant for the reported SAE or AESI, on the BI SAE form immediately (within 24 hours) to the sponsor's

Proprietary confidential information © 2020 Boehringer Ingelheim International GmbH or one or more of its affiliated companies

unique entry point (country specific contact details will be provided in the ISF). The same timeline applies if follow-up information becomes available. In specific occasions, the investigator could inform the sponsor upfront via telephone. This does not replace the requirement to complete and send the BI SAE form.

With receipt of any further information to these events, a follow-up SAE form has to be provided. For follow-up information the same rules and timeline apply as for initial information. All (S)AEs, including those persisting after individual patient's end of trial must be followed up until they have resolved, have been assessed as "chronic" or "stable" , or no further information can be obtained.

5.2.7.2.3 Pregnancy

In rare cases, pregnancy might occur in a clinical trial. Once a patient has been enrolled in the clinical trial and has taken trial medication, the investigator must report any drug exposure during pregnancy in a trial participant immediately (within 24 hours) by means of Part A of the Pregnancy Monitoring Form to the sponsor's unique entry point.

The outcome of the pregnancy associated with the drug exposure during pregnancy must be followed up and reported to the sponsor's unique entry point on the Pregnancy Monitoring Form for Clinical Studies (Part B).
The ISF will contain the Pregnancy Monitoring Form for Clinical Studies (Part A and B).

As pregnancy itself is not to be reported as an AE, in the absence of an accompanying SAE and/or AESI, only the Pregnancy Monitoring Form for Clinical Studies and not the SAE form is to be completed. If there is an SAE and/or AESI associated with the pregnancy an SAE form must be completed in addition.

5.3 DRUG CONCENTRATION MEASUREMENTS AND PHARMACOKINETICS

This study will not analyse pharmacokinetic or pharmacodynamics parameters.

5.4 ASSESSMENT OF BIOMARKER(S)

Established biomarkers of efficacy and safety are described and discussed in Section 5.1 and 5.2.

This study will not analyse exploratory biomarkers.

5.5 BIOBANKING

Not applicable.

Proprietary confidential information © 2020 Boehringer Ingelheim International GmbH or one or more of its affiliated companies

5.6 OTHER ASSESSMENTS

5.6.1 Health Care Resource Utilisation (HCRU)

HCRU will be captured via interview with the patient and verified against medical records where available and entered in the eCRF at the visits specified in the Flowchart. Information on utilization of following resources will be collected:

- All-cause hospital admissions (first and recurrent).
- Hospital admission due to worsening of heart failure (first and recurrent).
- All-cause emergency room visits (first and recurrent).
- Emergency room visits due to worsening of heart failure (requiring i.v. diuretic therapy) (first and recurrent).
- Any unscheduled outpatient visits (first and recurrent).
- Unscheduled outpatient visits related to heart failure (first and recurrent).
- Length of hospital stay(s) (initial and recurrent).
- Length of ICU stay during hospital stay (initial and recurrent).
- Intensification of diuretic therapy (adding a new diuretic, increase of dose).

5.7 APPROPRIATENESS OF MEASUREMENTS

All measurements performed during this trial are standard measurements and will be performed in order to monitor safety aspects, and to determine empagliflozin efficacy and safety in an appropriate way.

For more details on NT-proBNP, please refer to Section 3.2.

The scheduled measurements are appropriate to see drug induced changes in vital signs, standard laboratory values, the biomarker NT-proBNP, and in ECG. The primary and secondary endpoints are accepted for evaluation of efficacy, safety and tolerability of an oral HF drug and they are widely used in respective pivotal phase III HF studies.

Health related quality of life questionnaires (specifically the KCCQ) are a necessary part for this phase III trial in order to collect data on patient related outcomes (as part of the primary endpoint).

Therefore, the appropriateness of all measurements applied in this trial is given.

Proprietary confidential information © 2020 Boehringer Ingelheim International GmbH or one or more of its affiliated companies

6. INVESTIGATIONAL PLAN

6.1 VISIT SCHEDULE

All patients are to adhere to the visit schedule as specified in the Flow Chart. If any visit has to be rescheduled, subsequent visits should follow the original visit date schedule. The trial medication packs contain sufficient medication to allow for the allowed time windows.

All trial visits should take place at approximately the same time of day and preferably before noon.

During the COVID-19 pandemic, there might be situations when patients might not be able to come to the site for the scheduled visit. This might be e.g. due to restrictions set by authorities or by the investigator site/institution, because the patient is quarantined, or because of any patient specific situation that the investitator judges as being not safe for the patient to come to the site.

For details on potential modifications of the trial conduct related to the COVID-19 pandemic, please refer to Section 10.5.

6.2 DETAILS OF TRIAL PROCEDURES AT SELECTED VISITS

Please refer to the Flow Chart and Section 5 for details of the procedures performed at each visit.

6.2.1 Screening and run-in period(s)

Due to the short time window when patients can be randomised (between 24 hours after hospitalisation and no later than 5 days), potential study patients should be identified as soon as possible and their improvement followed up accordingly (pre-screening). Potential study patients should be approached to assess their interest in volunteering as a participant in this trial as soon as possible.

Screening Period

The investigations for the main diagnosis for inclusion are to be completed and documented per standard of care as a prerequisite to consideration for study participation. Patients will be included in the study based on local BNP or NT-proBNP and other available laboratory measurements.

Following informed consent, the patient will undergo Visit 1/screening assessments as indicated in the Flow Chart. The assessments must all fall within the acceptable screening visit window but do not need to be performed on the same day. The patient should be registered in IRT as a screened patient.

Re-screening within the same hospitalisation and current episode of acute decompensation is not allowed. Nevertheless, if a patient who failed screening before has another episode of

Proprietary confidential information © 2020 Boehringer Ingelheim International GmbH or one or more of its affiliated companies

acute decompensation that leads to a new hospitalisation, patient can be screened again and considered for the study.

Medical History:

Medical History will be documented using pre-specified categories as given in the eDC system. For detailed requirements please see Section 8.3.1.

Baseline Conditions

Ongoing conditions/diagnosis from the Medical History questionnaire will be documented as Baseline Conditions.

If the patient meets the entry criteria, Visit 2a should occur as soon as possible once it has been confirmed that the patient is eligible to continue. If the patient does not meet the entry criteria, (i.e. fail to meet one or more of the inclusion criteria, and/or meet one or more of the exclusion criteria) following Visit 1 procedures, they should be registered as a screen failure in IRT.

6.2.2 Treatment period(s)

After a final check of all in- and exclusion criteria, patients eligible will be randomised at Visit 2a using IRT. Randomisation can occur at the same day as the screening visit. All Visit 2a assessments must be performed before the first dose is taken in hospital. Assessments from Visit 2a will be used as baseline values.

Patients will undergo visit assessments as indicated in the Flow Chart.

Patients will have further visits with a reduced number of assessments two and four days thereafter if they are still hospitalised (Visits 2b and 2c at Day 3 and 5).

The patients will return to the clinic for regularly scheduled Visits 3, 4 and 5 (EoT) on days 15, 30 and 90 respectively after randomisation as specified in the Flow Chart. These on-site visits will assess the occurrence of safety and efficacy endpoints, trial medication compliance (Visit 3, 4 and 5), concomitant therapy or intervention. The assessments at these visits can be performed post-dose. Patients should be instructed to take their medication on the morning of their visits at home like all days. Visits should be routinely scheduled at approximately the same time of day for each visit.

All other medications the patient is receiving should be taken as instructed by the physician. At any time during the treatment period, the HF background therapy is allowed to be adjusted and optimised according to local and international guidelines.
If any additional therapy is considered necessary for the patient’s welfare during the treatment period it may be given at the discretion of the Investigator (see also restrictions in Section 4.2.2).

Patient reported outcome measures (first KCCQ, then PGI – Severity – Heart Failure Symptoms questionnaire) should be done first and laboratory samples should be taken at the end of each visit. All other measures should be performed after PGI-S and before laboratory sampling.

Proprietary confidential information © 2020 Boehringer Ingelheim International GmbH or one or more of its affiliated companies

Allocation of medication kit number(s) will be managed through the IRT. Patients will be assigned medication at Visit 2a, starting treatment in hospital and will receive their trial mediation kit at discharge to take home. This kit contains sufficient medication until Visit 4. At this visit patients should return the medication and compliance check will be done. At Visit 4 new medication kits will be dispensed. Visit 5 (EoT) is the last day of treatment and the patient should return all remaining medication during this visit.

Early permanent trial medication discontinuation is only justified when any contraindications arise, or when the patient requests to stop trial medication. See Section 3.3.4.1 for details on how to handle trial medication discontinuations. An Early Discontinuation Visit should be performed with the procedures indicated in the Flow Chart.

6.2.3 Follow-up period and trial completion

A Follow-up Visit will be performed 7 days (+7 day window) after the last dose of trial medication. The assessments to be performed at the Follow-up Visit are indicated in the Flow Chart. The Follow-up Visit marks the completion of the study for the individual patient who completed the study on trial medication.

As a standard, Follow-up Visit should be performed as phone call. If considered necessary e.g. for safety reasons requiring personal follow-up, the visit can be done as a site visit.

See Section 3.3.4.1 for procedures to be followed in case a patient prematurely discontinues trial treatment.

For patients who early discontinued trial medication but followed up according to the visit schedule, Visit 5 (EoT) marks the completion of the study for the individual patient.

Proprietary confidential information © 2020 Boehringer Ingelheim International GmbH or one or more of its affiliated companies

7. STATISTICAL METHODS AND DETERMINATION OF SAMPLE SIZE

The eligible patients for this trial will be randomised to one of two treatment groups (empagliflozin or placebo) in a 1:1 ratio, stratified according to HF status (de novo or decompensated chronic HF).

The primary endpoint is clinical benefit defined in Section 7.2.2 [R16-4813 and R19-3448]. The statistical model will be a non-parametric generalised pairwise comparison within HF status strata. The variance of the win ratio will be calculated using the asymptotic normal U statistics approach [R19-3448].

The primary model will be conducted on all randomised patients.

7.1 NULL AND ALTERNATIVE HYPOTHESES

For the primary endpoint, superiority of empagliflozin vs. placebo will be evaluated with one-sided tests, at a significance level of 0.025, in the following structure:

Null hypothesis – There is no difference between the effect of placebo and the effect of empagliflozin or the effect of placebo is greater.

Alternative hypothesis – The effect of empagliflozin is greater than the effect of placebo.

Secondary and further endpoints will be evaluated in an exploratory manner.

7.2 PLANNED ANALYSES

7.2.1 General considerations

The efficacy analysis will be based on the randomised set (RS), including all randomised patients. Analyses will be performed according to the intention-to-treat principle, with the use of all available data (on and off-treatment) through the trial period. This equates to a treatment-policy style estimand. Sensitivity analyses investigating hypothetical estimands (e.g. on-treatment data only) may be specified in the Trial Statistical Analysis Plan (TSAP). If a Per Protocol Set (PPS) is required, this will also be specified in the TSAP. Important protocol deviations will be described in the TSAP.

The safety analysis will be based on the treated set (TS), which consists of all patients treated with at least one dose of trial medication.

For both efficacy and safety analyses, treatment will be evaluated as randomised.

For all endpoints, baseline will be defined as the last available measurement before start of trial medication.

Proprietary confidential information © 2020 Boehringer Ingelheim International GmbH or one or more of its affiliated companies

7.2.2 Primary endpoint analyses

The primary endpoint is clinical benefit, a composite of death, number of HFEs, time to first HFE and change from baseline in KCCQ-TSS after 90 days of treatment. This will be assessed using a win ratio approach.
All patients randomised to empagliflozin are compared to all patients randomised to placebo within their stratum.

For any two patients, a patient will win, i.e. achieve a better clinical outcome, as determined by assessing the following criteria sequentially, stopping when an advantage for either patient is shown:

1. Death within common follow-up time
   - death is worse than no death
   - earlier death is worse
   - tied, if not possible to determine

2. Number of HFEs within common follow-up time
   - more HFEs is worse
   - tied, if same number of HFEs

3. Time to first HFE within common follow-up time
   - earlier HFE is worse
   - tied, if not possible to determine

4. KCCQ-TSS change from baseline at Day 90
   - more positive change from baseline is better
   - the threshold for the difference is >= 5 for a win
   - tied, if difference < 5

Note, priority is therefore given to death over HFE, and both of these over changes in KCCQ-TSS. Below are some examples:

1. Death, e.g.:
   - Patient A dies 30 days after randomisation (loses)
   - Patient B dies 40 days after randomisation (wins)

2. If no winner based on death, number of HFEs within common follow-up time, e.g.:
   - Patient A had two HFEs (loses)
   - Patient B had one HFE (wins)

3. If no winner based on number of HFEs, time to first HFE, e.g.:
   - Patient A had an HFE 30 days after randomisation (loses)
   - Patient B had an HFE 50 days after randomisation (wins)

Proprietary confidential information © 2020 Boehringer Ingelheim International GmbH or one or more of its affiliated companies

4. If no winner based on time to first HFE, KCCQ-TSS change from baseline at Day 90, e.g.:
    - Patient A: KCCQ-TSS change from baseline at Day 90 is 5 (loses)
    - Patient B: KCCQ-TSS change from baseline at Day 90 is 11 (wins)

The implemented generalised pairwise comparisons approach compares all patients in one treatment group to all other patients within their strata in the other treatment group. The win ratio is then calculated as the total number of wins in the empagliflozin group (N_W) across all strata divided by the total number of losses (N_L). So the win ratio is = N_W / N_L.

The variance is calculated by the asymptotic normal U statistic approach [R19-3448]

Separate summaries for each component of this endpoint will also be presented.

Sensitivity and subgroup analyses for the primary endpoint will be specified in the TSAP.

The method of handling missing KCCQ-TSS values for this analysis is described in Section 7.3.

7.2.3 Secondary endpoint analyses

Secondary endpoints will not be tested in a hierarchical sequence, and no adjustment for multiple comparisons is planned.

Change from baseline in continuous endpoints, such as KCCQ-TSS, will be analysed using restricted maximum likelihood estimation based on a mixed-effect model for repeated measures (MMRM) analysis to obtain adjusted means for the treatment effects. This model will include discrete fixed effects for treatment group (empagliflozin or placebo) and HF status (de novo or decompensated chronic HF) at each visit and continuous fixed effects for baseline value at each visit. Missing data caused by patient withdrawal or other reasons will be handled implicitly by the MMRM approach.

Area under the curve (AUC) of change from baseline in log-transformed NT-proBNP level over 30 days of treatment will be analysed by an analysis of covariance (ANCOVA). Based on literature reviews, NT-proBNP level is regarded as log-normally distributed, therefore values will be log-transformed prior to analysis [R19-3044]. The linear trapezoidal rule will be used to calculate the AUC after the log-transformation has been applied to each value. Analysis of covariance (ANCOVA) with a discrete fixed effect for HF status (de novo or decompensated chronic HF) and a continuous fixed effect for baseline NT-proBNP level (log-transformed) will be used to compare treatment groups. The method of handling missing NT-proBNP levels for this analysis is described in Section 7.3.

Comparisons between treatment groups regarding the binary endpoint variable (improvement in KCCQ-TSS of $\geq$ 10 points after 90 days of treatment) will be performed using a logistic regression model adjusting for the binary covariate HF status (de novo or decompensated chronic HF). The likelihood-ratio test will be used to test for a difference between treatments. Adjusted odds ratios together with 1-sided 97.5% confidence limits will be used to quantify the effect of treatment, comparing empagliflozin to placebo as the reference.

Proprietary confidential information © 2020 Boehringer Ingelheim International GmbH or one or more of its affiliated companies

Time to event endpoints will be analysed using the Cox proportional hazards model [R07-4680] with HF status (de novo or decompensated chronic HF) as a covariate. Hazard ratios (HRs) and their associated one-sided 97.5% confidence limits will be estimated for evaluating the superiority of empagliflozin to placebo.

Other secondary endpoints will be summarised descriptively (including days alive and out of hospital).

Analysis of recurrent events will be described in the TSAP.

7.2.4 Further endpoint analyses

Change from baseline in continuous endpoints will be analysed using the same methods as for secondary endpoints.

Comparisons between treatment groups regarding the binary endpoint variables will follow the same approach as for secondary endpoints.

All other further endpoints will be summarised descriptively.

7.2.5 Safety analyses

Adverse events will be coded using the Medical Dictionary for Drug Regulatory Activities (MedDRA). Standard BI summary tables and listings will be produced. All adverse events with an onset between start of treatment and end of the residual effect period (REP), a period of 7 days after the last dose of trial medication, will be assigned to the on-treatment period for evaluation.

All treated patients will be included in the safety analysis. In general, safety analyses will be descriptive in nature and will be based on BI standards. No hypothesis testing is planned.

Statistical analysis and reporting of adverse events will concentrate on treatment-emergent adverse events, i.e. all adverse events occurring between start of treatment and end of the residual effect period. Adverse events that start before first drug intake and deteriorate under treatment will also be considered as 'treatment-emergent'.

Frequency, severity, and causal relationship of adverse events will be tabulated by system organ class and preferred term after coding according to the current version of the Medical Dictionary for Drug Regulatory Activities (MedDRA) at the database lock.

Laboratory data will be analysed both quantitatively as well as qualitatively. The latter will be done via comparison of laboratory data to their reference ranges. Values outside the reference range as well as values defined as clinically relevant will be summarised. Treatment groups will be compared descriptively with regard to distribution parameters as well as with regard to frequency and percentage of patients with abnormal values or clinically relevant abnormal values.

Proprietary confidential information © 2020 Boehringer Ingelheim International GmbH or one or more of its affiliated companies

Vital signs, physical examinations, or other safety-relevant data observed at screening, baseline, during the course of the trial and at the end-of-trial evaluation will be assessed with regard to possible changes compared to findings before start of treatment.

Reasons for discontinuation and use of post-baseline concomitant medications will be tabulated.

The details of the analysis will be specified in the TSAP.

7.2.6 Interim Analyses

No interim analysis is planned but a Data Monitoring Committee (DMC) will be in place with tasks as described in Section 8.7.

7.3 HANDLING OF MISSING DATA

Missing data for KCCQ-TSS and NT-proBNP will be estimated using multiple imputation, according to whether patients are on-treatment or off-treatment.

Further details on multiple imputation and rules for handling missing data for other secondary and further endpoints will be specified in the TSAP.

7.4 RANDOMISATION

The trial will be performed as a double-blind design with respect to placebo and empagliflozin. Patients will be randomised to the trial treatments in a 1:1 ratio. Patients will be stratified by HF status (de novo or decompensated chronic HF).

Patients will be randomised in blocks to double-blind treatment via an IRT system.

Approximately equal numbers of patients will be randomised to each treatment group.

BI will arrange for the randomisation and the packaging and labelling of trial medication. The randomisation list will be generated using a validated system, which involves a pseudo-random number generator so that the resulting treatment will be both reproducible and non-predictable. The block size will be documented in the Clinical Trial Report. Access to the codes will be controlled and documented.

7.5 DETERMINATION OF SAMPLE SIZE

Powers for superiority in were estimated by simulation using a shared frailty approach. Initially a frailty value was simulated for each patient using a gamma distribution. Deaths were assumed to have constant hazard for any given patient dependent upon the frailty term, leading to a marginal Lomax distribution. HFEs were assumed to have a Poisson distribution within patients, again depending on the frailty parameter, leading to a marginal negative binomial distribution. The number of HFEs per patient was nominally capped at 7 per patient over the 90 day period. For KCCQ-TSS, the frailty terms were directly transformed into a capped normal distribution (values below 0 or above 100 set to 0 and 100 respectively). High frailties corresponded to low KCCQ-TSS scores and vice versa. The use of a shared frailty

Proprietary confidential information © 2020 Boehringer Ingelheim International GmbH or one or more of its affiliated companies

parameter ensures correlation between components of the primary endpoint so that patients with poor outcomes in one component are also more likely to have a poor outcome in the others. Ten thousand simulations were used for each scenario. Calculations of the win ratio were performed using the generalised pairwise comparisons method from Section 7.2.2. Simulations and calculations were performed using R statistical software Version 3.5.1.

A sample size of 500 randomised patients in total (250 per treatment group) would provide 87.4% power to detect a difference between the empagliflozin treatment group and placebo treatment group, in the win ratio, at a one-sided significance level of 0.025, assuming the following:

Proportion of patients who die within 90 days = 5% [R19-2932]

Hazard ratio for death component = 0.8 [P15-09840]

Proportion of patients with at least one HFE within 90 days = 15% [R19-2932]

Hazard ratio for HFE component = 0.7 [P15-09840]

KCCQ-TSS placebo mean = 55 [R17-3082]

KCCQ-TSS baseline adjusted standard deviation = 20 [R17-3097]

KCCQ-TSS treatment effect = 6 [R19-3124]

KCCQ-TSS win threshold = 5 [R17-2687]

Table 7.5: 1 Power estimates for various combinations of KCCQ-TSS treatment effects and death/HFE hazard ratios with 500 patients in total

HR for death	HR for HFE	KCCQ-TSS treatment effect			
		4	5	6	7
0.7	0.7	66.9%	80.1%	89.4%	94.7%
0.8	0.7	63.6%	77.2%	**87.4%**	93.4%
0.8	0.8	54.6%	68.8%	81.3%	89.7%

Proprietary confidential information © 2020 Boehringer Ingelheim International GmbH or one or more of its affiliated companies

8. INFORMED CONSENT, TRIAL RECORDS, DATA PROTECTION, PUBLICATION POLICY, AND ADMINISTRATIVE STRUCTURE

The trial will be carried out in compliance with the protocol, the ethical principles laid down in the Declaration of Helsinki, in accordance with the ICH Harmonized Guideline for Good Clinical Practice (GCP), relevant BI Standard Operating Procedures (SOPs), the EU directive 2001/20/EC / EU regulation 536/2014 and other relevant regulations. Investigators and site staff must adhere to these principles. Deviation from the protocol, the principles of ICH GCP or applicable regulations as will be treated as “protocol deviation”.
Standard medical care (prophylactic, diagnostic and therapeutic procedures) remains the responsibility of the treating physician of the patient.

The investigator will inform the sponsor immediately of any urgent safety measures taken to protect the trial patients against any immediate hazard, as well as of any serious breaches of the protocol or of ICH GCP.

The Boehringer Ingelheim transparency and publication policy can be found on the following web page: trials.boehringer-ingelheim.com. The rights of the investigator and of the sponsor with regard to publication of the results of this trial are described in the investigator contract. As a rule, no trial results should be published prior to finalisation of the Clinical Trial Report.

The certificate of insurance cover is made available to the investigator and the patients, and is stored in the ISF.

8.1 TRIAL APPROVAL, PATIENT INFORMATION, INFORMED CONSENT

This trial will be initiated only after all required legal documentation has been reviewed and approved by the respective Institutional Review Board (IRB / Independent Ethics Committee (IEC and competent authority (CA) according to national and international regulations. The same applies for the implementation of changes introduced by amendments.

Prior to patient participation in the trial, written informed consent must be obtained from each patient (or the patient’s legally accepted representative) according to ICH-GCP and to the regulatory and legal requirements of the participating country. Each signature must be personally dated by each signatory and the informed consent and any additional patient-information form retained by the investigator as part of the trial records. A signed copy of the informed consent and any additional patient information must be given to each patient or the patient’s legally accepted representative.”

The patient must be given sufficient time to consider participation in the trial. The investigator or delegate obtains written consent of the patient’s own free will with the informed consent form after confirming that the patient understands the contents. The investigator or his delegate must sign (or place a seal on) and date the informed consent form. If a trial collaborator has given a supplementary explanation, the trial collaborator also signs (or places a seal on) and dates the informed consent.

Proprietary confidential information © 2020 Boehringer Ingelheim International GmbH or one or more of its affiliated companies

Re-consenting may become necessary when new relevant information becomes available and should be conducted according to the sponsor's instructions.

If study conduct may need to be adjusted (see Sections 6.1 and 10.5) during the COVID-19 pandemic, the patient must be made aware of any modifications and agreement needs to be obtained prior to them being implemented.

The consent and re-consenting process should be properly documented in the source documentation.

8.2 DATA QUALITY ASSURANCE

A risk-based approach is used for trial quality management. It is initiated by the assessment of critical data and processes for trial subject protection and reliability of the results as well as identification and assessment of associated risks. An Integrated Quality and Risk Management Plan documents the rationale and strategies for risk management during trial conduct including monitoring approaches, vendor management and other processes focusing on areas of greatest risk.

Continuous risk review and assessment may lead to adjustments in trial conduct, trial design or monitoring approaches.

A quality assurance audit/inspection of this trial may be conducted by the sponsor, sponsor's designees, or by IRB / IEC or by regulatory authorities. The quality assurance auditor will have access to all medical records, the investigator's trial-related files and correspondence, and the informed consent documentation of this clinical trial.

8.3 RECORDS

CRFs for individual patients will be provided by the sponsor. See Section 4.1.5.2 for rules about emergency code breaks. For drug accountability, refer to Section 4.1.8.

8.3.1 Source documents

In accordance with regulatory requirements, the investigator should prepare and maintain adequate and accurate source documents and trial records that include all observations and other data pertinent to the investigation on each trial patient. Source data as well as reported data should follow the "Attributable, Legible, Contemporaneous, Original, Accurate (ALCOA) principles" and be attributable, legible, contemporaneous, original and accurate. Changes to the data should be traceable (audit trail).

Data reported on the CRF must be consistent with the source data or the discrepancies must be explained.

The current medical history of the patient may not be sufficient to confirm eligibility for the trial and the investigator may need to request previous medical histories and evidence of any diagnostic tests. In this case, the investigator must make at least one documented attempt to retrieve previous medical records. If this fails, a verbal history from the patient, documented in their medical records, would be acceptable.

Proprietary confidential information © 2020 Boehringer Ingelheim International GmbH or one or more of its affiliated companies

If the patient is not compliant with the protocol, any corrective action e.g. re-training must be documented in the patient file.

For the CRF, data must be derived from source documents, for example:
- Patient identification: gender, year of birth (in accordance with local laws and regulations).
- Patient participation in the trial (substance, trial number, patient number, date patient was informed).
- Dates of patient's visits, including dispensing of trial medication.
- Medical history (including trial indication and concomitant diseases, if applicable).
- Medication history.
- Adverse events and outcome events (onset date (mandatory), and end date (if available)).
- Serious adverse events (onset date (mandatory), and end date (if available)).
- Concomitant therapy (start date, changes).
- Originals or copies of laboratory results and other imaging or testing results, with proper documented medical evaluation (in validated electronic format, if available).
- Supporting documents and medical records for assessment of death and HFEs.
- Completion of patient's participation in the trial" (end date; in case of premature discontinuation document the reason for it).
- Prior to allocation of a patient to a treatment into a clinical trial, there must be documented evidence in the source data (e.g. medical records) that the trial participant meets all inclusion criteria and does not meet any exclusion criteria. The absence of records (either medical records, verbal documented feedback of the patient or testing conducted specific for a protocol) to support inclusion/exclusion criteria does not make the patient eligible for the clinical trial.

8.3.2 Direct access to source data and documents

The investigator /institution will allow site trial-related monitoring, audits, IRB / IEC review and regulatory inspections. Direct access must be provided to the CRF and all source documents/data, including progress notes, copies of laboratory and medical test results, which must be available at all times for review by the CRA, auditor and regulatory inspector (e.g. FDA). They may review all CRFs and informed consents. The accuracy of the data will be verified by direct comparison with the source documents described in Section 8.3.1. The sponsor will also monitor compliance with the protocol and GCP.

8.3.3 Storage period of records

Trial site(s):
The trial site(s) must retain the source and essential documents (including ISF) according to contract or the local requirements valid at the time of the end of the trial (whatever is longer).

Sponsor:
The sponsor must retain the essential documents according to the sponsor's SOPs.

Proprietary confidential information © 2020 Boehringer Ingelheim International GmbH or one or more of its affiliated companies

8.4 EXPEDITED REPORTING OF ADVERSE EVENTS

BI is responsible to fulfil their legal and regulatory reporting obligation in accordance with regulatory requirements.

8.5 STATEMENT OF CONFIDENTIALITY AND PATIENT PRIVACY

Data protection and data security measures are implemented for the collection, storage and processing of patient data in accordance with the principles 7 and 12 of the World Health Organisation (WHO) GCP handbook.
Individual patient data obtained as a result of this trial is considered confidential and disclosure to third parties is prohibited with the following exceptions:
Personalised treatment data may be given to the patient's personal physician or to other appropriate medical personnel responsible for the patient's welfare. Data generated at the site as a result of the trial need to be available for inspection on request by the participating physicians, the sponsor's representatives, by the IRB / IEC and the regulatory authorities.

8.6 TRIAL MILESTONES

The **start of the trial** is defined as the date when the first patient in the whole trial signs informed consent.

The end of the trial is defined as the date of the last visit of the last patient in the whole trial ("Last Patient Completed"). The "**Last Patient Last Treatment"** (LPLT) date is defined as the date on which the last patient in the whole trial is administered the last dose of trial treatment (as scheduled per protocol or prematurely). Individual investigators will be notified of SUSARs occurring with the trial medication until 30 days after LPLT at their site.

Early termination of the trial is defined as the premature termination of the trial due to any reason before the end of the trial as specified in this protocol.

Temporary halt of the trial is defined as any unplanned interruption of the trial by the sponsor with the intention to resume it.

Suspension of the trial is defined as an interruption of the trial based on a Health Authority request.

The IEC / competent authority in each participating EU member state will be notified about the trial milestones according to the respective laws.

A final report of the clinical trial data will be written only after all patients have completed the trial in all countries (EU or non-EU) to incorporate and consider all data in the report.

The sponsor will submit to the EU database a summary of the final trial results within one year from the end of a clinical trial as a whole, regardless of the country of the last patient (EU or non-EU).

Proprietary confidential information © 2020 Boehringer Ingelheim International GmbH or one or more of its affiliated companies

8.7 ADMINISTRATIVE STRUCTURE OF THE TRIAL

The trial is sponsored by Boehringer Ingelheim (BI).

Two Coordinating Investigators are responsible to coordinate investigators at the different sites participating in this trial. Tasks and responsibilities are defined in a contract.

An Executive Committee (ExCom) consisting of independent experts (including the Coordinating Investigators of this trial) and sponsor representatives will be established to support the sponsor in designing the trials and successful execution. The composition of the ExCom will be documented in the Trial Master File (TMF). The tasks and responsibilities will be agreed in contracts between the ExCom members and the Sponsor and also summarised in an ExCom charter. The Executive Committee will assess the baseline characteristics of the patients in an ongoing blinded manner and if needed, may take appropriate steps, which may include restrictions to enrolment for certain subpopulations.

A National Coordinator Committee (NCC) will be established and will consist of the leading expert(s) in each of the participating countries. The NCs will support the Sponsor in the successful execution of the trial. The NCC will have an advisory function in the trial. The tasks and responsibilities will be agreed in contracts between the NCC member and the Sponsor.

A Data Monitoring Committee (DMC) will be established. Members of the DMC are independent of BI, they are physicians experienced in the treatment of the disease under investigation and a statistician. The DMC will evaluate safety data. While DMC members may be unblinded, measures are in place to ensure the blinding for everyone else involved in the trial. Regular DMC meetings will be held at specified intervals. The DMC will recommend continuation, modification or termination of the trial as detailed in the DMC charter. DMC recommendations as well as the final BI decision will be reported to the appropriate Regulatory Authorities (RAs)/ Health Authority (Has), IRBs/ECs, and to investigators as requested by local law. The tasks and responsibilities of the DMC are specified in a charter.

An independent external committee (Clinical Event Committee (CEC)) will be established to adjudicate certain hepatic events and ketoacidosis.

Hepatic External adjudication for hepatic events

Certain hepatic events will be adjudicated by external independent experts for severity and causal relationship with the trial medication; both in a blinded fashion. Events to be reviewed will be defined in a charter.

Events may either be defined by abnormal laboratory values and/or relevant adverse events or both.

For qualifying events, relevant source documents generated from any medical evaluations of these events will be requested including for example laboratory values, histological analysis, reports from ultrasound, computed tomography (CT), magnetic resonance imaging (MRI),

Proprietary confidential information © 2020 Boehringer Ingelheim International GmbH or one or more of its affiliated companies

scintigraphy, hospital discharge letters, and medical reports from other physicians. All evaluations will be performed in a blinded fashion. The assessments will be analysed based on empagliflozin data combined from multiple trials (i.e. on project level).

Adjudication of ketoacidosis

Events suspected to be metabolic acidosis, ketoacidosis and DKA will be adjudicated by independent external experts in a blinded fashion. Events to be reviewed will be defined in a charter.

Relevant documentation on the participating (Principal) Investigators (e.g. their curricula vitae) will be filed in the ISF. The investigators will have access to the BI clinical trial portal (Clinergize) to facilitate document exchange.

BI has appointed a Clinical Trial Leader (CT Leader), responsible for coordinating all required activities, in order to

- manage the trial in accordance with applicable regulations and internal SOPs,
- direct the clinical trial team in the preparation, conduct, and reporting of the trial,
- ensure appropriate training and information of CT Managers, CRAs, and investigators of participating countries.

The organisation of the trial in the participating countries will be performed by the respective local or regional BI-organisation (Operating Unit, OPU) in accordance with applicable regulations and BI SOPs, or by a Contract Research Organisation (CRO) with which the responsibilities and tasks will have been agreed and a written contract filed before initiation of the clinical trial.

Data Management and Statistical Evaluation will be done by BI according to BI SOPs.

Tasks and functions assigned in order to organise, manage, and evaluate the trial are defined according to BI SOPs. A list of responsible persons and relevant local information can be found in the ISF.
A central laboratory service and an IRT vendor will be used in this trial. Details will be provided in the IRT Manual and Central Laboratory Manual, available in the ISF.

Proprietary confidential information © 2020 Boehringer Ingelheim International GmbH or one or more of its affiliated companies

9. REFERENCES

9.1 PUBLISHED REFERENCES

P13-04190 Seman L, Macha S, Nehmiz G, Simons G, Ren B, Pinnetti S, et.al. Empagliflozin (BI 10773), a potent and selective SGLT2 inhibitor, induces dose dependent glucosuria in healthy subjects. Clin Pharmacol Drug Dev. 2013. 2(2): 152-161.

P13-06404 Yancy CW, et.al. 2013 ACCF/AHA Guideline for the Management of Heart Failure. Circulation. 2013. 128: e240-e327.

P14-01668 Cherney DZI, Perkins BA, Soleymanlou N, Har R, Fagan N, Johansen OE, et.al. The effect of empagliflozin on arterial stiffness and heart rate variability in subjects with uncomplicated type 1 diabetes mellitus. Cardiovasc Diabetol. 2014. 13(28).

P15-00589 Inzucchi SE, Zinman B, Wanner C, Ferrari R, Fitchett D, Hantel S, et.al. SGLT-2 inhibitors and cardiovascular risk: proposed pathways and review of ongoing outcome trials. Diabetes Vasc Dis Res. 2015. 12(2): 90-100.

P15-09541 Chilton R, Tikkanen I, Cannon CP, Crowe S, Woerle HJ, Broedl UC, et.al. Effects of empagliflozin on blood pressure and markers of arterial stiffness and vascular resistance in patients with type 2 diabetes. Diabetes Obes Metab. 2015. 17(12): 1180-1193.

P15-09840 Zinman B, Wanner C, Lachin JM, Fitchett D, Bluhmki E, Hantel S, et.al. Empagliflozin, cardiovascular outcomes, and mortality in type 2 diabetes. New Engl J Med. 2015. 373(22): 2117-2128.

P16-01253 Fitchett D, Zinman B, Wanner C, Lachin JM, Hantel S, Salsali A, et.al. Heart failure outcomes with empagliflozin in patients with type 2 diabetes at high cardiovascular risk: results of the EMPA-REG OUTCOME trial. Eur Heart J. 2016. 37(19): 1526-1534.

P16-01830 Ferrannini E, Baldi S, Frascerra S, Astiarraga B, Heise T, Bizzotto R, et.al. Shift to fatty substrate utilization in response to sodium-glucose cotransporter 2 inhibition in subjects without diabetes and patients with type 2 diabetes. Diabetes. 2016. 65(5): 1190-1195.

P16-03952 Mozaffarian D, et.al. American Heart Association Statistics Committee and Stroke Statistics Subcommittee. Heart disease and stroke statistics - 2016 update: a report from the American Heart Association. Circulation. 2016. 133(4): e38-e360.

P16-05920 Ponikowski P, Voors AA, Anker SD, Bueno H, Cleland JGF, Coats AJS, et.al. 2016 ESC Guidelines for the diagnosis and treatment of acute and chronic heart failure: the Task Force for the Diagnosis and Treatment of Acute and Chronic Heart Failure of the European Society of Cardiology (ESC), developed with the special contribution of the Heart Failure Association (HFA) of the ESC. Eur Heart J. 2016. 37: 2129-2200.

P17-04479 Al-Jobori H, Daniele G, Cersosimo E, Triplitt C, Mehta R, Norton L, et.al. Empagliflozin and kinetics of renal glucose transport in healthy individuals and individuals with type 2 diabetes. Diabetes. 2017. 66 (7): 1999-2006.

Proprietary confidential information © 2020 Boehringer Ingelheim International GmbH or one or more of its affiliated companies

P17-10453	Wanner C, Lachin JM, Inzucchi SE, Fitchett D, Mattheus M, George J, et.al. Empagliflozin and clinical outcomes in patients with type 2 diabetes mellitus, established cardiovascular disease and chronic kidney disease. Circulation. 2018. 137(2): 119-129.
P18-10152	Inzucchi SE, Kosiborod M, Fitchett D, Wanner C, Hehnke U, Kaspers S, et.al. Improvement in cardiovascular outcomes with empagliflozin is independent of glycemic control. Circulation. 2018. 138(17): 1904-1907.
P19-01985	Savarese G, Sattar N, Januzzi J, Verma S, Lund LH, Fitchett D, et.al. Empagliflozin is associated with a lower risk of post-acute heart failure rehospitalization and mortality. Insights from the EMPA-REG OUTCOME trial. Circulation. 2019. 139(11): 1458-1460.
P19-02151	Verma S, McMurray J.J.V. SGLT2 inhibitors and mechanisms of cardiovascular benefit: a state-of-the-art review. Diabetologia. 2018. 61: 2108-2117.
R07-4680	Collett.D, Modelling Survival Data in Medical Research. A CRC Press Company. 2003.
R12-1392	Levey AS, Stevens LA, Schmid CH, Zhang Y, Castro AF, Feldman HI, et.al. Disease Epidemiology Collaboration (CKD-EPI) A new equation to estimate glomerular filtration rate. Ann Intern Med. 2009. 150(9):604-612.
R16-1528	Owan TE, Redfield MM. Epidemiology of diastolic heart failure. Progress in Cardiovascular Diseases. 2005. 47(5): 320-332.
R16-2217	Cheng RK, Cox M, Neely ML, Heidenreich PA, Bhatt DL, Eapen ZJ, et.al. Outcomes in patients with heart failure with preserved, borderline, and reduced ejection fraction in the Medicare population. Am Heart J. 2014. 168(5): 721 - 730.e3.
R16-4813	Pocock SJ, Ariti CA, Collier TJ, Wang D. The win ratio: a new approach to the analysis of composite endpoints in clinical trials based on clinical priorities. Eur Heart J. 2012. 33: 176-182.
R17-1562	Bishu K, Redfield MM. Acute heart failure with preserved ejection fraction: unique patient characteristics and targets for therapy. Curr Heart Fail Rep. 2013. 10(3): 190-197.
R17-2687	Spertus J, Peterson E, Conard MW, Heidenreich PA, Krumholz HM, Jones P, et.al. Cardiovascular Outcomes Research Consortium Monitoring clinical changes in patients with heart failure: a comparison of methods. American Heart Journal. 2005. 150(4): 707-715.
R17-3082	Sauser K, Spertus JA, Pierchala L, Davis E, Pang PS. Quality of life assessment for acute heart failure patients from emergency department presentation through 30 days after discharge: a pilot study with the Kansas City Cardiomyopathy Questionnaire. J Card Fail. 2014. 20(1): 18-22.
R17-3097	Comin-Colet J, Lainscak M, Dickstein K, Filippatos GS, Johnson P, Luescher TF, et.al. The effect of intravenous ferric carboxymaltose on health-related quality of life in patients with chronic heart failure and iron deficiency: a subanalysis of the FAIR-HF study. Eur Heart J. 2013. 34: 30-38.

Proprietary confidential information © 2020 Boehringer Ingelheim International GmbH or one or more of its affiliated companies

R19-2911 Gattis WA, O'Connor CM, Gallup DS, Hasselblad V, Gheorghiade M., et.al. Predischarge initiation of carvedilol in patients hospitalised for decompensated heart failure: results of the Initiation Management Predischarge: Process for Assessment of Carvedilol Therapy in Heart Failure (IMPACT-HF) trial. J Am Coll Cardiol. 2004. 43(9): 1534–41.

R19-2913 Logeart D, Thabut G, Jourdain P, Chavelas C, et al. Predischarge B-type natriuretic peptide assay for identifying patients at high risk of re-admission after decompensated heart failure. J Am Coll Cardiol. 2004. 43(4): 635-641.

R19-2914 Zile MR, Bennett TB, et.al. Transition from Chronic Compensated to Acute Decompensated Heart Failure: Pathophysiological insight from continuous monitoring of intracardiac Pressures. Circulation. 2008. 118. 1433-1441.

R19-2915 Gheorghiade M, Vaduganathan M, et al. Rehospitalisation for heart failure: problems and perspectives. J Am Coll Cardiol. 2013. 61(4): 391-403.

R19-2916 Torre-Amione G, Milo-Cotter O, Kaluski E, Perchenet L, Kobrin I, Frey A, et.al. Early worsening heart failure in patients admitted for acute heart failure: time course, hemodynamic predictors, and outcome. J Card Fail. 2009. 15(8): 639–644.

R19-2917 Heidenreich PA, et al. Forecasting the Impact of Heart Failure in the United States: A Policy Statement From the American Heart Association. Circ Heart Fail. 2013. 6: 606-619.

R19-2918 Blecker S, Paul M, Taksler G, Ogedegbe G, Katz S. Heart failure associated hospitalisations in the United States. J Am Coll Cardiol. 2013. 61(12): 1259-1267.

R19-2919 Butler J, Arbogast PG, Daugherty J, Jain MK, Ray WA, Griffin MR. Outpatient utilization of angiotensin-converting enzyme inhibitors among heart failure patients after hospital discharge. J Am Coll Cardiol. 2004. 43(11): 2036-2043.

R19-2920 Fonarow GC, Gawlinski A, Moughrabi S, Tillisch JH. Improved treatment of coronary heart disease by implementation of a Cardiac Hospitalisation Atherosclerosis Management Program (CHAMP). Am J Cardiol. 2001. 87(7): 819-822.

R19-2921 Grodin JL, Liebo MJ, et.al. Prognostic Implications of Changes in Amino-Terminal Pro-B-Type Natriuretic Peptide in Acute Decompensated Heart Failure: Insights from ASCEND-HF. Journal of Cardiac Failure. 2019. 00(00): 1-9.

R19-2924 Aditi A, Bhagat, et.al. Initiation, Continuation, Switching, and Withdrawal of Heart Failure Medical Therapies During Hospitalisation. JACC:HF. 2019. 7(1): 1-12.

R19-2932 Velazquez EJ, Morrow DA, DeVore AD, Duffy CI, Ambrosy AP, McCague K, et.al. PIONEER-HF Investigators. Angiotensin-neprilysin inhibition in acute decompensated heart failure. N Engl J Med. 2019. 380(6): 539-548.

R19-3040 Heidenreich PA. Patient-Reported Outcomes: The Future of Heart Failure Care. JACC HF. 2019. 7: 1-3.

R19-3044 Schou M, Gustafsson F, Kjaer A, Hildebrandt PM. Long-term clinical variation of NT-proBNP in stable chronic heart failure patients. European Heart Journal. 2007. 28(2): 177-182.

Proprietary confidential information © 2020 Boehringer Ingelheim International GmbH or one or more of its affiliated companies

R19-3124 Nassif ME, Windsor S, Tang F, Khariton Y, Husain M, Inzucchi S, et.al. Dapagliflozin Effects on Biomarkers, Symptoms, and Functional Status in Patients With Heart Failure With Reduced Ejection Fraction: The DEFINE-HF Trial. Circulation. 2019. 40(140): 1-14.

R19-3125 McMurray JJV, Solomon SD, Inzucchi L, et.al. Dapagliflozin with Patients in Heart Failure and Reduced Ejection Fraction. New England Journal of Medicine. 2019. 1-13.

R19-3126 U.S. Department of Health and Human Services, Food and Drug Administration, Center for Drug Evaluation and Research, Center for Biologics Evaluation and Research. Treatment for Heart Failure: Endpoints for Drug Development Guidance for Industry. June 2019.

R19-3245 Hicks KA, Mahaffey KW, Mehran R, Nissen SE, Wiviott SD, et.al. 2017 Cardiovascular and Stroke Endpoint Definitions for Clinical Trials. Journal of the American College of Cardiology. 2018. 71(9): 1021-34.

R19-3448 Dong G, Li D, Ballerstedt S, Vandemeulebroecke M. A generalized analytic solution to the win ratio to analyze a composite endpoint considering the clinical importance order among components. Pharm Stat. 2016. 15: 430-437.

9.2 UNPUBLISHED REFERENCES

c02695839-01 Devins T, Espadero RM, Frampton H, Koeppen M, Mattheus M, Joseph D. Clinical Trial Report: Empagliflozin (BI 10773). The EMPA-REG OUTCOME® Trial 1245.25.

c01678844 Investigators Brochure. Empagliflozin. Current Version.

c11963611-01 Lewis-D'Agostino D, Fagan N, Wu J, Stanley J. Clinical Trial Report: Jardiance® (empagliflozin). An open-label, mechanistic study to examine the effect of oral empagliflozin (25 mg q.d.) on kinetics of renal glucose reabsorption in patients with type 2 diabetes mellitus and healthy controls. Trial 1245.66.

c26554599-01 Macesic H, Haug M, Peil B. Clinical Trial Report: A phase III randomised, double-blind trial to evaluate the effect of 12 weeks treatment of once daily EMPagliflozin 10 mg compared with placebo on ExeRcise ability and heart failure symptoms, In patients with chronic HeArt FaiLure with preserved Ejection Fraction (HFpEF) (EMPERIAL – preserved). Trial 1245-0167

c26554767-01 Brun M, Teodorczyk M, Peil B. Clinical Trial Report: A phase III randomised, double-blind trial to evaluate the effect of 12 weeks treatment of once daily EMPagliflozin 10 mg compared with placebo on ExeRcise ability and heart failure symptoms, In patients with chronic HeArt FaiLure with reduced Ejection Fraction (HFrEF) (EMPERIAL – reduced). Trial 1245-0168

Proprietary confidential information © 2020 Boehringer Ingelheim International GmbH or one or more of its affiliated companies

10. APPENDICES

10.1 KCCQ (KANSAS CITY CARDIOMYOPATHY QUESTIONNAIRE)

THE KANSAS CITY CARDIOMYOPATHY QUESTIONNAIRE:

The following questions refer to your **heart failure** and how it may affect your life. Please read and complete the following questions. There are no right or wrong answers. Please mark the answer that best applies to you.

1. **Heart failure** affects different people in different ways. Some feel shortness of breath while others feel fatigue. Please indicate how much you are limited by **heart failure** (shortness of breath or fatigue) in your ability to do the following activities over the past 2 weeks.

Place an X in one box on each line

Activity	Extremely Limited	Quite a bit Limited	Moderately Limited	Slightly Limited	Not at all Limited	Limited for other reasons or did not do the activity
Dressing yourself	❑	❑	❑	❑	❑	❑
Showering/Bathing	❑	❑	❑	❑	❑	❑
Walking 1 block on level ground	❑	❑	❑	❑	❑	❑
Doing yardwork, housework or carrying groceries	❑	❑	❑	❑	❑	❑
Climbing a flight of stairs without stopping	❑	❑	❑	❑	❑	❑
Hurrying or jogging (as if to catch a bus)	❑	❑	❑	❑	❑	❑

2. Compared with 2 weeks ago, have your symptoms of **heart failure** (shortness of breath, fatigue or ankle swelling) changed?
My symptoms of **heart failure** have become . . .

Much worse	Slightly worse	Not changed	Slightly better	Much better	I've had no symptoms over the last 2 weeks
❑	❑	❑	❑	❑	❑

3. Over the past 2 weeks, how many times did you have **swelling** in your feet, ankles or legs when you woke up in the morning?

Every morning	3 or more times a week, but not every day	1–2 times a week	Less than once a week	Never over the past 2 weeks
❑	❑	❑	❑	❑

4. Over the past 2 weeks, how much has **swelling** in your feet, ankles or legs bothered you?
It has been . . .

**Extremely** bothersome	**Quite a bit** bothersome	**Moderately** bothersome	**Slightly** bothersome	**Not at all** bothersome	I've had **no swelling**
❑	❑	❑	❑	❑	❑

5. Over the past 2 weeks, on average, how many times has **fatigue** limited your ability to do what you want?

All of the time	Several times per day	At least once a day	3 or more times per week but not every day	1–2 times per week	Less than once a week	Never over the past 2 weeks
❑	❑	❑	❑	❑	❑	❑

6. Over the past 2 weeks, how much has your **fatigue** bothered you?
It has been . . .

**Extremely** bothersome	**Quite a bit** bothersome	**Moderately** bothersome	**Slightly** bothersome	**Not at all** bothersome	I've had **no fatigue**
❑	❑	❑	❑	❑	❑

Proprietary confidential information © 2020 Boehringer Ingelheim International GmbH or one or more of its affiliated companies

7. Over the past 2 weeks, on average, how many times has **shortness of breath** limited your ability to do what you wanted?

All of the time	Several times per day	At least once a day	3 or more times per week but not every day	1–2 times per week	Less than once a week	Never over the past 2 weeks
❑	❑	❑	❑	❑	❑	❑

8. Over the past 2 weeks, how much has your **shortness of breath** bothered you?
It has been . . .

**Extremely** bothersome	**Quite a bit** bothersome	**Moderately** bothersome	**Slightly** bothersome	**Not at all** bothersome	I've had **no shortness of breath**
❑	❑	❑	❑	❑	❑

9. Over the past 2 weeks, on average, how many times have you been forced to sleep sitting up in a chair or with at least 3 pillows to prop you up because of **shortness of breath**?

Every night	3 or more times a week, but not every day	1–2 times a week	Less than once a week	Never over the past 2 weeks
❑	❑	❑	❑	❑

10. **Heart failure** symptoms can worsen for a number of reasons. How sure are you that you know what to do, or whom to call, if your **heart failure** gets worse?

**Not at all** sure	**Not very** sure	**Somewhat** sure	**Mostly** sure	**Completely** sure
❑	❑	❑	❑	❑

11. How well do you understand what things you are able to do to keep your **heart failure** symptoms from getting worse? (for example, weighing yourself, eating a low salt diet, etc.)

Do not understand at all	Do not understand very well	Somewhat understand	Mostly understand	Completely understand
❑	❑	❑	❑	❑

12. Over the past 2 weeks, how much has your **heart failure** limited your enjoyment of life?

It has **extremely** limited my enjoyment of life	It has limited my enjoyment of life **quite a bit**	It has **moderately** limited my enjoyment of life	It has **slightly** limited my enjoyment of life	It has **not limited** my enjoyment of life at all
❑	❑	❑	❑	❑

13. If you had to spend the rest of your life with your **heart failure** the way it is right now, how would you feel about this?

Not at all satisfied	Mostly dissatisfied	Somewhat satisfied	Mostly satisfied	Completely satisfied
❑	❑	❑	❑	❑

14. Over the past 2 weeks, how often have you felt discouraged or down in the dumps because of your **heart failure**?

I felt that way **all of the time**	I felt that way **most of the time**	I **occasionally** felt that way	I **rarely** felt that way	I **never** felt that way
❑	❑	❑	❑	❑

15. How much does your **heart failure** affect your lifestyle? Please indicate how your **heart failure** may have limited your participation in the following activities over the past 2 weeks.

Please place an **X** in one box on each line

**Activity**	**Severely** limited	Limited **quite a bit**	**Moderately** limited	**Slightly** limited	**Did not** limit at all	Does not apply or did not do for other reasons
Hobbies, recreational activities	❑	❑	❑	❑	❑	❑
Working or doing household chores	❑	❑	❑	❑	❑	❑
Visiting family or friends out of your home	❑	❑	❑	❑	❑	❑
Intimate relationships with loved ones	❑	❑	❑	❑	❑	❑

Proprietary confidential information © 2020 Boehringer Ingelheim International GmbH or one or more of its affiliated companies

10.2 INCLUSION OF ILLITERATE PATIENTS - KCCQ

In the event of recruiting an illiterate patient, the following process should be followed with respect to completion of the self-reported PRO:

- At each visit where the administration of the Patient Reported Outcome form is required, the trial coordinator or designated site personnel will read each of the items on the questionnaire to the patient, word for word, and without any accompanying explanation.

- The questionnaires will be provided to patients in the language or local dialect that is understood by the patient using the different language versions of the questionnaire that are part of the eCRF for the trial.

- The patient will choose the most appropriate response to the question, and indicate the response on the questionnaire by him/herself. If this is not possible, the trial coordinator or designated site personnel or patient's caregiver will indicate the response on the questionnaire based on the patient's feedback.

In the same way as for all other patients, the completion of the questionnaires should be performed in a quiet area where the patient can consider his/her responses.

10.3 PATIENT GLOBAL IMPRESSION OF SEVERITY OF HEART FAILURE SYMPTOMS: SHORTNESS OF BREATH, FATIGUE AND SWELLING

Please choose one response below that best describes your most recent experience of Heart Failure Symptoms: shortness of breath, fatigue and swelling:

☐ Not at all
☐ Mild
☐ Moderate
☐ Severe
☐ Very severe

10.4 ACCEPTED FORMS OF CONTRACEPTION FOR PATIENTS IN ITALY

Acceptable methods include consistent and correct use of hormone containing implants and injection contraceptives, combined oral contraceptives, transdermal patches, hormone containing intrauterine devices or systems, bilateral tubal occlusion, complete sexual abstinence, and vasectomy. No other methods will be accepted.

10.5 POTENTIAL MODIFICATION OF TRIAL CONDUCT IN CASE OF RESTRICTIONS DUE TO COVID-19

As mentioned in Section 6.1, in case of any restrictions during the COVID-19 pandemic, study conduct may need to be adjusted. The following contingency measures have been introduced to ensure patient safety and appropriate trial continuation based on a thorough

Proprietary confidential information © 2020 Boehringer Ingelheim International GmbH or one or more of its affiliated companies

benefit risk assessment (see Section 1.4.2).

In exceptional cases, when it is impossible to conduct the visits at the trial site, visits may be performed at the patient's home or remotely (via telephone and/or internet based means of communication). The visits may also be performed as a combination of home and remote visits. Based on a thorough benefit-risk assessment (see Section 1.4.2), the visit procedures may be adjusted for the purpose of particular visits, whereby critical safety measures will remain in place. All home/remote visits need to be discussed with and approved by the sponsor`s trial team.
Local regulatory and legal requirements of the participating country need to be respected for all modifications.

Under these circumstances, the below modifications can be considered. Patients need to be informed about the modifications and agree to them before implementation (see Section 8.1):

Remote visit

If a patient is not able to come to the site for an outpatient visit, a remote visit (by phone) should be performed instead and all assessments that can be done by phone performed.

Assessments that can be performed during a remote visit are:
NYHA Classification, HCRU, parts of the congestion score (Dyspnoea, Orthopnoea, Fatigue), Adverse Events, concomitant therapy, Patient Global Impression of Severity of Heart Failure Symptoms (PGI-S).

KCCQ is a questionnaire that should be answered by the patient alone. Patients can be supplied with the questionnaire to be filled out at home. The questionnaire should be answered on the day of the remote visit and sent back to the site. The paitent needs to be instructed to go alone to a quiet area where she/he can record her/his response in the questionnaire without interaction with others. In instances where a patient cannot give or decide upon a response, no response should be recorded. For illiterate patients the the process described in Section 10.2 should be followed during the phone visit.

Safety lab, other laboratory tests

If taking blood samples for central lab is not possible, blood analysis for safety lab can be done in a local lab. The results of the lab tests are to be reported and transferred to the investigator, who has to ensure medical review and proper documentation.
Minimum required safety lab parameters are eGFR and liver enzymes which will be captured in the eCRF. An urine analysis should not be performed locally.

Dispensation of Trial medication (IMP)

If a patient is not able to come to visit 4 as planned but the investigator considers it favorable and save for patient to continue with IMP, IMP can be shipped from site directly to the patient (if legally acceptable according to local regulations).

Proprietary confidential information © 2020 Boehringer Ingelheim International GmbH or one or more of its affiliated companies

If home visits by trial staff members or e.g. “Home Healthcare Nurse” are possible, further assessments can be done like e.g. vital sings, blood draw for safety lab and biomarkers as well as collection of urine samples to be sent to central lab, or compliance check.

Proprietary confidential information © 2020 Boehringer Ingelheim International GmbH or one or more of its affiliated companies

11. DESCRIPTION OF GLOBAL AMENDMENT(S)

11.1 GLOBAL AMENDMENT 1

**Date of amendment**		04 Jun 2020
**EudraCT number**<br>**EU number**		2019-002946-19
**BI Trial number**		1245-0204
**BI Investigational Product(s)**		Jardiance®, empagliflozin
**Title of protocol**		A multicentre, randomised, double-blind, 90-day superiority trial to evaluate the effect on clinical benefit, safety and tolerability of once daily oral **EMP**agliflozin 10 mg compared to placebo, initiated in patients hospitalised for ac**U**te heart fai**L**ure (de novo or decompensated chronic HF) who have been **S**tabilis**E**d (**EMPULSE**)
**Global Amendment due to urgent safety reasons**		
**Global Amendment**		X
**Section to be changed**		Title page
**Description of change**		In the title “decompensatied” was changed to “decompensated”
**Rationale for change**		Correct typo
**Section to be changed**		Title page and CTP Synopsis
**Description of change**		Christiane E. Angermann, MD<br>Professor of Clinical Research in Cardiology<br>Comprehensive Heart Failure Centre<br>Deutsches Zentrum für Herzinsuffiziens Würzburg<br>Changed to<br>Christiane E. Angermann, MD<br>Professor of Clinical Research in Cardiology<br>Comprehensive Heart Failure Centre<br>University & University Hospital Würzburg
**Rationale for change**		Address corrected
**Section to be changed**		CTP Synopsis (Trial objective(s) and Trial endpoints),<br>Section 2.1 (Main Objectives, Primary and Secondary Endpoints) and<br>Section 2.2.2 (Further Endpoints)
**Description of change**		Exchange KCCQ-CSS by KCCQ-TSS in trial objective(s) and all respective endpoints (primary, secondary and further endpoints)

Proprietary confidential information © 2020 Boehringer Ingelheim International GmbH or one or more of its affiliated companies

		Exchange KCCQ-TSS by KCCQ-CSS in the respective further endpoints
**Rationale for change**		Based on results from EMPERIAL-reduced and other SGLT2-inhibitor trials (with dapagliflozin), total symptom score of KCCQ (KCCQ-TSS) was found to be more responsive to the patient's symptoms. The EMPERIAL-reduced trial has demonstrated a slightly greater effect of empagliflozin on the TSS subdomain compared to the CSS domain. TSS is comprised of "symptom frequency" and "symptom burden", whereas CSS also includes "physical limitation", where empagliflozin showed a neutral result in EMPERIAL (also supported by the result of the primary endpoint, the 6 minute walking test distance). Therefore, the decision to change KCCQ-CSS to KCCQ-TSS was to be more clinically relevant and increase the likelihood of success.
**Section to be changed**		CTP Synopsis (Primary Endpoint and Statistical methods), Section 2.1.2.
**Description of change**		Change in statistical methodology for primary endpoint: change from "net benefit" to "win ratio". Clarification of how the variance will be calculated by providing a more specific description.
**Rationale for change**		Based on recent publications and public scientific discussions involving representatives from FDA and other regulatory authorities, the win ratio has gained attention in the scientific community. Compared to the "net benefit" approach, the result arising from the "win ratio" will likely be better understood by regulatory authorities and by the scientific community.<br>The overall statistical power for the primary endpoint will remain very similar with the win ratio.
**Section to be changed**		Flow Chart, Section 2.2.2, Section 5.1.5 and Section 10.3
**Description of change**		Patient global impression of severity (PGI-S) of heart failure symptoms is added as an patient reported outcome measure. Time points of evaluation are the same than for KCCQ.<br>The following respective further endpoint is added in Section 2.2.2:<br>Change from baseline in Patient Global Impression

Proprietary confidential information © 2020 Boehringer Ingelheim International GmbH or one or more of its affiliated companies

		of Severity of Heart Failure Symptoms after 90 days of treatment.
**Rationale for change**		Patient global impression of severity (PGI-S) of HF symptoms can be used as an anchor to identify the clinically meaningful within-patient change in KCCQ (a component of the primary endpoint). This assessment was added considering comments from FDA and other authorities on the clinical meaningfulness of the KCCQ-endpoint. The collection of PGI-S data will therefore facilitate addressing the question of clinical meaningfulness.
**Section to be changed**		Flow Chart
**Description of change**		Reference to footnote 8 added to "Review of in-/exclusion criteria"
**Rationale for change**		Clarification: local lab is used for evaluation of all in-/exclusion criteria related to lab results
**Section to be changed**		Section 1.1
**Description of change**		Wording with regard to EMPERIAL trials updated: In addition, two clinical trials in patients with chronic HFpEF (EMPERIAL-preserved) or HFrEF (EMPERIAL-reduced) to evaluate the effect of empagliflozin on exercise ability using the 6 minute walk test **are completed**.
**Rationale for change**		To reflect current status of these trials.
**Section to be changed**		Section 2.2.2
**Description of change**		Wording of endpoints changed:<br>"proportion of patients" and "percentage of patients on medication" deleted in all respective endpoints
**Rationale for change**		Endpoints should be specified at the patient level. Descriptions of summary measures to be used at the treatment group level are not necessary in this section.
**Section to be changed**		Section 3.3.2
**Description of change**		Inclusion criterion 9 was changed to<br>HF episode leading to hospitalisation must have been treated with a minimum **single** dose of 40 mg of i.v. furosemide (or equivalent i.v. loop diuretic defined as 20 mg of torasemide or 1 mg of bumetanide).
**Rationale for change**		To clarify that the dose refers to a single dose given for the treatment of the HF episode.

Proprietary confidential information © 2020 Boehringer Ingelheim International GmbH or one or more of its affiliated companies

**Section to be changed**		Section 3.3.3
**Description of change**		Exclusion criterion 14:<br>Reference to "Visit 1" is replaced by "in ECG before randomisation (Visit 2a)"
**Rationale for change**		To reflect the availability of respective trial related assessments.
**Section to be changed**		Section 3.3.3
**Description of change**		Exclusion criteria 17 and 18:<br>Reference to "Visit 1" is replaced by "as measured during hospitalisation (latest local lab measurement before randomisation)".
**Rationale for change**		To clarify which local available lab assessment should be used for assessing the exclusion criteria.
**Section to be changed**		Section 5.1.1
**Description of change**		Assessment of efficacy "Net Clinical Benefit" was changed to "Clinical Benefit".
**Rationale for change**		To reflect the changes in the statistical analysis in the naming of the assessment.
**Section to be changed**		Section 5.2.4
**Description of change**		Table 5.2.4:1 Safety laboratory tests:<br>Urinalysis: "dipstick" deleted.
**Rationale for change**		To clarify and reflect the current process. Urinalysis will be done at central lab not using dipstick tests
**Section to be changed**		Section 5.2.7.2.2
**Description of change**		The mode of sending SAE forms ("by fax") is deleted.<br>The wording is changed to:<br>The investigator must report SAEs, AESIs, and non-serious AEs which are relevant for the reported SAE or AESI, on the BI SAE form immediately (within 24 hours) to the sponsor's unique entry point (country specific reporting process will be provided in the ISF). The same timeline applies if follow-up information becomes available.<br>In specific occasions, the investigator could inform the sponsor upfront via telephone. This does not replace the requirement to complete and send the BI SAE form.
**Rationale for change**		To reflect future changes in the SAE reporting when alternative methods of SAE report

Proprietary confidential information © 2020 Boehringer Ingelheim International GmbH or one or more of its affiliated companies

		transmission to fax will be introduced for all trials.
**Section to be changed**		Section 6.2.2
**Description of change**		The order to perform the assessments was changed to:<br>Patient reported outcome measures (first KCCQ, then PGI – Severity – Heart Failure Symptoms questionnaire) should be done first and laboratory samples should be taken at the end of each visit. All other measures should be performed after PGI-S and before laboratory sampling.
**Rationale for change**		Order updated to include the added assessment of PGI-S.
**Section to be changed**		Section 7.5
**Description of change**		Changed description of primary endpoint and added the win ratio. Changed KCCQ-CSS to KCCQ-TSS. Added relevant source references for assumptions made. Added further assumptions for KCCQ-TSS placebo mean and win threshold. Updated power estimates to be based on the win ratio.
**Rationale for change**		See rationale described above for Sections 2.1, 2.1.2 and 7.2.2. Source references omitted in previous version.
**Section to be changed**		Section 9
**Description of change**		The following references are deleted:<br>R19-3016, R19-3043, R19-3046, R98-1473<br>The following references are added:<br>R16-4813, R17-3082, R17-3097, R19-3448, c26554599-01, c26554767-01
**Rationale for change**		Updated to reflect the changes in the statistical analysis.
**Section to be changed**		Section 10.4
**Description of change**		An appendix with accepted forms of contraception for patients in Italy was added
**Rationale for change**		To comply with the request form AIFA (Italian Regulatory Authority).
**Section to be changed**		Section 1.4.2
**Description of change**		<u>Risk evaluation in relation with COVID-19 added:</u><br>Patients with chronic heart diseases are at higher risk for severe illness from COVID-19. Therefore, in case of local high risk of COVID-19 infection,

Proprietary confidential information © 2020 Boehringer Ingelheim International GmbH or one or more of its affiliated companies

		physical visits to the sites should be avoided as much as possible. In the event of restriction to visit the investigator site, certain procedures can be done remotely, and local labs can be used instead of central lab. These changes are meant to keep the integrity of the trial and they will not affect the benefit-risk of empagliflozin.<br><br>There is no indication that empagliflozin may increase the risk of COVID-19 infection. As with any acute illness, empagliflozin during COVID-19 infection has the potential to increase the risk of ketoacidosis. The risk of ketoacidosis in case of acute illness is adequately addressed in the IB. The study drug should be discontinued in case of severe COVID-19 disease.
**Rationale for change**		COVID-19 pandemic
**Section to be changed**		Section 4.1.4, 6.1, 8.1 and 10.5; Flow Chart
**Description of change**		Contingency measures have been introduced to ensure patient safety and appropriate trial continuation based on a thorough benefit risk assessment (see Section 1.4.2) and above. Introduce flexibility for trial visits: to allow in exemptional cases visits to be done as home/remote visists; to allow for IMP shipment from site to patient in this cases. Patients need to agree to changes prior to them being implemented. Comment added below the Flow Chart to refer to the contingency measures if they are needed.
**Rationale for change**		COVID-19 pandemic

APPROVAL / SIGNATURE PAGE

Document Number: c28603082 **Technical Version Number:2.0**

Document Name: clinical-trial-protocol-version-02

Title: A multicentre, randomised, double-blind, 90-day superiority trial to evaluate the effect on clinical benefit, safety and tolerability of once daily oral EMPagliflozin 10 mg compared to placebo, initiated in patients hospitalised for acUte heart faiLure (de novo or decompensated chronic HF) who have been StabilisEd (EMPULSE).

Signatures (obtained electronically)

Meaning of Signature	Signed by	Date Signed
Author-Clinical Trial Leader	[redacted]	04 Jun 2020 16:02 CEST
Approval-Therapeutic Area Head		04 Jun 2020 21:50 CEST
Author-Trial Statistician		05 Jun 2020 11:25 CEST
Approval-Team Member Medicine		05 Jun 2020 16:43 CEST
Verification-Paper Signature Completion		08 Jun 2020 09:21 CEST

(Continued) Signatures (obtained electronically)

Meaning of Signature	Signed by	Date Signed
